# Aqueous Micellar Environment Impacts the Co-Catalyzed Phototransformation: A Case Study

**DOI:** 10.1021/jacs.4c02682

**Published:** 2024-07-09

**Authors:** Aleksandra Wincenciuk, Piotr Cmoch, Maciej Giedyk, Martin P. Andersson, Dorota Gryko

**Affiliations:** † Institute of Organic Chemistry Polish Academy of Sciences;, Kasprzaka 44/52, 01-224 Warsaw, Poland; ‡ Center for Integrative Petroleum Research, King Fahd University of Petroleum and Minerals, Dhahran 31261, Kingdom of Saudi Arabia

## Abstract

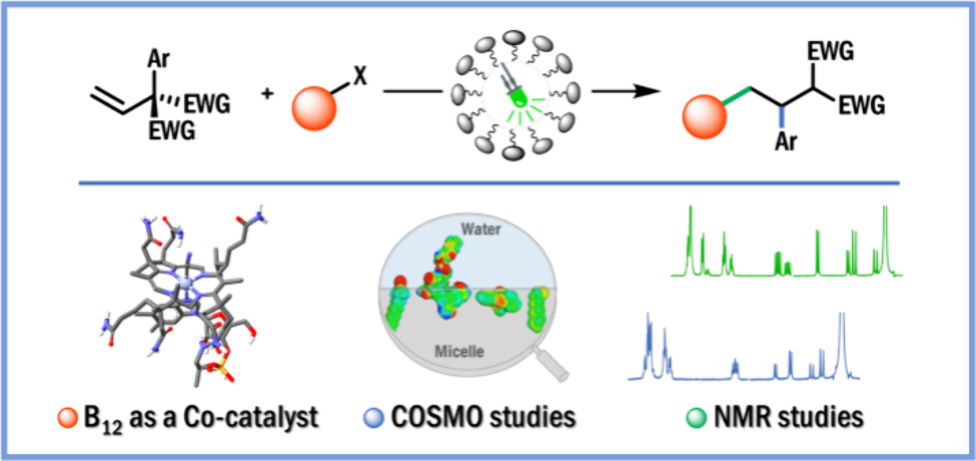

In recent years, methodologies that rely on water as the reaction medium have gained considerable attention. The unique properties of micellar solutions were shown to improve the regio-, stereo-, and chemoselectivity of different transformations. Herein, we demonstrate that the aqueous environment is a suitable medium for a visible light driven cobalt-catalyzed reaction involving radical species. In this system, reduced vitamin B_12_ reacts with alkyl halides, generating radicals that are trapped by the lipophilic olefin present in the Stern layer. A series of NMR measurements and theoretical studies revealed the location of reaction components in the micellar system.

## Introduction

Bioinspiration is a well-established approach in the field of chemistry. In contrast to biological systems where reactions take place in water-based confined compartments, water has been regarded as an unsuitable medium for reactions of lipophilic reactants. Micellar solutions do, however, allow their incorporation into the confined system, thus fostering their reactions.^1−6^ These systems, however, are not widely utilized in synthetic organic chemistry, and even less for reactions involving radicals.^7−13^ Common methods for the generation of these reactive intermediates often involve the application of precious transition metals, toxic promoters in stoichiometric amounts, or long-wavelength ultraviolet (UV) light. However, recent studies have successfully addressed this drawback; in parallel to photoredox transformations^14^ and electrochemistry,^15−17^ vitamin B_12_ catalysis has established itself as a sustainable bioinspired strategy for the generation of alkyl and acyl radicals from various molecules.^18,19^ These mainly involve alkyl (pseudo)halides, olefins, diazo compounds, strained molecules, carboxylic acid derivatives, and others.^20−24^

Most B_12_-catalyzed reactions take place in organic solvents. On the contrary, natural systems that involve vitamin B_12_ function in an aqueous, highly confined environment, ensuring excellent selectivity. Consequently, the strategy of merging B_12_ catalysis with micellar structures offers promising routes for advancing radical synthesis. Along this line, Rusling et al. have demonstrated that the electrochemical generation of the catalytically active nucleophilic Co(I) form of vitamin B_12_ can be performed in nanoreactor-type microemulsions that require the addition of an organic solvent.^25−32^ Using this strategy, dehalogenation^25^ and synthesis of bibenzyl^26,28^ and *trans*-1-decalone^31^ were achieved. In the latter case, remarkable *trans*-stereoselectivity was observed, in contrast to the homogeneous reaction in DMF. Despite these promising advances in B_12_ electrocatalysis in nanoreactor-type environments, reactions *that involve chemical reduction of vitamin B*_*12*_*in micellar solutions remain unexplored*, assumingly, because of fundamental problems: (1) Vitamin B_12_ is a water-soluble compound, while the substrates are mostly lipophilic. (2) The requirement for Zn as a reducing agent was shown to form organozinc intermediates in palladium-catalyzed cross-coupling reactions in self-assembled micelles.^33^ In addition, a fundamental understanding of reactions in micellar systems remains sparse.

**Scheme 1:**
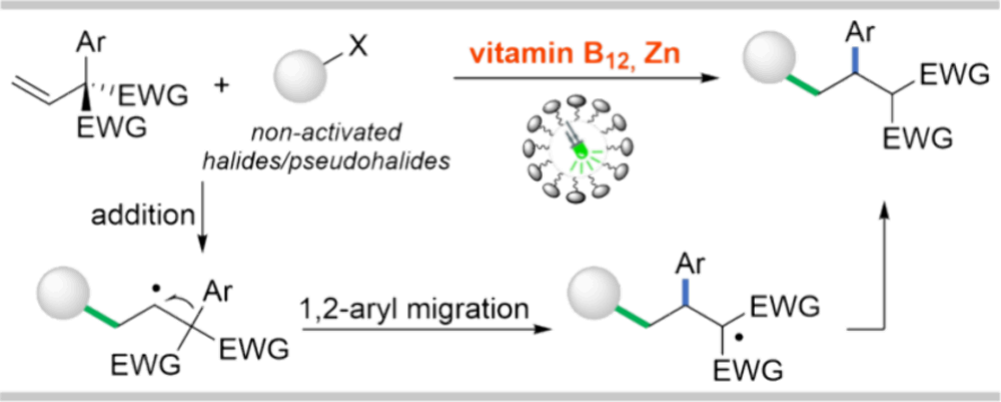
Co-Catalyzed Tandem Radical Addition/1,2-Aryl Migration: A Case Study

Herein, we report that the micellar solution is indeed a suitable medium for vitamin B_12_-catalyzed tandem radical addition/1,2-aryl migration reaction even though the catalyst is hydrophilic. The model reaction involving alkyl halides and functionalized olefins gives the desired products in good yields (Scheme 1). Experimental and theoretical studies shed light on the localization of reagents in the micellar system that allows effective reactions.

## Results and Discussion

### Model Reaction: Optimization Studies

Previous reports showed the beneficial effect of microemulsions requiring the addition of organic solvents as an oil component on vitamin B_12_-mediated electrochemical reactions.^25−32^ Consequently, we wondered whether an alternative strategy based solely on the use of surfactants would be beneficial. *The crucial issue was to find a suitable surfactant for a reaction involving a water-soluble catalyst, lipophilic starting materials, and zinc particles.* We commenced our studies on vitamin B_12_ catalysis in aqueous micellar solutions by focusing on a model tandem reaction of diethyl 2-phenyl-2-vinylmalonate (**1**) with 1-bromododecane (**2a**). In 2021 Shi and co-workers presented a mechanistically related perfluoroalkylation of vinyl-substituted quaternary centers in TFE.^34^

**Figure 1:**
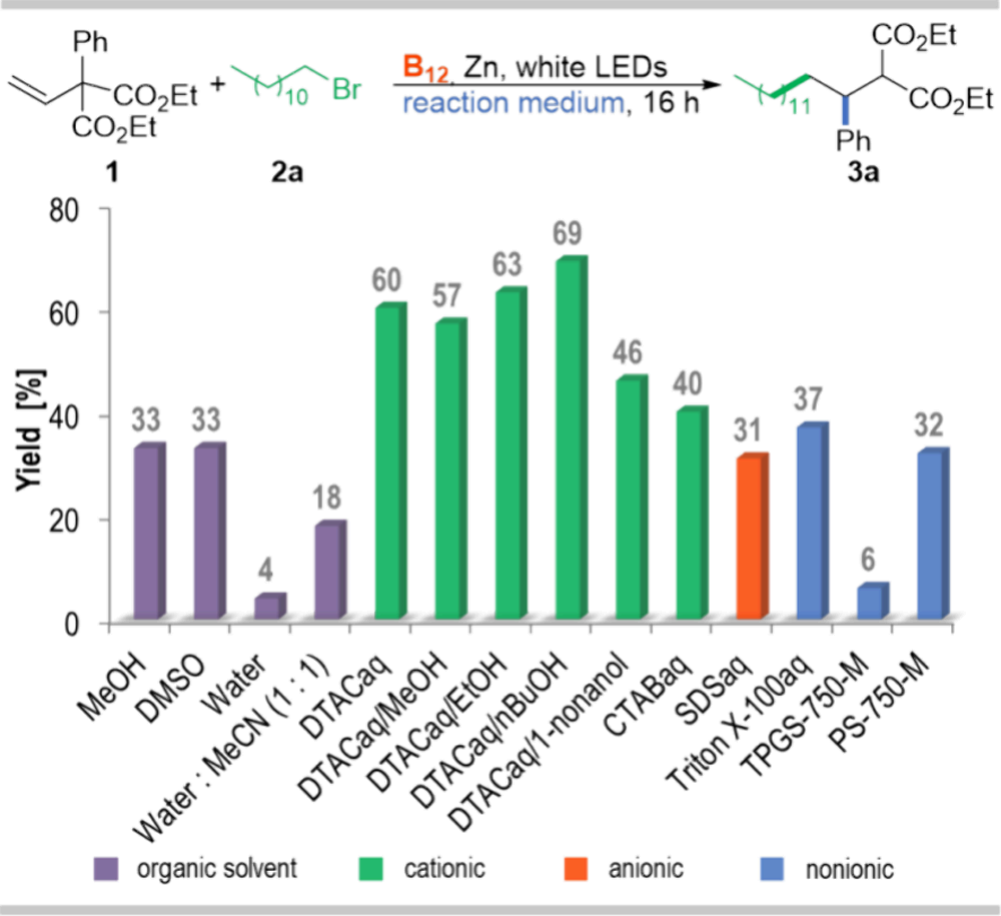
Preliminary screening of reaction media for the vitamin B_12_-catalyzed addition/1,2-phenyl migration. Reaction conditions: diethyl 2-phenyl-2-vinylmalonate (**1**, 0.10 mmol), 1-bromododecane (**2**, 5 equiv, 0.50 mmol), vitamin B_12_ (10 mol %), Zn (3 equiv), NH_4_Cl (1.5 equiv), solvent (5 mL), white LEDs (6500 K), 16 h, 40 °C. Yields determined by GC analysis.

A preliminary screening of conditions for the model reaction of olefin **1** with 1-bromododecane (**2**) was performed using native vitamin B_12_ as a catalyst, Zn/NH_4_Cl as a reducing system, and white LEDs as an energy source (Figure 1). Control reactions in the common organic solvents MeOH, DMSO, or a water/acetonitrile mixture (1:1) provided desired product **3a**, albeit in low yields, 33%, 33%, and 18%, respectively. Several amphiphiles, cationic, anionic, and nonionic, were screened. The model reaction in an aqueous micellar solution proved to be surfactant dependent with dodecyl trimethylammonium chloride (DTAC), giving superior results (60%) and thus supporting the hypothesized micellar effect. Other surfactants were less efficient (6–40% yield). Moreover, it is known that different anion salts of the same amphiphile influence the micellization process and therefore should affect reactions in micellar solutions. Indeed, the halide anion exerts some influence on the reaction efficacy, and it follows the trend DTAC (60%) > DTAB (55%) > DTAI (48%). It corresponds well with the hydration level of micelles. DTAC micelles are the least hydrated, and therefore, assumingly, hydrophilic vitamin B_12_ can interact with the micellar interface more effectively.^35^ The cationic surfactant with the head ammonium salt not only enhanced the reaction yield but also eliminated the need for NH_4_Cl, a required additive in B_12_-catalyzed reactions. Gratifyingly, it also facilitates required zinc dispersion (see photo in the Supporting Information) and cleans the metal surface for electron transfer.^33^ Even in the presence of unactivated zinc, the reaction yielded product **3a** with only a slightly diminished yield (67%), in contrast to reactions in organic solvents. Furthermore, the so-called “co-solvent trick” here also played a role, as it alters the hydrogen-bonded structure.^36,37^ Among the cosolvents/additives used, *n*-BuOH exhibited the greatest effect. The alcohol is incorporated into the micellar interface, making micelles more flexible and improving the hydrophobic microenvironment capacity within the aqueous solution. Extensive optimization of reaction conditions with respect to catalyst, surfactant and cosurfactant, light, time, concentrations of all reagents, and micelles type ultimately enabled desired product **3a** to be obtained in 80% yield (see SI).

The desired reaction also occurs in pure water (see SI), possibly taking advantage of the “on water” mode of interactions. But in the presence of DTAC and *n*-BuOH as an additive, not only does the yield increase significantly but also the rate of the reaction, corroborating the beneficial effect of the micellar environment. The exact role of this environment has to be, however, determined. Therefore, we next focused our efforts on elucidating the origin of the micellar impact on the reaction studied.

### Model System

**Figure 2:**
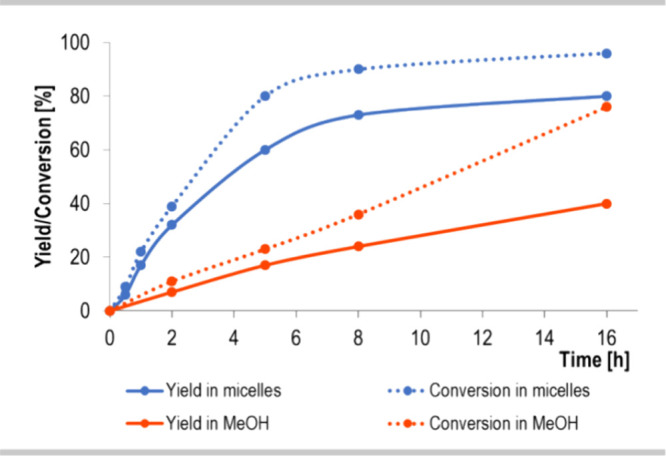
Kinetic profile of the model reactions. Dotted lines, conversion of olefin 1; solid lines, reaction yield.

The qualitatively different behavior of the yield/conversion vs time for the homogeneous organic solvent and the micellar solution agrees with recent theoretical predictions of micellar catalysis kinetics (Figure 2).^38^ It was found that the reaction rates in micellar systems can be higher than those in organic solvents, due to the change in the reaction entropy resulting from compartmentalization of reactants in microheterogeneous aqueous solutions. Thus, to better understand the molecular interactions within the entire noncovalent catalytic system, a series of in-depth studies were performed.

### **DTAC**

Computational chemistry predictions of the critical micellar concentrations (CMC)^39^ under the given reaction conditions are 18–25 mM DTAC, in agreement with the reported experimental data.^35,40^ DLS measurements of surfactant solutions in water show aggregation signals, and as the concentration of DTAC increases, the size of the aggregates increases from 0.72 to 1.27 nm. This trend is also observed in two-dimensional diffusion-ordered spectroscopy (2D DOESY NMR). The spectra were measured for DTAC solutions in D_2_O at various concentrations, including the one that corresponds to its concentration in the reaction studied. In all samples above the CMC, signals corresponding to aggregates were observed. Specific diffusion constants (*D*) of surfactant molecules decrease as their concentration increases, indicating the formation of larger aggregates (Table 1, column 3, entries 1–4). At the optimal reaction concentration (70 mM, far above the CMC), micelles of 1.20 nm hydrodynamic radius are formed (Table 2, entry 4). The addition of *n*-BuOH as an additive increases the size to 1.52 nm, which is in agreement with the literature data.^36^ At the same time, the phenomenon is expected to improve the permeability of the interface to organic compounds.^41^

**Table 1:** Specific Diffusion Constants and Hydrodynamic Radius Measured for DTAC and Olefin **1**^a^

		DTAC	**olefin in DTAC solution**	
**entry**	DTAC [μmol]	***D***_**DTAC**_ × 10^–10^ [m^2^ s^–1^]**(*****R***_**H**_ **[nm]**^b^**)**	***D***_**DTAC**_**×10**^**–10**^ [m^2^ s^–1^]**(*****R***_**H**_ **[nm]**^b^**)**	***D***_**olefin**_**×10**^**–10**^ [m^2^ s^–1^]^c^ **(*****R***_**H**_ **[nm]**^b^**)**
1	22	4.72 (0.55)	4.17 (0.61)	2.30 (0.99)
2	38	3.03 (0.78)	2.58 (0.90)	1.22 (1.73)
3	54	2.30 (0.99)	2.06 (1.09)	1.07 (1.96)
4	70	1.84 (1.20)	1.65 (1.32)	0.98 (2.21)
5	70/*n*BuOH^d^	1.41 (1.52)	1.20 (1.62)	0.98 (2.21)

a Samples were prepared in D_2_O (1 mL) and were shaken vigorously prior to measurements, measurement time 30 min.

b *R*_H_, hydrodynamic radius.

c Determined based on **O2** signals, olefin **1** (20 μmol) in DTAC at different concentrations in D_2_O (1 mL).

d *n-*BuOH (250 μmol).

**Table 2:**
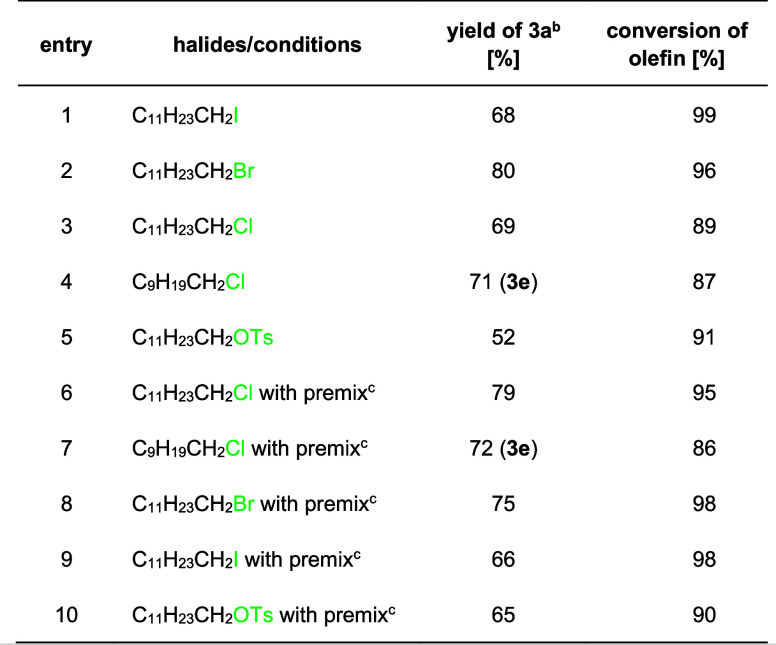
Premix Influence on the Model Reaction^a^

a Optimized reaction conditions: diethyl 2-phenyl-2-vinylmalonate (**1**, 0.10 mmol), bromide (3 equiv, 0.30 mmol), vitamin B_12_ (2.5 mol %), Zn (3 equiv), DTAC (0.35 mmol), *n*-BuOH (1.25 mmol), H_2_O (5 mL), green LEDs (525 nm), 16 h, 40 °C.

b Yields determined by GC analysis with mesitylene as an internal standard.

c Premix: zinc powder was stirred for 2 h in DTAC (0.35 mmol) solution in water prior to adding reagents and the catalyst.

In the ^1^H NMR spectra, the signals corresponding to the surfactant are slightly downfield shifted (from 0.72 to 0.76 ppm for CH_3_, 2.96 to 3.00 ppm for NCH_3_, 3.16 to 3.20 ppm for CH_2_) as the concentration increases, which, according to the literature, implies micelle formation.^42,43^

### Catalyst

**Figure 3:**
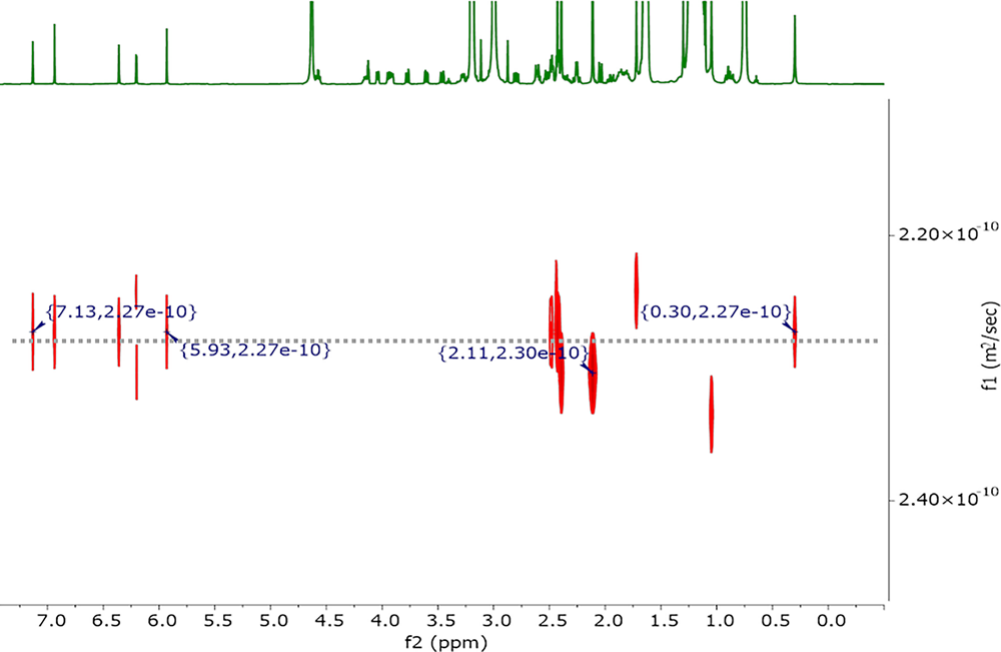
2D DOSY NMR spectra of vitamin B_12_ (0.6 μmol) in DTAC (70 μmol) solution in D_2_O (1 mL).

2D DOSY NMR data collected for the vitamin B_12_ (0.6 μmol) in DTAC (70 μmol) solution in D_2_O (1 mL) show peaks corresponding to only one catalyst entity for which the specific diffusion coefficient is equal to 2.27 × 10^–10^ m^2^ s^–1^, corresponding to a weight of 1440 g/mol (Figure 3). This corroborates that the hydrophilic vitamin B_12_ (1355 g/mol) remains in an aqueous phase as a monomer surrounded by water molecules and does not participate in the formation of aggregates. This might suggest that the transformation can be classified as type IIa, which means that the reaction takes place on the surface of self-assembled aggregates that accommodate lipophilic reagents with the catalyst being only in the aqueous phase.^44^ But in fact, in vitamin B_12_-catalyzed reactions the Co(I) form is catalytically active and *our theoretical calculations revealed that this species prefers to be located at the micelle–water interface* (see the Reactive Intermediates part).

### Olefin

**Figure 4:**
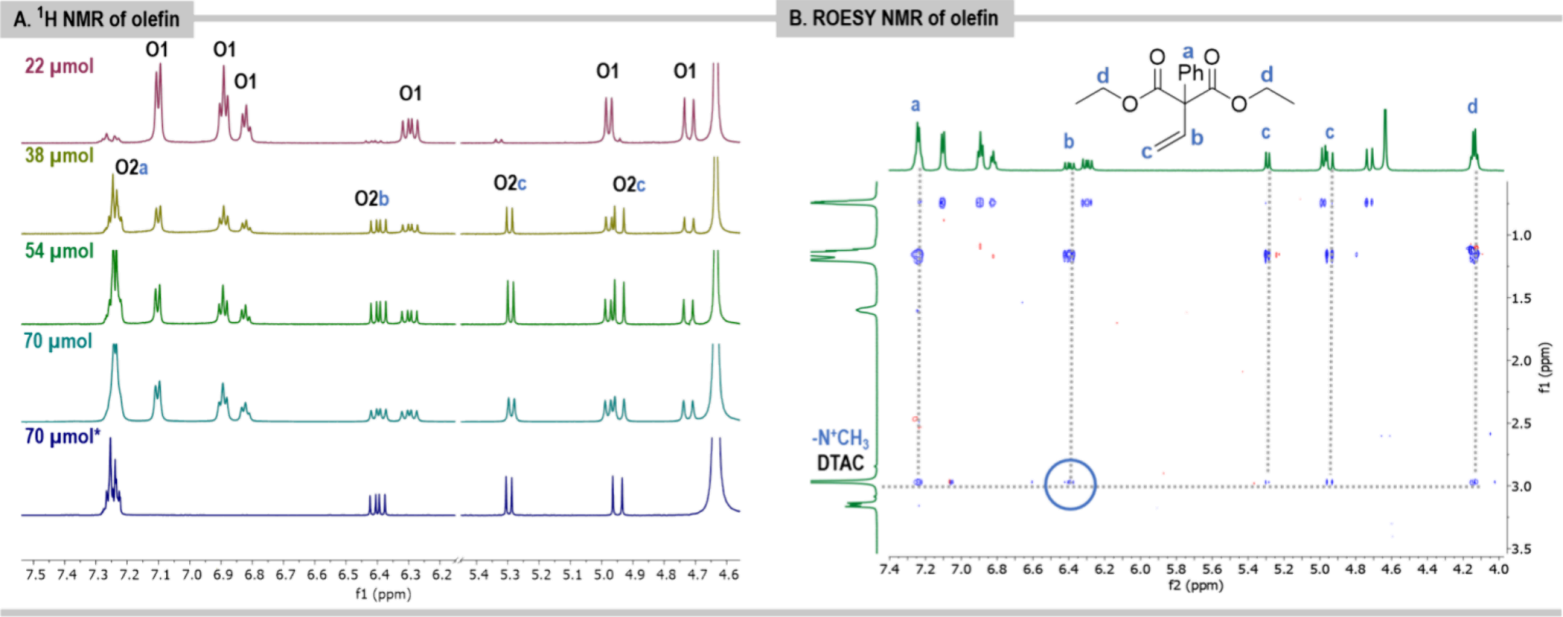
(A) ^1^H NMR spectra of olefin **1** (20 μmol) in DTAC at different concentrations in D_2_O (1 mL). *Olefin **1** (5 μmol) in DTAC (70 μmol) solution in D_2_O (1 mL). (B) ROESY NMR spectra of olefin **1** (20 μmol) in DTAC (54 μmol) solution in D_2_O (1 mL). ^1^H NMR spectra were measured for 1.5 min for samples that were vigorously shaken (as is during the reaction).

The addition of diethyl 2-phenyl-2-vinyl malonate (**1**) to a DTAC solution causes a decrease in the specific diffusion coefficient of surfactant molecules (Table 1, column 4). The increased size of the aggregates suggests the localization of hydrophobic olefin in the micelle, as proved by the Blum and Peacock FILM studies.^45^ As the concentration of the surfactant increases, the size of the aggregates with olefin also increases. The ^1^H NMR spectra of olefin (20 μmol) measured in DTAC solutions in D_2_O (1 mL) at various concentrations showed two distinct sets of sharp signals, **O1** and **O2**, corresponding to protons of the two olefin entities that correlate with DOSY results. These may suggest that only part of the olefin molecules are incorporated into the micelle and that the exchange between molecules occurs at a relatively slow rate on the NMR time scale (Figure 4A). Specific diffusion constants determined on **O2** signals decrease as the concentration of DTAC increases (Table 1, column 4, entries 1–4), while *D*s determined on **O1** signals are very similar in all measurements and are in the range (0.42–0.56) × 10^–10^ m^2^ s^–1^. In the ^1^H NMR spectra for the solution of 22 μmol of DTAC **O1** signals are of higher integrated intensity, suggesting that the equilibrium is shifted toward **O1** aggregates; in this case, the reaction efficiency is lower (63% vs 80%). At higher concentrations of DTAC, **O2** signals are more intense. The control ^1^H NMR spectra of a very diluted solution of olefin (5 μmol) solution show only **O2** signals. This suggests that **O2** signals may originate from olefinic protons that interact with micelles and that olefin aggregates (corresponding to **O1** signals) are not formed in this case. The possible interaction should be recognized from the occurrence of cross-peaks in the rotating-frame nuclear Overhauser-effect correlation spectra. Indeed, the ROESY experiment clearly shows the correlation of **O2** olefin protons with the surfactant, NCH_3_ (Figure 4B, the signal in the blue circle). Therefore, only protons corresponding to the **O2** form interact with the surfactant, confirming its location at the hydrophilic–hydrophobic interface.

*The ROESY NMR experiments indicate favorable preassociation of the olefin molecules at the micellar interface.*

### Halides

**Figure 5:**
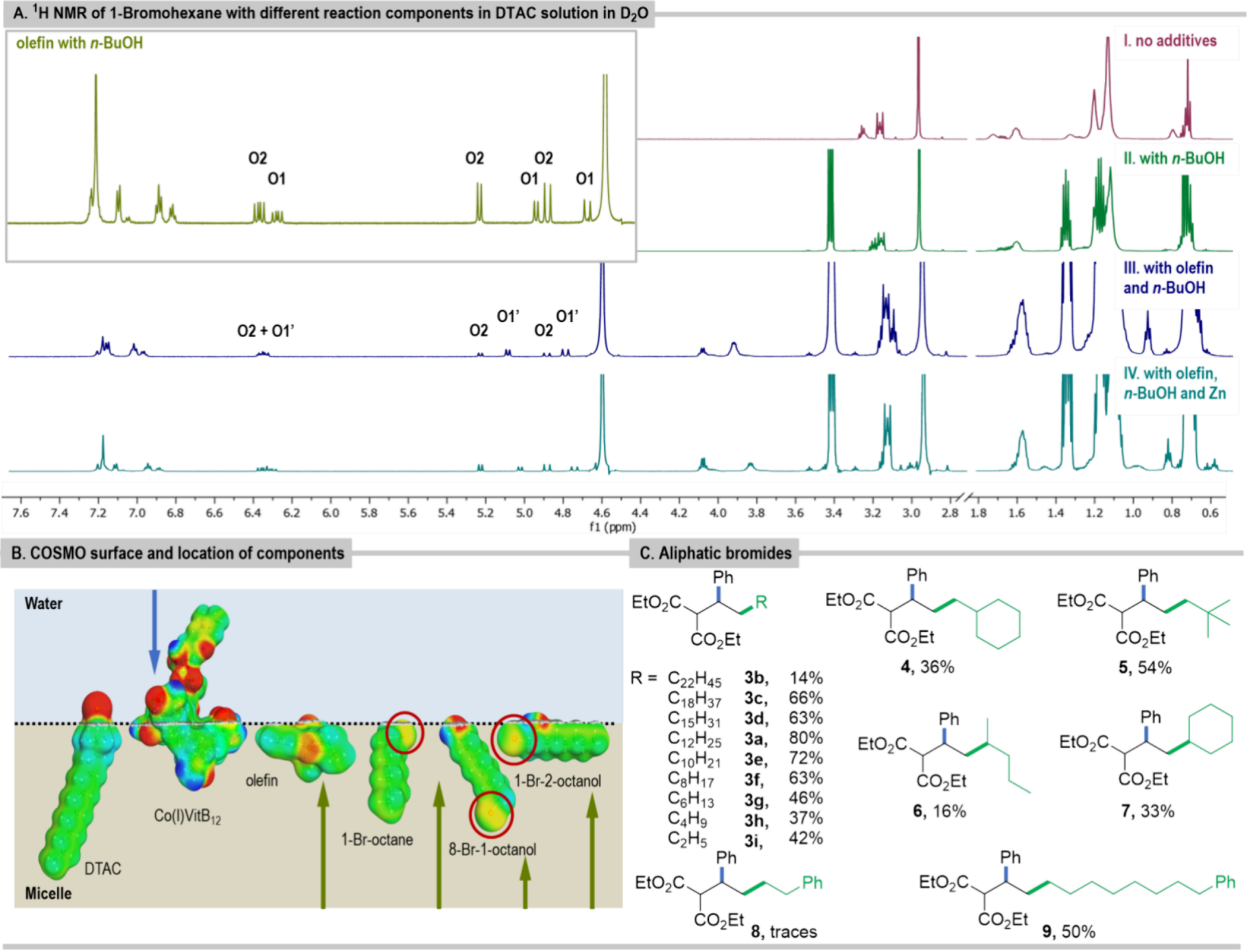
(A) ^1^H NMR spectra of 1-bromohexane (60 μmol) in (I) DTAC (70 μmol) solution in D_2_O (1 mL); (II) DTAC (70 μmol) solution in D_2_O (1 mL) with *n-*BuOH (250 μmol); (III) with olefin (20 μmol) and *n-*BuOH (250 μmol) in DTAC (70 μmol) solution in D_2_O (1 mL); (IV) DTAC (70 μmol) solution in D_2_O (1 mL) with olefin (20 μmol), *n-*BuOH (250 μmol), and Zn (60 μmol). (B) COSMO surface and the most stable location of the components of the reaction mixture in the micellar solution, including the surfactant DTAC. (C) Reaction products of olefin **1** with aliphatic bromides.

In the ^1^H NMR spectra measured for alkyl bromides in DTAC (70 μmol) solution in D_2_O (1 mL), not only are signals broadened, but additional sets of signals are also observed; the longer the aliphatic chain, the broader the signals (see SI). The results of the 2D DOSY NMR measurements for 1-bromohexane indicate that the specific diffusion coefficient of the surfactant increases to 2.10 × 10^–10^ m^2^ s^–1^, which corroborates the interaction of bromides with micelles, and in addition larger aggregates of the bromide are also present in the solution. When *n*-BuOH is added to a sample containing hexyl bromide in the DTAC solution (D_2_O), the signals become sharper as a consequence of changes in the partitioning between phases^41^ and the slower exchange rate between entities that are present at sufficient concentrations to be detected by NMR measurements (Figure 5A).

Functional groups influence substrate organization in the micellar environment and hence change the reaction rate.^46^ The strongest influence could be expected for compounds comprising the hydroxyl group in their structure due to its high affinity for forming hydrogen bonds; thus 1-bromooctan-2-ol and 8-bromooctan-1-ol were selected as extreme model cases. Due to their polar structure, these bromides can act as a cosurfactant and incorporate into the micellar structure. DLS measurements indicate that DTAC/1-bromooctan-2-ol aggregates are bigger than those with 8-bromooctan-1-ol. This may be explained by their better fit to the structure of the surfactant layer. The specific diffusion constant for 1-bromooctan-2-ol is equal to 1.10 × 10^–10^ m^2^ s^–1^, while for 8-bromooctan-1-ol it is 1.17 × 10^–10^ m^2^ s^–1^, reflecting this trend. For both bromides, in ^1^H NMR spectra the signals are broadened, and again the addition of *n*-BuOH sharpens the signals (see SI). Now, there are additional distinctive sets of signals corresponding to the bromides’ entities that are present in two different environments and form different aggregates.

Theoretical COSMO-RS studies indeed show that when the bromide substituent is in close proximity to the hydroxyl group, this part of the molecule is located in the hydrophilic section of the micelle (Figure 5B). On the other hand, in 8-bromooctan-1-ol, the groups are separated by the hydrophobic chain, and it is the hydroxyl group that stays predominantly at the micelle–water interface.

In general, in vitamin B_12_-catalyzed reactions, alkyl chlorides and tosylates are less reactive compared to their bromide counterparts. Here, in both cases, the reactions were, however, only slightly less efficient (Table 2, entries 1–5). We also investigated the effect of preencapsulation of zinc (premix), which according to Peacock and Blum reduces protodemetalation pathways in cross-coupling reactions in micellar solutions.^33^ In our case, this would lead to dehalogenation of alkyl halides that may also be catalyzed by vitamin B_12_. We have not, however, seen any significant differences; thus, this path is not valid here and the observed dehalogenation originates from the catalytic process (entries 6–10).

### Vitamin B_12_-Catalyzed Tandem Radical Addition/1,2-Aryl Migration

The reaction of olefin **1** with 1-bromododecane (**2**) in the presence of native vitamin B_12_ and Zn as a reductant under green light irradiation (525 nm) gave the desired product in 80% yield. Our NMR and theoretical studies on the localization of the reagents indicate that the reaction occurs in the interface region (Stern layer). Indeed, the predictions from the COSMO-RS calculations for the mole fractions of all components in the micellar core and in the micellar interface region revealed that for a chain length of 12, the micellar interface mole fractions for bromide **2** and olefin **1** are identical and equal to 0.022, while the micellar core mole fractions are dominated by bromide and olefin (for details see SI). Since the active form of the Co-catalyst is only present in the Stern layer (see Reactive Intermediates section), bromide points toward this region, and part of the olefin molecules are there, the reaction occurs in the interface region.

Because NMR techniques demonstrated utility in probing the micellar structure,^47^ we focused on studying interactions between reagents and micelles within the whole reacting mixture. The ^1^H NMR spectra of substrates, 1-bromohexane (60 μmol) and olefin **1** (20 μmol), in DTAC (70 μmol) solution in D_2_O (1 mL) with the addition of *n*-BuOH (250 μmol) show two sets of resonances for olefinic protons (Figure 5A). Chemical shifts for one set are very similar to chemical shifts of **O2** resonances, 0.02 ppm upfield shifted, that correspond to the olefin/micelle aggregates. The second set of resonances (**O1′**) is downfield shifted. Based on ROESY measurements (Figure 4B) and COSMO-RS data, it can be assumed that only the **O2** form reacts with radicals, and since the yield of the reaction is 46% yield, it must exist in the equilibrium with **O1′**.

**Figure 6:**
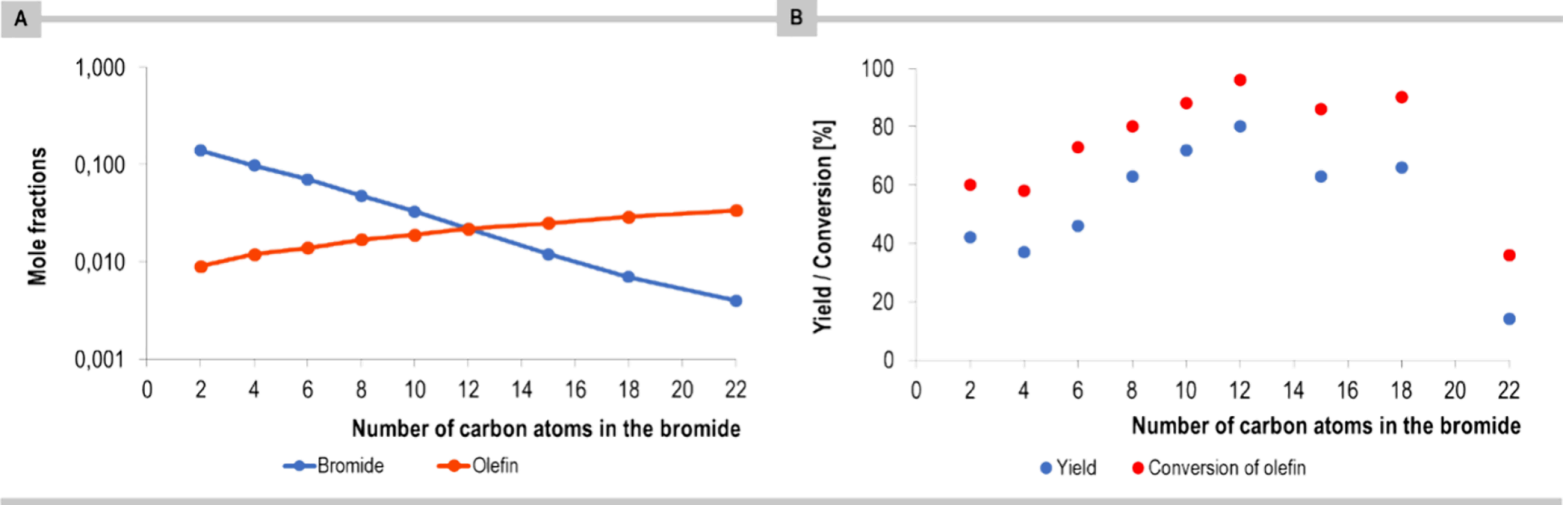
(A) Mole fractions in the interfacial region of alkyl bromide and olefin **1**. (B) The impact of the length of the aliphatic chain on the reaction outcome.

**Figure 7:**
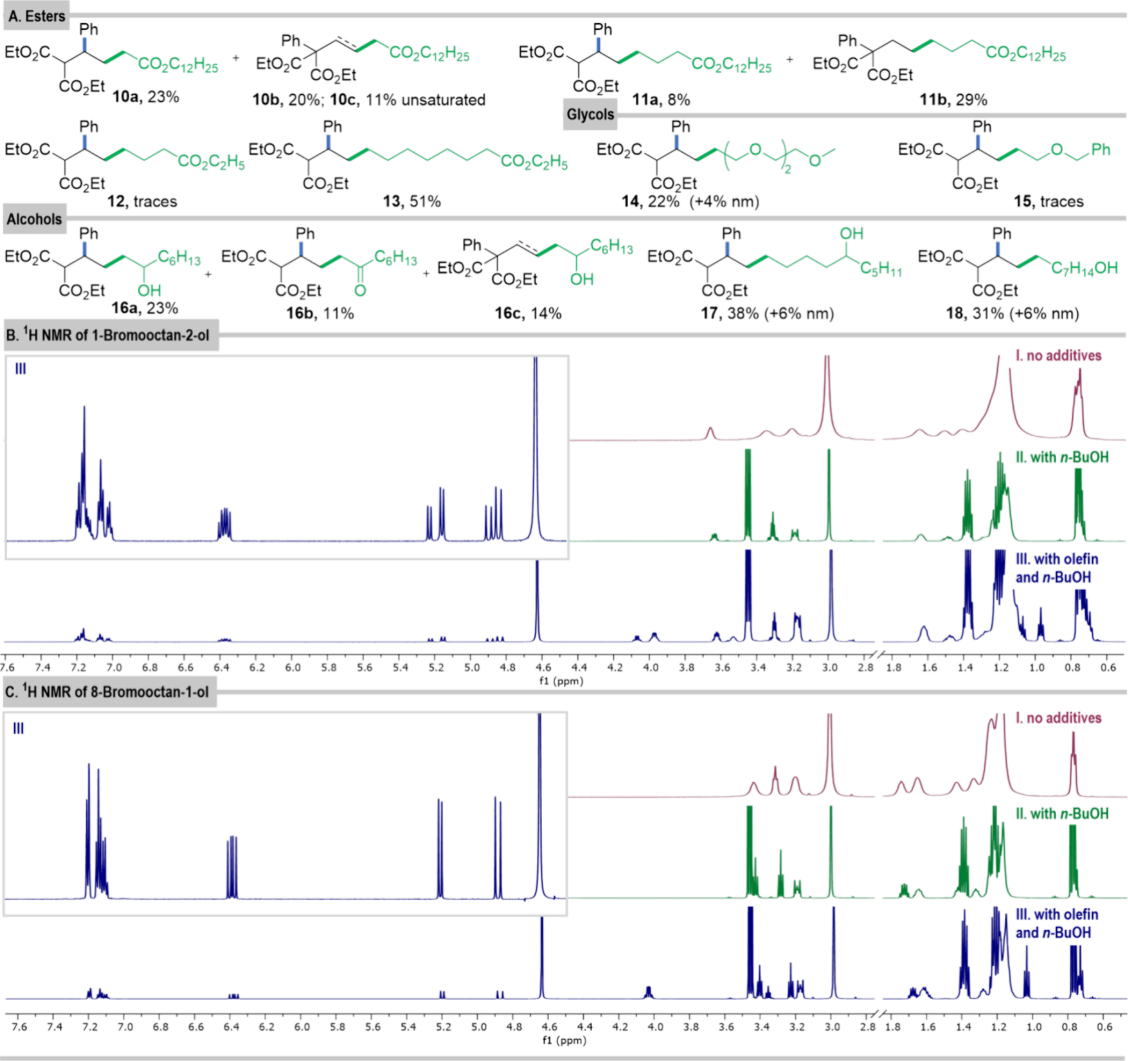
(A) Products formed from olefin and bromides with ester, glycol, and alcohol groups. (B) ^1^H NMR spectra of 1-bromooctan-2-ol (60 μmol) in (I) DTAC (70 μmol) solution in D_2_O (1 mL); (II) DTAC (70 μmol) solution in D_2_O (1 mL) with *n-*BuOH (250 μmol); (III) DTAC (70 μmol) solution in D_2_O (1 mL) with olefin (20 μmol) and *n*-BuOH (250 μmol). (C) ^1^H NMR spectra of 8-bromooctan-1-ol (60 μmol) in (I) DTAC (70 μmol) solution in D_2_O (1 mL); (II) DTAC (70 μmol) solution in D_2_O (1 mL) with *n*-BuOH (250 μmol); (III) in DTAC (70 μmol) solution in D_2_O (1 mL) with olefin (20 μmol) and *n-*BuOH (250 μmol); nm, product with the aryl group not migrated.

Furthermore, the reaction efficiency is strongly dependent on the length of the reacting alkyl bromide. The calculated mole fractions of the olefin and the alkyl bromide in the micellar interface region are shown in Figure 6A. The minimum mole fraction of the two reactants has a maximum for a chain length of 12, which is in agreement with the yield and conversion observed experimentally (Figure 6B). This is consistent with the formation of a very short-lived and reactive radical species, which needs a 1:1 partner of olefin for optimum efficiency. For shorter chain radicals, there is a surplus of alkyl bromide, and the proposed reaction mechanism would result in a side reaction of alkylation of the radical. The longer-chain radicals would have a reaction partner, so fewer side reactions are expected, and only slower reactions because of the lower concentration of the alkyl bromide. Experimentally, the best yield, 80%, was obtained for the model 1-bromododecane, whose length corresponds well to the diameter of the micelle core (an alkyl chain length corresponds to that present in the surfactant). As the diameter decreases or increases (using surfactants that possess shorter alkyl chains (C8) or longer (C18) than DTAC (C12)), the yield diminishes to 7% and 50%, respectively. Both longer and shorter alkyl bromides give inferior results, which can be explained by the less advantageous alignment of the substrate inside the micelles.^41,46^ Long-chain halides must fold to fit into the structure of the surfactant layer, enhancing the steric hindrance around the bromide-substituted carbon atom and impairing the interaction with the catalyst molecule. Shorter-chain substrates have a lot of space to freely move within the confinement, which minimizes the micellar effect. More sterically bulky, cyclohexyl methyl bromide and neopentyl bromide provide the desired products though in yields of 36% and 54%, respectively. Expectedly, secondary bromides proved less efficient, as it is well documented that the formation of the respective alkyl cobalamins is unfavorable and that secondary halides are by far more reactive toward undesired insertion of zinc, leading to organozinc halides and subsequent protodemetalation.^48^ As a consequence, products **6** and **7** form in lower yields, respectively, 16% and 33%. Reactions with aliphatic bromides containing a phenyl ring (bromo ethylbenzene) proved unsuccessful (**8**). Only separation between the bromide substituent and the phenyl ring that exceeds eight bonds allowed the synthesis of desired products (**9**, 50%), seemingly due to the possibility of the bromide folding inside the micelles and thus reaching the preferred orientation. The importance of a proper fitting to the surfactant layer is also reflected in the reaction efficacy of olefin **1** with bromides having a terminal ester group (Figure 7A, **10**–**13**). Again, the longer the aliphatic chain, the higher the yield of the reaction. We were also interested in the reactivity of hydrophilic PEGylated bromides, since in this case the polyoxyethylene chain should point toward the water phase, thus altering the localization of an alkyl bromide. Indeed, it afforded product **14**, albeit in a low yield, thus further corroborating that effective collision of the substrates takes place in the interface layer.

To form alkylcobalamin, the reduced catalyst has to intercept an alkyl bromide; thus we assume that the bromide atom should point toward the surface of the micelle where the catalyst is present. Consequently, the presence of any functional groups influencing the organization of substrates in the micelles should impact the reaction rate.

The interaction occurring between substrates was investigated at the atomic level based on NMR measurements of mixtures of 1-bromoctan-2-ol and 8-bromoctan-1-ol (60 μmol) with olefin **1** (20 μmol) in DTAC (70 μmol) solution in D_2_O (1 mL) with *n-*BuOH (250 μmol). In both cases, the size of the aggregates becomes larger, regardless of the location of the hydroxy group in the bromide (0.98 × 10^–10^ m^2^ s^–1^ and 0.88 × 10^–10^ m^2^ s^–1^ for 1,2- and 1,8-regioisomers, respectively); hence the reactants fit in the surfactant layers. COSMO-RS calculations showed that for 8-bromoctan-1-ol, the bromide substituent is deeply buried in the aggregate, making it difficult to react with the catalyst. Furthermore, ^1^H NMR spectra of the olefin with the two bromo-alcohols show substantial differences in the olefinic proton region. For 1-bromoctan-2-ol, as in the model case, two sets of signals (**O2** and **O3**) corresponding to olefinic protons are observed, and they are slightly shifted (**O2** upfield, **O3** downfield). On the contrary, for 8-bromoctan-1-ol, only one set is present. These differences are reflected in the reactivity of these substrates toward olefin in the micellar system. The reaction of diethyl 2-phenyl-2-vinylmalonate (**1**) with 8-bromoctan-1-ol yields the mixture of products in 37% yield (**18**). The yield increases to 44% for 1-bromodecan-5-ol (**17**) and up to 48% for 1-bromooctan-2-ol (**16a**–**c**); we compare total yields, as they reflect efficiency of the radical formation from bromo-alcohols.

**Figure 8:**
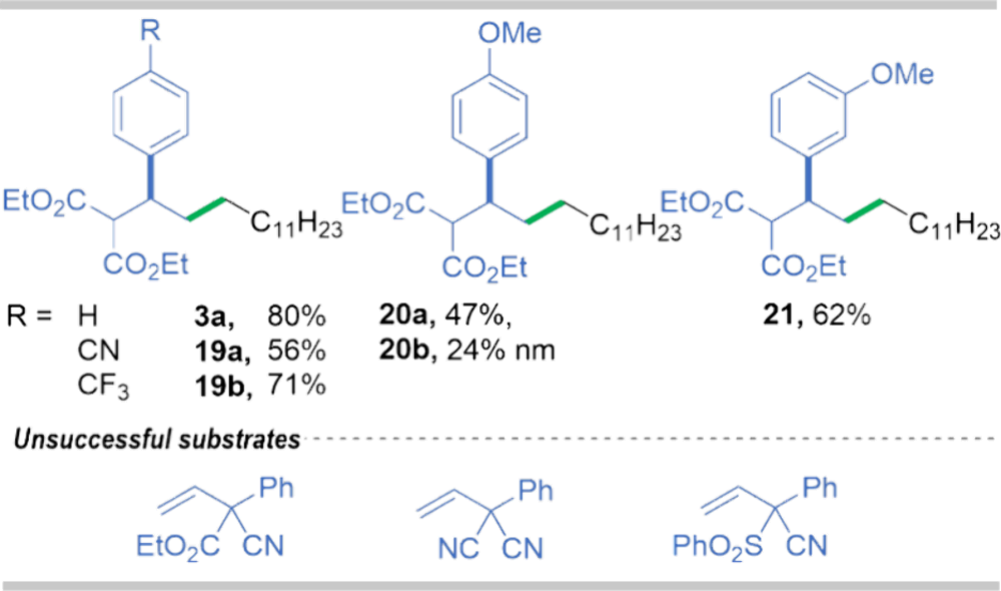
Products of reactions 1-bromododecane with various olefins.

In terms of olefins, the presence of functional groups and their position in the aromatic ring of the olefin affect the reaction course. The introduction of both electron withdrawing group (EWG) (−CN, −CF_3_) and electron donating groups (EDG) (−OMe)results in a slight decrease in reaction yields, suggesting that the interreactant orientation in the micellar solution was not significantly altered (**19**–**21**, Figure 8). In contrast, olefins with other functional groups of different polarity (−CN, −SO_2_Ph) that may have a strong impact on the localization of a substrate furnished a complex mixture of products. The challenging synthesis of ketoesters and diketones precluded their use as substrates in the developed transformation. The results above confirm that in the presence of DTAC and *n*-BuOH as an additive, the yield and reaction rate increase significantly, confirming the beneficial effect of the micellar environment. *The experimental and theoretical data clearly indicate the influence of the bromide structure on the interposition of the reactants*.

These amplifications can be explained by the favorable distribution of the reactants and the restriction of their free movement inside the micellar solution and thus the increase in the likelihood of an effective collision.

### Reactive Intermediates

Control experiments revealed that vitamin B_12_, zinc, and light are essential to obtain the desired product (Table 3, entries 2–5). Reactions without either the surfactant or cosolvent are less efficient. From the point of view of the reaction mechanism, we assume that the use of micellar solutions should not affect the formation of main reactive intermediates but should have an impact on the selectivity and the reaction rate.

**Table 3:**
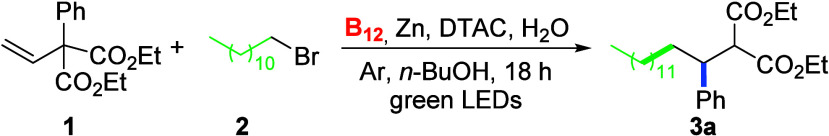
Control Experiments for the Vitamin B_12_-Catalyzed Addition/1,2-Phenyl Migration^a^

**entry**	**deviation from the reaction conditions**	**yield of3a[%]**^b^
1	-	80
2	no B_12_	0
3	no Zn	0
4	no light	0
5	no B_12_, Zn and light	0
6	under air	7
7	no surfactant	31
8	no butan-1-ol	65

a Optimized reaction conditions: diethyl 2-phenyl-2-vinylmalonate (**1**, 0.10 mmol), 1-bromododecane (3 equiv, 0.30 mmol), vitamin B_12_ (2.5 mol %), Zn (3 equiv), DTAC (0.35 mmol), *n*-BuOH (1.25 mmol), H_2_O (5 mL), green LEDs (525 nm), 16 h, 40 °C.

b Yields determined by GC analysis with mesitylene as an internal standard.

**Scheme 2:**
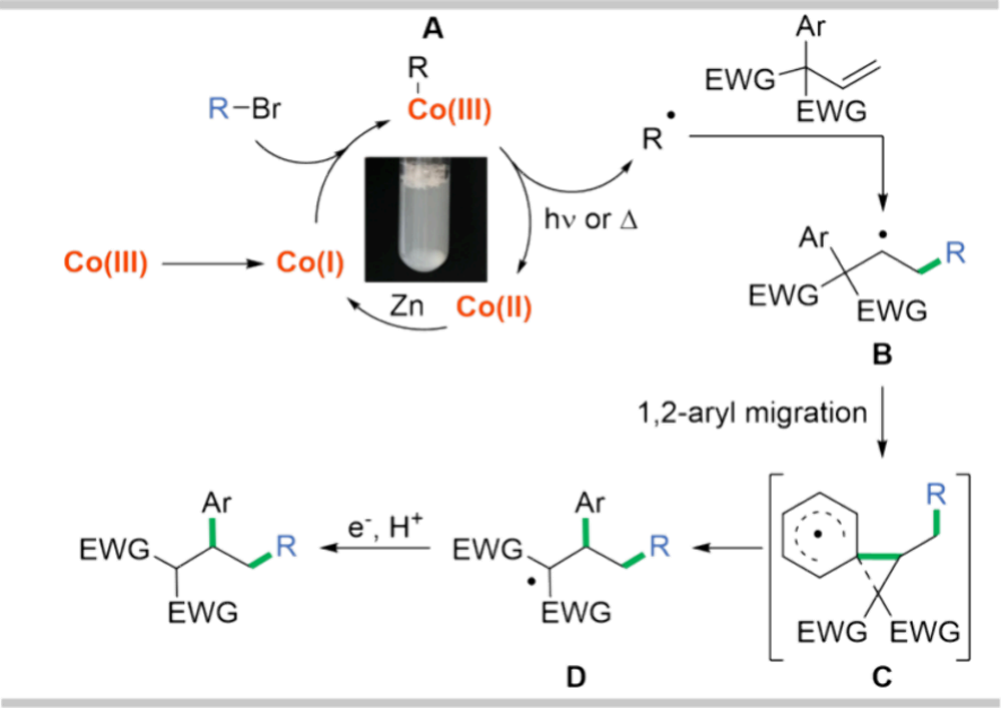
Plausible Reaction Mechanism

Based on our knowledge and previous reports,^49,50^ we formulated the hypothetical mechanism for the vitamin B_12_-catalyzed tandem addition/1,2-phenyl migration of alkyl bromides with olefins (Scheme 2). In the first step, zinc reduces vitamin B_12_ to its active Co(I) form. This “supernucleophile” undergoes a reaction with bromide that furnishes alkylcobalamin **A**. The resulting intermediate, upon light irradiation or heating, generates a radical, which reacts with an electron-deficient olefin, providing alkyl radical **B**. After 1,2-aryl migration via transition state **C**, radical **D** forms and after protonation delivers the desired product. A set of mechanistic experiments corroborated the formation of reactive intermediates in the proposed mechanistic pathway.

#### Co(I) Form

The effective reduction of vitamin B_12_ to its active Co(I) form by zinc is usually ensured by the use of activated zinc powder, the addition of NH_4_Cl, and virulent stirring.^19^ Herein, even though the micelles are positioned on the zinc surface as found by Blum using imaging techniques,^33^ the effective reduction of the Co^3+^ ion to Co^1+^ occurs as a usual color change of the reaction mixture was observed from red to deep green/brown.

**Figure 9:**
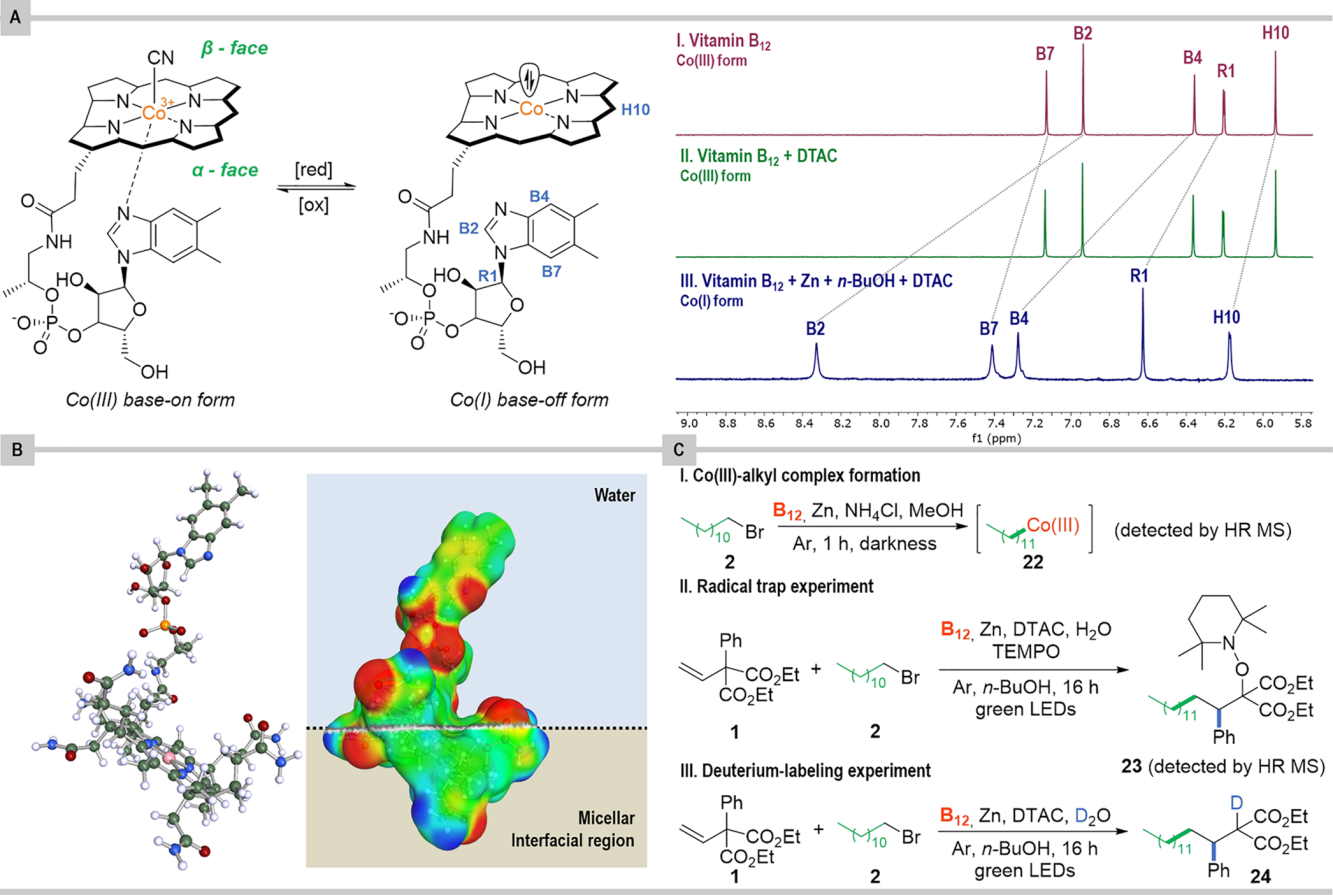
Mechanistic studies. (A) ^1^H NMR spectra of (I) vitamin B_12_ (0.6 μmol) in D_2_O (1 mL); (II) vitamin B_12_ (0.6 μmol) in DTAC (70 μmol) solution in D_2_O (1 mL); (III) vitamin B_12_ (2.4 μmol) in DTAC (70 μmol) solution in D_2_O (1 mL) with *n-*BuOH (250 μmol) and Zn (240 μmol). (B) Molecular structure of Co(I) vitamin B_12_ (left) and the COSMO surface and the most stable location at the micellar interface. (C) Mechanistic experiments.

The calculated free energy of transfer of a Zn nanoparticle model from the micellar core to the micellar interface region is only +4 kJ/mol.^51^ This indicates that Zn prefers the micellar core, but will have a nonnegligible probability of being at the interface, where it can reduce Co(III) into the active Co(I) form, which is inherently in a *base-off* form.^50^^1^H NMR studies of the cobalamin solution show resonances corresponding to protons in the nucleotide loop in the range between 6 and 7 ppm. The addition of zinc powder causes a shift to 6–9 ppm (Figure 9A). This downfield shift is characteristic of the *base-off* form of cobalamin.^52^ For computational reasons, we used a nanoparticle model for Zn, but we assume that the surface interactions from this model can be generalized to imply which part of the surfactant will interact with a larger zinc surface. This parallel is analogous to our previous computational work for explaining why water-sensitive Negeshi couplings using zinc powder work in aqueous micellar systems.^51^

Furthermore, a set of calculations for the free energy of transfer from the aqueous phase to the micellar interface for Co(I) species **A** showed a favorable interaction, −14 kJ/mol. The most stable interaction geometry off the *base-off* form is shown in Figure 9B and indicates that the Co(I) ion is in the micellar interface region and can therefore react with the alkyl bromide. Thus, it further supports the postulated alignment of the reactants in a micellar system (Figure 5B).

*Calculations and NMR data confirm that the Co(I) form is generated in a micellar system, even though Zn prefers the micellar core, thus allowing the reaction to proceed*.

#### Alkyl Cobalamin

Once the Co(I) species is generated, it reacts with alkyl bromides to afford alkyl cobalamin **A**. HR-MS of the crude reaction mixture in MeOH shows the peak at [M + H], *m*/*z* 1498.7650, that corresponds to the Co(III)-alkyl complex **22**. Fortunately, ^1^H NMR spectra measured for the reaction mixture without olefin show signals at −0.24 and −0.80 ppm, which are characteristic for alkyl cobalamin, corroborating its formation during the catalytic cycle.^53^

As an alternative, the reaction mechanism involving alkylzinc bromide may be considered. It has been assumed that, in micellar systems based on DL-alpha-tocopherol methoxypolyethylene glycol succinate (TPGS), palladium-catalyzed cross-coupling reactions involve the formation of alkylzinc(II) halides.^33^ It is not, however, the case under the developed conditions. The ^1^H NMR spectrum for the mixture of octyl bromide, zinc, and DTAC in deuterated water shows only signals corresponding to hydrogen atoms present in alkyl bromide and DTAC (see SI). Characteristic signals for alkylzinc bromide are not observed.^54^ Therefore, *the only role of zinc in the transformation developed is as a reducing agent*.

#### Radicals

The mechanism is radical in nature, as the reaction was completely halted once the radical trap was added prior to exposure to light (Figure 9C). Analysis of the reaction mixture by ESI-MS showed the presence of a peak corresponding to the TEMPO adduct **23**, which was formed from a radical **D** generated by adding 1-bromododecane (**2**) to olefin **1**.

#### Anion

The reaction in D_2_O, which is a source of deuterium cation, provides the desired product **24** with the deuterium atom incorporated at the α-position to the carbonyl group (see SI), thus corroborating the formation of an anion at this position that after protonation furnishes the desired product. This result is consistent with 1,2-aryl migration and protonation, as shown in Scheme 2.

All reactive intermediates involved in the catalytic cycle are confirmed, but with the experimental evidence collected we cannot exclude the activity of chain reaction processes and/or alternative mechanistic pathways.

## Conclusions

The micellar system proved to be suitable for the Co-catalyzed radical addition/1,2-aryl migration, and the micellar environment is pivotal to obtain the desired products in high yields. NMR studies of the model reaction indicate the localization of reactants in the micellar system and enabled the determination of reactive intermediates in the reaction pathway. Our mechanistic analysis and theoretical studies, along with understanding the interactions within the entire noncovalent catalytic system, reveal that the aliphatic chain length and the presence of functional groups have a strong impact on the organization of substrates in the micellar solution.

This work expands the chemical space related to both Co(porphyrinoid) catalysis and an aqueous micellar environment, opening access to a new research area at the intersection of these fields. We believe that these findings will serve as an inspiration for broadening the utility of micelle-mediated radical transformations for the advancement of green chemistry applications.

## Supporting Information Available

Experimental details and procedures, optimization studies, mechanistic experiments, and spectral data for all new compounds (PDF)
