# Supplementary material for: Aqueous Micellar Environment Impacts the Co-Catalyzed Phototransformation: A Case Study
Source: J Am Chem Soc. 2024 Jul 9;146(29):19828–38. doi: 10.1021/jacs.4c02682 (PMC11273611; doi:10.1021/jacs.4c02682)

Supporting Information

Aqueous Micellar Environment Impacts the Co-Catalyzed Phototransformation: A Case Study

Aleksandra Wincenciuk,[a] Piotr Cmoch,[a]
Maciej Giedyk,[a]* Martin Andersson,[b]* and Dorota Gryko[a]*

*[a]Institute of Organic Chemistry Polish Academy of Sciences;
Kasprzaka 44/52, 01-224 Warsaw, Poland*

*[b]Center for Integrative Petroleum Research, King Fahd University of Petroleum and Minerals,
Dhahran 31261, Kingdom of Saudi Arabia*

*correspondence:
dorota.gryko@icho.edu.pl,
martin.andersson@kfupm.edu.sa,
maciej.giedyk@icho.edu.pl.*

Table of Contents

1. General Informations S5

2. Setup for photoreactions S6

3. Optimization studies S7

3.1. Background experiments S7

3.2. Type of cobalt catalyst S7

3.3. Screening of surfactant S8

3.4. Additives S9

3.5. Amount of DTAC S9

3.6. Amount of *n*-BuOH S10

3.7. Amount of water S10

3.8. Substrates ratio S10

3.9. The influence of light S11

3.10. Amount of vitamin B_{12} and Zn S11

3.11. Screening of reductant S12

3.12. Influence of buffer S12

3.13. Reaction time S12

4. Preparation of starting materials (S1-S11) and characterization of new compounds S13

*diethyl 2-(3-methoxyphenyl)-2-vinylmalonate, **S3*** S13

*diethyl 2-(4-(trifluoromethyl)phenyl)-2-vinylmalonate, **S5*** S14

*dodecyl 4-bromobutanoate, **S11*** S14

5. General Procedures for Tandem Radical Addition/1,2-Aryl Migration Reaction S15

5.1. General procedure for liquid substrats S15

5.2. General procedure for solid substrates S16

5.3. Notes S16

6. Products and characterization of new compounds S17

*diethyl 2-(1-phenyltetracosyl)malonate, **3b*** S17

*diethyl 2-(1-phenylicosyl)malonate, **3c*** S17

*diethyl 2-(1-phenylheptadecyl)malonate, **3d*** S18

*diethyl 2-(1-phenyltetradecyl)malonate, **3a*** S18

*diethyl 2-(1-phenyldodecyl)malonate, **3e*** S19

*diethyl 2-(1-phenyldecyl)malonate, **3f*** S19

*diethyl 2-(1-phenyloctyl)malonate, **3g*** S20

*diethyl 2-(1-phenylhexyl)malonate, **3h*** S21

*diethyl 2-(1-phenylbutyl)malonate, **3i*** S21

*diethyl 2-(3-cyclohexyl-1-phenylpropyl)malonate, **4*** S22

*diethyl 2-(4,4-dimethyl-1-phenylpentyl)malonate, **5*** S22

*diethyl 2-(3-methyl-1-phenylhexyl)malonate, **6*** S23

*diethyl 2-(2-cyclohexyl-1-phenylethyl)malonate, **7*** S23

*diethyl 2-(1,10-diphenyldecyl)malonate, **9*** S24

4-dodecyl 1,1-diethyl 2-phenylbutane-1,1,4-tricarboxylate, ***10a*** ... S24

4-dodecyl 1,1-diethyl 1-phenylbutane-1,1,4-tricarboxylate, ***10b*** ... S25

4-dodecyl 1,1-diethyl-1-phenylbut-2-ene-1,1,4-tricarboxylate, ***10c*** ... S25

6-dodecyl 1,1-diethyl 2-phenylhexane-1,1,6-tricarboxylate, ***11a*** ... S26

6-dodecyl 1,1-diethyl 1-phenylhexane-1,1,6-tricarboxylate, ***11b*** ... S26

triethyl 2-phenyldecane-1,1,10-tricarboxylate, ***13*** ... S27

a mixture of diethyl 2-(4-(2-(2-methoxyethoxy)ethoxy)-1-phenylbutyl)malonate, ***14*** *and diethyl 2-(4-(2-(2-methoxyethoxy)ethoxy)butyl)-2-phenylmalonate* ... S27

diethyl 2-(4-hydroxy-1-phenyldecyl)malonate, ***16a*** ... S28

diethyl 2-(4-oxo-1-phenyldecyl)malonate, ***16b*** ... S28

diethyl 2-(4-hydroxydecyl)-2-phenylmalonate, ***16c*** ... S29

diethyl 2-(7-hydroxy-1-phenyldodecyl)malonate, ***17*** ... S29

diethyl 2-(10-hydroxy-1-phenyldecyl)malonate, ***18*** ... S30

diethyl 2-(1-(4-cyanophenyl)tetradecyl)malonate, ***19a*** ... S30

diethyl 2-(1-(4-(trifluoromethyl)phenyl)tetradecyl)malonate, ***19b*** ... S31

diethyl 2-(1-(4-methoxyphenyl)tetradecyl)malonate, ***20a*** ... S32

diethyl 2-(4-methoxyphenyl)-2-tetradecylmalonate, ***20b*** ... S32

diethyl 2-(1-(3-methoxyphenyl)tetradecyl)malonate, ***21*** ... S33

7. Mechanistic consideration ... S34

7.1. Proposed mechanism ... S34

7.2. Co(III)-alkyl complex formation ... S34

7.3. Experiment with a radical trap ... S36

7.4. Deuterium labeling experiment ... S37

7.5. Undesired products ... S38

7.6. NMR spectroscopy measurements and calculation of the sizes. ... S40

7.6.1. ^{1}H NMR data for reaction components ... S41

DTAC at different concentrations ... S41

DTAC systems. ... S42

Olefin at variable concentration of DTAC ... S43

Aliphatic bromides in DTAC. ... S44

1-Bromohexane in micellar systems. ... S45

1-Bromooctane in micellar systems. ... S47

1-Bromooctan-2-ol in micellar systems. ... S48

8-Bromooctan-1-ol in micellar systems. ... S49

Product ***3a*** *in micellar systems.* ... S50

Vitamin B_{12} in micellar systems. ... S51

Alkyl cobalamin. ... S52

Organozinc compoud. ... S53

ROESY NMR spectra of olefin 1in micellar systems. ... S54

Olefin ***1*** *in micellar system over time.* ... S56

2D DOSY NMR spetra of vitamin B_{12} in micellar systems. ... S57

7.7. Computational Methods and Additional Results S58
7.8. Dynamic-light-scattering (DLS) measurements..... S59
8. NMR spectra S60
*diethyl 2-(1-phenyltetracosyl)malonate, **3b*** S60
*diethyl 2-(1-phenylicosyl)malonate, **3c*** S61
*diethyl 2-(1-phenylheptadecyl)malonate, **3d*** S62
*diethyl 2-(1-phenyltetradecyl)malonate, **3a*** S63
*diethyl 2-(1-phenyldodecyl)malonate, **3e*** S64
*diethyl 2-(1-phenyldecyl)malonate, **3f*** S65
*diethyl 2-(1-phenyloctyl)malonate, **3g*** S66
*diethyl 2-(1-phenylhexyl)malonate, **3h*** S67
*diethyl 2-(1-phenylbutyl)malonate, **3i*** S68
*diethyl 2-(3-cyclohexyl-1-phenylpropyl)malonate, **4*** S69
*diethyl 2-(4,4-dimethyl-1-phenylpentyl)malonate, **5*** S70
*diethyl 2-(3-methyl-1-phenylhexyl)malonate, **6*** S71
*diethyl 2-(2-cyclohexyl-1-phenylethyl)malonate, **7*** S72
*diethyl 2-(1,10-diphenyldecyl)malonate, **9*** S73
*4-dodecyl 1,1-diethyl 2-phenylbutane-1,1,4-tricarboxylate, **10a*** S74
*4-dodecyl 1,1-diethyl 1-phenylbutane-1,1,4-tricarboxylate, **10b*** S75
*4-dodecyl 1,1-diethyl (E)-1-phenylbut-2-ene-1,1,4-tricarboxylate, **10c*** S76
*6-dodecyl 1,1-diethyl 2-phenylhexane-1,1,6-tricarboxylate, **11a*** S77
*6-dodecyl 1,1-diethyl 1-phenylhexane-1,1,6-tricarboxylate, **11b*** S78
*triethyl 2-phenyldecane-1,1,10-tricarboxylate, **13*** S79
*diethyl 2-(4-(2-(2-methoxyethoxy)ethoxy)-1-phenylbutyl)malonate, **14*** S80
*diethyl 2-(4-hydroxy-1-phenyldecyl)malonate, **16a*** S81
*diethyl 2-(4-oxo-1-phenyldecyl)malonate, **16b*** S82
*diethyl 2-(4-hydroxydecyl)-2-phenylmalonate, **16c*** S83
*diethyl 2-(7-hydroxy-1-phenyldodecyl)malonate, **17*** S84
*diethyl 2-(10-hydroxy-1-phenyldecyl)malonate, **18*** S85
*diethyl 2-(1-(4-cyanophenyl)tetradecyl)malonate, **19a*** S86
*diethyl 2-(1-(4-(trifluoromethyl)phenyl)tetradecyl)malonate, **19b*** S87
*diethyl 2-(1-(4-methoxyphenyl)tetradecyl)malonate, **20a*** S89
*diethyl 2-(4-methoxyphenyl)-2-tetradecylmalonate, **20b*** S90
*diethyl 2-(1-(3-methoxyphenyl)tetradecyl)malonate, **21*** S91
*diethyl 2-(3-methoxyphenyl)-2-vinylmalonate, **S3*** S92
*diethyl 2-(4-(trifluoromethyl)phenyl)-2-vinylmalonate, **S5*** S93
*dodecyl 4-bromobutanoate, **S11*** S95

1. General Informations

All solvents and commercially available reagents were purchased as reagent grade and were used without further purification, unless otherwise stated. Yields refer to spectroscopically (^1H NMR) homogeneous materials. Reactions were monitored by thin layer chromatography (TLC), using 0.20 mm Merck silica plates (60F-254) and visualised using UV-light or potassium permanganate stain with heat as a developing agent. GC yields were calibrated with mesitilene as an internal standard. NMR spectra were recorded on Bruker 400 MHz or Varian 600 MHz and calibrated using residual undeuterated solvent ($CHCl_3$ – 7.26 ppm ^1H NMR, 77.16 ppm ^{13}C NMR, D_2O– 4.635 ppm ^1H NMR) or TMS as an internal reference. Chemical shifts are reported relatively in δ-scale as parts per million (ppm) referenced to the residual solvent peak. Coupling constants *J* are given in Hertz (Hz) and the following abbreviations were used for indicating signal multiplicity: ^1H NMR: s = singlet, d = doublet, t = triplet, q = quartet, m = multiplet and the respective combinations. Low-resolution mass spectra (LRMS) were recorded on an Applied Biosystems API 365 mass spectrometer using electrospray ionization (ESI) technique. High-resolution mass spectra (HRMS) were recorded on a Waters AutoSpec Premier instrument using electron ionization (EI) or a Waters SYNAPT G2-S HDMS instrument using electrospray ionization (ESI) or atmospheric-pressure chemical ionization (APCI) with time of flight detector (TOF). Melting points were recorded on a Marienfeld MPM-H2 melting point apparatus and are uncorrected. GC analyses were performed using Shimadzu GCMS-QP2010 SE with helium as the carrier gas and a Zebron ZB 5MSi column. (length: 30.0 m; thickness: 0.25 um, diameter: 0.25 mm).

GC program: time: 19.39 min; pressure: 121.8 kPa; total flow: 30.3 mL/min; column flow: 1.30 mL/min; linear velocity: 33.1 cm/s; purge flow: 3.0 mL/min; split ratio: 20.0.

	**rate**	**temperature [°C]**	**hold time**
0	-	100.0	1.00
1	40.00	180.0	1.50
2	40.00	260.0	1.50
3	45.00	300.0	1.00
4	50.00	325.0	9.00

Colum chromatography was performed using Merck silica gel 60 (230-400 mesh). Preparative HPLC separations were performed using Knauer HPLC chromatograph with PDA detector and Preparative column chromatography Knauer EII 100-10 Si column (250 x 20 mm). Flash column chromatography was performed on CombiFlash NextGen 300 Flash Chromatography System.

Flash program: time: 35 min; column: silica 4g; flow rate: 13 mL/min; automatic peak hold: on.

**entry**	**time [min]**	**hexane [%]**	**AcOEt [%]**
1	0	100	0
2	2	100	0
3	7	95	5
4	13	95	5
5	23	90	10
6	28	90	10
7	33	0	100
8	35	0	100

2. Setup for photoreactions

Reactions were carried out in a homemade photoreactors made of 400 mL beakers covered on the inside with LED tape. A cooling fan with adjustable spin rate was used to maintain temperature inside the photoreactor (40 °C).

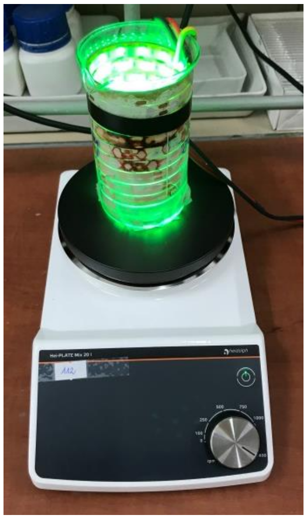

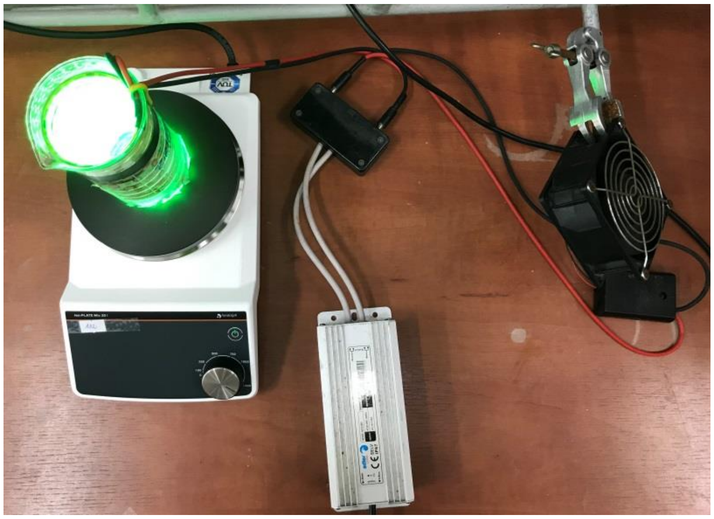

LED tapes characteristics:

Green LED tape: 10 mm SMD5050 LED strip, 60 LED diodes/m.

Power consumption: 10 W/m.

Green light – λ_{max} = 525 nm, 20 lm.

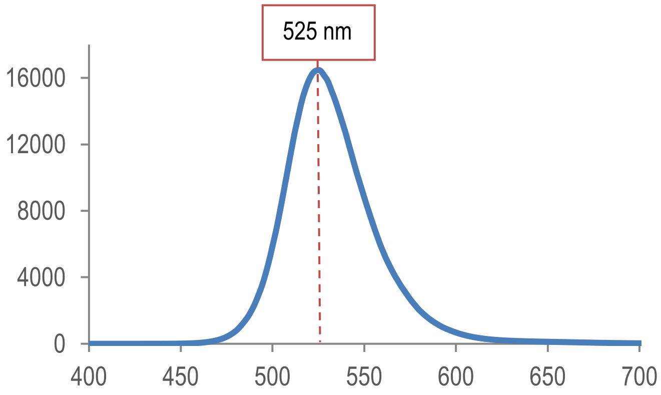

White LED tape: 8 mm SMD3528 LED strip, 120 LED diodes/m

Power consumption: 9.6 W/m

White light – 6500 K, 30 lm

3. Optimization studies

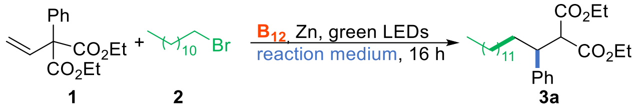

3.1. Background experiments[a]

entry	without	yield of 3a [%][b]	conversion of 1 [%][b]
1	hν	7	15
2	catalyst	0	0
3	Zn	0	0
4	NH_4Cl	51	100
5	B_{12} and hν	0	0
6	CTAB	4	16

[a]**Reaction conditions:** diethyl 2-phenyl-2-vinylmalonate (0.10 mmol), 1-bromododecane (5 equiv., 0.50 mmol), B_{12} (10 mol%, 0.01 mmol), Zn (3 equiv. 0.30 mmol), NH_4Cl (1.5 equiv., 0.15 mmol), CTAB (2.5 equiv., 0.25 mmol), H_2O (5 mL), white LEDs (6500 K), 16 h, 40 °C. [b] Calculated based on GC analysis. Mesitylene was used as an internal standard.

3.2. Type of cobalt catalyst[a]

entry	catalyst	yield of 3a [%][c]	conversion of 1 [%][c]
1	B_{12}	40 (49)[d]	93
2	HME	33	70
3[b]	$(CN)_2Cbi$	59	94
4[b]	Cobalester	21	58

[a]**Reaction conditions:** diethyl 2-phenyl-2-vinylmalonate (0.10 mmol), 1-bromododecane (5 equiv., 0.50 mmol), catalyst (10 mol%, 0.01 mmol), Zn (3 equiv. 0.30 mmol), NH_4Cl (1.5 equiv., 0.15 mmol), CTAB (2.5 equiv., 0.25 mmol), H_2O (5 mL), white LEDs (6500 K), 16 h, 40 °C. [b]**Reaction conditions:** diethyl 2-phenyl-2-vinylmalonate (0.10 mmol), 1-bromododecane (3 equiv., 0.30 mmol), catalyst (2.5 mol%, 0.003 mmol), Zn (3 equiv. 0.30 mmol), DTAC (2.5 equiv., 0.25 mmol), *n*-BuOH (12.5 equiv., 1.25 mmol) H_2O (5 mL), green LEDs (525 nm), 16 h, 40 °C. [c]Calculated based on GC analysis. Mesitylene was used as an internal standard. [d]Isolated yield.

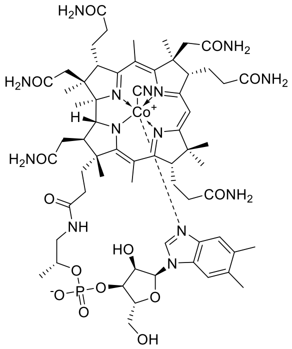

Vitamin B_{12}

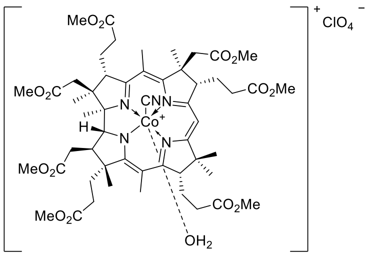

Aqua(cyano)heptamethyl cobyrinate
HME

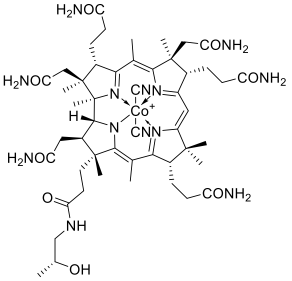

Cobinamide
$(CN)_2Cbi$

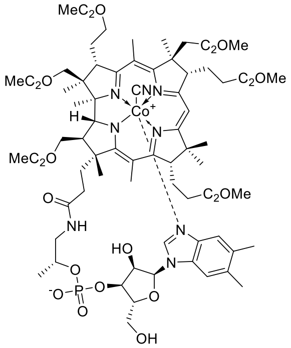

Cobalester

3.3. Screening of surfactant[a]

entry	surfactant	yield of 3a [%][d]	conversion of 1 [%][d]
1	DTAC	60 (61)[e]	94
2	DTAB	55	95
3	DTAI	48	89
4	CTAC	53	100
5	CTAB	40 (49)[e]	92
6	SLES	40	88
7	Triton X-100	37	99
8	SB3-14	33	100
9	PS-750-M	32	95
10	SDS	31	54
11	Triton X-45	30	61
12	STAB	20	31
13	Potassium laurate	5	35
14	Brij-35	10	100
15	TPGS-750-M	6	80
	TPGS-750-M[b]	43	100
16	Tween 60	5	98
17	Tween 20	2	98
18[b]	Polyoxyethanyl – α-tocopheryl	11	54
19[c]	AOT (B_{12})	0	9
	AOT (HME)	4	15

[a]**Reaction conditions:** diethyl 2-phenyl-2-vinylmalonate (0.10 mmol), 1-bromododecane (5 equiv., 0.50 mmol), vitamin B_{12} (10 mol%, 0.01 mmol,), Zn (3 equiv., 0.30 mmol), surfactant (2.5 equiv., 0.25 mmol), H_2O (5 mL), white LEDs (6500 K), 16 h, 40 °C. [b]**Reaction conditions:** diethyl 2-phenyl-2-vinylmalonate (0.10 mmol), 1-bromododecane (3 equiv., 0.3 mmol), B_{12} (2.5 mol%, 0.003 mmol), Zn (3 equiv., 0.30 mmol), surfactant (2.5 equiv., 0.25 mmol), *n*-BuOH (12.5 equiv., 1.25 mmol), H_2O (5 mL), green LEDs (525 nm), 16 h, 40 °C. [c]**Reaction conditions:** diethyl 2-phenyl-2-vinylmalonate (0.10 mmol), bromide (3 equiv., 0.30 mmol), B_{12} (2.5 mol%, 0.003 mmol), Zn (3 equiv., 0.30 mmol), surfactant (5 equiv., 0.50 mmol), H_2O (0.18 mL), *n*-heptane (5 mL), green LEDs (525 nm), 16 h, 40 °C. [d]Calculated based on GC analysis. Mesitylene was used as an internal standard. [e] Isolated yield.

3.4. Additives[a]

entry	additives and solvent	yield of 3a [%][b]	conversion of 1 [%][b]
1	methanol	57	86
2	ethanol	63	95
3	propan-1-ol	62	91
4	propan-2-ol$_{dry}$	63	95
5	propan-2-ol$_{p.a.grade}$	61	88
6	butan-1-ol	68	96
7	pentan-1-ol	66	93
8	2-butyl-1-octanol	49	89
9	nonan-1-ol	46	79
10	aceton	70	92
11	THF	70	95
12	PEG-200	61	97
13	MeCN : H_2O (1 : 1)	18	24
14	CTAB / dodecane / *n*-BuOH / H_2O (17.5 / 12.5 / 35 / 35 wt%)	51	65
15	DTAC / heksan / *n*-BuOH / H_2O (17.5 / 12.5 / 35 / 35 wt%)	45	60
16	CTAB / dodecane / *n*-BuOH / H_2O (17.5 / 12.5 / 35 / 35 wt%)	47	71
17	DTAC / *n*-BuOH / H_2O (33.3 / 33.3 / 33.3 wt%)	38	40

[a]**Reaction conditions:** diethyl 2-phenyl-2-vinylmalonate (0.10 mmol), 1-bromododecane (5 equiv., 0.50 mmol), vitamin B_{12} (10 mol%, 0.01 mmol), Zn (3 equiv., 0.30 mmol), DTAC (2.5 equiv.0.25 mmol), additives (40 equiv., 4 mmol), H_2O (5 mL), white LEDs (6500 K), 16 h, 40 °C. [b]Calculated based on GC analysis. Mesitylene was used as an internal standard.

3.5. Amount of DTAC[a]

entry	DTAC [mmol]	ratio of DTAC / *n*-BuOH / H_2O [wt%]	yield of 3a [%][b]	conversion of 1 [%][b]
1	0	0 / 0.2 / 99.8	26	51
2	0.05	0.2 / 5.6 / 94.2	43	67
3	0.15	0.7 / 5.6 / 93.7	67	91
4	0.25	1.2 / 5.6 / 93.2	69	96
5	0.35	1.7 / 5.6 / 92.7	71	99
6	0.95	4.5 / 5.6 / 89.9	71	98
7	3.41	14.5 / 5.6 / 79.9	66	93

[a]**Reaction conditions:** diethyl 2-phenyl-2-vinylmalonate (0.10 mmol), 1-bromododecane (5 equiv., 0.50 mmol), vitamin B_{12} (10 mol%, 0.01 mmol), Zn (3 equiv., 0.30 mmol), DTAC, *n*-BuOH (40 equiv., 4 mmol), H_2O (5 mL), white LEDs (6500 K), 16 h, 40 °C. [b]Calculated based on GC analysis. Mesitylene was used as an internal standard.

3.6. Amount of *n*-BuOH[a]

entry	*n*-BuOH [mmol]	ratio of DTAC / n-BuOH / H_2O [wt%]	yield of 3a [%][b]	conversion of 1 [%][b]
1	0	1.8 / 0.0 / 98.8	54	86
2	0.09	1.8 / 0.1 / 98.1	47	64
3	0.51	1.8 / 0.7 / 97.5	62	98
4	1.25	1.8 / 1.8 / 96.4	72	98
5	2.02	1.8 / 2.9 / 95.3	65	96
6	4.04	1.8 / 5.6 / 92.6	71	99
7	6.07	1.8 / 8.1 / 90.1	69	99

[a]**Reaction conditions:** diethyl 2-phenyl-2-vinylmalonate (0.10 mmol), 1-bromododecane (5 equiv., 0.50 mmol), vitamin B_{12} (10 mol%, 0.01 mmol), Zn (3 equiv., 0.30 mmol), DTAC (3.5 equiv., 0.35 mmol), *n*-BuOH, H_2O (5 mL), white LEDs (6500 K), 16 h, 40 °C. [b]Calculated based on GC analysis. Mesitylene was used as an internal standard.

3.7. Amount of water[a]

entry	DTAC [mmol]	H_2O [mL]	ratio of DTAC / *n*-BuOH / H_2O [wt%]	yield of 3a [%][b]	conversion of 1 [%][b]
1	0.35	7.5	1.2 / 1.2 / 97.6	62	97
2		5.0	1.8 / 1.8 / 96.4	72	98
3		2.5	3.4 / 3.4 / 93.2	68	98
4		1.0	7.8 / 7.8 / 84.4	57	76
5	0.53	7.5	1.8 / 1.8 / 96.4	68	94
6	0.35	5.0		72	98
7	0.175	2.5		65	100
8	0.07	1.0		60	96

[a]**Reaction conditions:** diethyl 2-phenyl-2-vinylmalonate (0.10 mmol), 1-bromododecane (5 equiv., 0.50 mmol), vitamin B_{12} (10 mol%, 0.01 mmol), Zn (3 equiv., 0.30 mmol), DTAC, *n*-BuOH (12.5 equiv., 1.25 mmol), H_2O, white LEDs (6500 K), 16 h, 40 °C. [b]Calculated based on GC analysis. Mesitylene was used as an internal standard.

3.8. Substrates ratio[a]

entry	olefin 1 [mmol]	bromide 2 [mmol]	ratio 1 : 2	yield of 3a [%][b]	conversion of 1 [%][b]
1	0.10	0.10	1 : 1	32	55
2	0.10	0.25	1 : 2.5	64	86
3	0.10	0.30	1 : 3	68	94
4	0.10	0.40	1 : 4	71	97
5	0.10	0.50	1 : 5	72	98
6	0.10	0.75	1 : 7.5	66	94
7	0.10	1.00	1 : 10	60	86

[a]**Reaction conditions:** diethyl 2-phenyl-2-vinylmalonate, 1-bromododecane, vitamin B_{12} (10 mol%, 0.01 mmol), Zn (3 equiv., 0.30 mmol), DTAC (3.5 equiv., 0.35 mmol), *n*-BuOH (12.5 equiv., 1.25 mmol), H_2O (5 mL), white LEDs (6500 K), 16 h, 40 °C. [b]Calculated based on GC analysis. Mesitylene was used as an internal standard.

3.9. The influence of light[a]

entry	light	yield of 3a [%][b]	conversion of 1 [%][b]
1	green LEDs (tape)	80	96
2	white LEDs (tape)	68	94
3	blue LEDs (tape)	56	80
4	violet LEDs (tape)	24	43
5	red LEDs (single diode)	42	66
6	green LEDs (single diode, 6 W)	34	56
7	white LEDs (single diode)	25	57
8	blue LEDs (single diode, 3 W)	12	21
9	violet LEDs (single diode, 40 W)	12	29
10	green LEDs (single diode, 40 W)	15	26
11	green LEDs (single diode, 20 W)	6	10
12	green LEDs (single diode, 10 W)	12	20
13	no light, 40 ˚C	7	12
14	no light, 70 ˚C	47	84
15	no light, 70 ˚C (microwave, 3h)	3	43

[a]**Reaction conditions:** diethyl 2-phenyl-2-vinylmalonate (0.10 mmol), 1-bromododecane (3 equiv., 0.30 mmol), vitamin B_{12} (10 mol%, 0.01 mmol), Zn (3 equiv., 0.30 mmol), DTAC (3.5 equiv., 0.35 mmol), *n*-BuOH (12.5 equiv., 1.25 mmol), H_2O (5 mL), light, 16h, 40 °C. [b]Calculated besed on GC analysis. Mesitylene was used as an internal standard.

3.10. Amount of vitamin B_{12} and Zn[a]

<table>
<tr><th>entry</th><th>B₁₂ [mol%]</th><th>Zn [equiv.]</th><th>yield of 3a [%][b]</th><th>conversion of 1[%][b]</th></tr>
<tr><td>1</td><td>1</td><td rowspan="5">3</td><td>66</td><td>96</td></tr>
<tr><td>2</td><td>2.5</td><td>80</td><td>98</td></tr>
<tr><td>3</td><td>5</td><td>80</td><td>96</td></tr>
<tr><td>4</td><td>10</td><td>80</td><td>97</td></tr>
<tr><td>5</td><td>15</td><td>61</td><td>94</td></tr>
<tr><td>6</td><td rowspan="6">2.5</td><td>1</td><td>53</td><td>77</td></tr>
<tr><td>7</td><td>2</td><td>57</td><td>85</td></tr>
<tr><td>8</td><td>3</td><td>80</td><td>98</td></tr>
<tr><td>9</td><td>4</td><td>63</td><td>78</td></tr>
<tr><td>10</td><td>5</td><td>63</td><td>75</td></tr>
<tr><td>11</td><td>6</td><td>44</td><td>50</td></tr>
</table>

[a]**Reaction conditions:** diethyl 2-phenyl-2-vinylmalonate (0.10 mmol,), 1-bromododecane (3 equiv., 0.30 mmol), vitamin B_{12}, Zn, DTAC (3.5 equiv., 0.35 mmol), *n*-BuOH (12.5 equiv., 1.25 mmol), H_2O (5 mL), green LEDs (525 nm), 16 h, 40 °C. [b]Calculated based on GC analysis. Mesitylene was used as an internal standard.

3.11. Screening of reductant[a]

entry	reductant	yield of 3a [%][b]	conversion of 1 [%][b]
1	Zn	80	96
2	$NaBH_4$	7	74
3	Mn	1	7

[a]**Reaction conditions:** diethyl 2-phenyl-2-vinylmalonate (0.10 mmol), 1-bromododecane (3 equiv., 0.30 mmol), vitamin B_{12} (2.5 mol%, 0.003 mmol), reductant (3 equiv., 0.30 mmol), DTAC (3.5 equiv., 0.35 mmol), *n*-BuOH (12.5 equiv., 1.25 mmol), H_2O (5 mL), green LEDs (525 nm), 16 h, 40 °C. [b]Calculated based on GC analysis. Mesitylene was used as an internal standard.

3.12. Influence of buffer[a]

entry	buffer	pH	yield of 3a [%][c]	conversion of 1 [%][c]
1	-	-	80	96
2[b]	acetate	5.0	70	90
3	acetate	5.0	67	87
4	acetate	4.0	43	56
5[b]	acetate	4.0	42	60
6	PBS	7.4	3	8

[a]**Reaction conditions:** diethyl 2-phenyl-2-vinylmalonate (0.1 mmol), 1-bromododecane (3 equiv., 0.30 mmol), vitamin B_{12} (2.5 mol%, 0.003 mmol), Zn (3 equiv., 0.30 mmol), DTAC (3.5 equiv., 0.35 mmol), *n*-BuOH (12.5 equiv., 1.25 mmol), buffer (5 mL), green LEDs (525 nm), 16 h, 40 °C.[b]48 h. [c]Calculated based on GC analysis. Mesitylene was used as an internal standard.

3.13. Reaction time[a]

entry	time [h]	yield of 3a [%][b]	conversion of 1 [%][b]	conversion of 2 [%][b]
1	0.5	6	9	7
2	1	17	22	30
3	2	32	39	35
4	5	60	80	61
5	8	73	90	92
6	16	80 (76)[c]	96	100
7	40	76	99	100
8	64	77	95	100

[a]**Reaction conditions:** diethyl 2-phenyl-2-vinylmalonate (0.10 mmol), 1-bromododecane (3 equiv., 0.30 mmol), vitamin B_{12} (2.5 mol%, 0.003 mmol), Zn (3 equiv., 0.30 mmol), DTAC (3.5 equiv., 0.35 mmol), *n*-BuOH (12.5 equiv.1.25 mmol), H_2O (5 mL), green LEDs (525 nm), time, 40 °C. [b]Calculated based on GC analysis. Mesitylene was used as an internal standard.[c]Isloated yield.

4. Preparation of starting materials (S1-S11) and characterization of new compounds

Not commercially available substrates were synthesized according to the reported procedures. [1-6] The observed characterization data (^{1}H and ^{13}C NMR) are consistent with those previously reported.

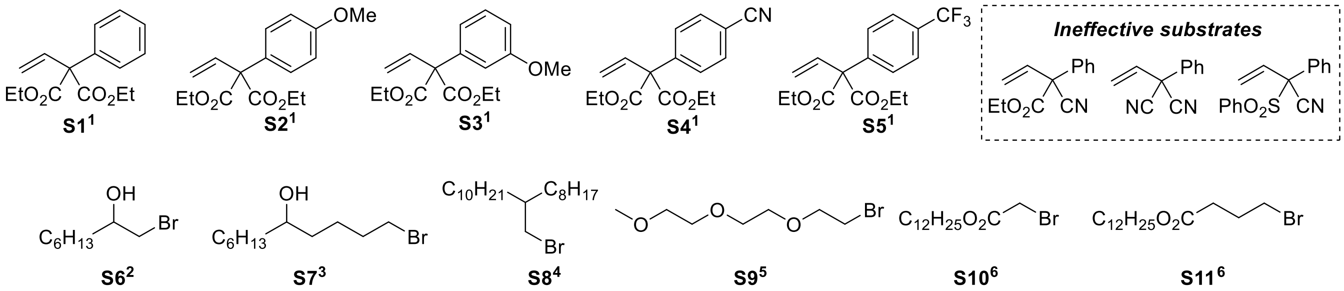

diethyl 2-(3-methoxyphenyl)-2-vinylmalonate, S3

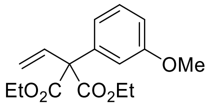

Following the reported procedure,[1] **S3** was obtained from 1-iodo-3-methoxybenzene (2.34 g, 10 mmol) and diethyl malonate (3.20 g, 20 mmol, 2 equiv.) to afford 439 mg of diethyl 2-(3-methoxyphenyl)-2-vinylmalonate as colorless oil, (yield = 15 %, after three steps).

^{1}H NMR (600 MHz, $CDCl_3$) δ 7.27 – 7.24 (m, 1H), 7.00 – 6.94 (m, 2H), 6.87 – 6.82 (m, 1H), 6.56 (dd, *J* = 17.7, 10.8 Hz, 1H), 5.42 (d, *J* = 10.8 Hz, 1H), 5.10 (d, *J* = 17.6 Hz, 1H), 4.26 (qd, *J* = 7.1, 1.1 Hz, 4H), 3.79 (s, 3H), 1.27 (t, *J* = 7.1 Hz, 6H).

^{13}C NMR (126 MHz, $CDCl_3$) δ 169.6, 159.3, 138.3, 136.2, 129.1, 121.3, 118.5, 115.2, 113.1, 66.3, 62.0, 55.4, 14.1.

HRMS (ESI) m/z $[M + Na]^+$ calcd for $C_{16}H_{20}O_5Na$ 315.1208, found 315.1214.

GC chromatogram:

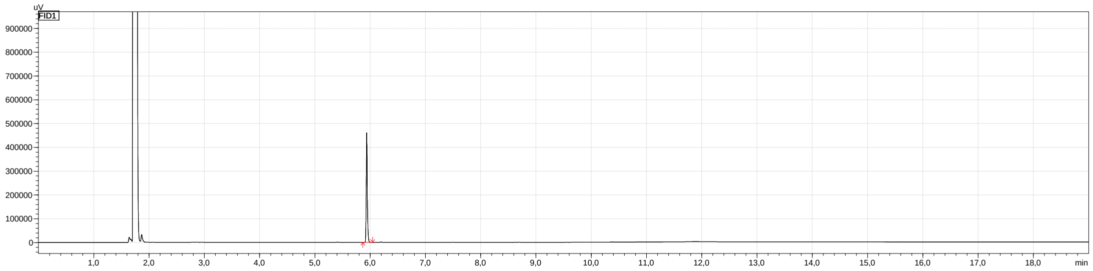

[1] Li, Z.; Wang, M.; Shi, Z. *Angew. Chemie Int. Ed.* **2021**, *60* (1), 186–190.

[2] Zav'yalov, S. I.; Sitkareva, I. V. and Ezhova, G. I.; *Russ Chem Bull,* **1989**, *38*, 127–130.

[3a] Prasanth C. P.; Ebbin J.; Abhijith A.; Nair D. S.; Ibnusaud I.; Raskatov , J.; Singaram B. *J. Org. Chem.* **2018**, *83*, 3, 1431–1440.

[3b] Tian, B.; Li, X.; Chen, P.; Liu, G. *Angew. Chem. Int. Ed.* **2021**, *60*, 14881 –14886.

[4] Chen, M.; Li, J.; Jiao, X.; Yang, X.; Wu, W.; McNeill; C. R. and Gao, X.; *J. Mater. Chem. C*, **2019**, *7*, 2659-2665.

[5] Karimi, B.; Mansouri, F.; and Vali, H, *Green Chem.*, **2014**, *16*, 2587-2596.

[6] Boz M.; Baştűrk S. S. *J Surfact Deterg.* **2016**, *19*, 663–671.

diethyl 2-(4-(trifluoromethyl)phenyl)-2-vinylmalonate, S5

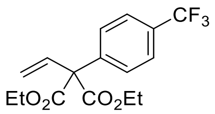

Following the general procedure,[1] compound **S5** was obtained from 1-iodo-4-(trifluoromethyl)benzene (3.17 g, 10 mmol) and diethyl malonate (3.20 g, 20 mmol, 2 equiv.) to afford 83 mg of diethyl 2-(4-(trifluoromethyl)phenyl)-2-vinylmalonate as colorless oil, (yield = 5 %, after three steps).

^{1}H NMR (500 MHz, $CDCl_3$) δ 7.62 – 7.58 (m, 2H), 7.56 – 7.51 (m, 2H), 6.59 (ddd, *J* = 17.7, 10.8, 1.4 Hz, 1H), 5.47 (dd, *J* = 10.7, 1.3 Hz, 1H), 5.08 (d, *J* = 17.5 Hz, 1H), 4.28 (qd, *J* = 7.1, 1.9 Hz, 4H), 1.27 (t, *J* = 7.2, 1.2 Hz, 6H).

^{13}C NMR (125 MHz, $CDCl_3$) δ 169.2, 140.9, 140.9, 135.7, 130.1 (q_{CF3}, *J* = 32.2 Hz), 129.8, , 129.5, 125.1 (q_{CF3}, *J* = 3.8 Hz), 124.2 (q_{CF3}, *J* = 272.7, 271.7, 271.6 Hz), 119.3, 66.1, 62.4, 14.1.

^{19}F NMR (470 MHz, $CDCl_3$) δ -62.76 (s, 3F).

HRMS (ESI) m/z $[M + Na]^+$ calcd for $C_{16}H_{17}F_3O_4Na$ 353.0977, found 353.0979.

GC chromatogram:

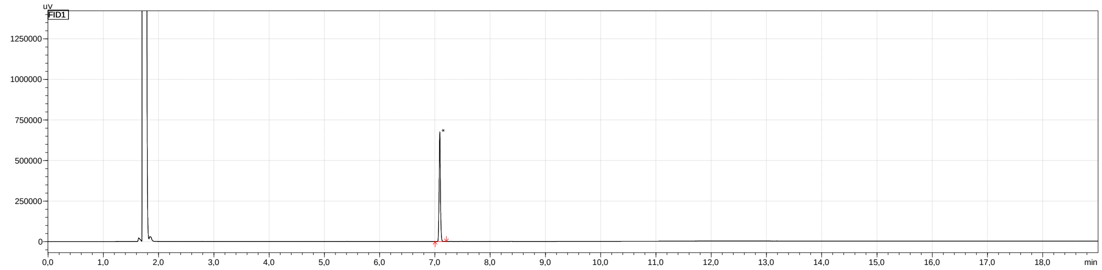

dodecyl 4-bromobutanoate, S11

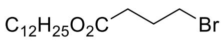

Following the reported procedure,[6] **S11** was obtained from 1-dodecanol (1.86 g, 10 mmol) and 4-bromobutanoyl chloride (3.71 g, 20 mmol, 2 equiv.) to afford 2.5 g of dodecyl 4-bromobutanoate as colorless oil, (yield = 75 %).

^{1}H NMR (500 MHz, $CDCl_3$) δ 4.07 (t, *J* = 6.8 Hz, 2H), 3.46 (t, *J* = 6.5 Hz, 2H), 2.49 (t, *J* = 7.2 Hz, 2H), 2.17 (p, *J* = 6.8 Hz, 2H), 1.64 – 1.59 (m, 2H), 1.34 – 1.24 (m, 18H), 0.87 (t, *J* = 6.9 Hz, 3H).

^{13}C NMR (126 MHz, $CDCl_3$) δ 172.7, 64.9, 32.8, 32.7, 32.1, 29.77, 29.75, 29.7, 29.6, 29.5, 29.4, 28.8, 28.0, 26.0, 22.8, 14.2.

HRMS (ESI) m/z $[M + Na]^+$ calcd for $C_{16}H_{31}O_2NaBr$ 357.1405, found 357.1402.

GC chromatogram:

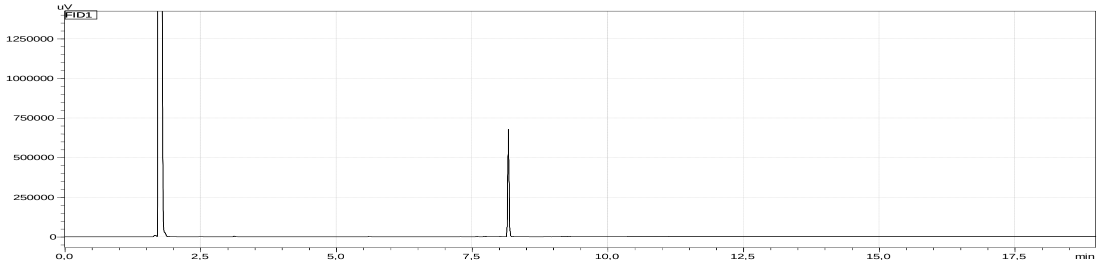

5. General Procedures for Tandem Radical Addition/1,2-Aryl Migration Reaction

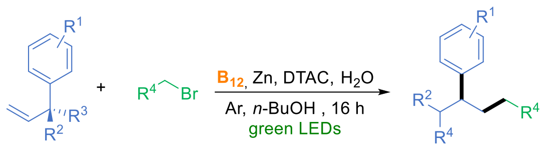

Reaction conditions: olefin (0.10 mmol), bromide (3 equiv, 0.30 mmol), vitamin B_{12} (2.5 mol%, 0.003 mmol), Zn (3 equiv., 0.30 mmol), DTAC (3.5 equiv., 0.35 mmol,), *n*-BuOH (12.5 mmol, 1.25 mmol,), H_2O (5 mL), green LEDs (525 nm), 16 h, 40 °C.

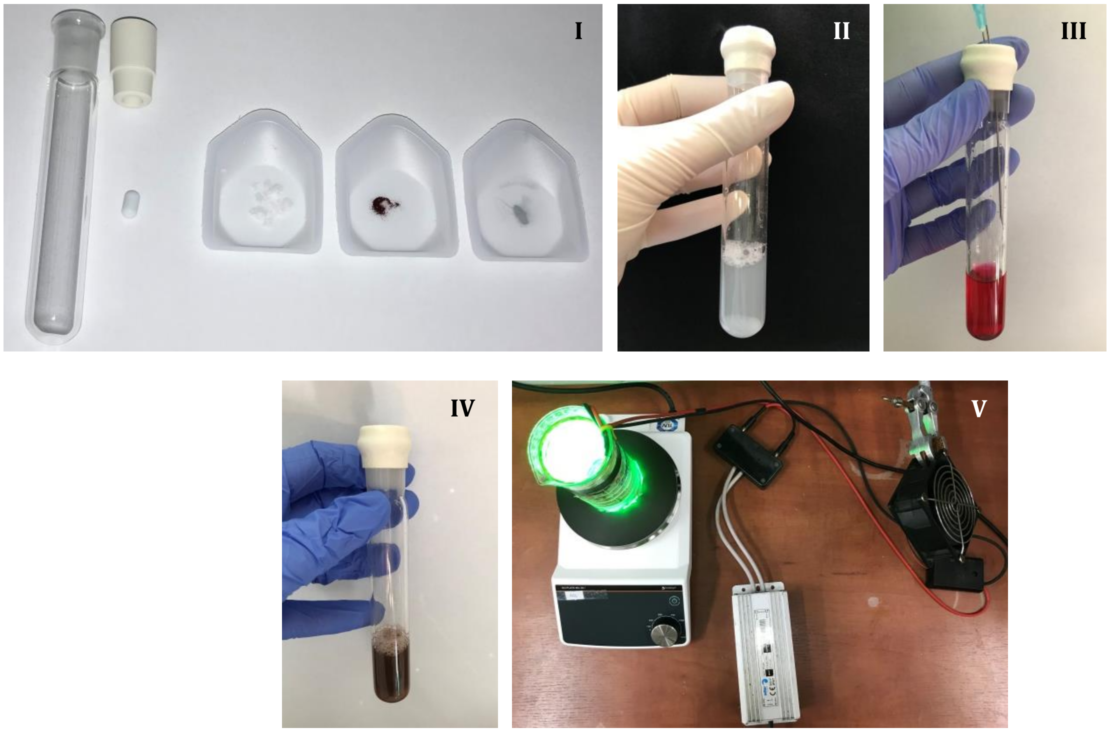

Figure S1. Graphics for the main reaction steps. I) Reaction tubes and weighted sample of DTAC, vitamin B_{12} and Zn; II) Zn in DTAC solution in H_2O; III) vitamin B_{12} and Zn in DTAC solution in H_2O - mixture before degassing, red; IV) vitamin B_{12} and Zn in DTAC solution in H_2O – mixture after degassing, dark green or brown; V) setup for photoreactions.

5.1. General procedure for liquid substrats:

A glass reaction tube (inner diameter = 18 mm) equipped with a magnetic bar was charged with DTAC (92.4 mg, 0.35 mmol, 3.5 equiv.), vitamin B_{12} (3.4 mg, 0.003 mmol, 2.5 mol%) and activated zinc (19.6 mg, 0.30 mmol, 3.0 equiv.), then distillated H_2O (5 mL) and *n*-BuOH (114.2 µL, 1.25 mmol, 12.5 equiv.) were added. The tube was sealed with a septum and the resulting mixture was degassed by purging the solution with argon for 20 min with simultaneous sonication in an ultrasonic bath (the solution turned from red to dark green or brown). Subsequently, an electron-deficient olefin (0.10 mmol, 1.0 equiv.) and bromide (0.30 mmol, 3.0 equiv.) were added and the reaction vessel was placed in a photoreactor and irradiated with green LEDs (tape, 525 nm) for 16 h. The resulting mixture was diluted with brine, extracted with AcOEt (3 x 10 mL), and washed with water (10 mL). The organic phase was dried over Na_2SO_4, then filtered through the cotton wool and concentrated in vacuo. A crude product was purified using flash column chromatography with hexanes/AcOEt eluent system. If necessary, product was repurified using preparative HPLC with hexanes/AcOEt eluent system.

5.2. General procedure for solid substrates:

A glass reaction tube (inner diameter = 18 mm) equipped with a magnetic bar was charged with DTAC (92.4 mg, 0.35 mmol, 3.5 equiv.), vitamin B_{12} (3.4 mg,0.003 mmol, 2.5 mol%) an electron-deficient olefin (0.10 mmol, 1.0 equiv.) and activated zinc (19.6 mg, 0.30 mmol, 3.0 equiv.), then distillated H_2O (5 mL) and *n*-BuOH (114.2 μL, 1.25 mmol, 12.5 equiv.) were added. The tube was sealed with a septum and the resulting mixture was degassed by purging the solution with argon for 20 min with simultaneous sonication in an ultrasonic bath (the solution turned from red to dark green or brown). Subsequently, bromide (0.30 mmol, 3.0 equiv.) was added and the reaction vessel was placed in a photoreactor and irradiated with green LEDs (tape, 525 nm) for 16 h. The resulting mixture was diluted with brine, extracted with AcOEt (3 x 10 mL), and washed with water (10 mL). The organic phase was dried over Na_2SO_4, then filtered through the cotton wool and concentrated in vacuo. A crude product was purified using flash column chromatography with hexanes/AcOEt eluent system. If necessary, product was repurified using preparative HPLC with hexanes/AcOEt eluent system.

5.3. Notes:

- ✓ The reaction can be easily monitored by GC and TLC chromatography (AcOEt/Hexane) using UV visualization and $KMnO_4$ stain;
- ✓ Reactions require using activated zinc (unactivated zinc gives a lower yield);[7]
- ✓ The mixture containing Zn, Cobalt-catalyst (vitamin B_{12}) and a solvent should turned from red to dark green and finally brown. The color indicates the reduction of the cobalt from Co(III) to Co(I) oxidation state;
- ✓ If the color of the reaction does not change (from red to dark brown/green), we highly recommend to repeat zinc activation step.

[7] M. Ociepa; O. Baka; J. Narodowiec; D. Gryko; *Adv. Synth. Catal.* **2017**, *359*, 3560 –3565.

6. Products and characterization of new compounds

diethyl 2-(1-phenyltetracosyl)malonate, 3b

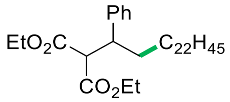

Following the general procedure 5.2 compound **3b** was obtained from diethyl 2-phenyl-2-vinylmalonate (26 mg, 0.10 mmol) and 1-bromodocosane (117 mg, 0.30 mmol). The crude product was purified by flash column chromatography to afford 8 mg of diethyl 2-(1-phenyltetracosyl)malonate as white solid, (yield = 14 %, mp = 64 °C).

^{1}H NMR (500 MHz, $CDCl_3$) δ 7.28 – 7.25 (m, 2H), 7.21 – 7.16 (m, 3H), 4.24 (q, *J* = 7.1 Hz, 2H), 3.86 (q, *J* = 7.1 Hz, 2H), 3.61 (d, *J* = 10.9 Hz, 1H), 3.34 (td, *J* = 10.9, 3.7 Hz, 1H), 1.68 – 1.58 (m, 2H), 1.31 – 1.16 (m, 41H), 1.14 – 0.95 (m, 4H), 0.93 (t, *J* = 7.1 Hz, 3H), 0.88 (t, *J* = 6.8 Hz, 3H).

^{13}C NMR (125 MHz, $CDCl_3$) δ 168.7, 168.05, 141.2, 128.5, 128.4, 126.9, 61.6, 61.2, 59.1, 45.8, 34.1, 32.1, 29.85, 29.83, 29.81, 29.80, 29.75, 29.7, 29.5, 29.51, 29.49, 27.2, 22.8, 14.3, 13.8.

HRMS (ESI) *m/z* $[M + Na]^+$ calcd for $C_{37}H_{64}O_4Na$ 595.4702, found 595.4695.

GC chromatogram:

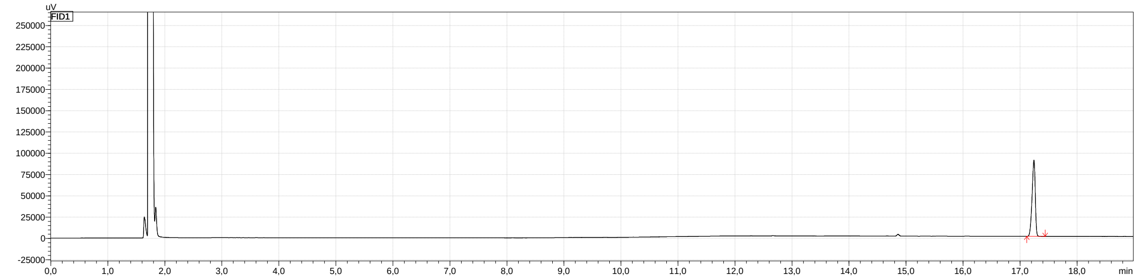

diethyl 2-(1-phenylicosyl)malonate, 3c

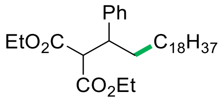

Following the general procedure 5.2 compound **3c** was obtained from diethyl 2-phenyl-2-vinylmalonate (26 mg, 0.10 mmol) and 1-bromooctadecane (100 mg, 0.30 mmol). The crude product was purified by flash column chromatography to afford 35 mg of diethyl 2-(1-phenyltetracosyl)malonate as white solid, (yield = 67 %, mp = 53 °C).

With C_{18}TAC instead of DTAC, yield = 41 %.

^{1}H NMR (500 MHz, $CDCl_3$) δ 7.28 – 7.25 (m, 2H), 7.19 (m, 3H), 4.24 (q, *J* = 7.1 Hz, 2H), 3.89 – 3.84 (m, 2H), 3.61 (d, *J* = 10.9 Hz, 1H), 3.34 (td, *J* = 10.9, 3.7 Hz, 1H), 1.70 – 1.57 (m, 2H), 1.30 – 1.14 (m, 37H), 0.93 (t, *J* = 7.1 Hz, 3H), 0.88 (t, *J* = 6.9 Hz, 3H).

^{13}C NMR (125 MHz, CDCl3) δ 168.7, 168.1, 141.2, 128.5, 128.4, 126.9, 61.6, 61.2, 59.1, 45.8, 34.1, 32.1, 29.84, 29.82, 29.80, 29.79, 29.74, 29.69, 29.53, 29.51, 29.49, 27.2, 22.8, 14.27, 14.25, 13.8.

HRMS (ESI) *m/z* $[M + Na]^+$ calcd for $C_{33}H_{56}O_4Na$ 539.4076, found 539.4080.

GC chromatogram:

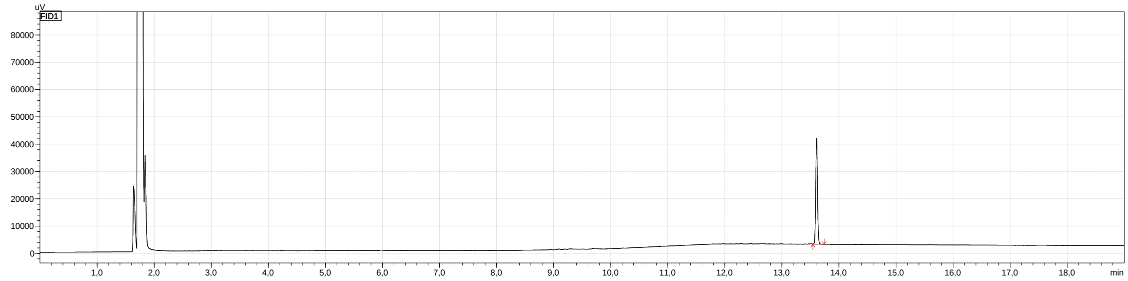

diethyl 2-(1-phenylheptadecyl)malonate, 3d

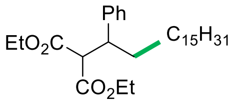

Following the general procedure 5.2 compound **3d** was obtained from diethyl 2-phenyl-2-vinylmalonate (26 mg, 0.10 mmol) and 1-bromopentadecane (87 mg, 0.30 mmol). The crude product was purified by flash column chromatography to afford 30 mg of diethyl 2-(1-phenylheptadecyl)malonate as white solid, (yield = 63 %, mp = 38 °C).

^{1}H NMR (500 MHz, $CDCl_3$) δ 7.26 (m, 2H), 7.19 (m, 3H), 4.24 (q, *J* = 7.1 Hz, 2H), 3.86 (qd, *J* = 7.1, 1.1 Hz, 2H), 3.61 (d, *J* = 10.9 Hz, 1H), 3.34 (td, *J* = 10.9, 3.7 Hz, 1H), 1.69 – 1.58 (m, 2H), 1.30 – 1.10 (m, 31H), 0.93 (t, *J* = 7.1 Hz, 3H), 0.88 (t, J = 6.9 Hz, 3H).

^{13}C NMR (125 MHz, $CDCl_3$) δ 168.7, 168.0, 141.2, 128.5, 128.4, 126.9, 61.6, 61.2, 59.1, 45.80 34.1, 32.1, 29.8, 29.8, 29.8, 29.79, 29.78, 29.7, 29.7, 29.52, 29.49, 29.47, 27.2, 22.8, 14.3, 14.2, 13.8.

HRMS (ESI) *m/z* $[M + Na]^+$ calcd for $C_{30}H_{50}O_4Na$ 497.3607, found 497.3613.

GC chromatogram:

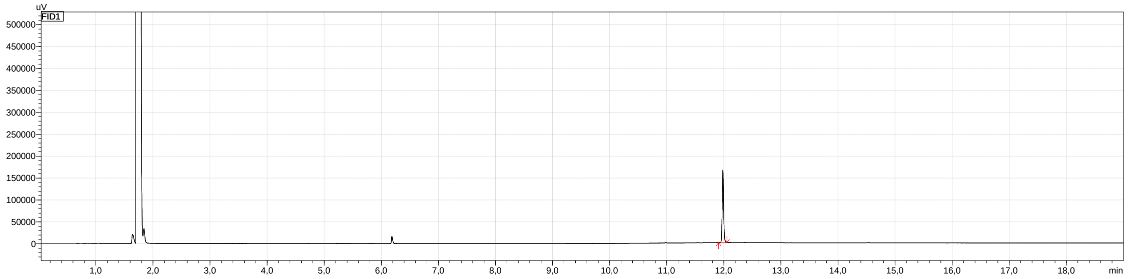

diethyl 2-(1-phenyltetradecyl)malonate, 3a

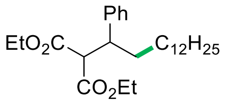

Following the general procedure 5.1 compound **3a** was obtained from diethyl 2-phenyl-2-vinylmalonate (26 mg, 0.10 mmol) and 1-bromododecane (75 mg, 0.30 mmol). The crude product was purified by flash column chromatography to afford 33 mg of diethyl 2-(1-phenyltetradecyl)malonate as colorless oil, (yield = 76 %).

With: C_{18}TAC instead of DTAC, yield = 50 %; C_8TAC instead of DTAC, yield = 7 %.

^{1}H NMR (600 MHz, $CDCl_3$) δ 7.27 – 7.23 (m, 2H), 7.20 – 7.15 (m, 3H), 4.22 (q, *J* = 7.1 Hz, 2H), 3.85 (qd, *J* = 7.1, 1.3 Hz, 2H), 3.60 (d, *J* = 10.9 Hz, 1H), 3.33 (td, *J* = 11.0, 3.6 Hz, 1H), 1.67 – 1.58 (m, 2H), 1.29 – 0.99 (m, 25H), 0.91 (t, *J* = 7.1 Hz, 3H), 0.86 (t, *J* = 7.1 Hz, 3H).

^{13}C NMR (151 MHz, $CDCl_3$) δ 168.7, 168.0, 141.1, 128.5, 128.4, 126.9, 61.6, 61.2, 59.1, 45.8, 34.1, 32.1, 29.80, 29.76, 29.72, 29.66, 29.51, 29.48, 29.46, 27.2, 22.8, 14.3, 13.8.

HRMS (ESI) *m/z* $[M + Na]^+$ calcd for $C_{27}H_{44}O_4Na$ 455.3137, found 455.3140.

GC chromatogram:

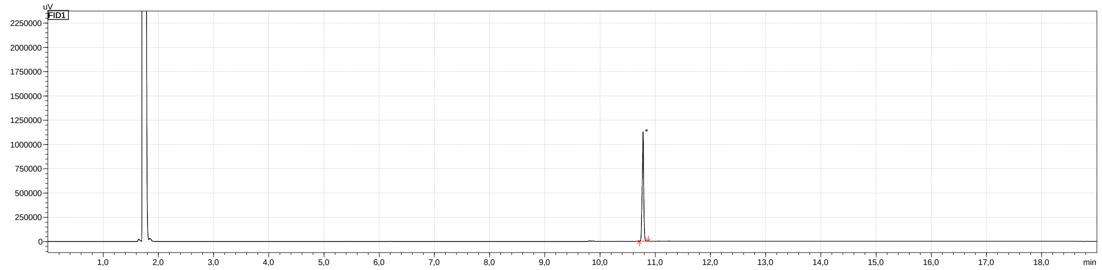

diethyl 2-(1-phenyldodecyl)malonate, 3e

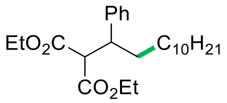

Following the general procedure 5.1 compound **3e** was obtained from diethyl 2-phenyl-2-vinylmalonate (26 mg, 0.10 mmol) and 1-bromodecane (66 mg, 0.30 mmol). The crude product was purified by flash column chromatography to afford 29 mg of diethyl 2-(1-phenyldodecyl)malonateas as colorless oil, (yield = 72 %).

^{1}H NMR (500 MHz, $CDCl_3$) δ 7.28 – 7.25 (m, 2H), 7.21 – 7.17 (m, 3H), 4.24 (q, *J* = 7.1 Hz, 2H), 3.86 (qd, *J* = 7.1, 1.1 Hz, 2H), 3.61 (d, *J* = 10.9 Hz, 1H), 3.34 (td, *J* = 10.9, 3.7 Hz, 1H), 1.68 – 1.58 (m, 2H), 1.30 – 1.08 (m, 21H), 0.93 (t, *J* = 7.1 Hz, 3H), 0.87 (t, *J* = 7.0 Hz, 3H).

^{13}C NMR (126 MHz, $CDCl_3$) δ 168.7, 168.0, 128.5, 128.4, 126.9, 61.6, 61.2, 59.1, 45.8, 34.1, 32.0, 29.72, 29.66, 29.51, 29.47, 29.45, 27.2, 22.8, 14.3, 14.2, 13.8.

HRMS (ESI) *m/z* $[M + Na]^+$ calcd for $C_{25}H_{40}O_4Na$ 427.2824, found 427.2824.

GC chromatogram:

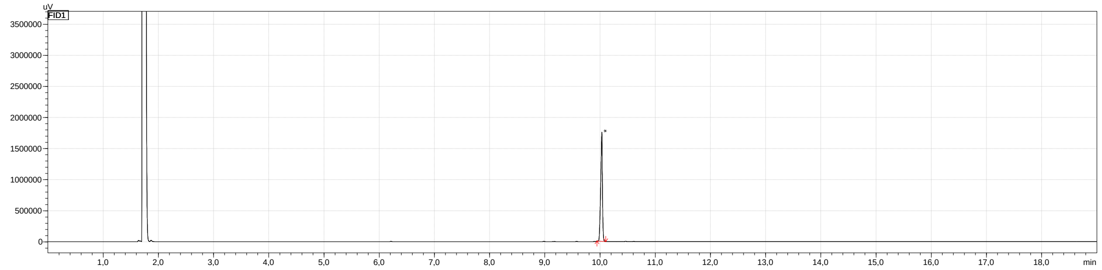

diethyl 2-(1-phenyldecyl)malonate, 3f

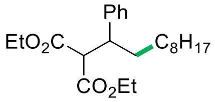

Following the general procedure 5.1 compound **3f** was obtained from diethyl 2-phenyl-2-vinylmalonate (26 mg, 0.1 mmol) and 1-bromooctane (58 mg, 0.30 mmol). The crude product was purified by flash column chromatography to afford 24 mg of diethyl 2-(1-phenyldecyl)malonate as colorless oil, (yield = 63 %).

With: C_8TAC instead of DTAC, yield = 4 %; C_8TAC (0.70 mmol) instead of DTAC, yield = 17 %; C_8TAC (1.05 mmol) instead of DTAC, yield = 10 %.

^{1}**H NMR** (500 MHz, $CDCl_3$) δ 7.28 – 7.24 (m, 2H), 7.21 – 7.17 (m, 3H), 4.24 (q, *J* = 7.1 Hz, 2H), 3.86 (qd, *J* = 7.1, 1.1 Hz, 2H), 3.61 (d, *J* = 11.0 Hz, 1H), 3.34 (td, *J* = 10.9, 3.7 Hz, 1H), 1.69 – 1.58 (m, 2H), 1.30 – 1.05 (m, 17H), 0.93 (t, *J* = 7.1 Hz, 3H), 0.86 (t, *J* = 7.1 Hz, 3H).

^{13}**C NMR** (125 MHz, $CDCl_3$) δ 168.7, 168.0, 141.2, 128.5, 128.4, 126.9, 61.6, 61.2, 59.1, 45.8, 34.1, 32.0, 29.6, 29.51, 29.46, 29.37, 27.2, 22.8, 14.3, 14.2, 13.8.

HRMS (ESI) *m/z* $[M + Na]^+$ calcd for $C_{23}H_{36}O_4Na$ 399.2511, found 399.2520.

GC chromatogram:

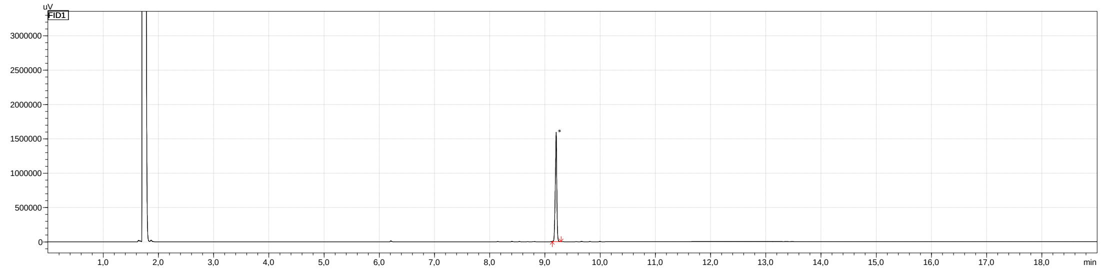

diethyl 2-(1-phenyloctyl)malonate, 3g

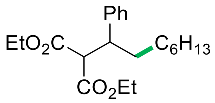

Following the general procedure 5.1 compound **3g** was obtained from diethyl 2-phenyl-2-vinylmalonate (26 mg, 0.10 mmol) and 1-bromohexane (50 mg, 0.30 mmol). The crude product was purified by flash column chromatography to afford 16 mg of diethyl 2-(1-phenyloctyl)malonate as colorless oil, (yield = 46 %).

^{1}**H NMR** (500 MHz, $CDCl_3$) δ 7.28 – 7.25 (m, 2H), 7.22 – 7.17 (m, 3H), 4.24 (q, *J* = 7.2 Hz, 2H), 3.86 (qd, *J* = 7.1, 1.1 Hz, 2H), 3.61 (d, *J* = 10.9 Hz, 1H), 3.35 (td, *J* = 10.9, 3.7 Hz, 1H), 1.69 – 1.58 (m, 2H), 1.29 (t, *J* = 7.2 Hz, 3H), 1.25 – 1.03 (m, 10H), 0.93 (t, *J* = 7.1 Hz, 3H), 0.83 (t, *J* = 7.1 Hz, 3H).

^{13}**C NMR** (125 MHz, $CDCl_3$) δ 168.7, 168.0, 141.2, 128.5, 128.4, 126.9, 61.6, 61.2, 59.1, 45.8, 34.1, 31.9, 29.4, 29.17, 27.16, 22.7, 14.3, 14.2, 13.8.

HRMS (ESI) *m/z* $[M + Na]^+$ calcd for $C_{21}H_{32}O_4Na$ 371.2198, found 371.2201.

GC chromatogram:

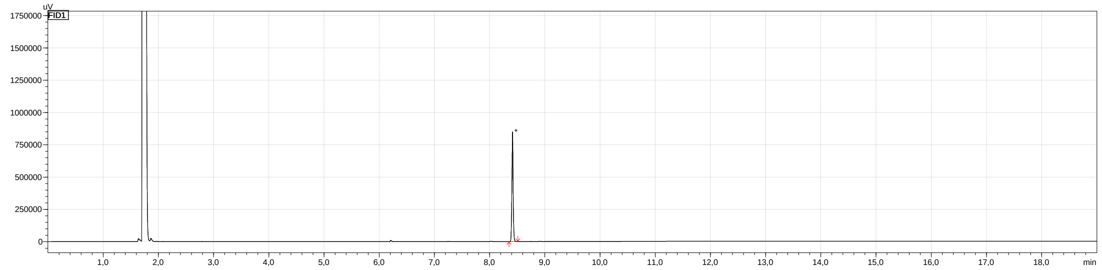

diethyl 2-(1-phenylhexyl)malonate, 3h

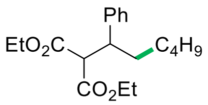

Following the general procedure 5.1 compound **3h** was obtained from diethyl 2-phenyl-2-vinylmalonate (26 mg, 0.10 mmol) and 1-bromobutane (41 mg, 0.30 mmol). The crude product was purified by flash column chromatography to afford 12 mg of diethyl 2-(1-phenylhexyl)malonate as colorless oil, (yield = 37 %).

^{1}H NMR (600 MHz, $CDCl_3$) δ 7.28 – 7.25 (m, 2H), 7.22 – 7.17 (m, 3H), 4.24 (q, *J* = 7.1 Hz, 2H), 3.86 (qd, *J* = 7.1, 1.4 Hz, 2H), 3.61 (d, *J* = 11.0 Hz, 1H), 3.35 (td, *J* = 11.0, 3.7 Hz, 1H), 1.69 – 1.59 (m, 2H), 1.29 (t, *J* = 7.2 Hz, 3H), 1.25 – 1.04 (m, 6H), 0.93 (t, *J* = 7.1 Hz, 3H), 0.80 (t, 3H).

^{13}C NMR (126 MHz, $CDCl_3$) δ 168.7, 168.1, 141.2, 128.5, 128.4, 126.9, 61.6, 61.2, 59.1, 45.8, 34.1, 31.7, 26.8, 22.5, 14.3, 14.1, 13.8.

HRMS (ESI) *m/z* $[M + Na]^+$ calcd for $C_{19}H_{28}O_4Na$ 343.1885, found 343.1889.

GC chromatogram:

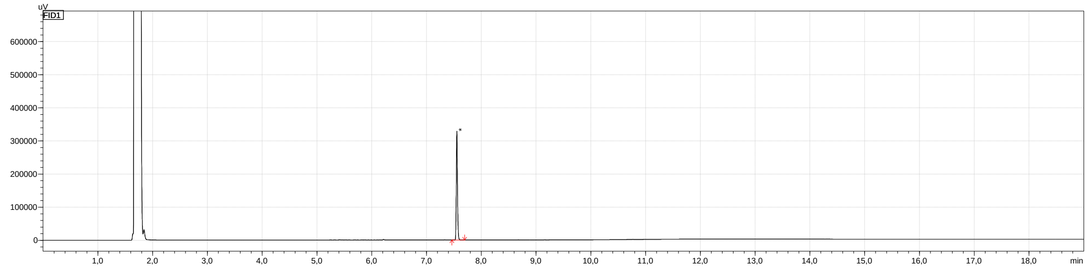

diethyl 2-(1-phenylbutyl)malonate, 3i

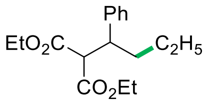

Following the general procedure 5.1 compound **3i** was obtained from diethyl 2-phenyl-2-vinylmalonate (26 mg, 0.10 mmol) and 1-bromoethane (33 mg, 0.30 mmol). The crude product was purified by flash column chromatography to afford 12 mg of diethyl 2-(1-phenylbutyl)malonate as colorless oil, (yield = 42 %).

^{1}H NMR (500 MHz, $CDCl_3$) δ 7.29 – 7.25 (m, 2H), 7.21 – 7.17 (m, 3H), 4.24 (q, *J* = 7.1 Hz, 2H), 3.87 (q, 2H), 3.62 (d, *J* = 11.0 Hz, 1H), 3.37 (td, *J* = 10.6, 4.4 Hz, 1H), 1.66 – 1.59 (m, 2H), 1.30 (t, *J* = 7.1 Hz, 3H), 1.13 – 1.06 (m, 2H), 0.93 (t, *J* = 7.1 Hz, 3H), 0.82 (t, *J* = 7.3 Hz, 3H).

^{13}C NMR (125 MHz, $CDCl_3$) δ 168.7, 168.1, 141.2, 128.5, 128.4, 127.0, 61.6, 61.2, 59.1, 45.6, 36.3, 20.4, 14.3, 14.0, 13.8.

HRMS (ESI) *m/z* $[M + Na]^+$ calcd for $C_{17}H_{24}O_4Na$ 315.1572 found 315.1574.

GC chromatogram:

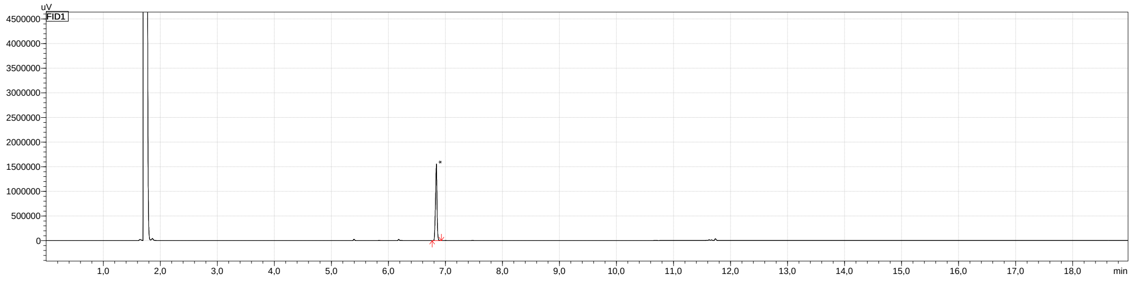

diethyl 2-(3-cyclohexyl-1-phenylpropyl)malonate, 4

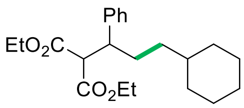

Following the general procedure 5.1 compound **4** was obtained from diethyl 2-phenyl-2-vinylmalonate (26 mg, 0.10 mmol) and (bromomethyl)cyclohexane (53 mg, 0.30 mmol). The crude product was purified by flash column chromatography to afford 13 mg of diethyl 2-(3-cyclohexyl-1-phenylpropyl)malonate as colorless oil, (yield =36 %).

^{1}H NMR (500 MHz, $CDCl_3$) δ 7.28 – 7.24 (m, 2H), 7.22 – 7.15 (m, 3H), 4.24 (q, *J* = 7.1 Hz, 2H), 3.86 (qd, *J* = 7.1, 1.0 Hz, 2H), 3.61 (d, *J* = 10.8 Hz, 1H), 3.31 (td, *J* = 11.0, 3.5 Hz, 1H), 1.74 – 1.68 (m, 1H), 1.64 – 1.54 (m, 6H), 1.29 (t, *J* = 7.1 Hz, 3H), 1.19 – 0.99 (m, 5H), 0.95 – 0.85 (m, 4H), 0.81 – 0.68 (m, 2H).

^{13}C NMR (125 MHz, $CDCl_3$) δ 168.7, 168.1, 141.2, 128.5, 128.4, 126.9, 61.6, 61.2, 59.2, 46.0, 37.5, 34.8, 33.7, 33.0, 31.4, 26.8, 26.5, 26.4, 14.3, 13.8.

HRMS (ESI) *m/z* $[M + Na]^+$ calcd for $C_{22}H_{32}O_4Na$ 383.2198, found 383.2197.

GC chromatogram:

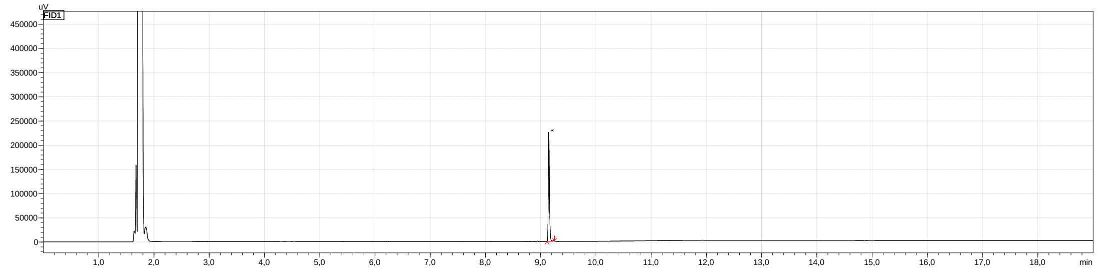

diethyl 2-(4,4-dimethyl-1-phenylpentyl)malonate, 5

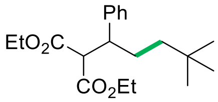

Following the general procedure 5.1 compound **5** was obtained from diethyl 2-phenyl-2-vinylmalonate (26 mg, 0.10 mmol) and 1-bromo-2,2-dimethylpropane (45 mg, 0.30 mmol). The crude product was purified by flash column chromatography to afford 18 mg of diethyl 2-(4,4-dimethyl-1-phenylpentyl)malonate as colorless oli, (yield = 54 %).

^{1}H NMR (500 MHz, $CDCl_3$) δ 7.28 – 7.25 (m, 2H), 7.21 – 7.16 (m, 3H), 4.24 (q, *J* = 7.1 Hz, 2H), 3.87 (q, *J* = 7.1 Hz, 2H), 3.62 (d, *J* = 10.8 Hz, 1H), 3.28 (td, *J* = 10.9, 3.5 Hz, 1H), 1.72 – 1.65 (m, 1H), 1.59 – 1.56 (m, 1H), 1.29 (t, *J* = 7.1 Hz, 3H), 1.08 – 1.01 (m, 1H), 0.95 – 0.88 (m, 4H), 0.77 (s, 9H).

^{13}C NMR (125 MHz, $CDCl_3$) δ 168.7, 168.1, 141.2, 128.5, 128.4, 126.9, 61.6, 61.2, 59.3, 46.5, 41.4, 30.3, 29.3, 29.0, 14.3, 13.9.

HRMS (ESI) *m/z* $[M + Na]^+$ calcd for $C_{20}H_{30}O_4Na$ 357.2042, found 357.2040.

GC chromatogram:

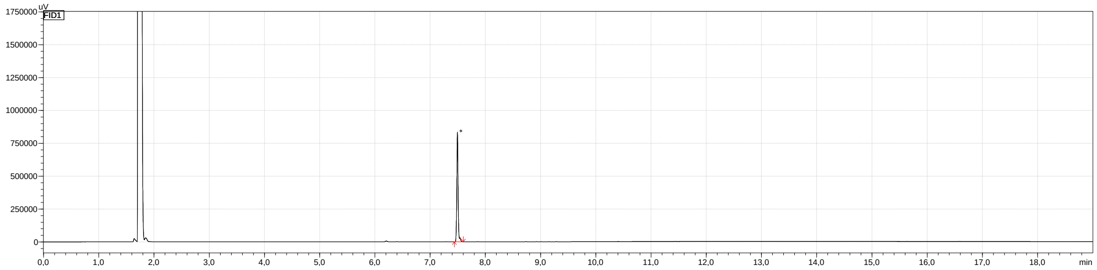

diethyl 2-(3-methyl-1-phenylhexyl)malonate, 6

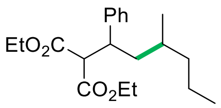

Following the general procedure 5.1 compound **6** was obtained from diethyl 2-phenyl-2-vinylmalonate (26 mg, 0.10 mmol) and 2-bromopentane (45 mg, 0.30 mmol). The crude product was purified by flash column chromatography to afford 5 mg of diethyl 2-(3-methyl-1-phenylhexyl)malonate as colorless oil, (yield = 16 %).

^{1}H NMR (500 MHz, $CDCl_3$) δ 7.27 – 7.26 (m, 2H), 7.22 – 7.16 (m, 3H), 4.23 (qd, *J* = 7.2, 1.9 Hz, 2H), 3.85 (q, *J* = 7.1 Hz, 2H), 3.57 (d, *J* = 10.8 Hz, 1H), 3.47 (td, *J* = 11.0, 3.7 Hz, 1H), 1.59 – 1.54 (m, 2H), 1.47 – 1.43 (m, 1H), 1.31 – 1.26 (m, 4H), 1.17 – 1.09 (m, 2H), 1.06 – 0.99 (m, 1H), 0.92 (t, *J* = 7.1 Hz, 3H), 0.83 (t, *J* = 7.2 Hz, 3H), 0.73 (d, 3H).

^{13}C NMR (125 MHz, $CDCl_3$) δ 168.6, 168.1, 141.3, 128.6, 128.4, 126.9, 61.6, 61.2, 59.5, 43.7, 41.8, 37.3, 29.7, 20.9, 19.6, 14.5, 14.3, 13.9.

HRMS (ESI) *m/z* $[M - H]^-$ calcd for $C_{20}H_{29}O_4$ 333.2066, found 333.2069.

GC chromatogram:

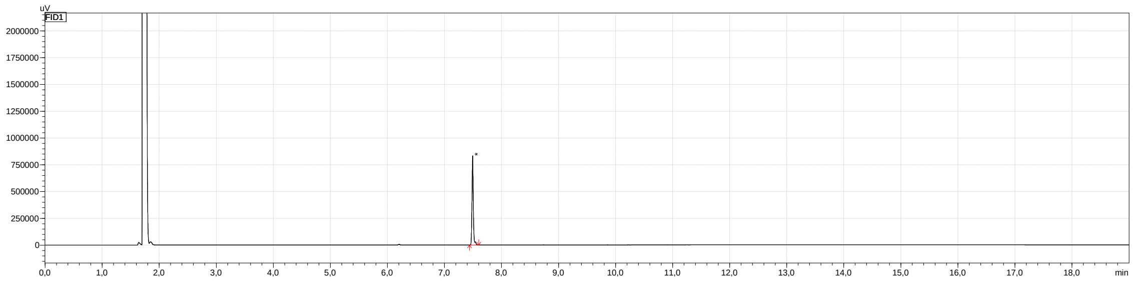

diethyl 2-(2-cyclohexyl-1-phenylethyl)malonate, 7

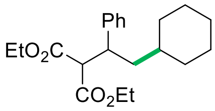

Following the general procedure 5.1 compound **7** was obtained from diethyl 2-phenyl-2-vinylmalonate (26 mg, 0.10 mmol) and bromocyclohexane (49 mg, 0.30 mmol). The crude product was purified by flash column chromatography to afford 12 mg of diethyl 2-(2-cyclohexyl-1-phenylethyl)malonate as colorless oil, (yield = 33 %).

^{1}H NMR (500 MHz, $CDCl_3$) δ 7.28 – 7.26 (m, 2H), 7.25 – 7.16 (m, 3H), 4.24 (qt, *J* = 7.1, 3.5 Hz, 2H), 3.85 (q, *J* = 7.1 Hz, 2H), 3.56 (d, *J* = 10.9 Hz, 1H), 3.49 (td, *J* = 11.1, 3.2 Hz, 1H), 1.92 – 1.87 (m, 1H), 1.67 – 1.56 (m, 3H), 1.47 – 1.41 (m, 2H), 1.34 – 1.21 (m, 4H), 1.10 – 0.99 (m, 3H), 0.95 – 0.81 (m, 6H).

^{13}C NMR (125 MHz, $CDCl_3$) δ 168.6, 168.1, 141.2, 128.5, 128.4, 126.9, 61.6, 61.2, 59.6, 43.0, 41.8, 34.7, 34.5, 31.9, 26.7, 26.3, 26.0, 14.3, 13.9.

HRMS (ESI) *m/z* $[M + Na]^+$ calcd for $C_{21}H_{30}O_4Na$ 369.2042, found 369.2040.

GC chromatogram:

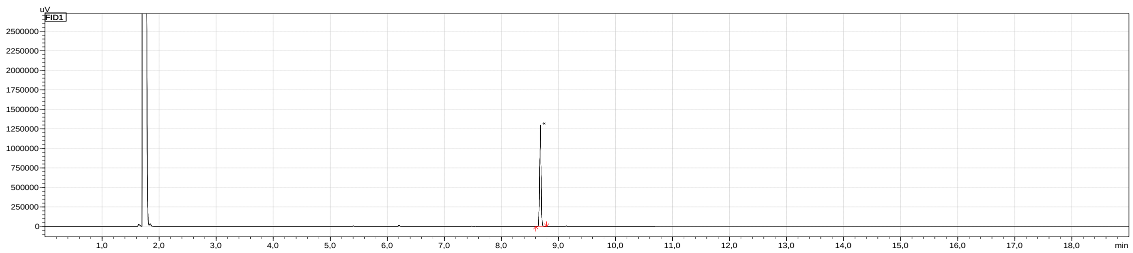

diethyl 2-(1,10-diphenyldecyl)malonate, 9

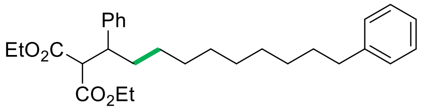

Following the general procedure 5.1 compound **9** was obtained from diethyl 2-phenyl-2-vinylmalonate (26 mg, 0.10 mmol) and 1-bromo-8-phenyloctane (81 mg, 0.30 mmol). The crude product was purified by flash column chromatography to afford 14 mg of diethyl 2-(1,10-diphenyldecyl)malonate as colorless oil, (yield = 50 %).

^{1}H NMR (500 MHz, $CDCl_3$) δ 7.29 – 7.23 (m, 5H), 7.20 – 7.15 (m, 5H), 4.24 (q, *J* = 7.1 Hz, 2H), 3.86 (qd, *J* = 7.1, 1.1 Hz, 2H), 3.61 (d, *J* = 10.9 Hz, 1H), 3.34 (td, *J* = 10.9, 3.7 Hz, 1H), 2.59 – 2.54 (m, 2H), 1.68 – 1.63 (m, 1H), 1.62 – 1.54 (m, 3H), 1.32 – 1.21 (m, 9H), 1.20 – 1.11 (m, 5H), 1.07 – 1.01 (m, 1H), 0.93 (t, *J* = 7.1 Hz, 3H).

^{13}C NMR (125 MHz, $CDCl_3$) δ 168.7, 168.1, 143.1, 141.2, 128.53, 128.50, 128.4, 128.3, 126.9, 125.7, 61.6, 61.2, 59.1, 45.8, 36.1, 34.1, 31.6, 29.57, 29.55, 29.49, 29.45, 29.42, 27.2, 14.3, 13.8.

HRMS (ESI) *m/z* $[M + H]^+$ calcd for $C_{29}H_{41}O_4$ 453.3005, found 453.3004.

GC chromatogram:

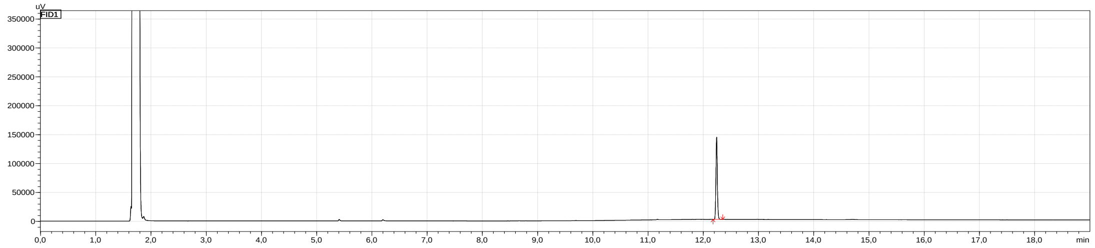

4-dodecyl 1,1-diethyl 2-phenylbutane-1,1,4-tricarboxylate, 10a

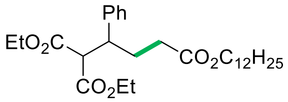

Following the general procedure 5.1 compound **10a** was obtained from diethyl 2-phenyl-2-vinylmalonate (26 mg, 0.10 mmol) and dodecyl 2-bromoacetate (92 mg, 0.30 mmol). The crude product was purified by flash column chromatography to afford 11 mg of 4-dodecyl 1,1-diethyl 2-phenylbutane-1,1,4-tricarboxylate as yellowish oil, (yield = 23 %).

^{1}H NMR (500 MHz, $CDCl_3$) δ 7.30 – 7.26 (m, 2H), 7.25 – 7.16 (m, 3H), 4.25 (q, *J* = 7.1 Hz, 2H), 3.96 (q, 2H), 3.87 (qd, *J* = 7.1, 1.5 Hz, 2H), 3.65 (d, *J* = 10.9 Hz, 1H), 3.36 (td, *J* = 11.1, 2.7 Hz, 1H), 2.16 – 2.02 (m, 3H), 1.94 – 1.87 (m, 1H), 1.61 – 1.50 (m, 3H), 1.31 – 1.23 (m, 20H), 0.93 (t, *J* = 7.1 Hz, 3H), 0.88 (t, *J* = 6.8 Hz, 3H).

^{13}C NMR (125 MHz, $CDCl_3$) δ 173.2, 168.4, 167.7, 139.9, 128.7, 128.5, 127.4, 64.7, 61.8, 61.3, 58.7, 45.2, 32.2, 32.1, 29.79, 29.76, 29.72, 29.65, 29.5, 29.4, 29.3, 28.7, 26.0, 22.8, 14.3, 14.2, 13.8.

HRMS (ESI) *m/z* $[M + Na]^+$ calcd for $C_{29}H_{46}O_6Na$ 513.3192, found 513.3194.

GC chromatogram:

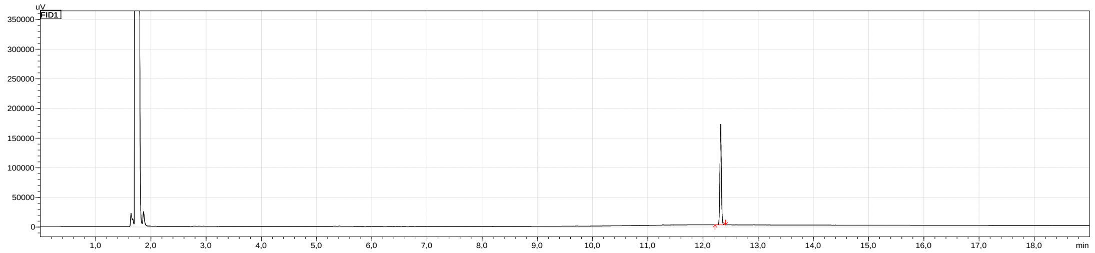

4-dodecyl 1,1-diethyl 1-phenylbutane-1,1,4-tricarboxylate, 10b

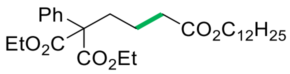

Following the general procedure 5.1 compound **10b** was obtained from diethyl 2-phenyl-2-vinylmalonate (26 mg, 0.10 mmol) and dodecyl 2-bromoacetate (92 mg, 0.30 mmol). The crude product was purified by flash column chromatography to afford 10 mg of 4-dodecyl 1,1-diethyl 1-phenylbutane-1,1,4-tricarboxylate as yellowish oil, (yield = 20 %)

^{1}H NMR (500 MHz, $CDCl_3$) δ 7.45 – 7.38 (m, 2H), 7.37 – 7.30 (m, 2H), 7.30 – 7.26 (m, 1H), 4.27 – 4.18 (m, 4H), 4.03 (t, *J* = 6.8 Hz, 2H), 2.36 – 2.26 (m, 4H), 1.62 – 1.54 (m, 4H), 1.33 – 1.19 (m, 24H), 0.88 (t, *J* = 6.9 Hz, 3H).

^{13}C NMR (125 MHz, $CDCl_3$) δ 173.3, 170.7, 136.9, 128.3, 128.2, 127.6, 64.7, 62.6, 61.7, 35.4, 34.5, 32.1, 29.80, 29.77, 29.73, 29.67, 29.5, 29.4, 28.8, 26.1, 22.8, 20.5, 14.3, 14.1.

HRMS (ESI) *m/z* $[M + Na]^+$ calcd for $C_{29}H_{46}O_6Na$ 513.3192, found 513.3195.

GC chromatogram:

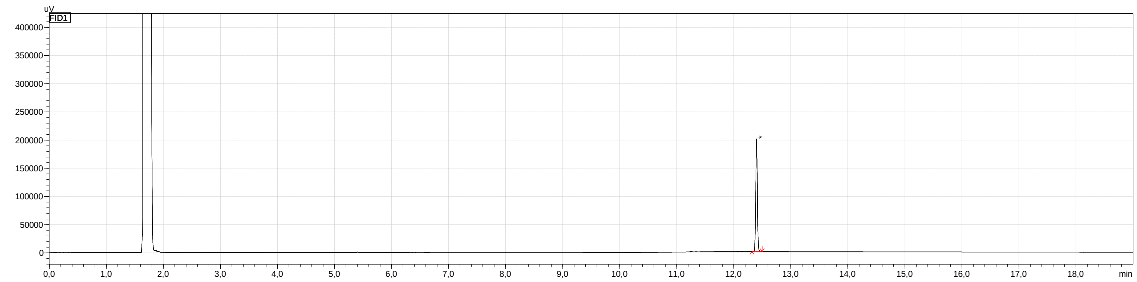

4-dodecyl 1,1-diethyl-1-phenylbut-2-ene-1,1,4-tricarboxylate, 10c

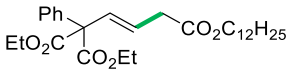

Following the general procedure 5.1 compound **10c** was obtained from diethyl 2-phenyl-2-vinylmalonate (26 mg, 0.10 mmol) and dodecyl 2-bromoacetate (92 mg, 0.30 mmol). The crude product was purified by flash column chromatography to afford 5 mg of 4-dodecyl 1,1-diethyl-1-phenylbut-2-ene-1,1,4-tricarboxylate as yellowish oil, (yield = 11 %)

^{1}H NMR (500 MHz, $CDCl_3$) δ 7.40 – 7.35 (m, 2H), 7.36 – 7.28 (m, 3H), 6.34 (d, 1H), 5.61 (dt, *J* = 16.0, 7.1 Hz, 1H), 4.26 (qd, *J* = 7.1, 2.6 Hz, 4H), 4.06 (t, *J* = 6.8 Hz, 2H), 3.17 (d, *J* = 7.0, 1.5 Hz, 2H), 1.62 – 1.58 (m, 2H), 1.32 – 1.25 (m, 24H), 0.88 (t, *J* = 6.8 Hz, 3H).

^{13}C NMR (125 MHz, $CDCl_3$) 171.3, 169.8, 137.2, 132.0, 128.9, 128.2, 127.8, 126.6, 65.1, 62.1, 38.4, 32.1, 29.80, 29.77, 29.74, 29.65, 29.5, 29.4, 28.7, 26.0, 22.8, 14.3, 14.1.

HRMS (ESI) *m/z* $[M + H]^+$ calcd for $C_{29}H_{45}O_6$ 489.3216, found 489.3223.

GC chromatogram:

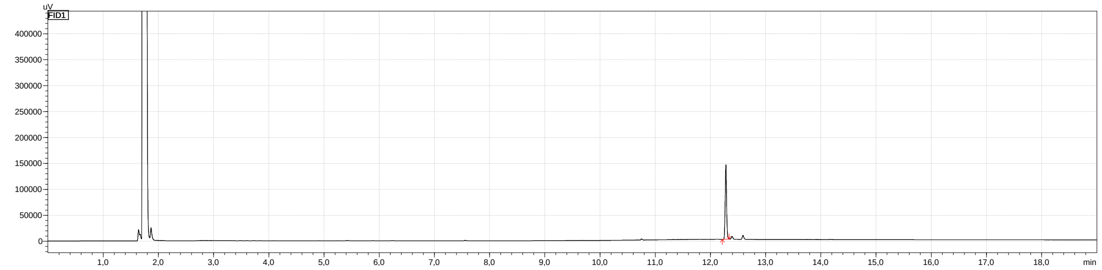

6-dodecyl 1,1-diethyl 2-phenylhexane-1,1,6-tricarboxylate, 11a

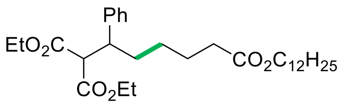

Following the general procedure 5.1 compound **11a** was obtained from diethyl 2-phenyl-2-vinylmalonate (26 mg, 0.10 mmol) and dodecyl 4-bromobutanoate (101 mg, 0.30 mmol). The crude product was purified by flash column chromatography to afford 4 mg of 6-dodecyl 1,1-diethyl 2-phenylhexane-1,1,6-tricarboxylate as colorless oil, (yield = 8 %).

^{1}H NMR (500 MHz, $CDCl_3$) δ 7.28 – 7.25 (m, 2H), 7.23 – 7.15 (m, 3H), 4.24 (q, *J* = 7.2 Hz, 2H), 3.99 (t, *J* = 6.8 Hz, 2H), 3.87 (qd, *J* = 7.1, 1.0 Hz, 2H), 3.61 (d, *J* = 10.9 Hz, 1H), 3.34 (td, *J* = 11.0, 3.6 Hz, 1H), 2.22 – 2.15 (m, 2H), 1.72 – 1.67 (m, 1H), 1.64 – 1.56 (m, 3H), 1.32 – 1.23 (m, 23H), 1.17 – 1.05 (m, 2H), 0.93 (t, *J* = 7.1 Hz, 3H), 0.88 (t, *J* = 6.8 Hz, 3H).

^{13}C NMR (125 MHz, $CDCl_3$) δ 173.8, 168.6, 168.0, 140.8, 128.5, 127.1, 64.6, 61.7, 61.3, 59.0, 45.6, 34.3, 33.7, 32.1, 29.80, 29.78, 29.73, 29.67, 29.5, 29.4, 28.8, 26.7, 26.1, 24.9, 22.8, 14.3, 13.8.

HRMS (ESI) *m/z* $[M + Na]^+$ calcd for $C_{31}H_{50}O_6Na$ 541.3505, found 514.3512.

GC chromatogram:

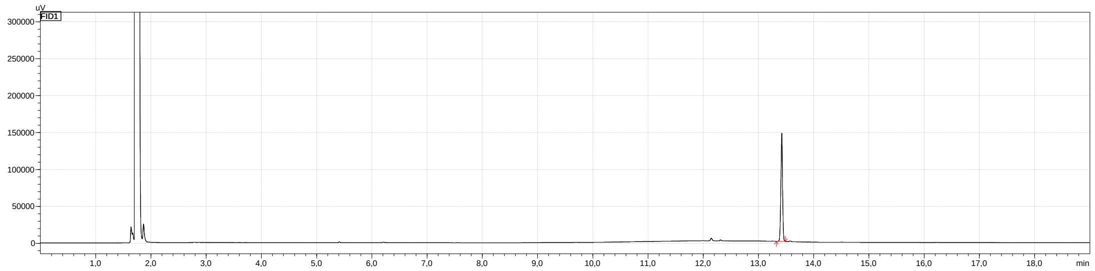

6-dodecyl 1,1-diethyl 1-phenylhexane-1,1,6-tricarboxylate, 11b

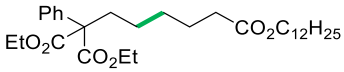

Following the general procedure 5.1 compound **11b** was obtained from diethyl 2-phenyl-2-vinylmalonate (26 mg, 0.10 mmol) and dodecyl 4-bromobutanoate (101 mg, 0.30 mmol). The crude product was purified by flash column chromatography to afford 15 mg of 6-dodecyl 1,1-diethyl 1-phenylhexane-1,1,6-tricarboxylate as colorless oil, (yield = 29 %)

^{1}H NMR (500 MHz, $CDCl_3$) δ 7.43 – 7.38 (m, 2H), 7.35 – 7.30 (m, 2H), 7.29 – 7.26 (m, 1H), 4.26 – 4.17 (m, 4H), 4.03 (t, *J* = 6.8 Hz, 2H), 2.32 – 2.22 (m, 4H), 1.62 – 1.56 (m, 4H), 1.38 – 1.21 (m, 28H), 0.88 (t, *J* = 6.9 Hz, 3H).

^{13}C NMR (125 MHz, $CDCl_3$) δ 173.9, 170.9, 137.3, 128.2, 128.1, 127.5, 64.6, 62.7, 61.6, 35.7, 34.4, 32.1, 29.79, 29.77, 29.73, 29.67, 29.51, 29.49, 29.4, 28.8, 26.1, 24.8, 24.5, 22.8, 14.3, 14.1.

HRMS (ESI) *m/z* $[M + Na]^+$ calcd for $C_{31}H_{50}O_6Na$ 541.3505, found 541.3513.

GC chromatogram:

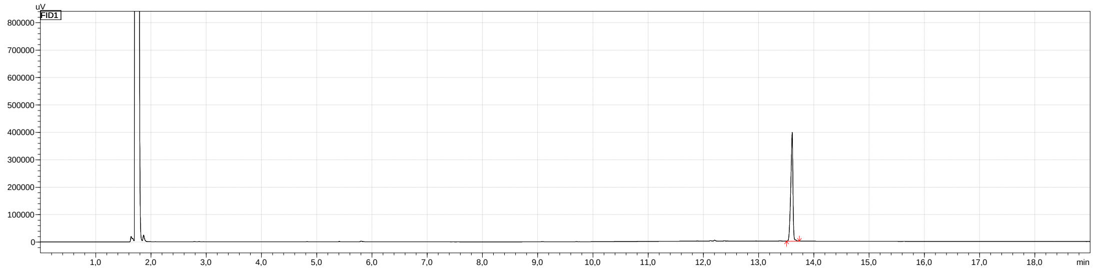

triethyl 2-phenyldecane-1,1,10-tricarboxylate, 13

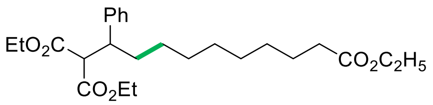

Following the general procedure 5.1 compound **13** was obtained from diethyl 2-phenyl-2-vinylmalonate (26 mg, 0.10 mmol) and ethyl 8-bromooctanoate (75 mg, 0.30 mmol). The crude product was purified by flash column chromatography to afford 22 mg of triethyl 2-phenyldecane-1,1,10-tricarboxylate as colorless oil, (yield = 51 %).

^{1}H NMR (500 MHz, $CDCl_3$) δ 7.28 – 7.25 (m, 2H), 7.22 – 7.15 (m, 3H), 4.24 (q, *J* = 7.1 Hz, 2H), 4.11 (q, *J* = 7.1 Hz, 2H), 3.86 (qd, *J* = 7.1, 1.1 Hz, 2H), 3.61 (d, *J* = 10.9 Hz, 1H), 3.34 (td, *J* = 10.9, 3.7 Hz, 1H), 2.25 (t, *J* = 7.6 Hz, 2H), 1.68 – 1.63 (m, 1H), 1.61 – 1.53 (m, 3H), 1.29 (t, *J* = 7.1 Hz, 3H), 1.27 – 1.01 (m, 13H), 0.93 (t, *J* = 7.1 Hz, 3H).

^{13}C NMR (125 MHz, $CDCl_3$) δ 174.0, 168.7, 168.0, 141.1, 128.5, 128.39, 128.38, 126.9, 61.6, 61.2, 60.3, 59.1, 45.8, 34.5, 34.1, 29.4, 29.30, 29.25, 29.2, 27.1, 25.1, 14.4, 14.3, 13.8.

HRMS (ESI) *m/z* $[M + Na]^+$ calcd for $C_{25}H_{38}O_6Na$ 457.2566, found 457.2568.

GC chromatogram:

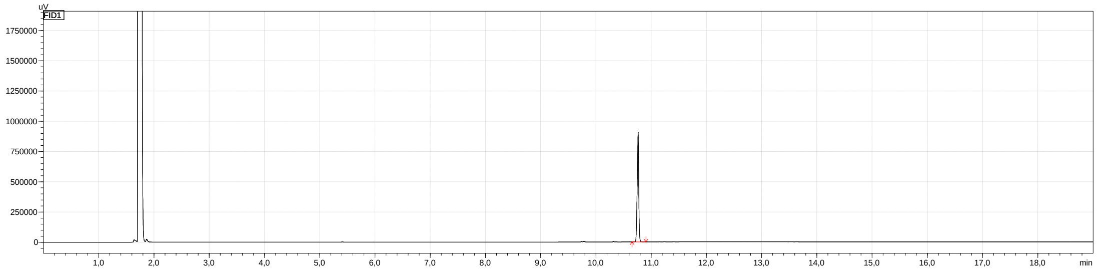

a mixture of diethyl 2-(4-(2-(2-methoxyethoxy)ethoxy)-1-phenylbutyl)malonate, 14 and diethyl 2-(4-(2-(2-methoxyethoxy)ethoxy)butyl)-2-phenylmalonate

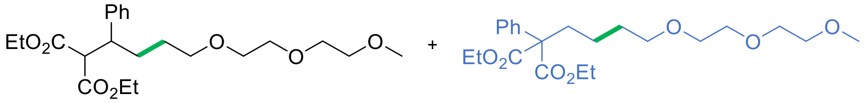

1 : 0.2

Following the general procedure 5.1 compound **14** was obtained from diethyl 2-phenyl-2-vinylmalonate (26 mg, 0.10 mmol) and 1-bromo-2-(2-(2-methoxyethoxy)ethoxy)ethane (68 mg, 0.30 mmol). The crude product was purified by flash column chromatography to afford 9 mg (+ 2 mg of no migration product) of diethyl 2-(4-(2-(2-methoxyethoxy)ethoxy)-1-phenylbutyl)malonate as yellowish oil, (yield = 22 % + 4 % no migration producti).

^{1}H NMR (500 MHz, $CDCl_3$) δ 7.44 – 7.38 (m, 0.4H), 7.35 – 7.29 (m, 0.6H), 7.28 – 7.22 (m, 2H), 7.21 – 7.13 (m, 3H), 4.22 (p, *J* = 6.9 Hz, 2H + 0.8H), 3.86 (qd, *J* = 7.1, 1.1 Hz, 2H), 3.68 – 3.56 (m, 5H + 0.4H), 3.55 – 3.51 (m, 2H + 0.6H), 3.51 – 3.47 (m, 2H), 3.43-3.39 (t, *J* = 6.8 Hz, 0.4H), 3.36 (s, 3H + 0.6H), 3.36 – 3.32 (m, 3H + 0.6H), 2.32 – 2.25 (m, 0.4H), 1.80 – 1.70 (m, 1H + 0.2H), 1.67 – 1.61 (m, 1H + 0.2H), 1.45 – 1.32 (m, 2H + 0.4H), 1.29 (t, *J* = 7.1 Hz, 3H), 1.23 (t, *J* = 7.1 Hz, 1.2H), 0.92 (t, *J* = 7.1 Hz, 3H).

^{13}C NMR (125 MHz, $CDCl_3$) δ 170.7, 168.4, 167.8, 140.6, 128.4, 128.3, 128.1, 128.0, 127.4, 126.9, 71.9, 71.0, 70.9, 70.60, 70.59, 70.53, 70.51, 70.1, 70.0, 62.6, 61.5, 61.4, 61.1, 59.0, 58.9, 45.5, 30.5, 29.9, 27.2, 14.1, 14.0, 13.7.

HRMS (ESI) *m/z* $[M + Na]^+$ calcd for $C_{22}H_{34}O_7Na$ 433.2202, found 433.2206.

GC chromatogram:

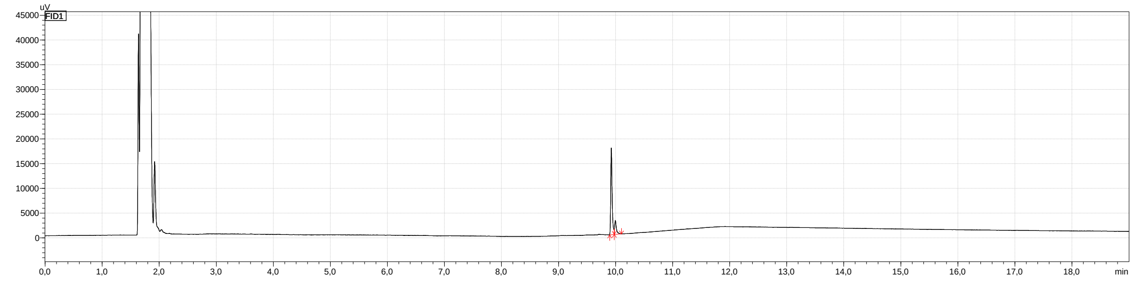

diethyl 2-(4-hydroxy-1-phenyldecyl)malonate, 16a

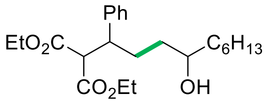

Following the general procedure 5.1 compound **16a** was obtained from diethyl 2-phenyl-2-vinylmalonate (26 mg, 0.10 mmol) and 1-bromooctan-2-ol (63 mg, 0.30 mmol). The crude product was purified by flash column chromatography to afford 9 mg of diethyl 2-(4-hydroxy-1-phenyldecyl)malonate as colorless oil, (yield = 23 %).

^{1}H NMR (500 MHz, $CDCl_3$) δ 7.30 – 7.26 (m, 2H), 7.25 – 7.15 (m, 3H), 4.24 (q, *J* = 7.1 Hz, 2H), 3.86 (q, *J* = 7.1 Hz, 2H), 3.62 (d, *J* = 9.8 Hz, 2H), 3.36 (td, *J* = 10.8, 3.6 Hz, 1H), 1.86 – 1.72 (m, 2H), 1.38 – 1.11 (m, 16H), 0.92 (t, *J* = 7.1 Hz, 3H), 0.87 (t, *J* = 6.9 Hz, 3H).

^{13}C NMR (125 MHz, $CDCl_3$) δ 168.9, 167.9, 140.7, 128.6, 128.5, 127.2, 71.0, 61.8, 61.3, 58.9, 45.1, 37.7, 34.6, 31.9, 30.1, 29.4, 25.8, 22.7, 14.3, 14.2, 13.8.

HRMS (ESI) *m/z* $[M + Na]^+$ calcd for $C_{23}H_{36}O_5Na$ 415.2460, found 415.2463.

GC chromatogram:

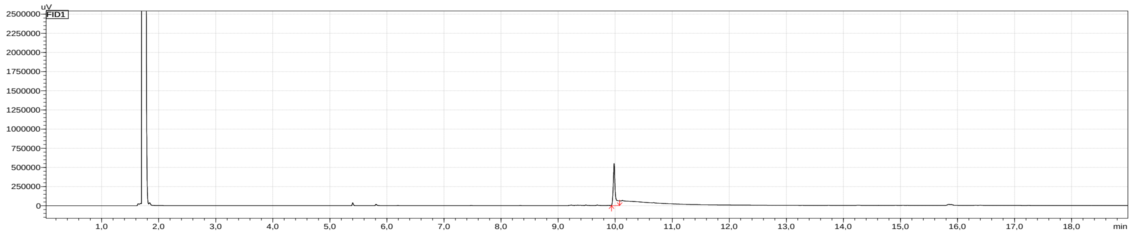

diethyl 2-(4-oxo-1-phenyldecyl)malonate, 16b

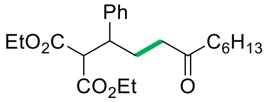

Following the general procedure 5.1 compound **16b** was obtained from diethyl 2-phenyl-2-vinylmalonate (26 mg, 0.10 mmol) and 1-bromooctan-2-ol (63 mg, 0.30 mmol). The crude product was purified by flash column chromatography to afford 4 mg of diethyl 2-(4-oxo-1-phenyldecyl)malonate as colorless oil, (yield = 11 %).

^{1}H NMR (500 MHz, $CDCl_3$) δ 7.30 – 7.26 (m, 2H), 7.24 – 7.15 (m, 3H), 4.24 (qd, *J* = 7.1, 0.9 Hz, 2H), 3.86 (qd, *J* = 7.1, 1.5 Hz, 2H), 3.63 (d, 1H), 3.32 (td, *J* = 11.3, 3.3 Hz, 1H), 2.31 – 2.20 (m, 3H), 2.12 – 2.00 (m, 2H), 1.88 – 1.81 (m, 1H), 1.49 – 1.40 (m, 2H), 1.30 (t, *J* = 7.1 Hz, 3H), 1.26 – 1.17 (m, 6H), 0.92 (t, *J* = 7.1 Hz, 3H), 0.85 (t, *J* = 7.1 Hz, 3H).

^{13}C NMR (125 MHz, $CDCl_3$) δ 210.6, 168.4, 167.8, 140.2, 128.6, 128.5, 127.4, 61.8, 61.3, 58.9, 45.2, 42.9, 40.5, 31.7, 29.0, 27.9, 23.9, 22.6, 14.3, 14.2, 13.8.

HRMS (ESI) *m/z* $[M + Na]^+$ calcd for $C_{23}H_{34}O_5Na$ 413.2304, found 413.2311.

GC chromatogram:

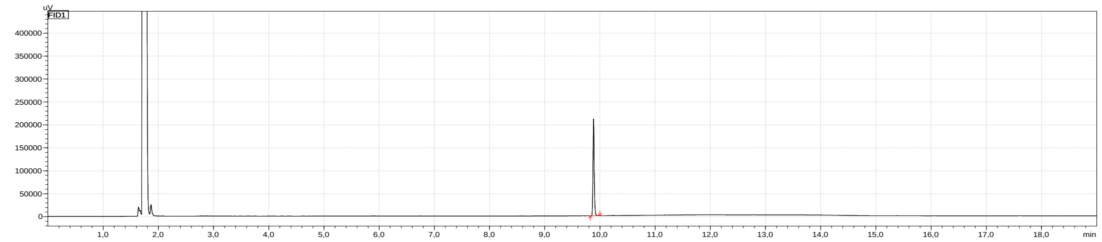

diethyl 2-(4-hydroxydecyl)-2-phenylmalonate, 16c

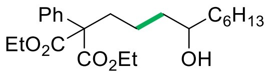

Following the general procedure 5.1 compound **16c** was obtained from diethyl 2-phenyl-2-vinylmalonate (26 mg, 0.10 mmol) and 1-bromooctan-2-ol (63 mg, 0.30 mmol). The crude product was purified by flash column chromatography to afford 6 mg of diethyl 2-(4-hydroxydecyl)-2-phenylmalonate as colorless oil, (yield = 14 %).

^{1}H NMR (500 MHz, $CDCl_3$) δ 7.44 – 7.37 (m, 2H), 7.36 – 7.30 (m, 2H), 7.30 – 7.26 (m, 1H), 4.22 (dqd, *J* = 10.6, 7.0, 3.3 Hz, 4H), 3.61 – 3.55 (m, 1H), 2.36 – 2.24 (m, 2H), 1.47 – 1.36 (m, 5H), 1.33 – 1.20 (m, 15H), 0.88 (t, 3H).

^{13}C NMR (125 MHz, $CDCl_3$) δ 170.9, 137.3, 128.3, 128.1, 127.6, 71.5, 62.8, 61.66, 61.65, 37.58, 37.57, 35.7, 32.0, 29.5, 25.8, 22.8, 21.0, 14.2, 14.14, 14.13.

HRMS (ESI) *m/z* $[M + Na]^+$ calcd for $C_{23}H_{36}O_5Na$ 415.2460, found 415.2458.

GC chromatogram:

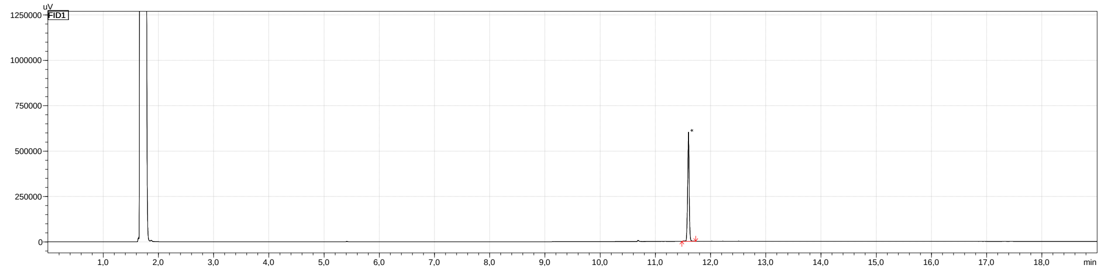

diethyl 2-(7-hydroxy-1-phenyldodecyl)malonate, 17

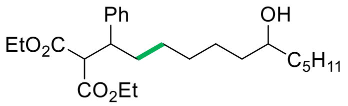

Following the general procedure 5.1 compound **17** was obtained from diethyl 2-phenyl-2-vinylmalonate (26 mg, 0.10 mmol) and 1-bromodecan-5-ol (71 mg, 0.30 mmol). The crude product was purified by flash column chromatography to afford 16 mg of diethyl 2-(7-hydroxy-1-phenyldodecyl)malonate as colorless oil, (yield = 38 %).

^{1}H NMR (500 MHz, $CDCl_3$) δ 7.28 – 7.25 (m, 2H), 7.22 – 7.16 (m, 3H), 4.24 (q, *J* = 7.1 Hz, 2H), 3.86 (q, *J* = 7.1 Hz, 2H), 3.61 (d, *J* = 10.9 Hz, 1H), 3.51 (s, 1H), 3.34 (td, *J* = 10.8, 3.7 Hz, 1H), 1.71 – 1.59 (m, 2H), 1.40 – 1.18 (m, 19H), 0.93 (t, *J* = 7.1 Hz, 3H), 0.88 (t, *J* = 6.9 Hz, 3H).

^{13}C NMR (125 MHz, $CDCl_3$) δ 168.7, 168.0, 141.1, 128.5, 128.4, 127.0, 72.1, 61.6, 61.2, 59.1, 45.8, 37.6, 37.5, 34.0, 32.0, 29.5, 29.4, 27.1, 25.5, 22.8, 14.3, 14.2, 13.8.

HRMS (ESI) *m/z* [M + Na]+ calcd for $C_{25}H_{40}O_5Na$ 443.2773, found 443.2778.

GC chromatogram:

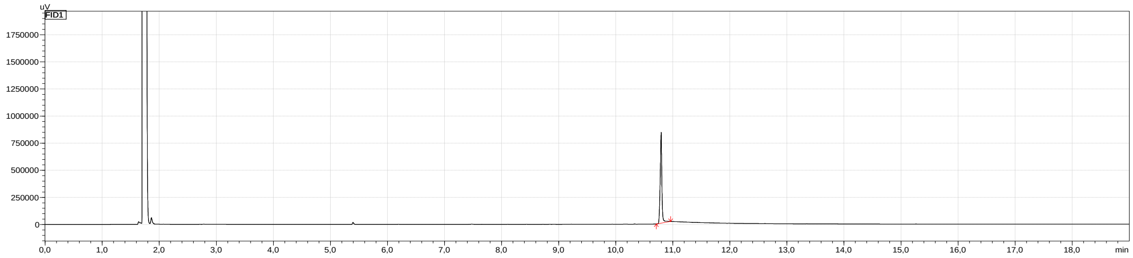

diethyl 2-(10-hydroxy-1-phenyldecyl)malonate, 18

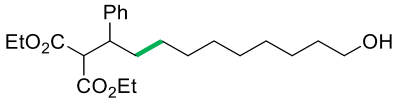

Following the general procedure 5.1 compound **18** was obtained from diethyl 2-phenyl-2-vinylmalonate 26 mg, 0.10 mmol) and 8-bromooctan-1-ol (63 mg, 0.30 mmol). The crude product was purified by flash column chromatography to afford 12 mg of diethyl 2-(10-hydroxy-1-phenyldecyl)malonate as colorless oil, (yield = 31 %).

^{1}H NMR (500 MHz, $CDCl_3$) δ 7.28 – 7.25 (m, 2H), 7.21 – 7.16 (m, 3H), 4.24 (q, *J* = 7.1 Hz, 2H), 3.86 (q, *J* = 7.2, 0.7 Hz, 2H), 3.64 – 3.58 (m, 3H), 3.34 (td, *J* = 10.9, 3.9 Hz, 1H), 1.70 – 1.58 (m, 2H), 1.57 – 1.51 (m, 2H), 1.33 – 1.12 (m, 15H), 0.93 (t, *J* = 7.1 Hz, 3H).

^{13}C NMR (125 MHz, $CDCl_3$) δ 168.7, 168.0, 141.1, 128.5, 128.4, 126.9, 63.2, 61.6, 61.2, 59.1, 45.8, 34.1, 32.9, 29.6, 29.4, 29.39, 29.37, 27.1, 25.8, 14.3, 13.8.

HRMS (ESI) *m/z* [M + H]+ calcd for $C_{23}H_{37}O_5$ 393.2641, found 393.2639.

GC chromatogram:

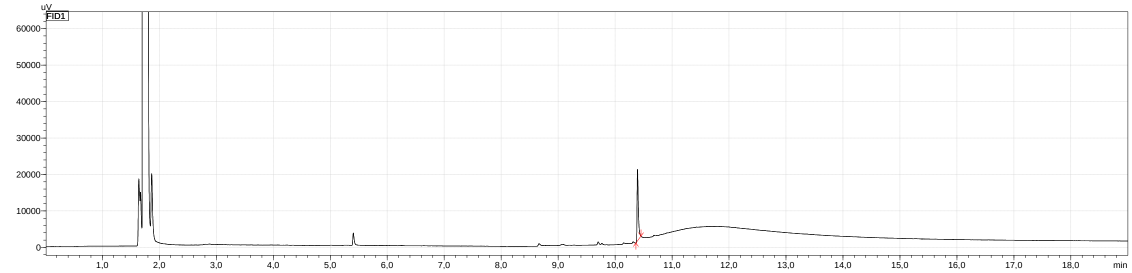

diethyl 2-(1-(4-cyanophenyl)tetradecyl)malonate, 19a

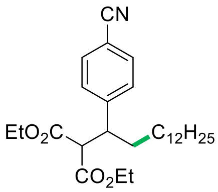

Following the general procedure 5.1 compound **19a** was obtained from diethyl 2-(4-cyanophenyl)-2-vinylmalonate (29 mg, 0.10 mmol) and 1-bromododecane (75 mg, 0.30 mmol). The crude product was purified by flash column chromatography to afford 26 mg of diethyl 2-(1-(4-cyanophenyl)tetradecyl)malonate as colorless oil, (yield = 56 %).

^{1}H NMR (500 MHz, $CDCl_3$) δ 7.58 (d, *J* = 8.1 Hz, 2H), 7.32 (d, *J* = 8.1 Hz, 2H), 4.24 (q, *J* = 7.1 Hz, 2H), 3.94 – 3.86 (m, 2H), 3.60 (d, *J* = 10.7 Hz, 1H), 3.42 (td, *J* = 10.9, 3.6 Hz, 1H), 1.74 – 1.66 (m, 1H), 1.60 – 1.53 (m, 1H), 1.30 – 1.08 (m, 25H), 0.97 (t, *J* = 7.1 Hz, 3H), 0.87 (t, *J* = 6.9 Hz, 3H).

^{13}C NMR (125 MHz, $CDCl_3$) δ 168.1, 167.6, 147.2, 132.3, 129.4, 118.9, 111.0, 61.9, 61.5, 58.4, 45.7, 33.8, 32.0, 29.78, 29.75, 29.74, 29.7, 29.6, 29.5, 29.43, 29.35, 27.1, 22.8, 14.2, 13.9.

HRMS (ESI) *m/z* $[M + H]^+$ calcd for $C_{28}H_{44}NO_4$ 458.3270 found 458.3276.

GC chromatogram:

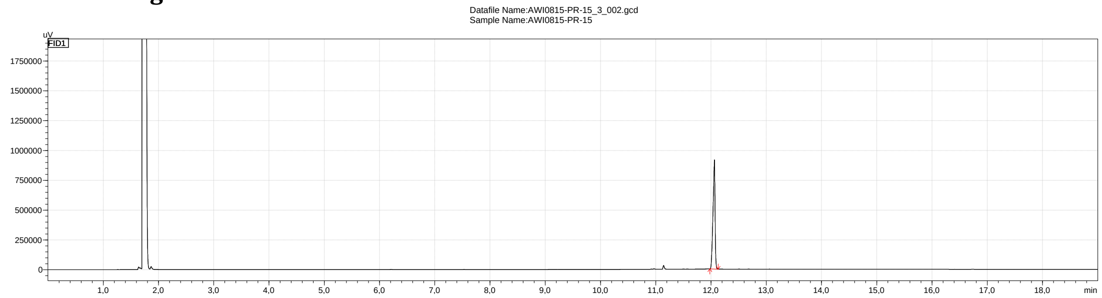

diethyl 2-(1-(4-(trifluoromethyl)phenyl)tetradecyl)malonate, 19b

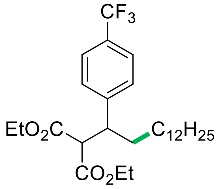

Following the general procedure 5.1 compound **19b** was obtained from diethyl 2-(4-(trifluoromethyl)phenyl)-2-vinylmalonate (33 mg, 0.10 mmol) and 1-bromododecane (75 mg, 0.30 mmol). The crude product was purified by flash column chromatography to afford 36 mg of diethyl 2-(1-(4-(trifluoromethyl)phenyl)tetradecyl)malonate as colorless oil, (yield = 71 %).

^{1}H NMR (500 MHz, $CDCl_3$) δ 7.54 (d, *J* = 8.1 Hz, 2H), 7.32 (d, *J* = 8.0 Hz, 2H), 4.24 (q, *J* = 7.1 Hz, 2H), 3.88 (q, *J* = 7.1 Hz, 2H), 3.62 (d, *J* = 10.8 Hz, 1H), 3.43 (td, *J* = 10.9, 3.6 Hz, 1H), 1.70 (tdd, *J* = 9.8, 6.1, 3.1 Hz, 1H), 1.65 – 1.58 (m, 1H), 1.31 – 1.13 (m, 23H), 1.07 (dd, *J* = 9.0, 4.9 Hz, 1H), 1.00 (td, *J* = 9.1, 8.4, 4.2 Hz, 1H), 0.93 (t, *J* = 7.1 Hz, 3H), 0.87 (t, *J* = 6.9 Hz, 3H).

^{13}C NMR (125 MHz, $CDCl_3$) δ 168.3, 167.7, 145.6, 129.3 (q_{CF3}, *J* = 32.4 Hz), 125.4 (q_{CF3}, *J* = 3.9, 3.4 Hz), 124.3 (q_{CF3}, *J* = 271.9 Hz), 123.2, 61.8, 61.4, 58.7, 45.5, 34.0, 32.1, 29.80, 29.77, 29.71, 29.65, 29.48, 29.47, 29.4, 27.1, 22.8, 14.2, 13.8.

^{19}F NMR (470 MHz, $CDCl_3$) δ -62.5 (s, 3F).

HRMS (ESI) *m/z* $[M + H]^+$ calcd for $C_{28}H_{44}O_4F_3$ 501.3192, found 501.3195.

GC chromatogram:

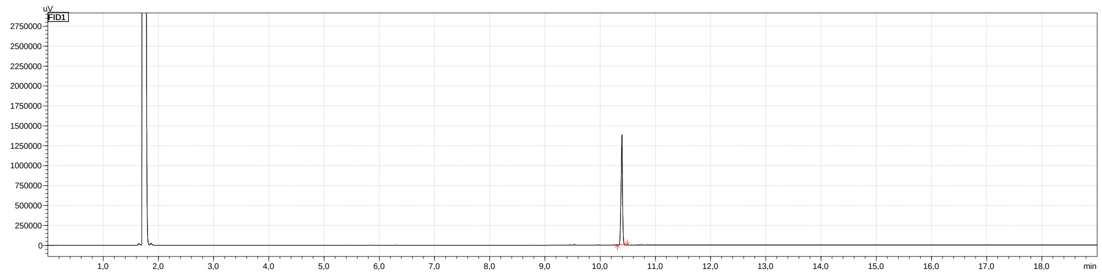

diethyl 2-(1-(4-methoxyphenyl)tetradecyl)malonate, 20a

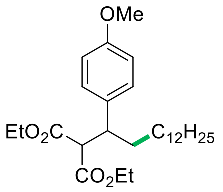

Following the general procedure 5.1 compound **20a** was obtained from diethyl 2-(4-methoxyphenyl)-2-vinylmalonate (29 mg, 0.10 mmol) and 1-bromododecane (75 mg, 0.30 mmol). The crude product was purified by flash column chromatography to afford 22 mg of diethyl 2-(1-(4-methoxyphenyl)tetradecyl)malonate, (yield = 47 %).

^{1}H NMR (500 MHz, $CDCl_3$) δ 7.13 – 7.08 (m, 2H), 6.85 – 6.77 (m, 2H), 4.23 (q, *J* = 7.1 Hz, 2H), 3.89 (qd, *J* = 7.1, 1.4 Hz, 2H), 3.78 (s, 3H), 3.56 (d, *J* = 10.8 Hz, 1H), 3.29 (td, *J* = 11.0, 3.6 Hz, 1H), 1.70 – 1.54 (m, 2H), 1.29 – 1.05 (m, 25H), 0.97 (t, *J* = 7.1 Hz, 3H), 0.88 (t, *J* = 6.9 Hz, 3H).

^{13}C NMR (125 MHz, $CDCl_3$) δ 168.8, 168.1, 158.5, 133.2, 129.4, 113.8, 61.6, 61.2, 59.3, 55.3, 45.0, 34.2, 32.1, 29.82, 29.79, 29.76, 29.7, 29.6, 29.5, 27.2, 22.8, 14.28, 14.25, 13.9.

HRMS (ESI) *m/z* $[M + Na]^+$ calcd for $C_{28}H_{46}O_5Na$ 485.3243, found 485.3235.

GC chromatogram:

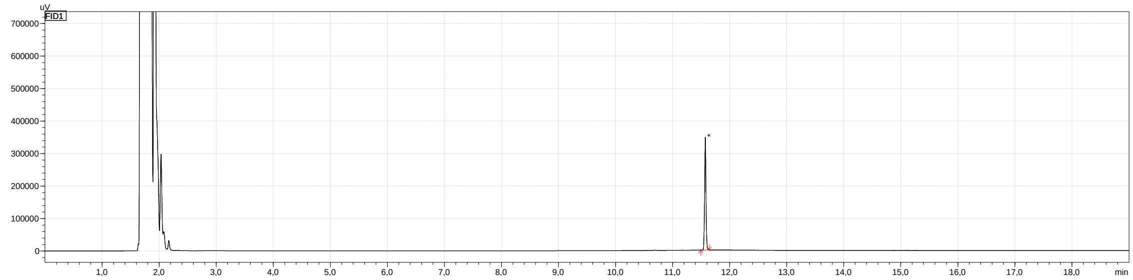

diethyl 2-(4-methoxyphenyl)-2-tetradecylmalonate, 20b

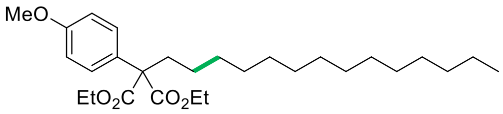

Following the general procedure 5.1 compound **20b** was obtained from diethyl 2-phenyl-2-vinylmalonate (29 mg, 0.10 mmol) and 1-bromododecane (75 mg, 0.30 mmol). The crude product was purified by flash column chromatography to afford 11 mg of diethyl 2-(4-methoxyphenyl)-2-tetradecylmalonate as colorless oil, (yield = 24 %).

^{1}H NMR (500 MHz, $CDCl_3$) δ 7.37 – 7.34 (m, 2H), 6.87 – 6.85 (m, 2H), 4.22 – 4.18 (m, 4H), 3.80 (s, 3H), 2.28 – 2.24 (m, 2H), 1.26 – 1.22 (m, 27H), 0.88 (t, *J* = 6.9 Hz, 6H).

^{13}C NMR (125 MHz, $CDCl_3$) δ 171.2, 158.8, 129.5, 129.2, 113.6, 62.0, 61.5, 55.4, 35.6, 32.1, 30.5, 30.1, 29.84, 29.81, 29.77, 29.7, 29.51, 29.47, 24.7, 22.8, 14.3, 14.2.

HRMS (ESI) *m/z* $[M + Na]^+$ calcd for $C_{28}H_{46}O_5Na$ 485.3243, found 485.3241.

GC chromatogram:

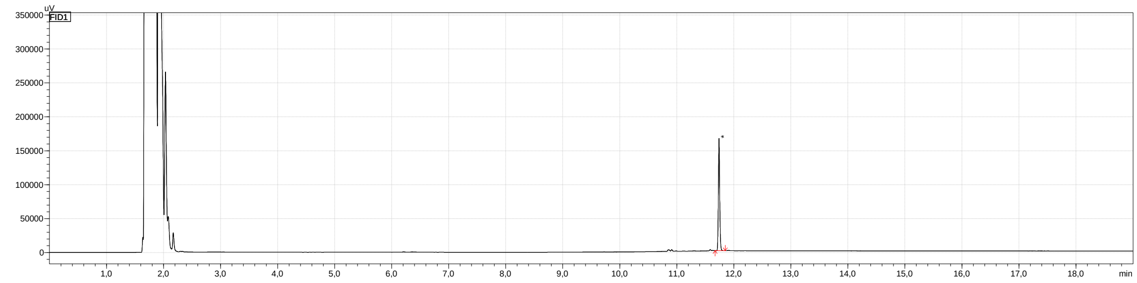

diethyl 2-(1-(3-methoxyphenyl)tetradecyl)malonate, 21

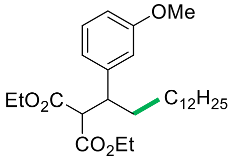

Following the general procedure 5.1 compound **21** was obtained from diethyl 2-(3-methoxyphenyl)-2-vinylmalonate (29 mg, 0.10 mmol) and 1-bromododecane (75 mg, 0.30 mmol). The crude product was purified by flash column chromatography to afford 29 mg of diethyl 2-(1-(3-methoxyphenyl)tetradecyl)malonate as yellowish oil, (yield = 62 %).

^{1}H NMR (600 MHz, $CDCl_3$) δ 7.20 – 7.16 (m, 1H), 6.80 – 6.77 (m, 1H), 6.75 – 6.71 (m, 2H), 4.23 (q, *J* = 7.1 Hz, 2H), 3.89 (q, *J* = 7.1 Hz, 2H), 3.78 (s, 3H), 3.60 (d, *J* = 10.9 Hz, 1H), 3.32 (td, *J* = 10.9, 3.7 Hz, 1H), 1.65 – 1.57 (m, 2H), 1.30 – 1.04 (m, 25H), 0.96 (t, *J* = 7.1 Hz, 3H), 0.87 (t, *J* = 7.0 Hz, 3H).

^{13}C NMR (126 MHz, $CDCl_3$) δ 168.7, 168.0, 159.6, 142.9, 129.3, 120.9, 114.3, 112.2, 61.6, 61.2, 59.0, 55.3, 45.8, 34.1, 32.1, 29.81, 29.78, 29.74, 29.70, 29.53, 29.51, 29.49, 27.2, 22.8, 14.3, 13.9.

HRMS (ESI) *m/z* $[M + Na]^+$ calcd for $C_{28}H_{46}O_5Na$ 485.3243, found 485.3246.

GC chromatogram:

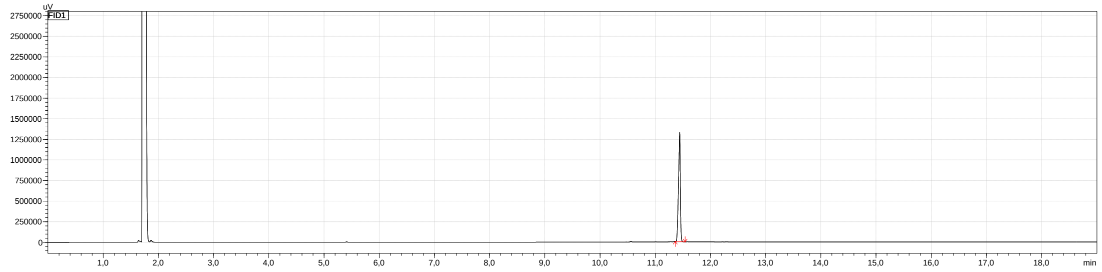

7. Mechanistic consideration

7.1. Proposed mechanism

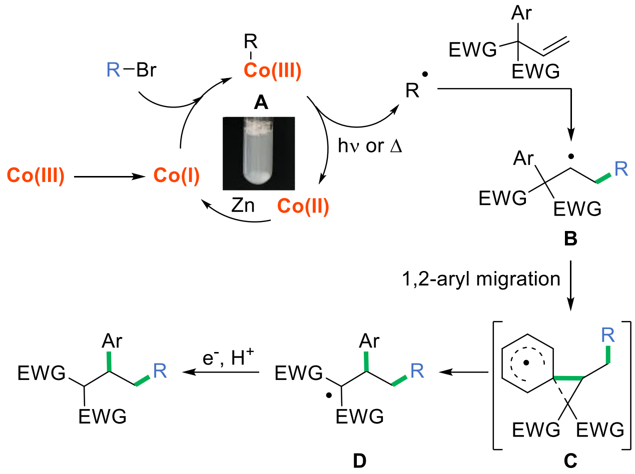

7.2. Co(III)-alkyl complex formation

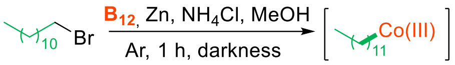

<u>Reaction conditions:</u> 1-bromododecane (0.10 mmol, 1 equiv.,), vitamin B_{12} (0.10 mmol, 1 equiv.), Zn (3.00 mmol, 30 equiv.), NH_4Cl (3.00 mmol, 30 equiv.), MeOH (2 mL), darkness, 1h.

A glass reaction tube equipped with a magnetic bar was charged with vitamin B_{12} (136 mg, 0.10 mmol, 1 equiv.) ammonium chloride (80 mg, 3.00 mmol, 30 equiv.) and activated zinc (196 mg, 3.00 mmol, 30.0 equiv.), then MeOH (2 mL) was added. Tube was sealed with a septum and the resulting mixture was degassed by purging the solution with argon for 20 min with simultaneous sonication in ultrasonic bath (the solution turned from red to dark green or brown). Subsequently, the reaction tube was then sealed with aluminium foil, bromide (25 mg, 0.10 mmol, 1.0 equiv.) was added and the reaction was placed on a magnetic stirrer. After 60 minutes an aliquot was taken from the reaction mixture and its composition was studied by HRMS ESI(+).

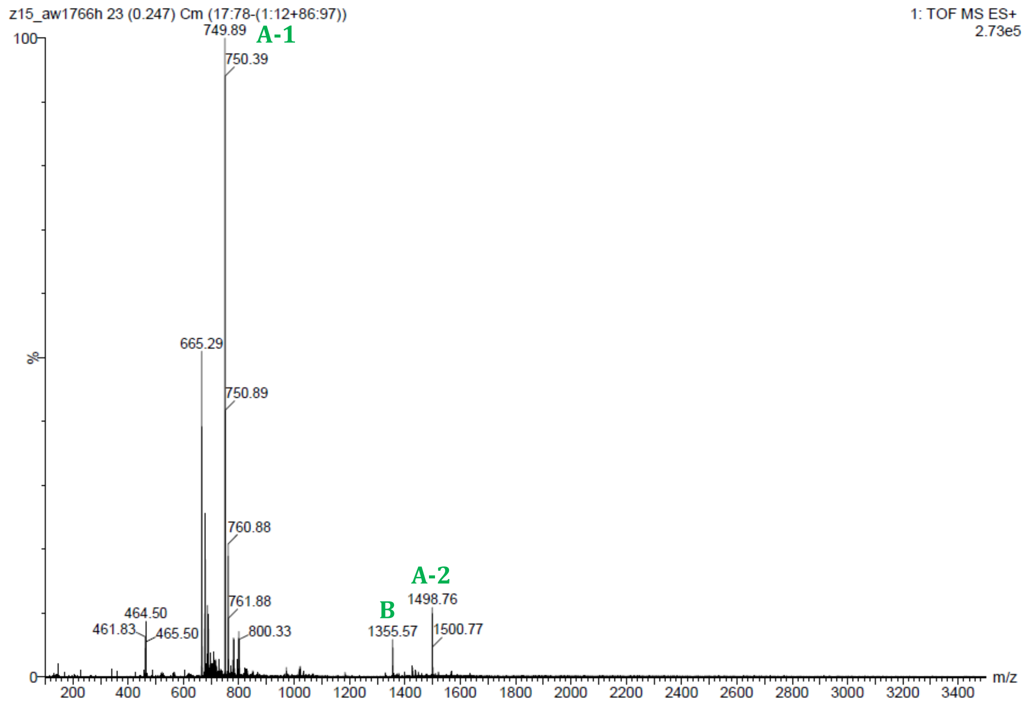

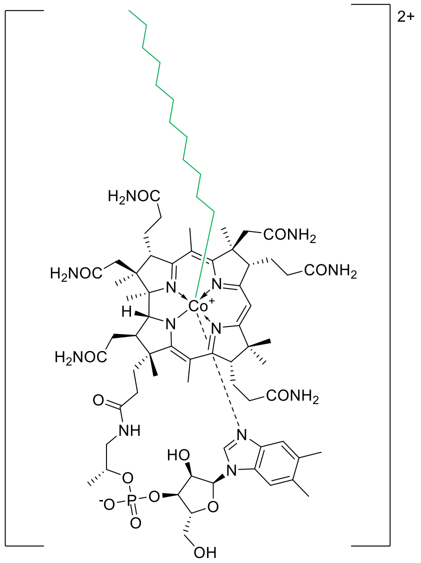

Signal A-1
$C_{74}H_{113}CoN_{13}O_{14}P^{2+}$
Calcd mass = 749,88
Found = 749,89

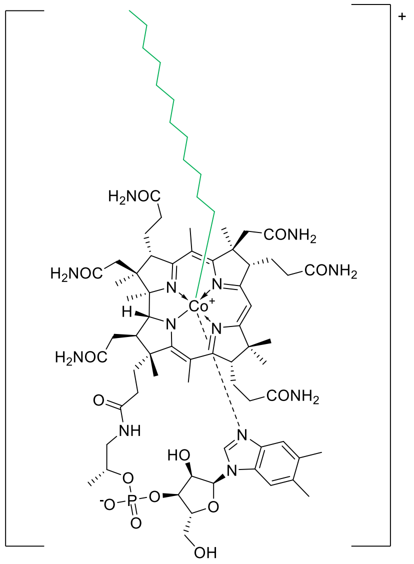

Signal A-2
$C_{74}H_{113}CoN_{13}O_{14}P^{+}$
Calcd mass = 1498,76
Found = 1498,76

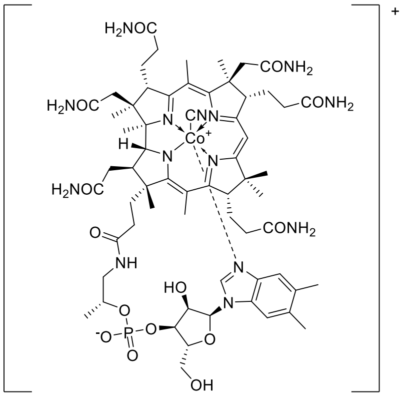

Signal B
$C_{63}H_{88}CoN_{14}O_{14}P^{+}$
Calcd mass = 1355,57
Found = 1355,57

The HRMS ESI (+) spectrum of the reaction mixture indicates the presence of three main forms of the catalyst (signals A-1-2 and B). Signals A-1-2 corresponds to the mass of alkylcobalamin complexes. Signal B corresponds to the mass of the catalyst.

This experiment proves the hypothesis that the reaction involves alkyl-cobalt complex.

7.3. Experiment with a radical trap

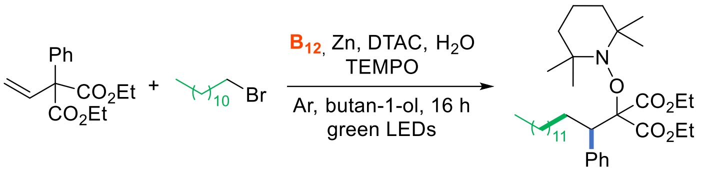

Reaction conditions: diethyl 2-phenyl-2-vinylmalonate (0.10 mmol), 1-bromododecane (3 equiv., 0.30 mmol), TEMPO (3 equiv., 0.30 mmol) vitamin B_{12} (2.5 mol%, 0.003 mmol), Zn (3 equiv., 0.30 mmol), DTAC (3.5 equiv., 0.35 mmol), *n*-BuOH (12.5 equiv., 1.25 mmol), H_2O (5 mL), green LEDs (525 nm), 16 h, 40 °C.

The reaction was set up following the general procedure A (in 4 mL of H_2O). Subsequently (after 2 h), TEMPO (3 equiv., 0.30 mmol in 1 mL of DTAC solution in H_2O) was added. Then the reaction was worked up as usual. HRMS ESI(+) analysis of the crude reaction mixture indicates the formation of the TEMPO adduct.

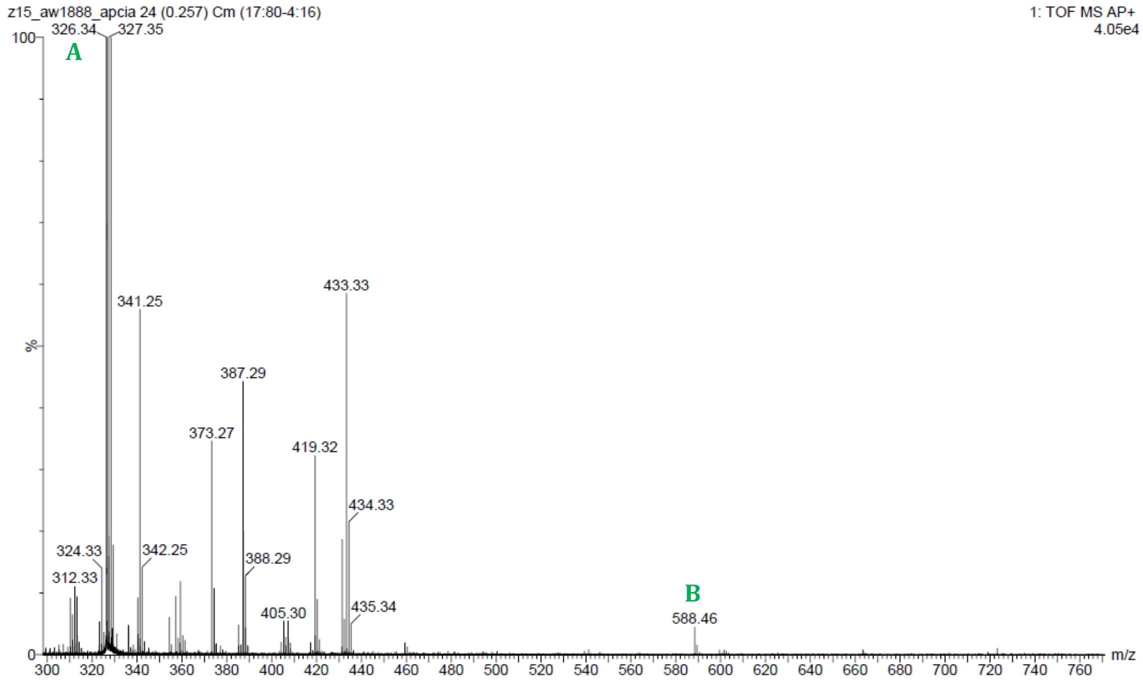

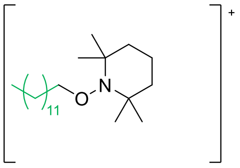

Signal A
Chemical Formula: $C_{22}H_{46}NO_5^+$
Exact Mass: 326.33
Found: 326.34

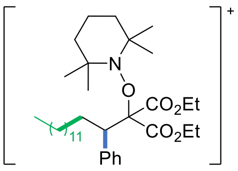

Signal B
Chemical Formula: $C_{36}H_{62}NO_5^+$
Exact Mass: 588.45
Found: 588.46

The HRMS ESI (+) spectrum of the reaction mixture indicates the presence of two main forms of the complex (signals A and B). Signals A corresponds to the mass of dodecane-TEMPO adduct. Signal B corresponds to the mass of the product-TEMPO adduct.

This experiment proves the hypothesis that reaction involves radical as an intermediate.

7.4. Deuterium labeling experiment

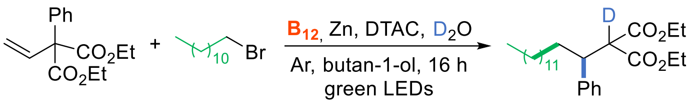

Reaction conditions: diethyl 2-phenyl-2-vinylmalonate (0.10 mmol), 1-bromododecane (3 equiv., 0.30 mmol), vitamin B_{12} (2.5 mol%, 0.003 mmol), Zn (3 equiv, 0.30 mmol), DTAC (3.5 equiv., 0.35 mmol), *n*-BuOH (12.5 equiv., 1.25 mmol), D_2O (5 mL), green LEDs (525 nm), 16 h, 40 °C.

The reaction was set up following the general procedure 5.1. An aliquot was taken and its composition was studied by ^{1}H NMR.

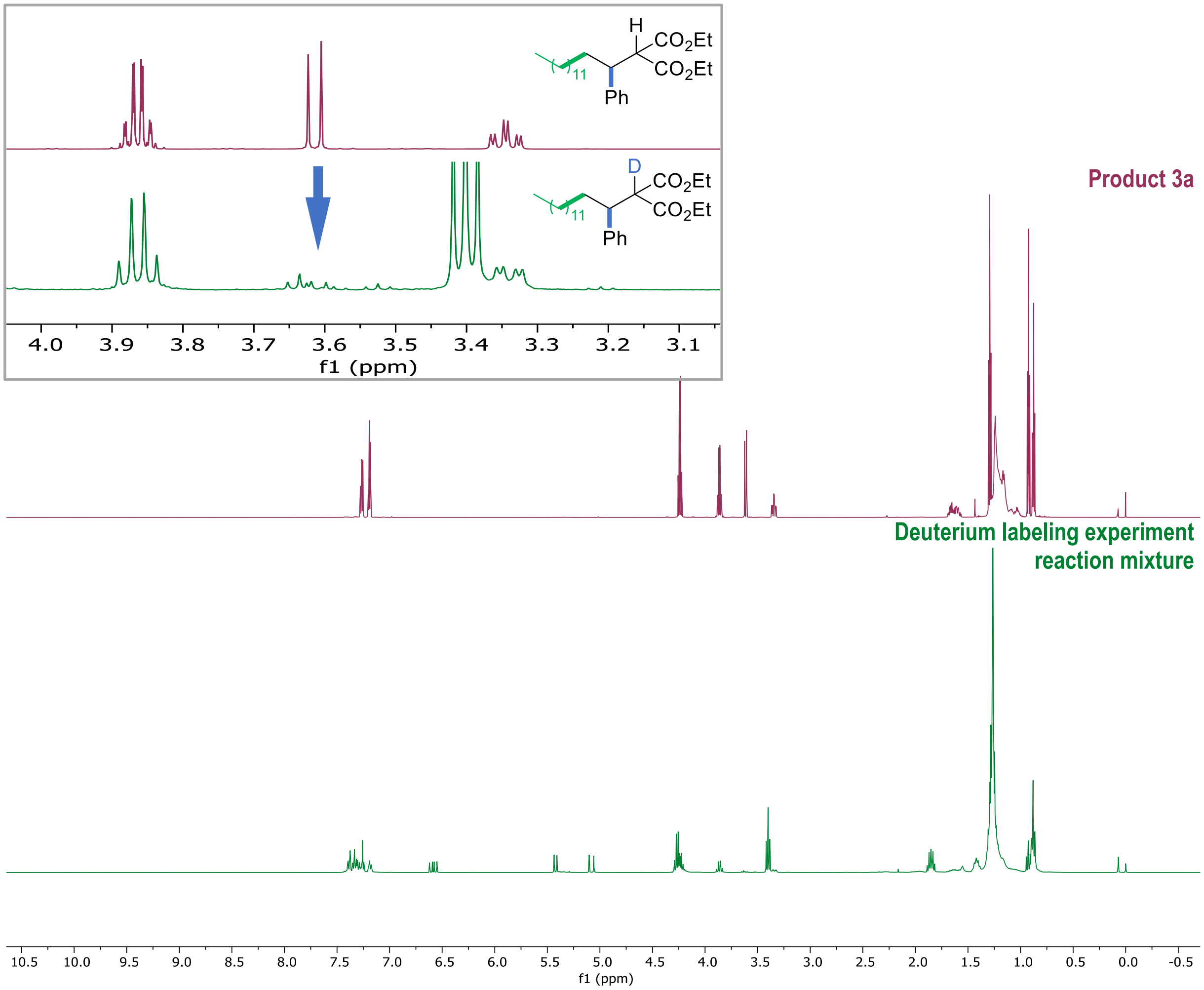

The reaction in D_2O, which is a source of deuterium cation, *provides the desired product with the deuterium atom incorporated at the α-position to the carbonyl group*, thus corroborating the formation of an anion at this position that after protonation furnishes the desired product.

7.5. Undesired products

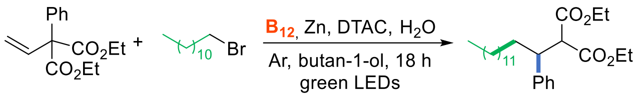

Reaction conditions: diethyl 2-phenyl-2-vinylmalonate (0.10 mmol), 1-bromododecane (3 equiv., 0.30 mmol), vitamin B_{12} (2.5 mol%, 0.003 mmol), Zn (3 equiv., 0.30 mmol), DTAC (3.5 equiv. 0.35 mmol,), *n*-BuOH (12.5 equiv., 1.25 mmol), H_2O (5 mL), green LEDs (525 nm), 16 h, 40 °C.

The reaction was set up following the general procedure 5.1. After 16 h an aliquot was taken from the water and organic phase (during workup) and its composition was studied by LR MS (ESI+).

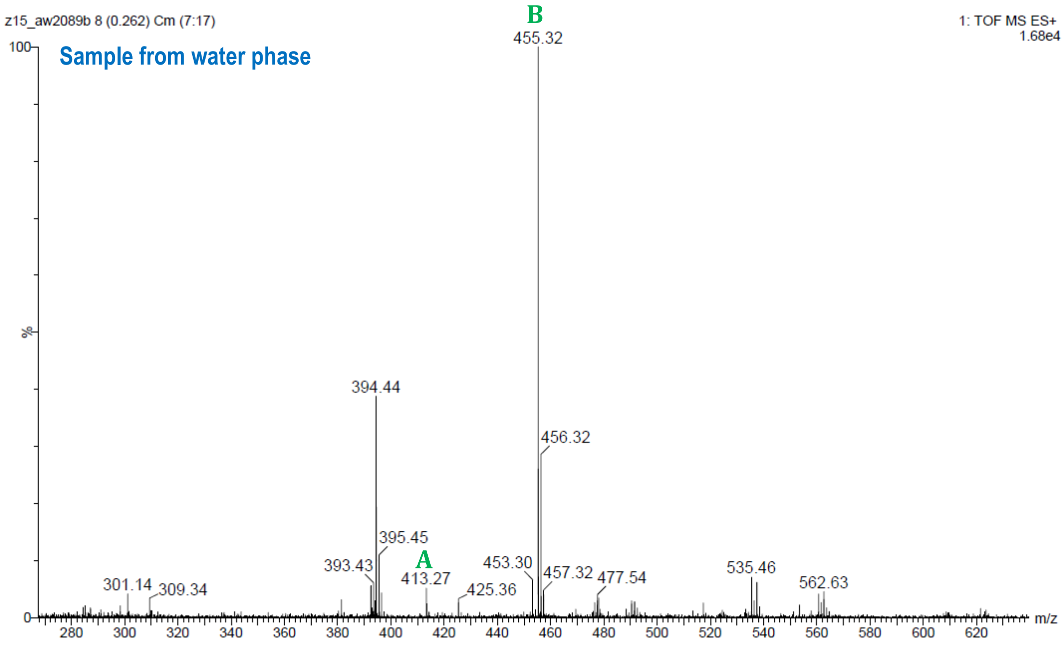

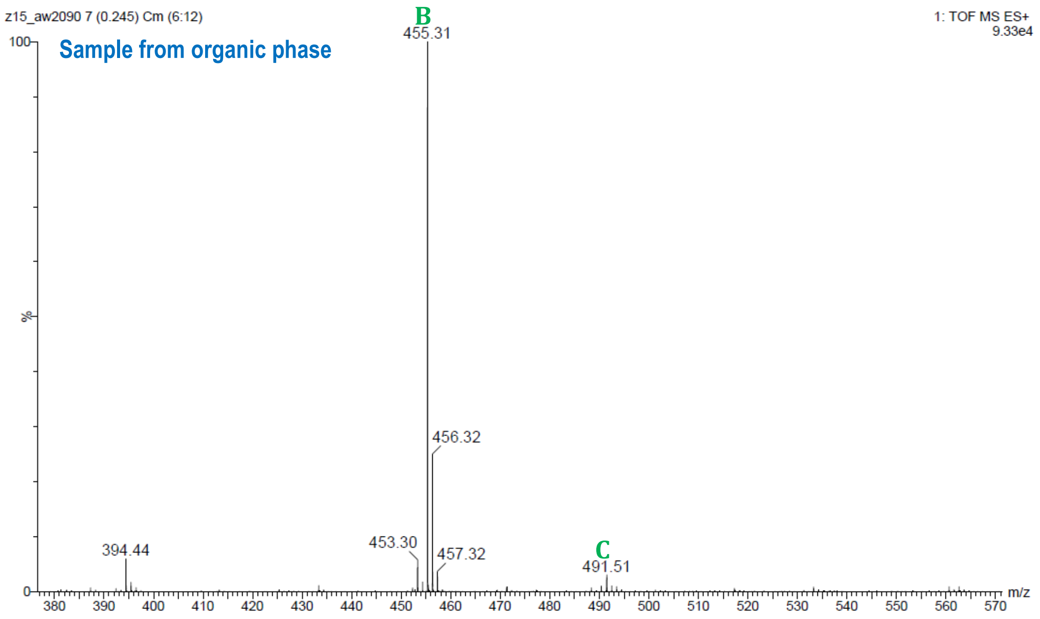

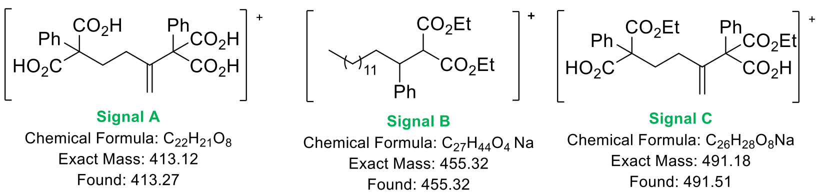

LR MS ESI(+) analysis of the crude reaction mixture indicates the formation of three forms of the complex (signals A, B and C). Signal A corresponds to the mass of dimer with full hydrolysis of ester groups. Signal B corresponds to the mass of the reaction product. Signals C corresponds to the mass of dimer with partial hydrolysis of ester groups.

The small discrepancy between the yield and conversion in model raction (80% vs. 96%) stems from undesired dimerization of the olefin along with full and partial hydrolysis of ester groups.

7.6. NMR spectroscopy measurements and calculation of the sizes.

The samples for NMR spectroscopy were prepared by dissolving the appropriate amount of components in 1 mL of D_2O to obtain a desired concentrations and aliquots (0.7 mL) were taken.

NMR experiments were recorded on a Varian NMR vnmrs 600 MHz spectrometer equipped with a PFG Auto XID (1H/15N-31P 5 mm) indirect z-gradient 5 mm probe head. All NMR spectra, including ^{1}H NMR, 2D ROESY and 2D DOSY experiments, taken for interaction/diffusion studies between all components of water solutions (in D_2O and at 298 K) containing the studied substances (DTAC, olefin, alkyl bromides, *n*-BuOH and vitamin B_{12}), were shaken vigorously just prior to measurements.

Proton spectra were run with 16 scans, at = 4 s and d1 = 1 s acquisition time and delay time (ca. 80 s), respectively. Longer experiments used to investigate different interactions/diffusion phenomena (ROESY/DOSY sequences) were run in a shortest possible time.* ROESY sequence (to identify intra-/intermolecular interactions in appropriate solutions) was run with 4 scans, 256 increments, acquisition time 200 ms, and mixing time 200 ms, total time ca. 50 min). To investigate diffusion and related with it hydrodynamic radius and molecular mass of the solution ingredients the pseudo-2D DOSY experiments (ONESHOT sequence) were run. Typical parameters were: acquisition time - 3.2 s, delay - 2 s, 16 scans for each of 16 gradient levels, with interleave option to average results, Δ - ca. 0.12 – 0.2 s, δ = 0.002 s, total time ca. 24 minutes.

The proton, DOESY and ROESY spectra were processed and analyzed with the MestReNova software packages. The specific diffusion coefficient (D) and molecular weight (M) and hydrodynamic radious (R_H) estimations were determined using SEGWE (The Stokes-Einstein-Gierer-Wirtz Estimation) D/MW Calculator.

* Note that the shaken solutions are “alive” and time where complete recovering (visible in proton spectra, Figure S15, str. S56) of equilibrium is reached approximately 120 minutes.

1.1.1. ^{1}H NMR data for reaction components

Figure S2. DTAC at different concentrations

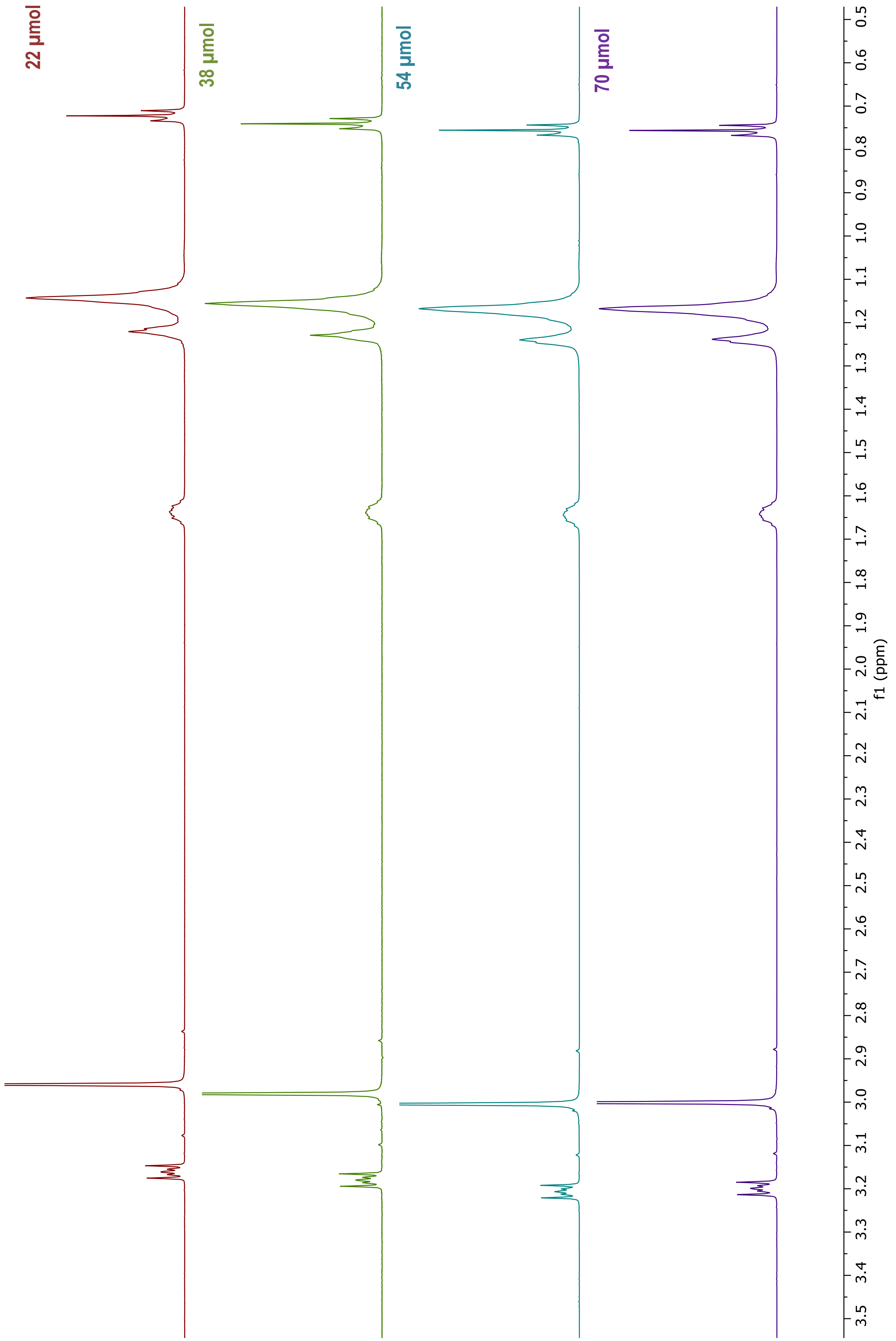

Sample: DTAC at different concetrations in D_2O (1 mL).

Figure S3. DTAC systems.

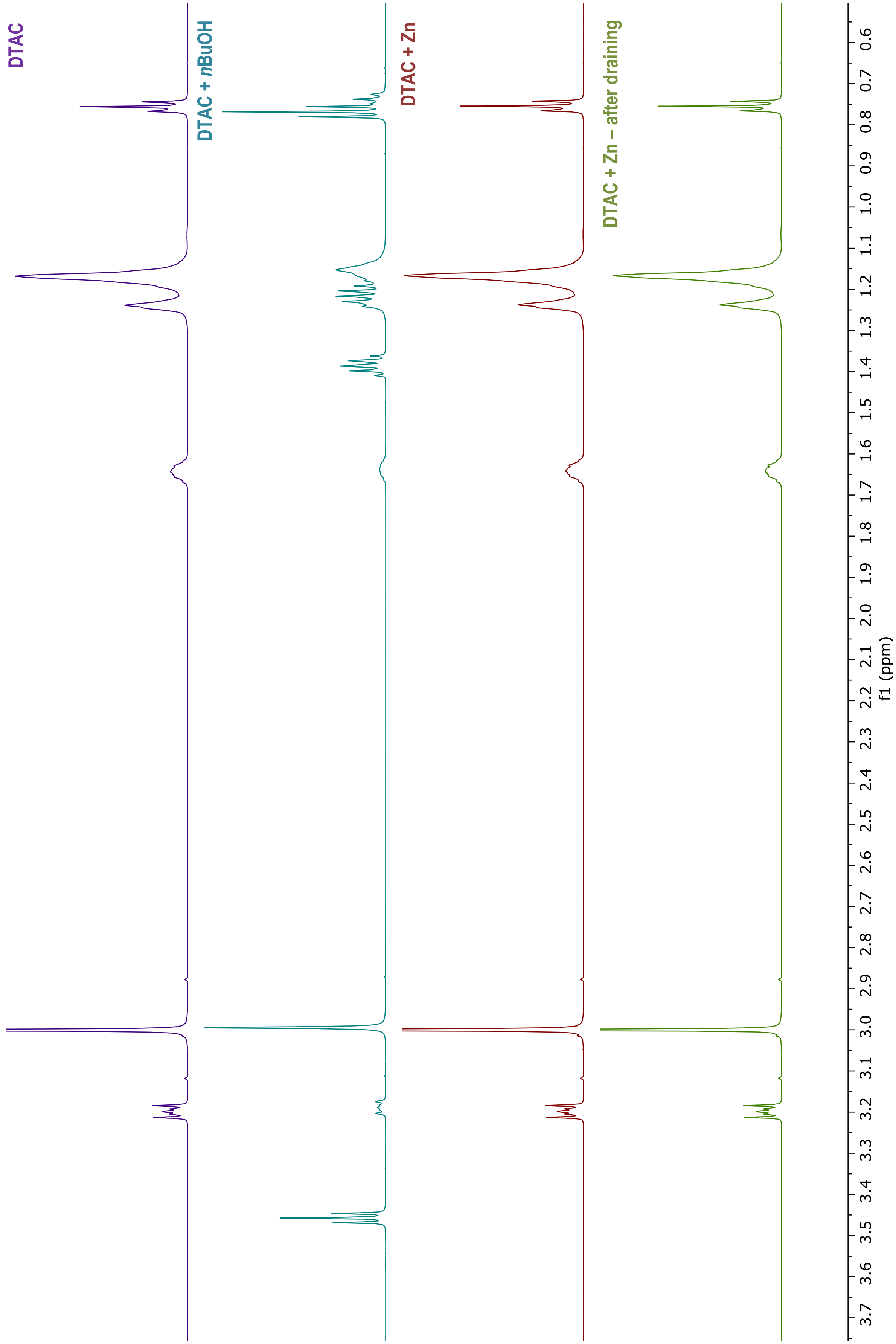

Sample: DTAC (70 µmol) in D_2O (1 mL) with *n*-BuOH (250 µmol), Zn (60 µmol) and Zn (60 µmol) after drying.

Figure S4. Olefin at variable concentration of DTAC.

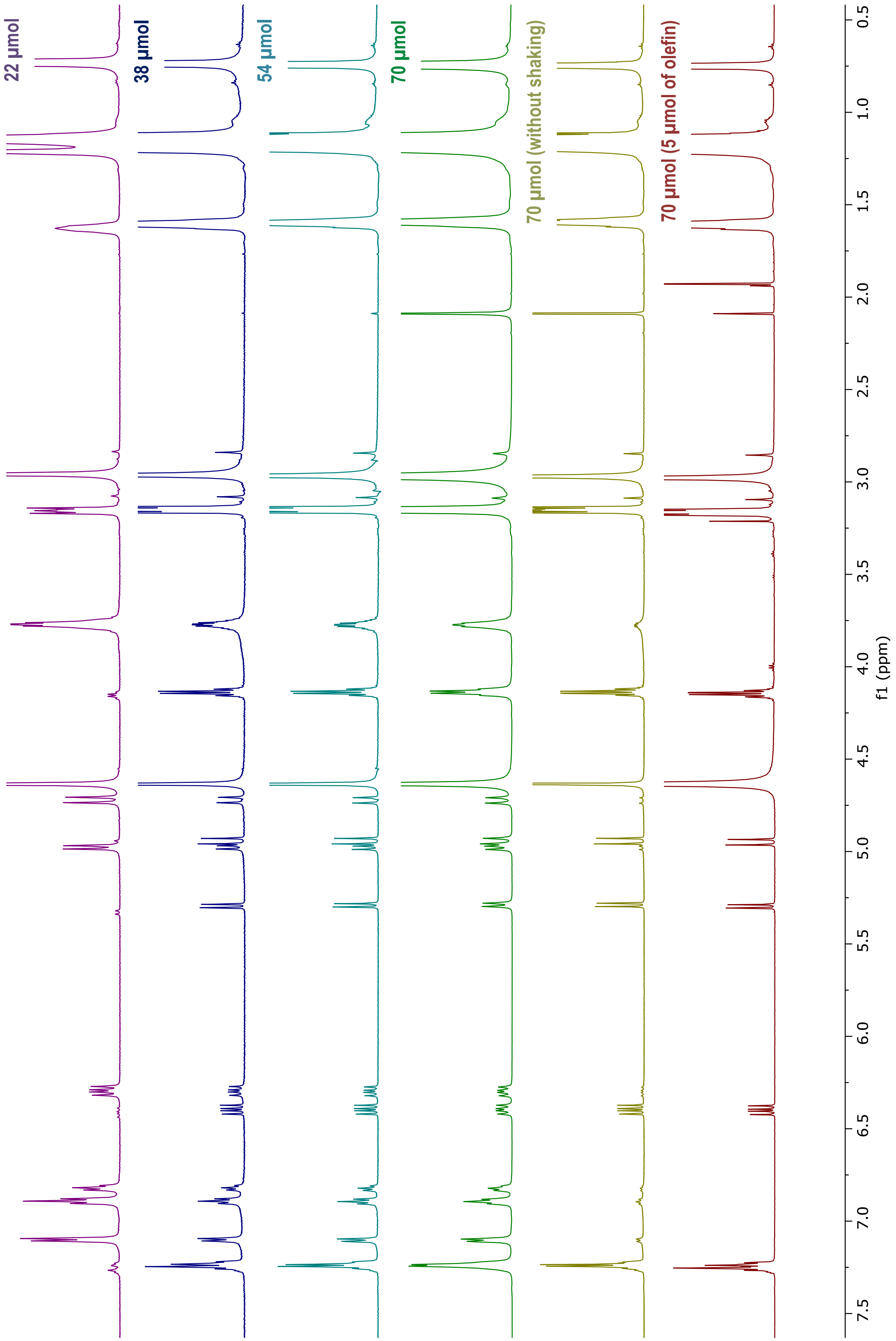

Sample: olefin (20 µmol) in DTAC at different concetrations in D_2O (1 mL).

Figure S5. Aliphatic bromides in DTAC.

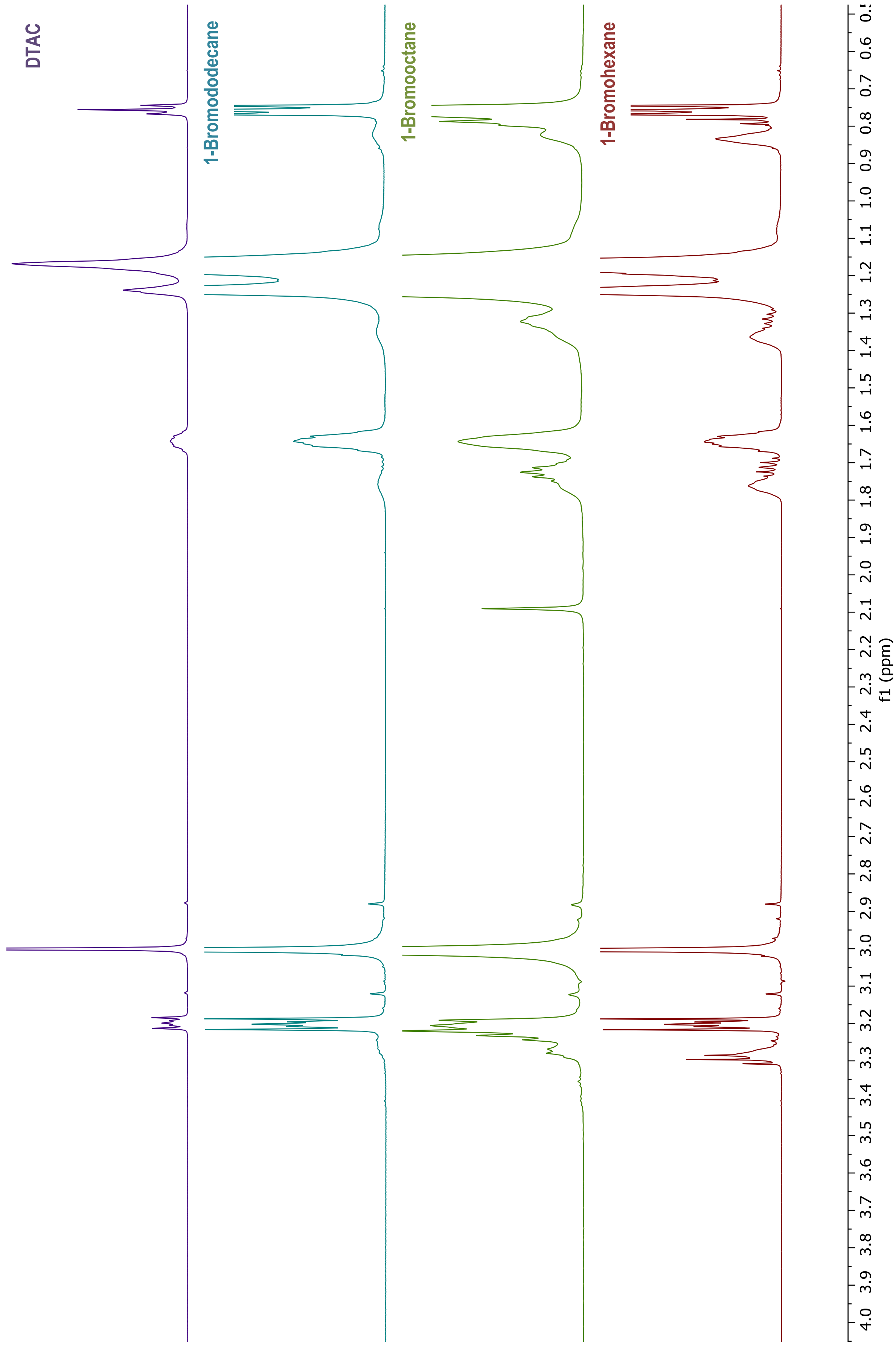

Sample: aliphatic bromides (60 µmol) in DTAC (70 µmol) solution in D_2O (1 mL).

Figure S6. 1-Bromohexane in micellar systems.

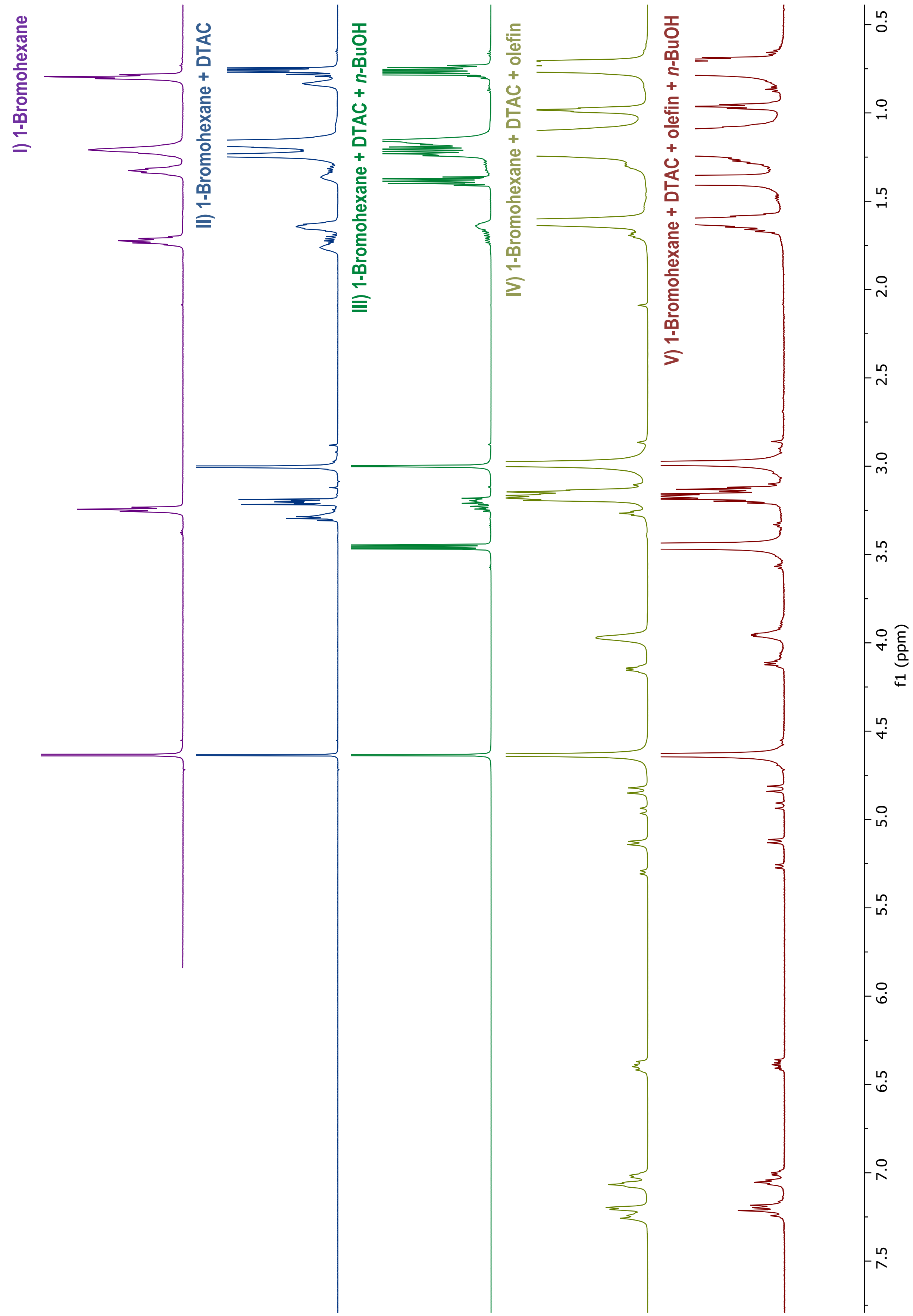

Sample: 1-bromohexane (60 µmol) in I) D_2O (1 mL); II) DTAC (70 µmol) in D_2O (1mL); III) DTAC (70 µmol) in D_2O (1 mL) with *n*-BuOH (250 µmol); IV)) DTAC (70 µmol) in D_2O (1 mL) with olefin (20 µmol); V) DTAC (70 µmol) in D_2O (1 mL) with *n*-BuOH (250 µmol) and olefin (20 µmol).

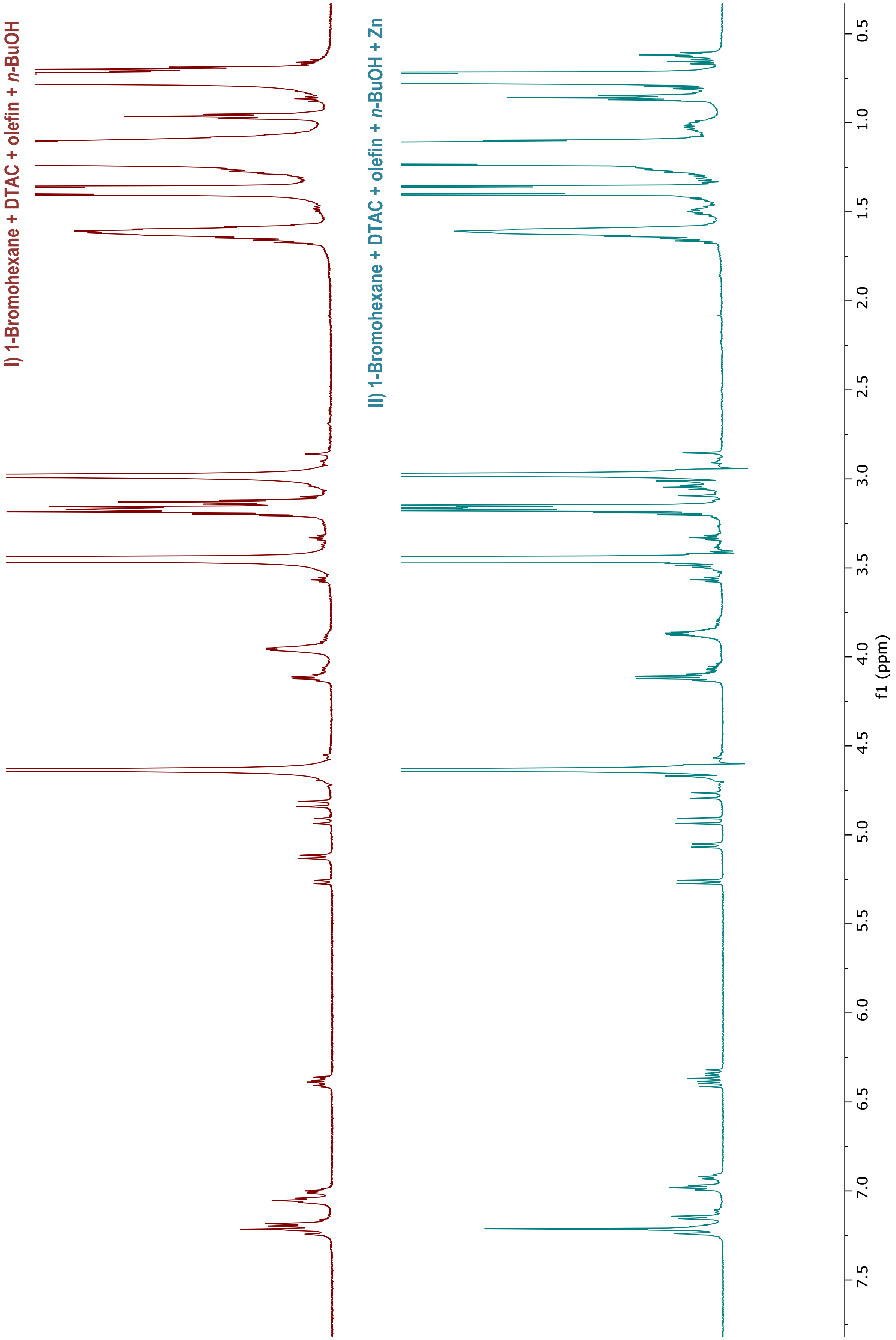

Sample: 1-bromohexane (60 µmol) in I) DTAC (70 µmol) in D_2O (1 mL) with *n*-BuOH (250 µmol) and olefin (20 µmol); II) DTAC (70 µmol) in D_2O (1 mL) with *n*-BuOH (250 µmol), olefin (20 µmol) and Zn (60 µmol).

Figure S7. 1-Bromooctane in micellar systems.

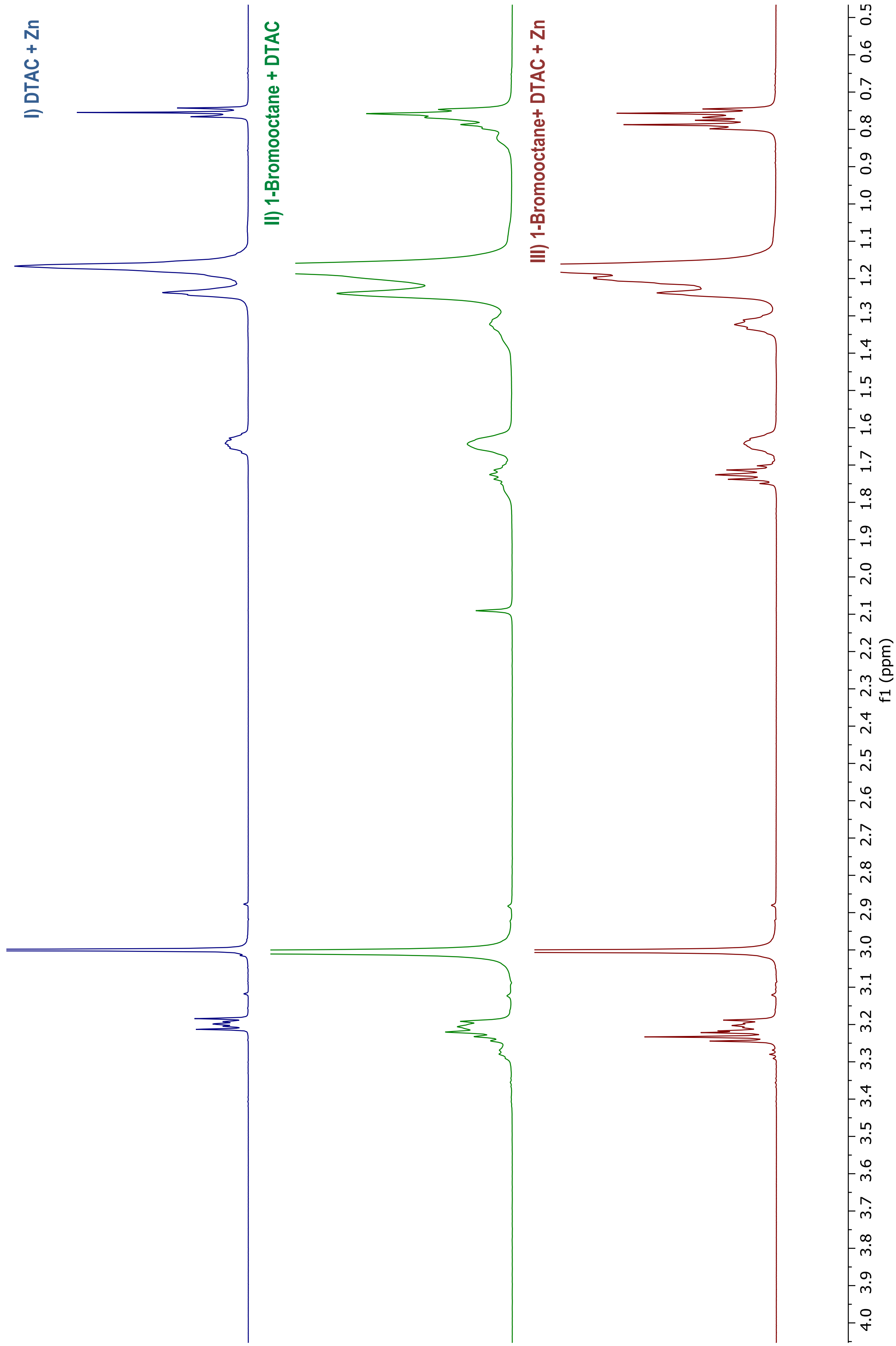

Sample: DTAC (70 µmol) solution in D_2O (1 mL); II) 1-bromooctane (60 µmol) in DTAC (70 µmol) solution in D_2O (1 mL); III) 1-bromooctane (60 µmol) in DTAC (70 µmol) solution in D_2O with Zn (60 µmol).

Figure S8. 1-Bromooctan-2-ol in micellar systems.

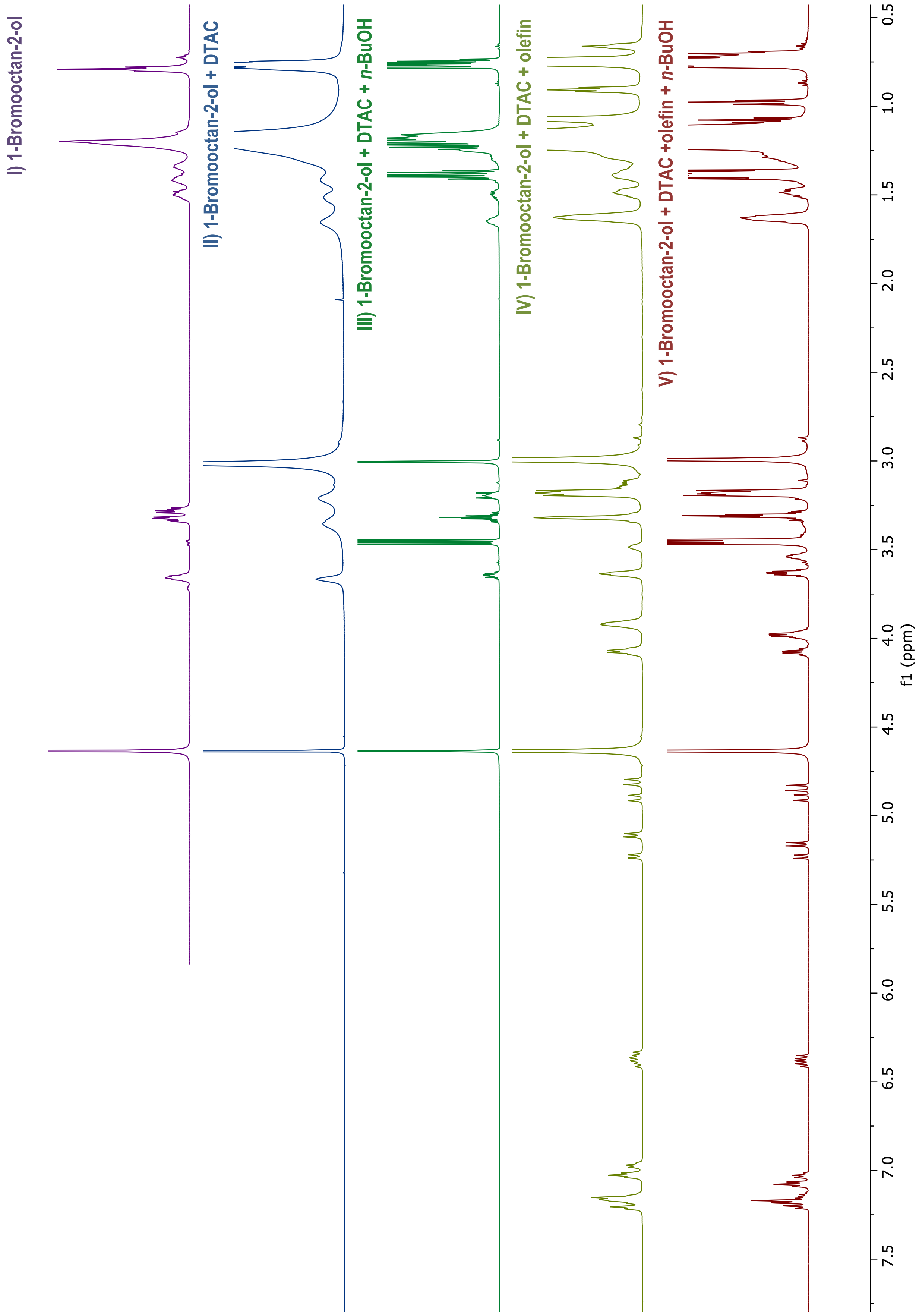

Sample: 1-bromooctan-2-ol (60 µmol) in I) D_2O (1 mL); II) DTAC (70 µmol) solution in D_2O (1 mL); III) DTAC (70 µmol) solution in D_2O (1 mL) with *n*-BuOH (250 µmol); IV) DTAC (70 µmol) solution in D_2O (1 mL) with olefin (20 µmol); V) DTAC (70 µmol) solution in D_2O (1 mL) with *n*-BuOH (250 µmol) and olefin (20 µmol).

Figure S9. 8-Bromooctan-1-ol in micellar systems.

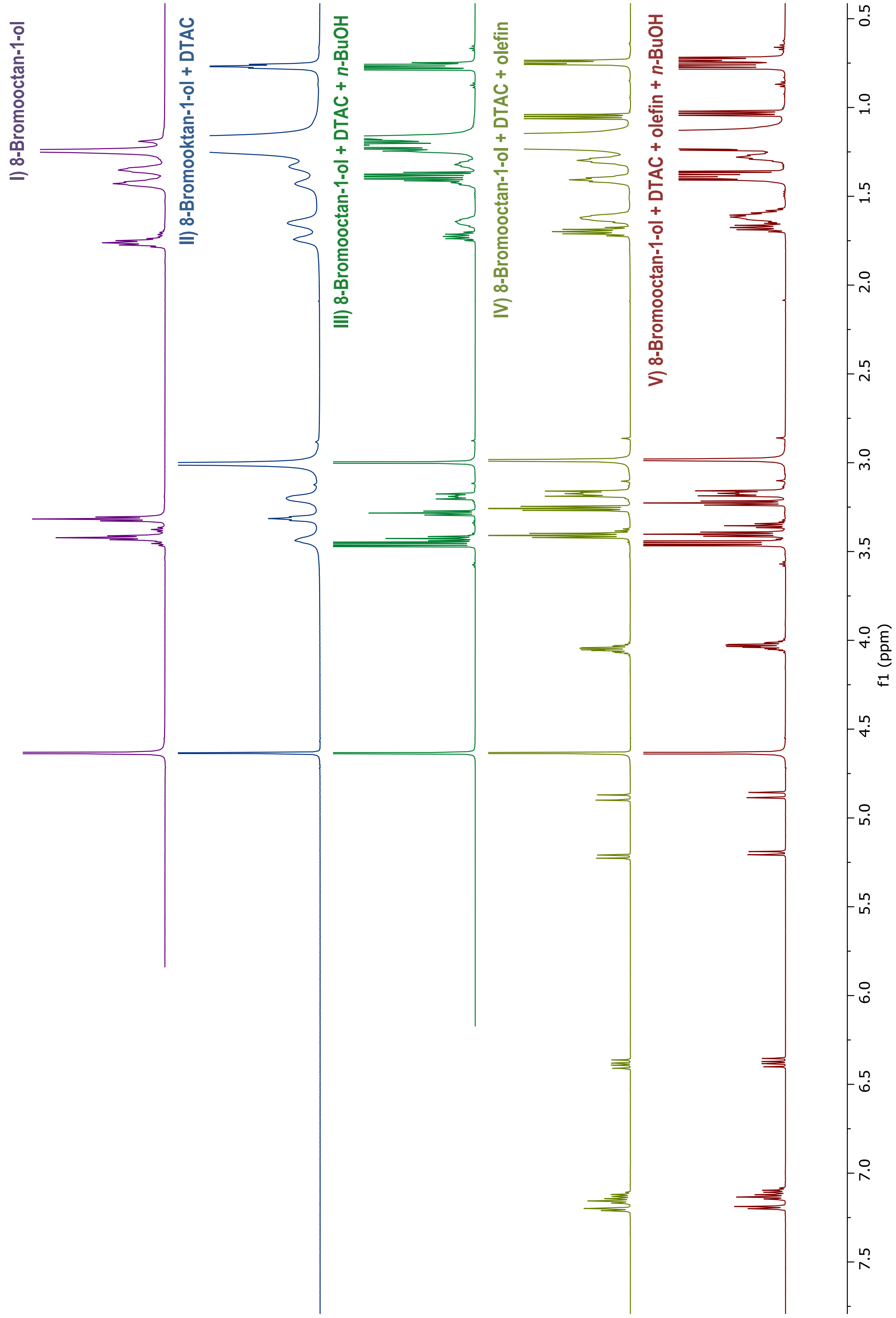

Sample: 8-bromooktan-1ol (60 µmol) in I) D_2O (1 mL); II) DTAC (70 µmol) solution in D_2O (1 mL); III) DTAC (70 µmol) solution in D_2O (1 mL) with *n*-BuOH (250 µmol); IV) DTAC (70 µmol) solution in D_2O (1 mL) with olefin (20 µmol); V) DTAC (70 µmol) solution in D_2O (1 mL) with *n*-BuOH (250 µmol) and olefin (20 µmol).

Figure S10. Product 3a in micellar systems.

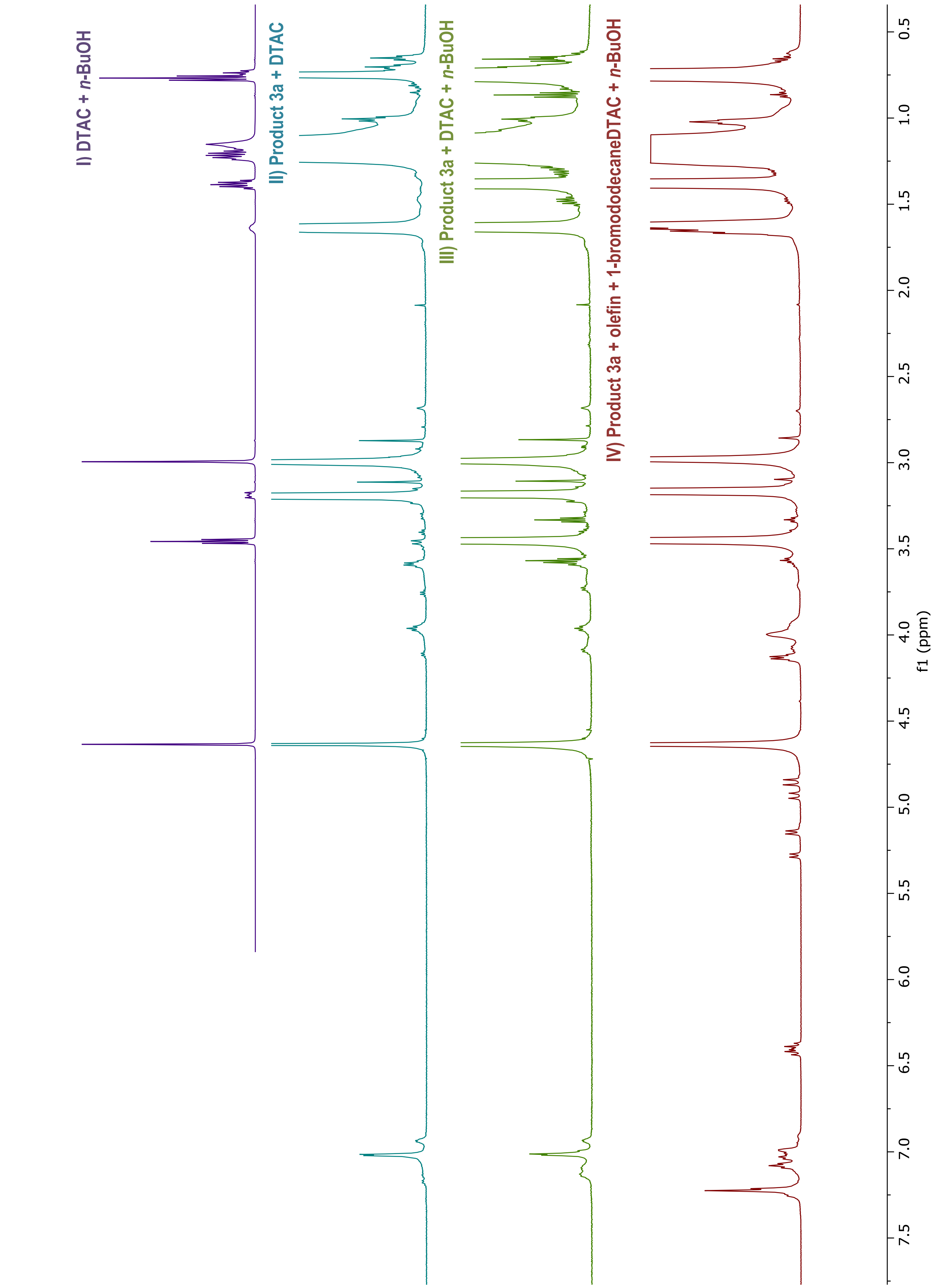

Sample: I) DTAC (70 µmol) solution in D_2O (1 mL) with *n*-BuOH (250 µmol); Product 3a (10 µmol) in II) DTAC (70 µmol) solution in D_2O (1 mL); III) DTAC (70 µmol) solution in D_2O (1 mL) with *n*-BuOH (250 µmol); IV) DTAC (70 µmol) solution in D_2O (1 mL) with *n*-BuOH (250 µmol), olefin (10 µmol) and 1-bromododecane (30 µmol).

Figure S11. Vitamin B_{12} in micellar systems.

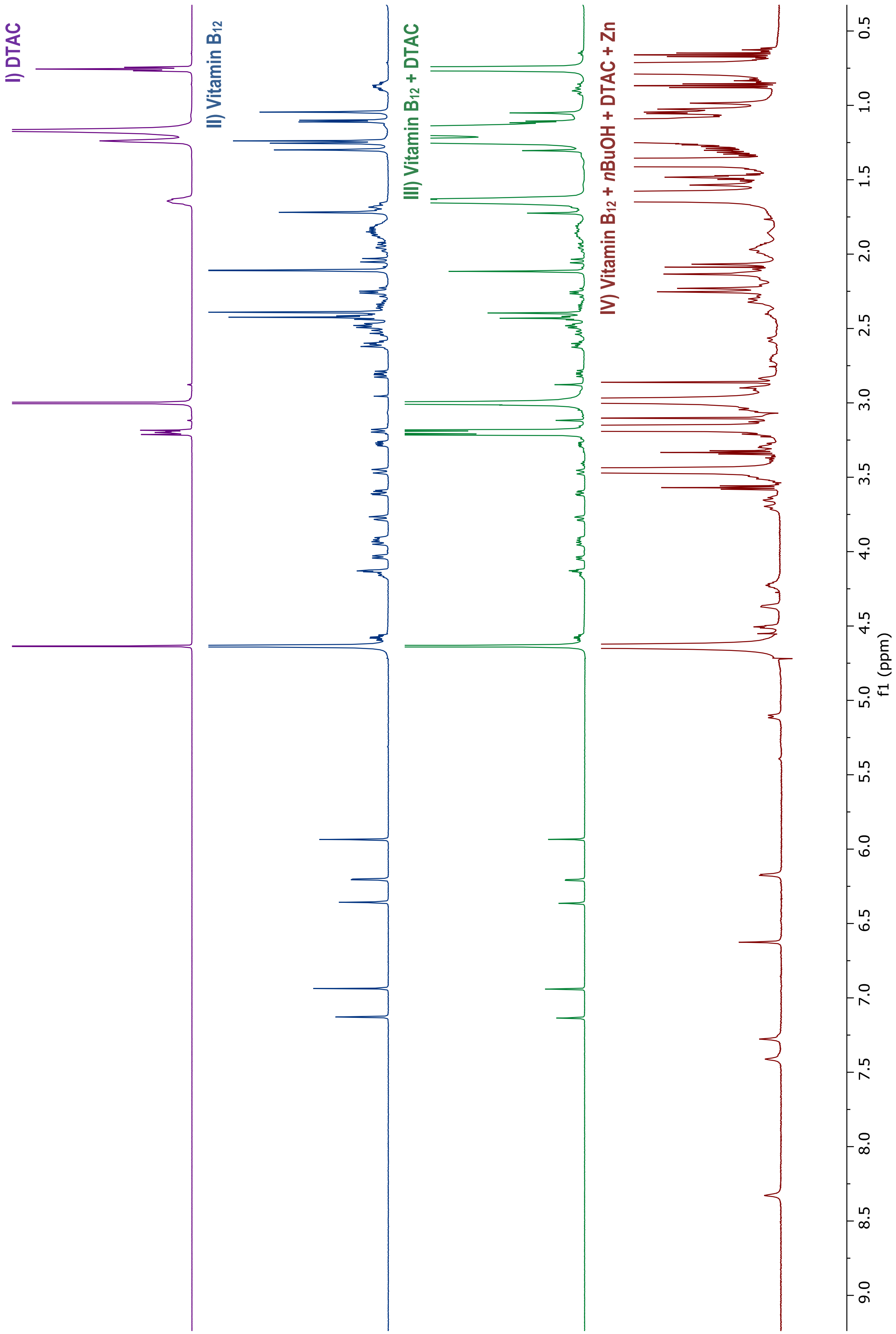

Sample: I) DTAC (70 µmol) solution in D_2O (1 mL); II) vitamin B_{12} (0.6 µmol) in D_2O (1 mL); III) vitamin B_{12} (0.6 µmol) in DTAC (70 µmol) solution in D_2O (1 mL); IV) vitamin B_{12} (2.4 µmol) in DTAC (70 µmol) solution in D_2O (1 mL) with *n*-BuOH (250 µmol) and Zn (240 µmol).

Figure S12. Alkyl cobalamin.

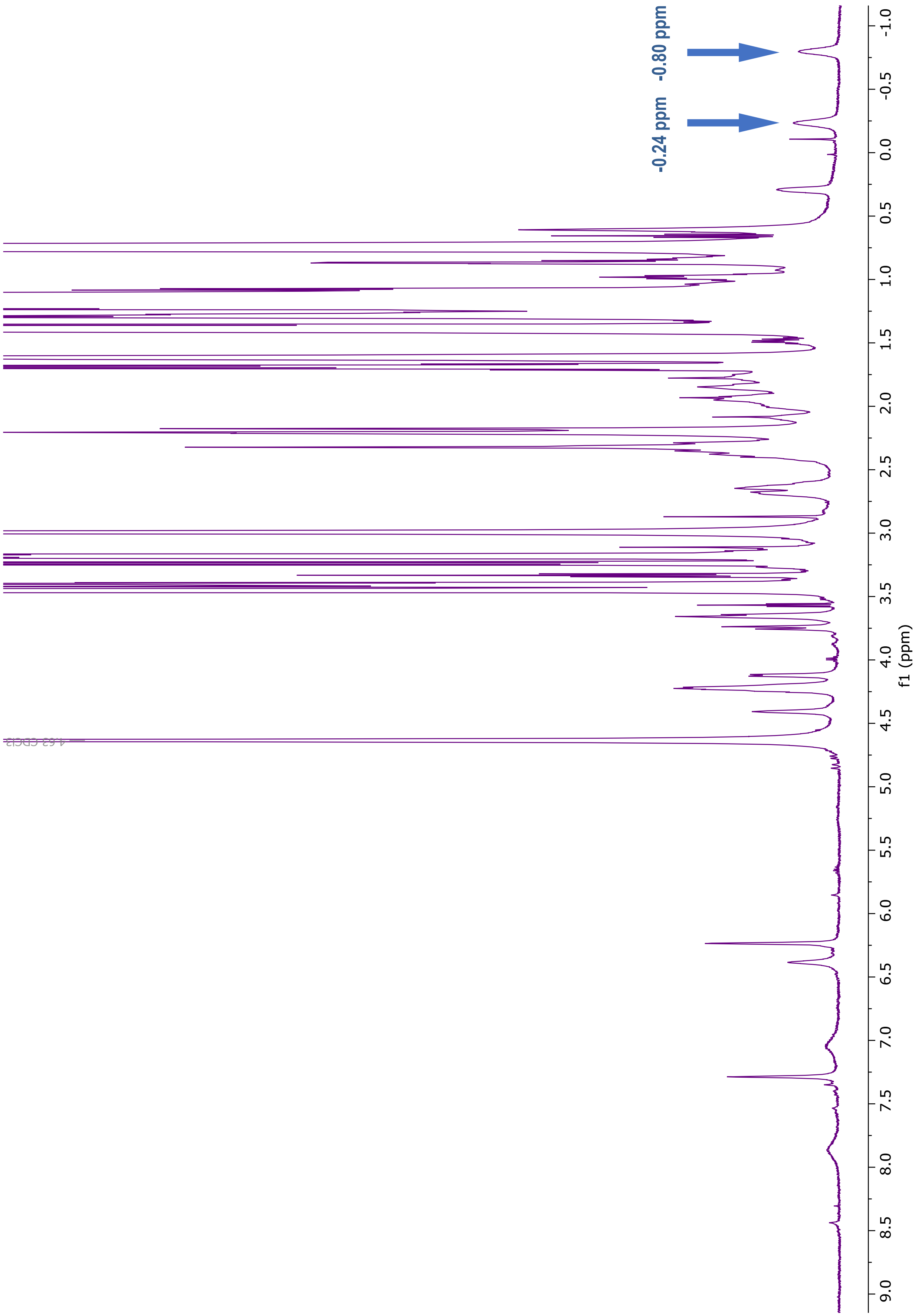

Sample: vitamin B_{12} (10 µmol) in DTAC (70 µmol) solution in D_2O (1 mL) with *n*-BuOH (250 µmol), 1-bromooctan-2-ol (60 µmol) and Zn (308 µmol).

Figure S13. Organozinc compoud.

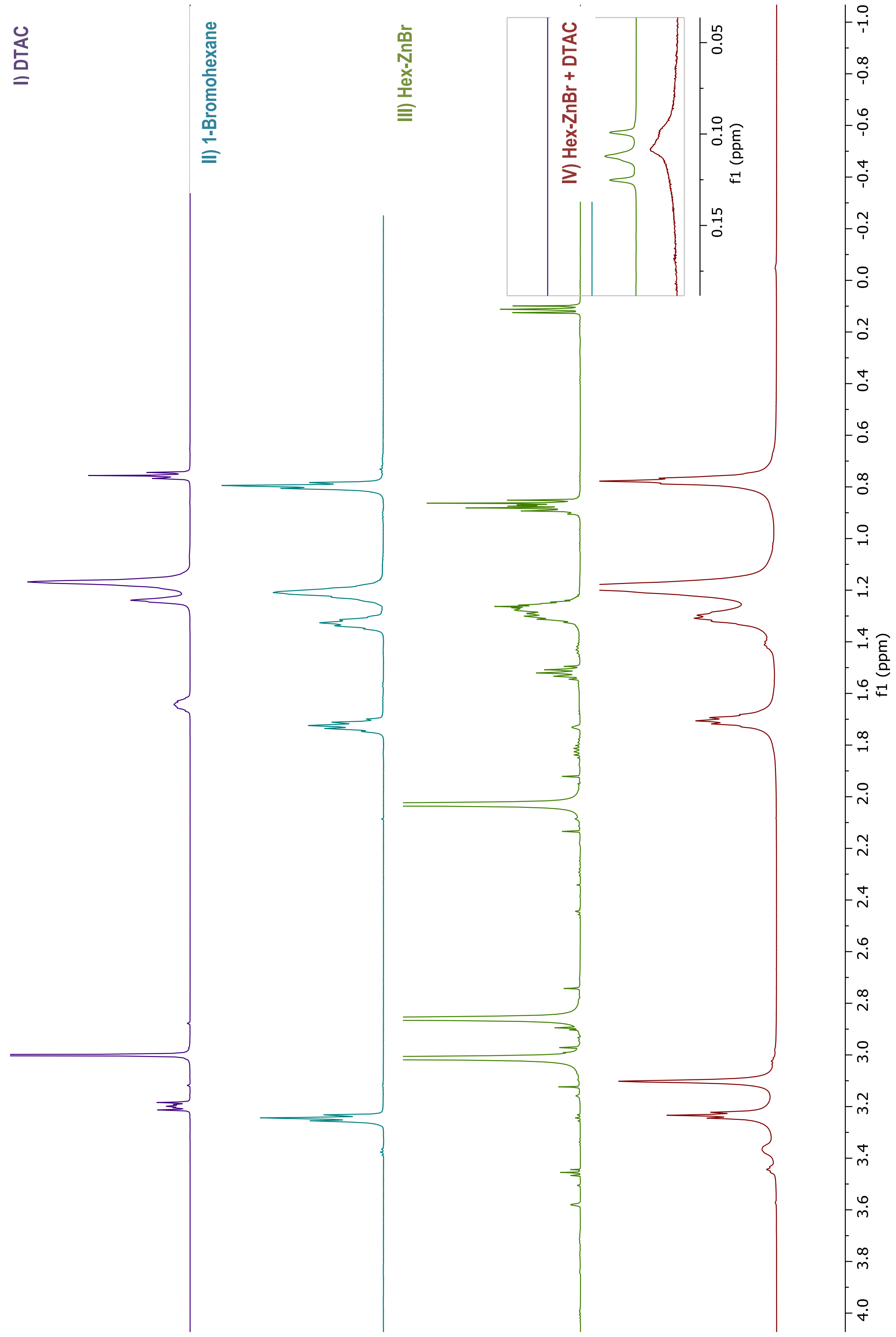

Sample: I) DTAC (70 µmol) solution in D_2O (1 mL); II) 1-bromohexane (60 µmol) in D_2O (1 mL); III) Hex-ZnBr in THF_{d8}; IV) Hex-ZnBr in DTAC (70 µmol) solution in D_2O (1 mL).

Figure S14. ROESY NMR spectra of olefin 1 in micellar systems.

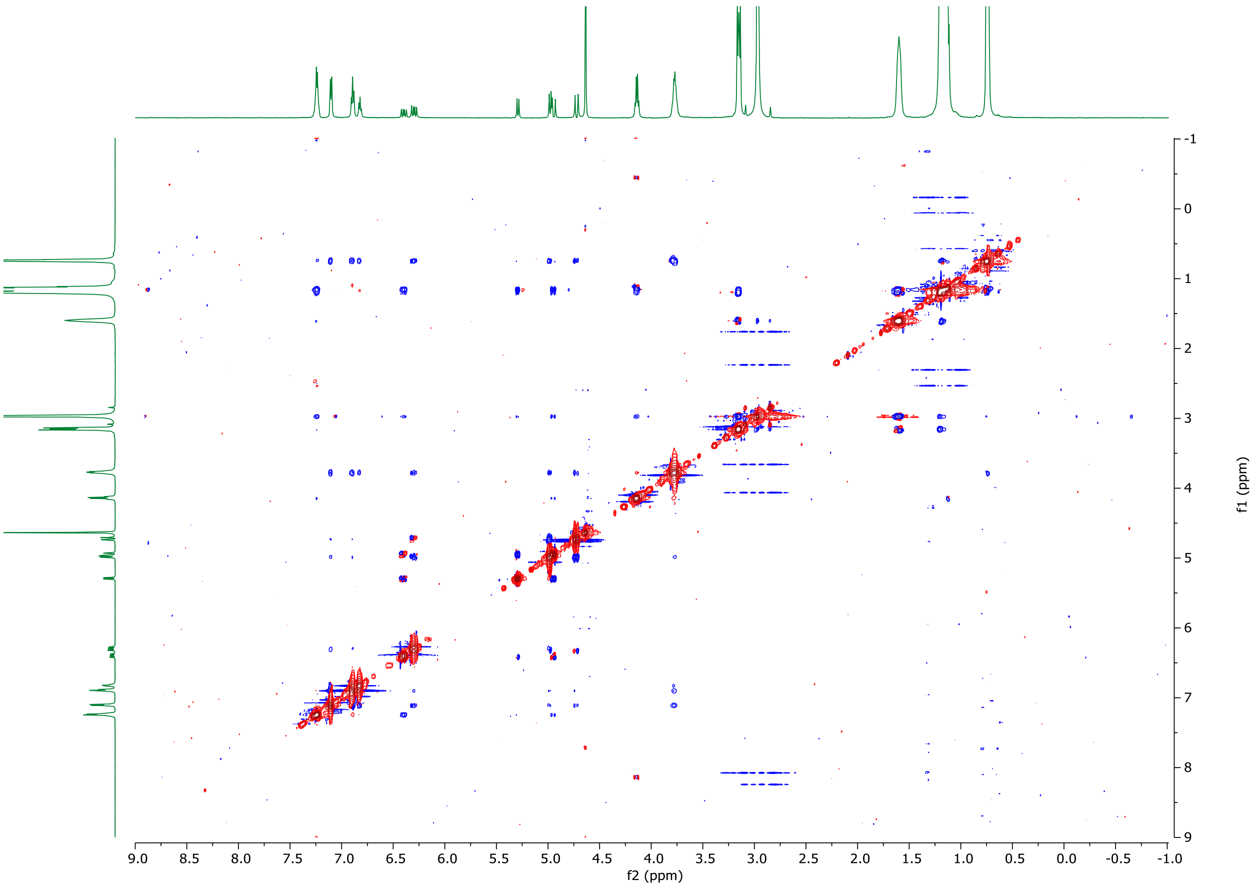

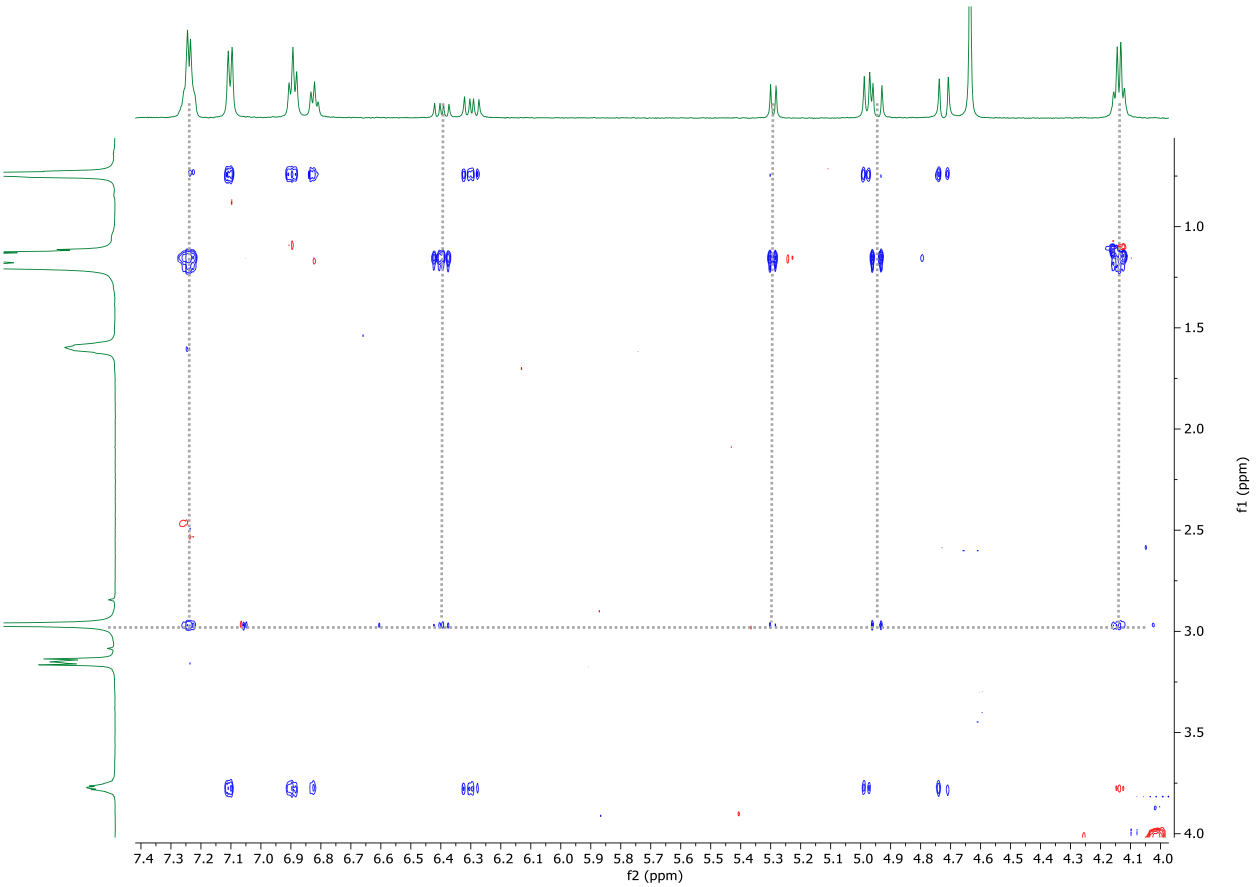

Sample: olefin 1 (20 µmol) in DTAC (54 µmol) solution in D_2O (1 mL).

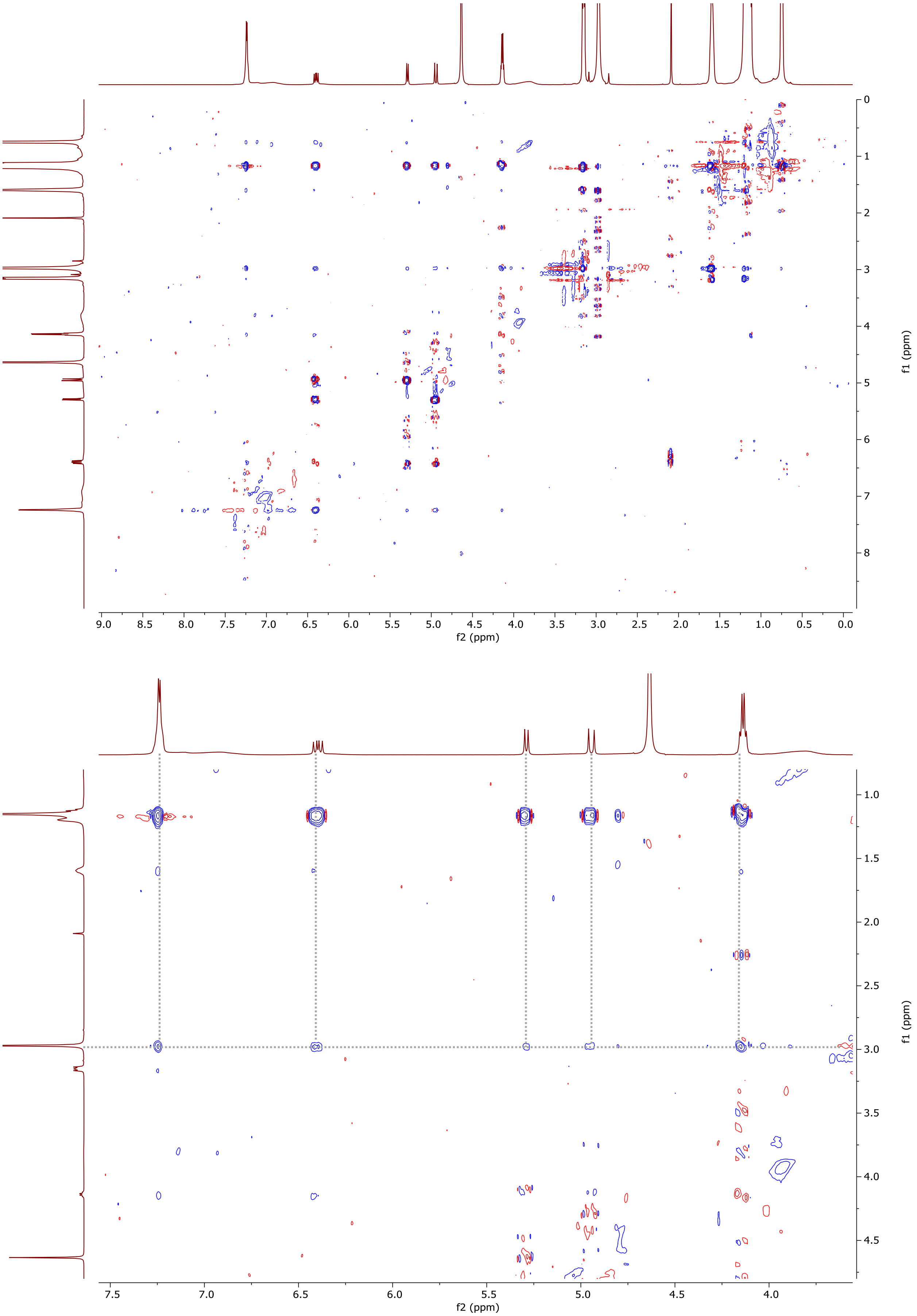

Sample: olefin 1 (20 µmol) in DTAC (70 µmol) solution in D_2O (1 mL).

Figure S15. Olefin 1 in micellar system over time.

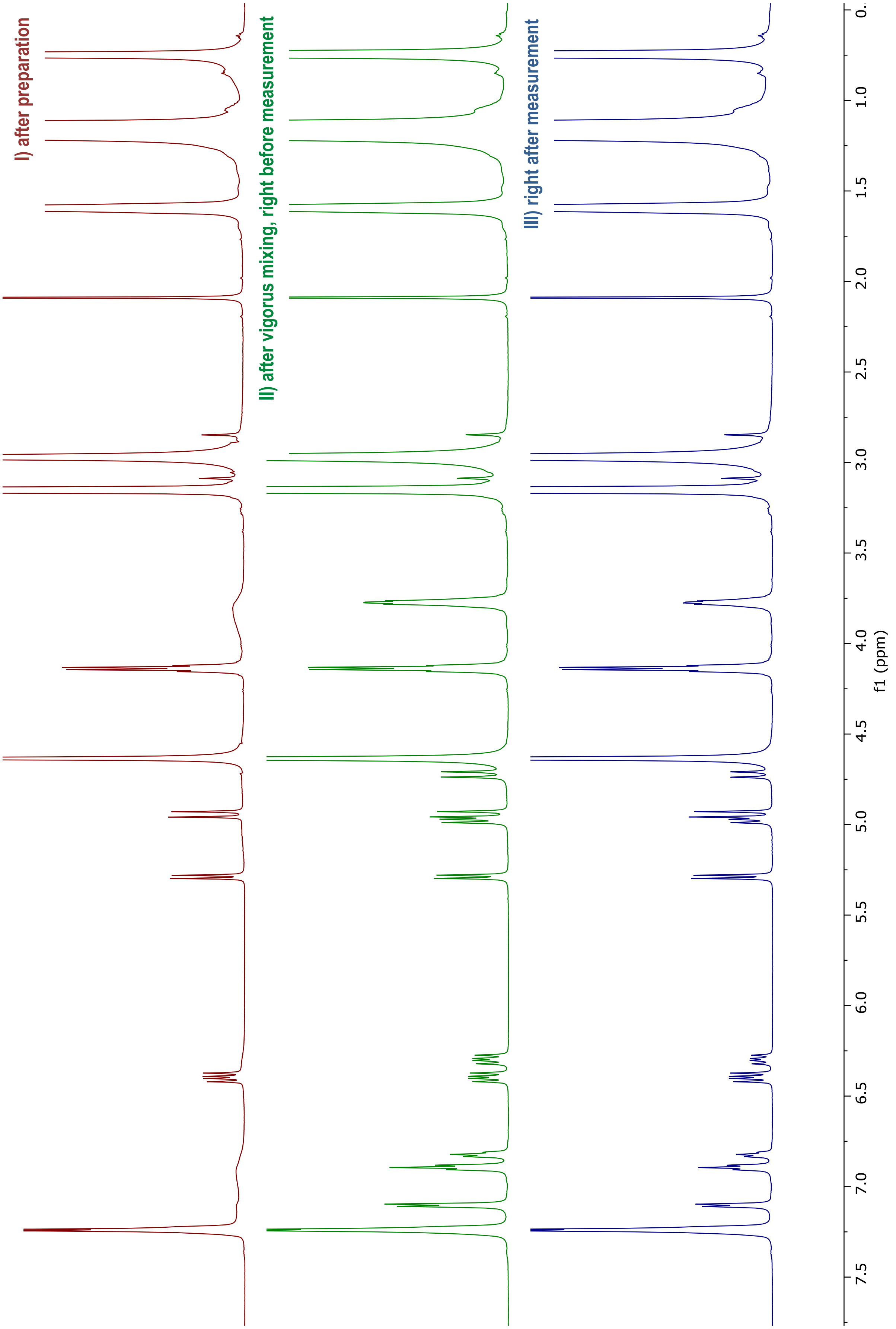

Sample: olefin 1 (20 µmol) in DTAC (70 µmol) solution in D_2O (1 mL).

Figure S16. *2D DOSY NMR spectra of vitamin B_{12} in micellar systems.*

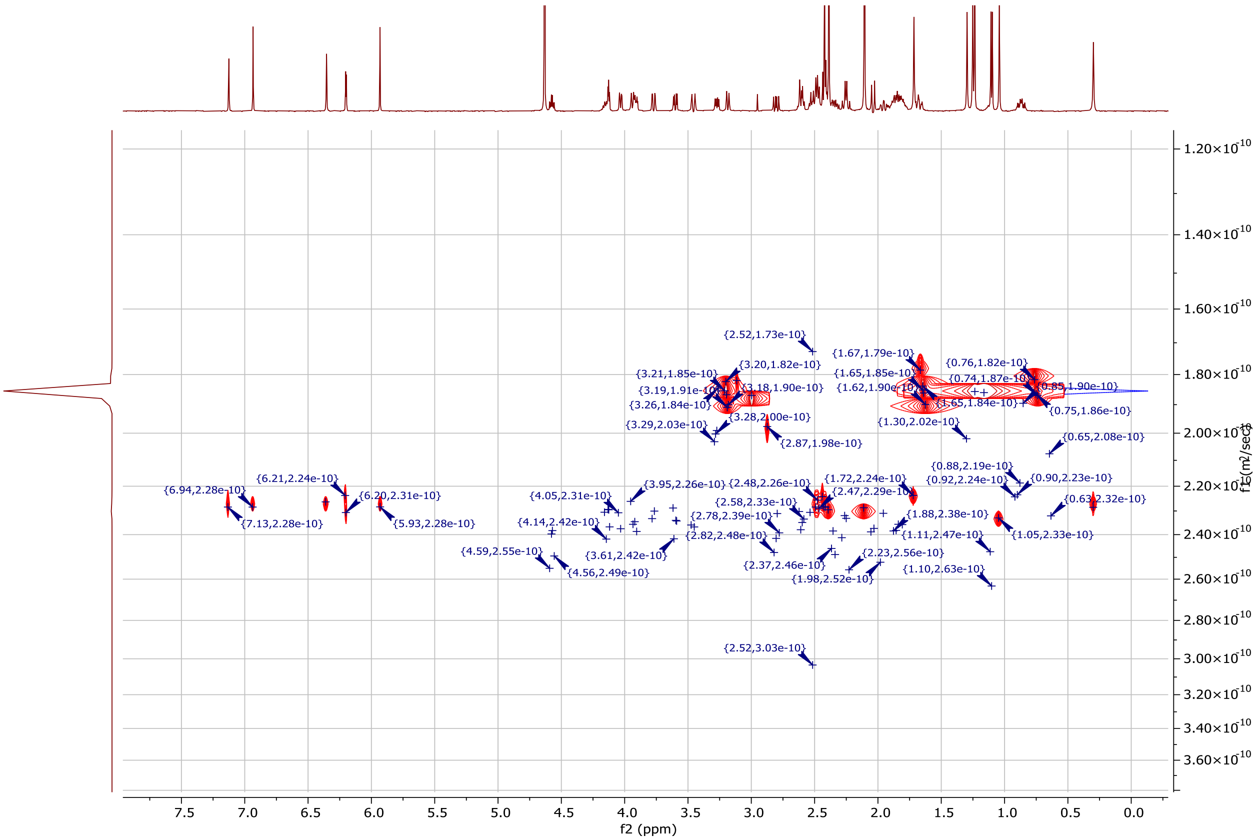

Sample: vitamin B_{12} (0.6 µmol) in DTAC (70 µmol) solution in D_2O (1 mL).

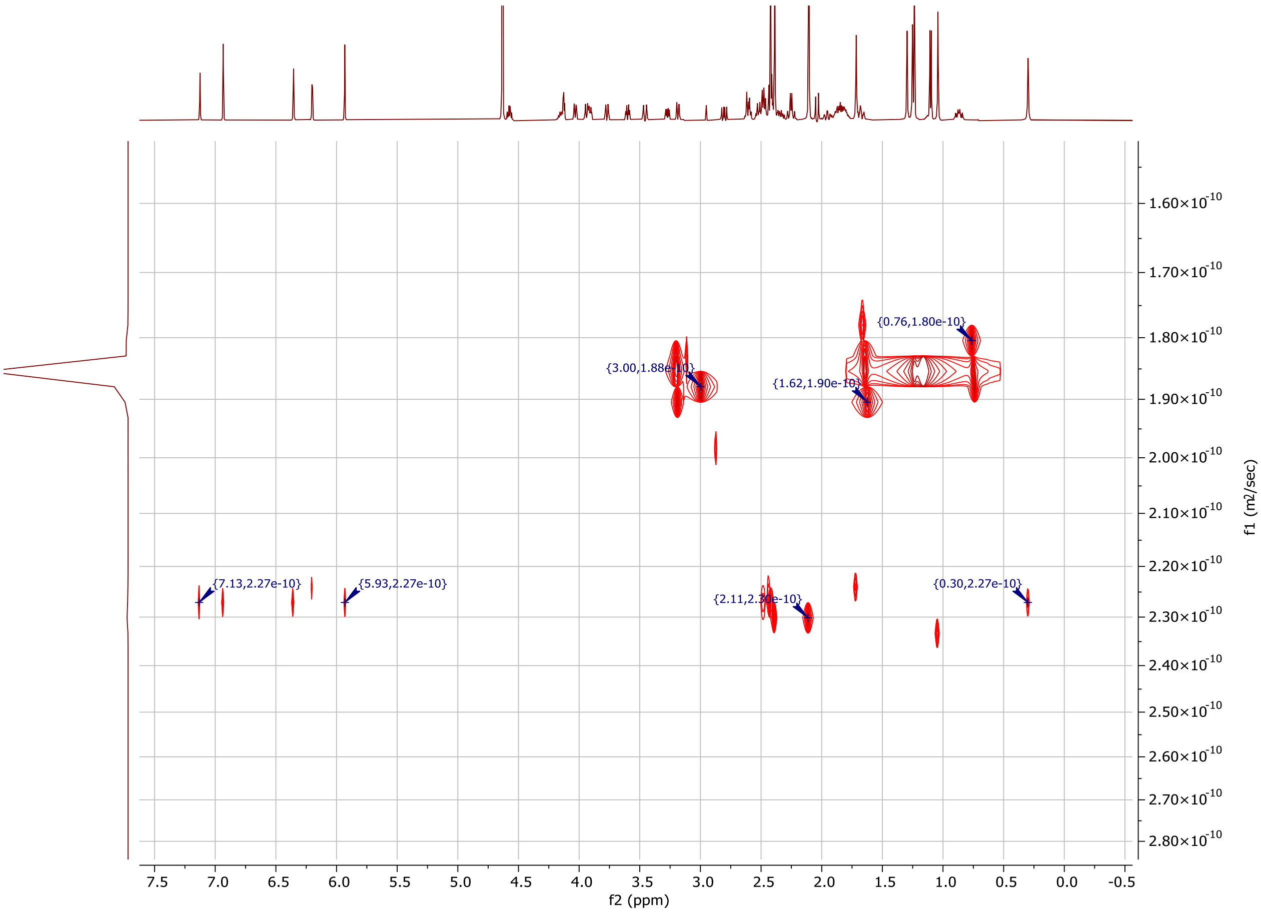

7.7. Computational Methods and Additional Results

The density functional theory (DFT) calculations were performed using Turbomole 7.3.[8] We used the BP functional[9] and the TZVP basis set[10] along with the COSMO implicit solvent model[11] using an infinite dielectric constant, in order to allow for COSMO-RS[12] calculations. The ensuing COSMO-RS calculations were performed using COSMOtherm 21 and the BP_TZVP_C30_1601 parameterisation. The DTAC surfactant was modelled as a contact ion pair, to make it a neutral molecule, which is a requirement for the interfacial tension calculations.

We predicted the critical micellar concentration (CMC) using our recent method[13] with dodecane as the equivalent tail model for the surfactant. The method is based on our COSMO-RS based method for predicting liquid-liquid interfacial tension (IFT)[14] and allows to calculate the interfacial mole fraction of all components at the liquid-liquid interface, which in our case is the micelle-water interface. The first part of any liquid-liquid IFT calculation is a liquid extraction calculation, an equilibrium calculation between the two bulk phases, which were

1. Surfactant + water
2. Dodecane (modelling surfactant tail), alkyl bromide and olefin

In short, for the system including all components in the calculation (including the bromide and olefine reactants), the CMC was found by changing the surfactant concentration in the calculations until the computed IFT was equal to 0. Thermodynamically, this is the concentration at which the free Energy cost for creating the micelle-water interface vanishes, and micelles can start to form spontaneously. For more details on the procedure, see[13].

Table S1. COSMO-RS predicted equilibrium mole fractions in the surface phase in the IFT calculations (the micellar interface region). The length of the aliphatic chain in the bromide is denoted by x.

entry	x	water	DTAC	bromide 2	olefin 1
1	2	0.73	0.12	0.140	0.009
2	4	0.75	0.14	0.098	0.012
3	6	0.77	0.15	0.071	0.014
4	8	0.78	0.15	0.048	0.017
5	10	0.79	0.16	0.033	0.019
6	12	0.80	0.16	0.022	0.022
7	15	0.80	0.16	0.012	0.025
8	18	0.80	0.16	0.007	0.029
9	22	0.80	0.16	0.004	0.034

[8] R. Ahlrichs; M. Bar; M. Haser; H. Horn and C. Kolmel; *Chem. Phys. Lett.,* **1989**, *162*, 165–169.

[9a] A. D. Becke;. *Phys. Rev. A,* ***1988**, 38,* 3098. [9b] J. P. Perdew, *Phys. Rev. B*, **1986**, 33, 8822–8824.

[10] F. Weigend and R. Ahlrichs, *Phys. Chem. Chem. Phys.,* **2005**, **7**, 3297–3305.

[11] A. Klamt and G. Schüürmann; *J. Chem. Soc. Perkin Trans.,* **1993**, *2,* 799–805.

[12] A. Klamt, F. Eckert and W. Arlt, in Annual Review of Chemical and Biomolecular Engineering, **2010**, *vol. 1*, 101–122.

[13] M. Turchi, A. P. Karcz and M. P. Andersson, J. Colloid Interface Sci., **2022**, *606*, 618–627.

[14] M. P. Andersson, M. Bennetzen, A. Klamt and S. L. S. Stipp, *J. Chem. Theory Comput.*, **2014,** *10*, 3401–3408.

7.8. Dynamic-light-scattering (DLS) measurements

The samples for DLS were prepared by dissolving the appropriate amount of DTAC in 3 mL of H_2O and after 2 h in 40 °C aliquots were taken.

Measurements of dynamic-light-scattering (DLS) were conducted using Malvern Zetasizer Nano – ZS at 40°C, which corresponds to the conditions.

Table S2. DLS measurements of micelle size.[a]

No	C_{DTAC} [mM]	additives	d [nm]
1	70	-	1.27
2	54	-	1.12
3	38	-	0.72
4	22	-	-
5	70	1-bromooctan-2-ol, *n*-BuOH	0.91
6	70	8-bromooctan-1-ol, *n*-BuOH	0.73

[a]**Measurements conditions:** bromide (15 µL), DTAC, *n*-BuOH (68 µL), H_2O (3 mL), 40 °C.

Analysis of the data confirmed the presence of small micelles under the conditions developed and their increase in size with increasing surfactant concentration.

8. NMR spectra

diethyl 2-(1-phenyltetracosyl)malonate, 3b

^{1}H NMR ($CDCl_3$, 500 MHz)

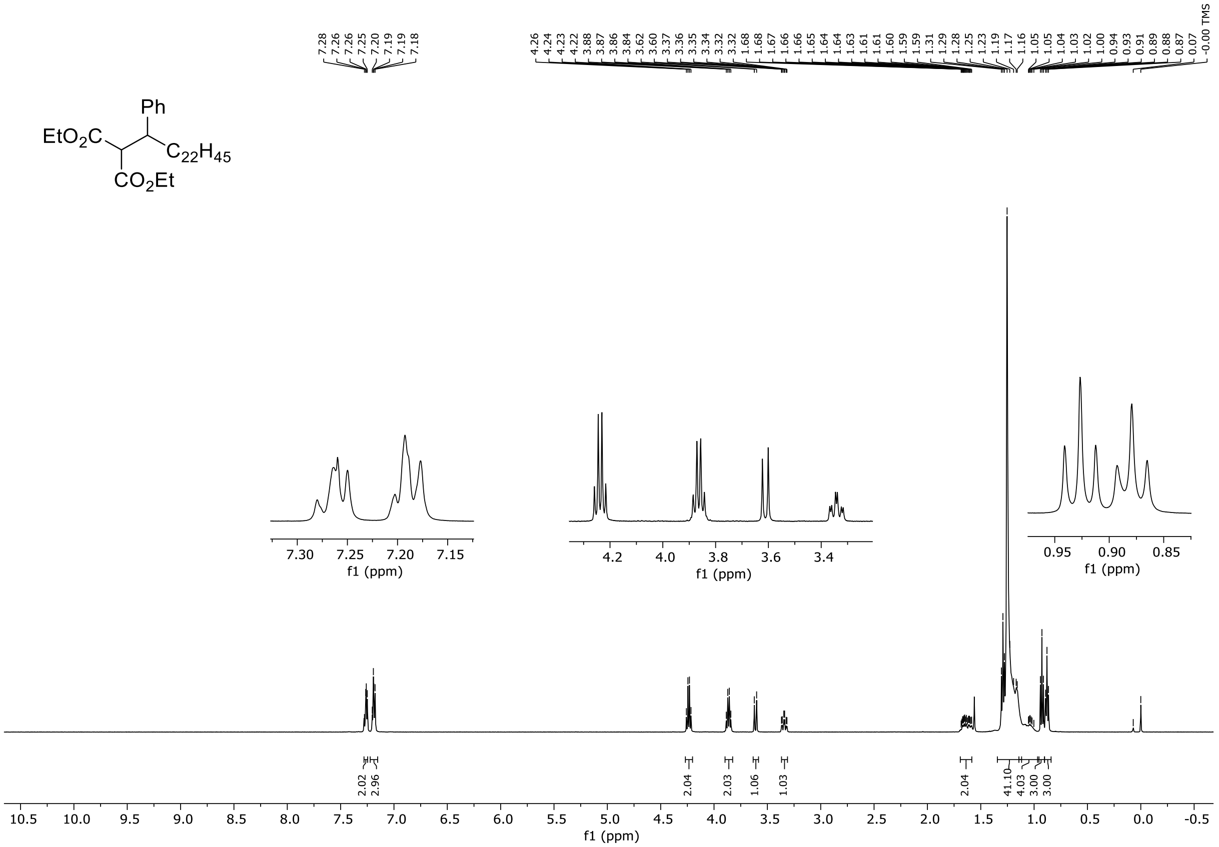

^{13}C NMR ($CDCl_3$, 126 MHz)

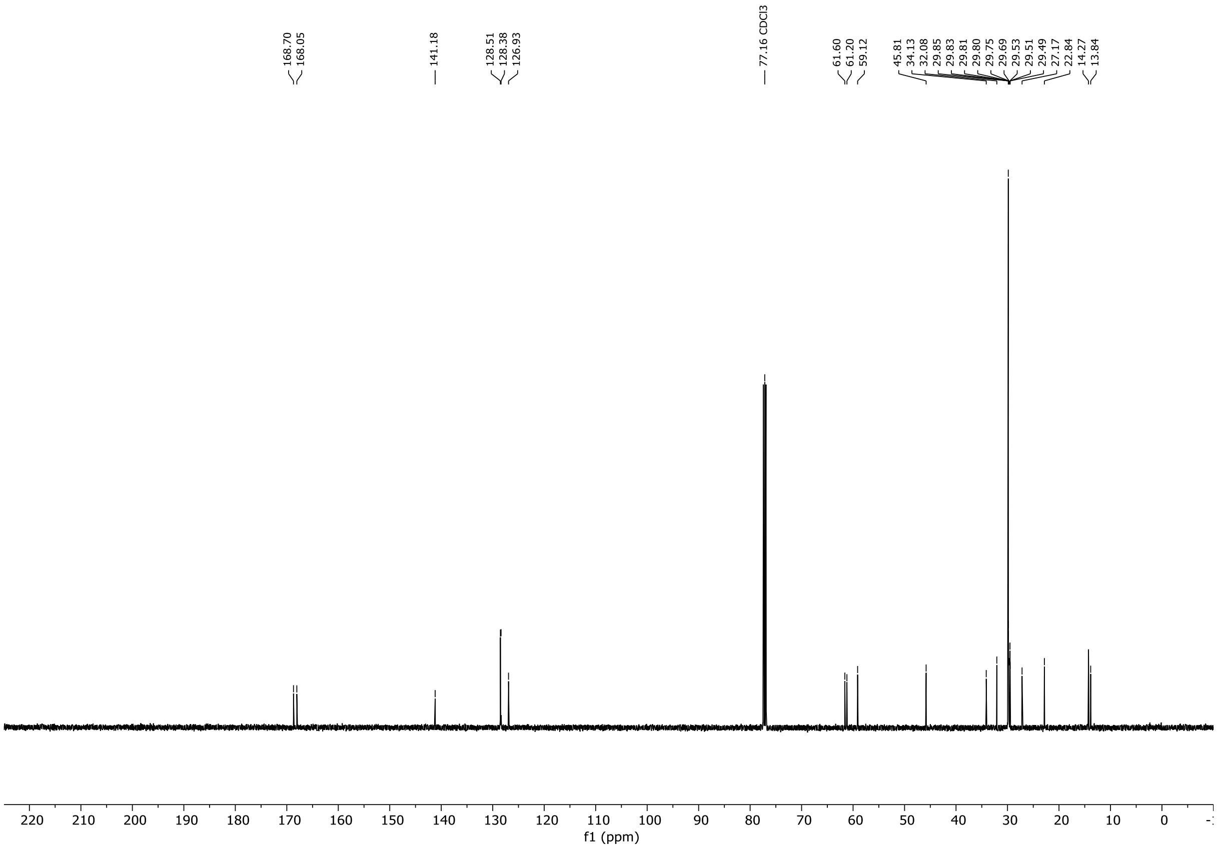

diethyl 2-(1-phenylicosyl)malonate, 3c

^{1}H NMR ($CDCl_3$, 500 MHz)

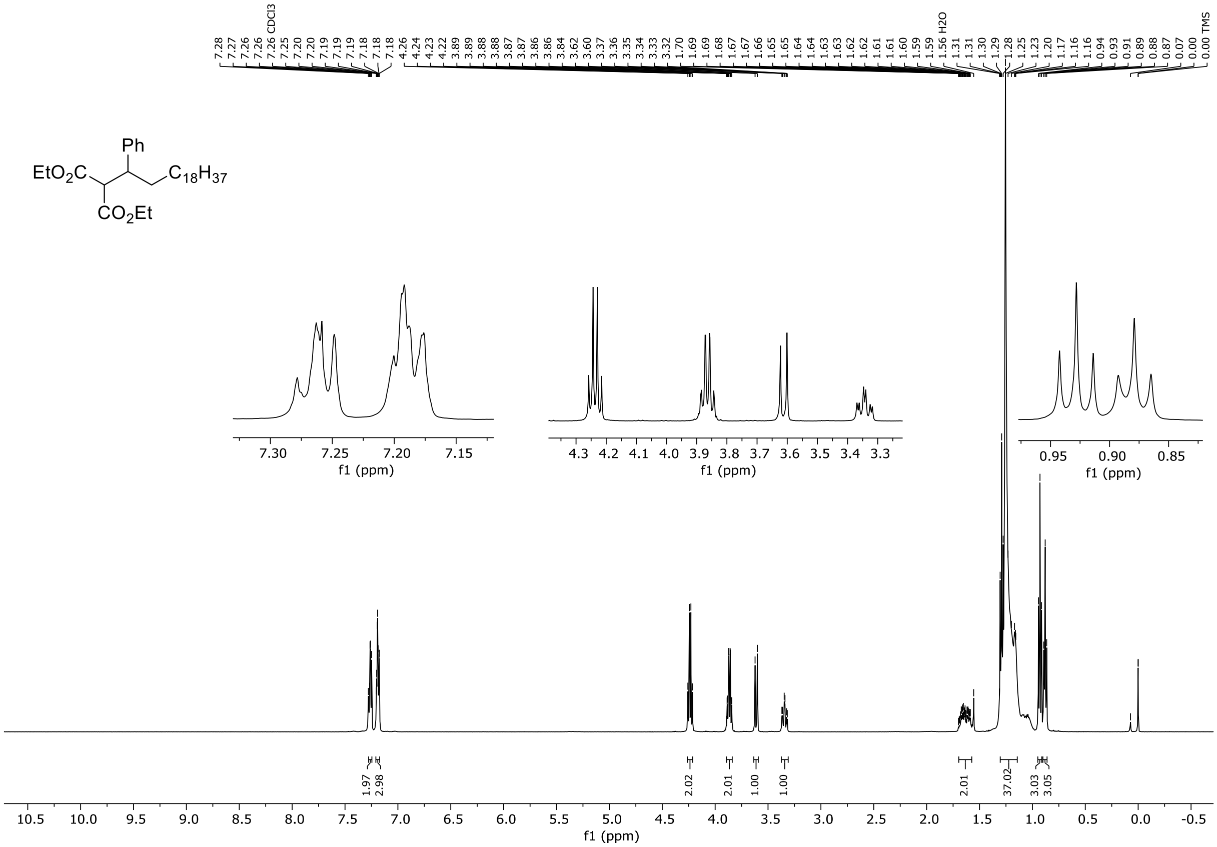

^{13}C NMR ($CDCl_3$, 126 MHz)

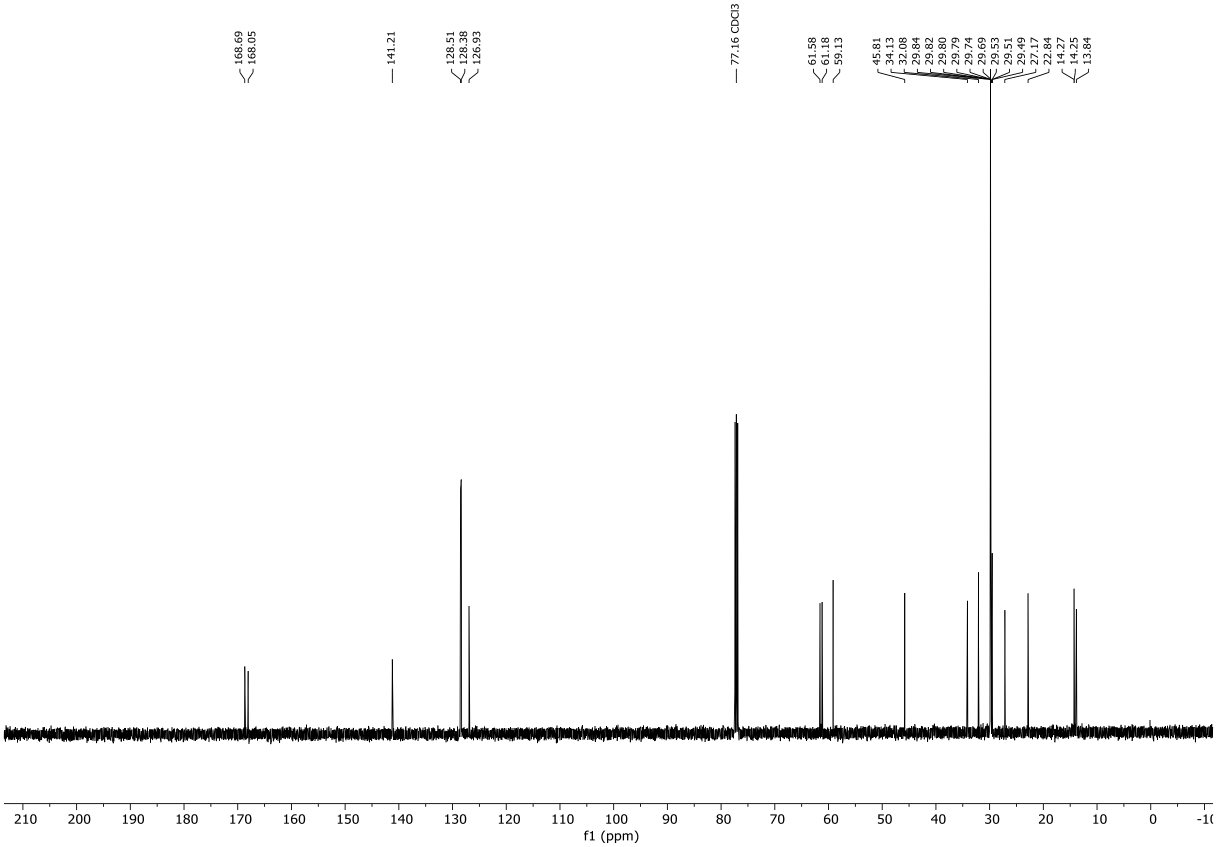

diethyl 2-(1-phenylheptadecyl)malonate, 3d

^{1}H NMR ($CDCl_3$, 500 MHz)

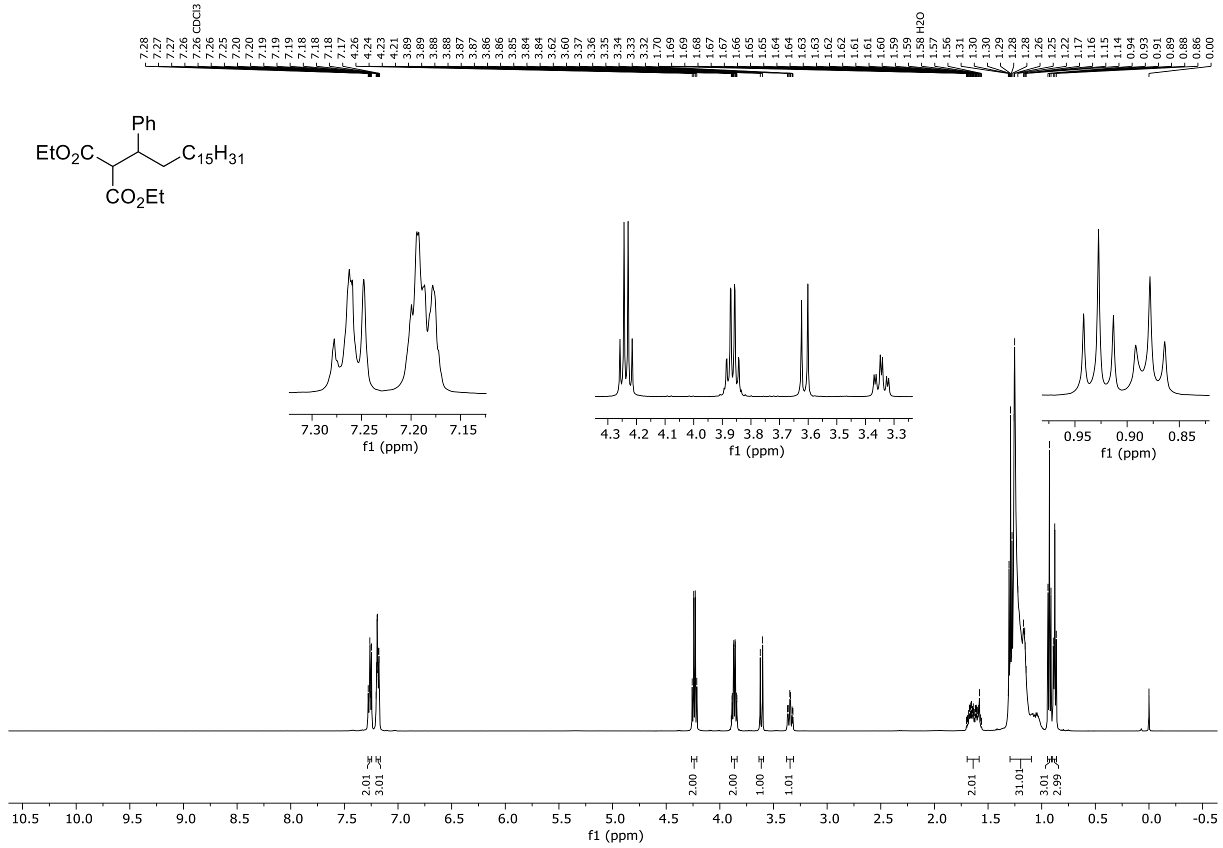

^{13}C NMR ($CDCl_3$, 126 MHz)

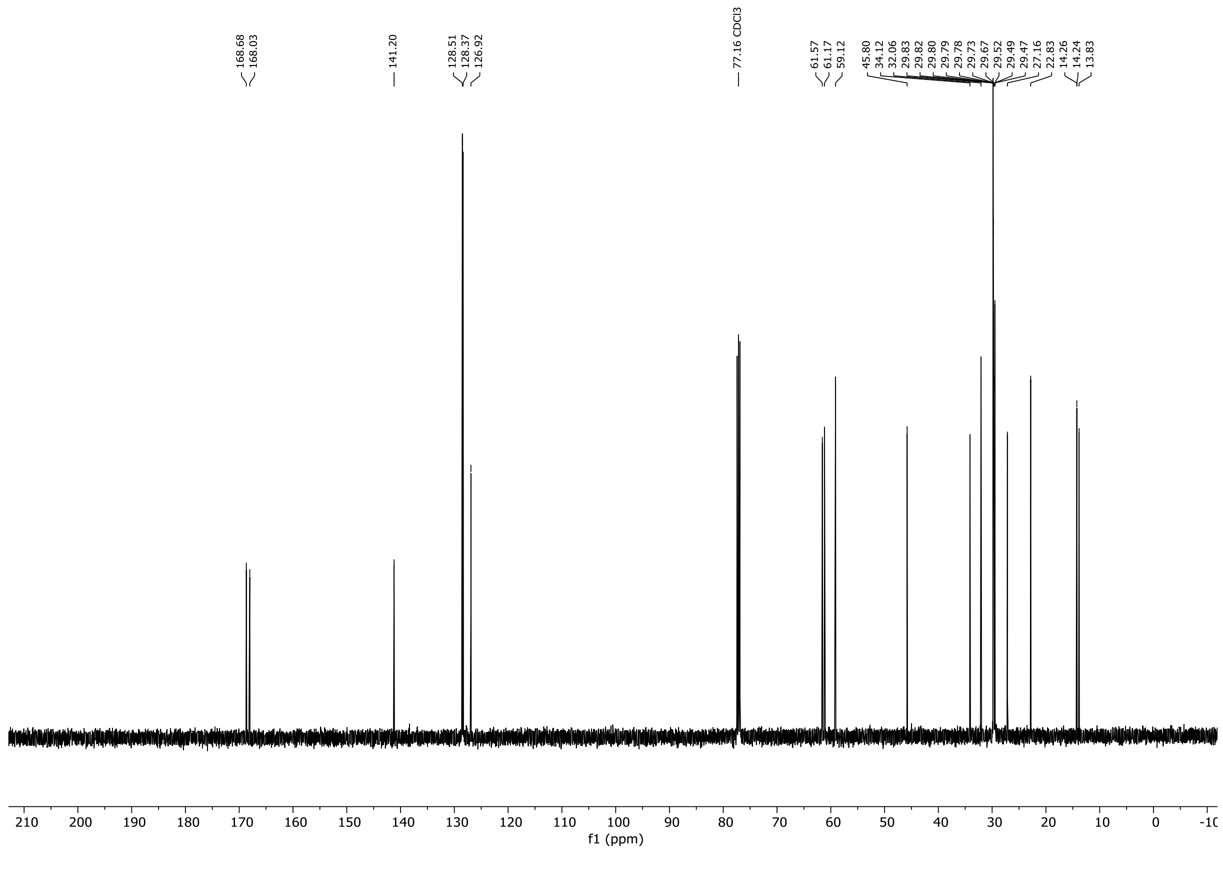

diethyl 2-(1-phenyltetradecyl)malonate, 3a

^{1}H NMR ($CDCl_3$, 600 MHz)

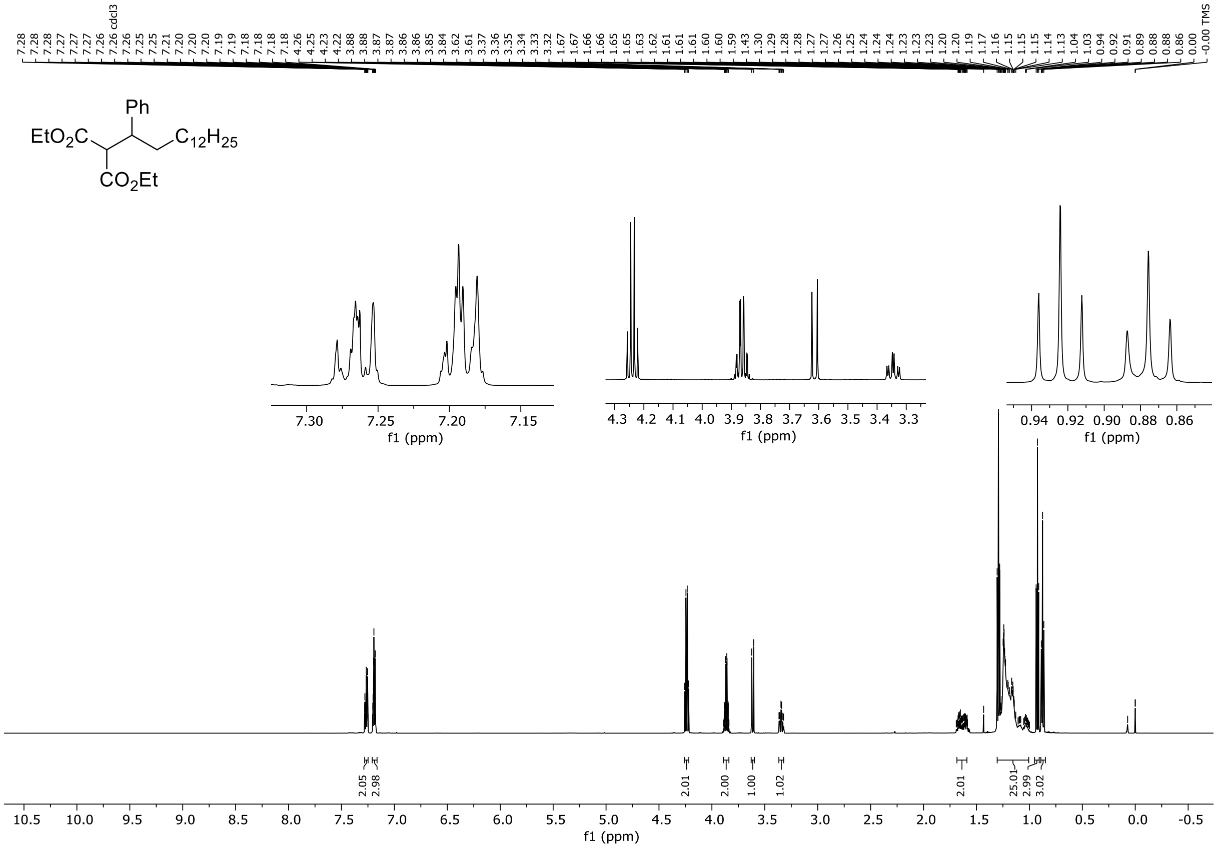

^{13}C NMR ($CDCl_3$, 126 MHz)

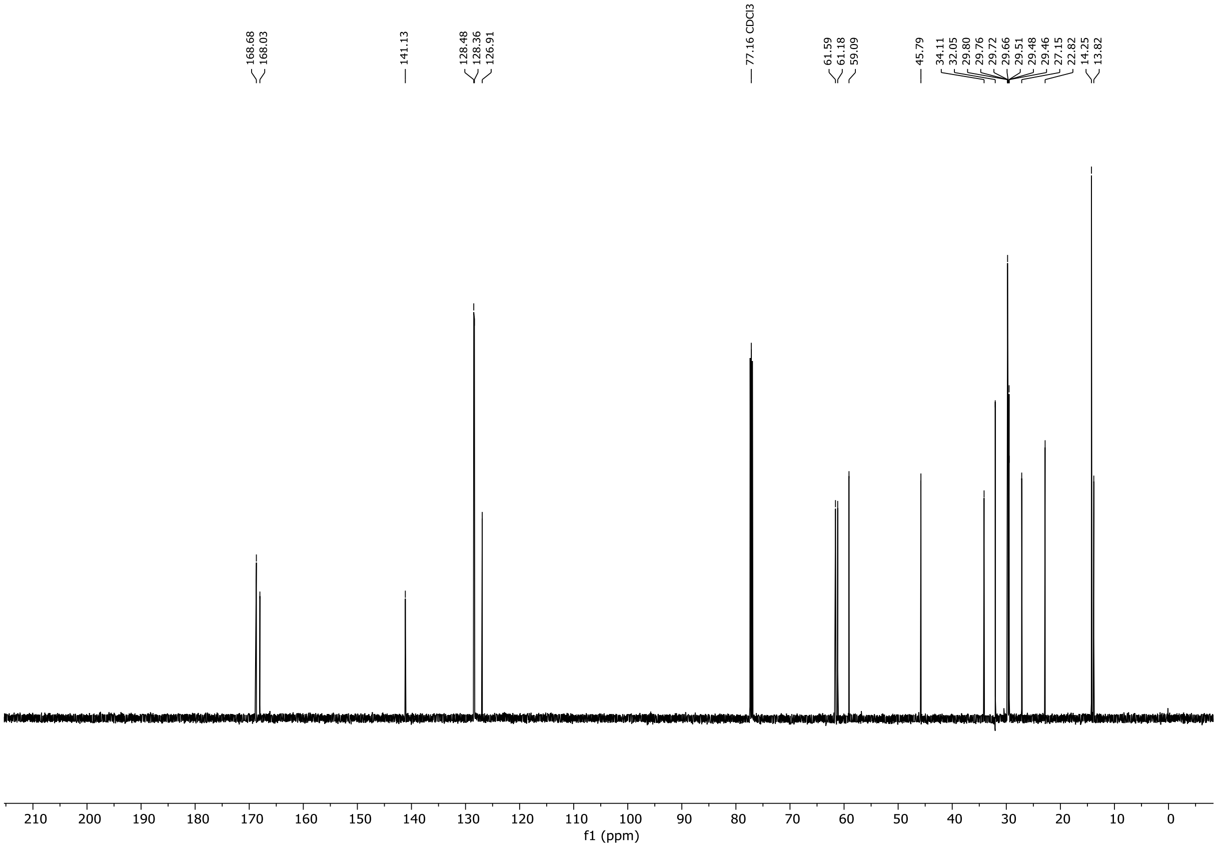

diethyl 2-(1-phenyldodecyl)malonate, 3e

^{1}H NMR ($CDCl_3$, 500 MHz)

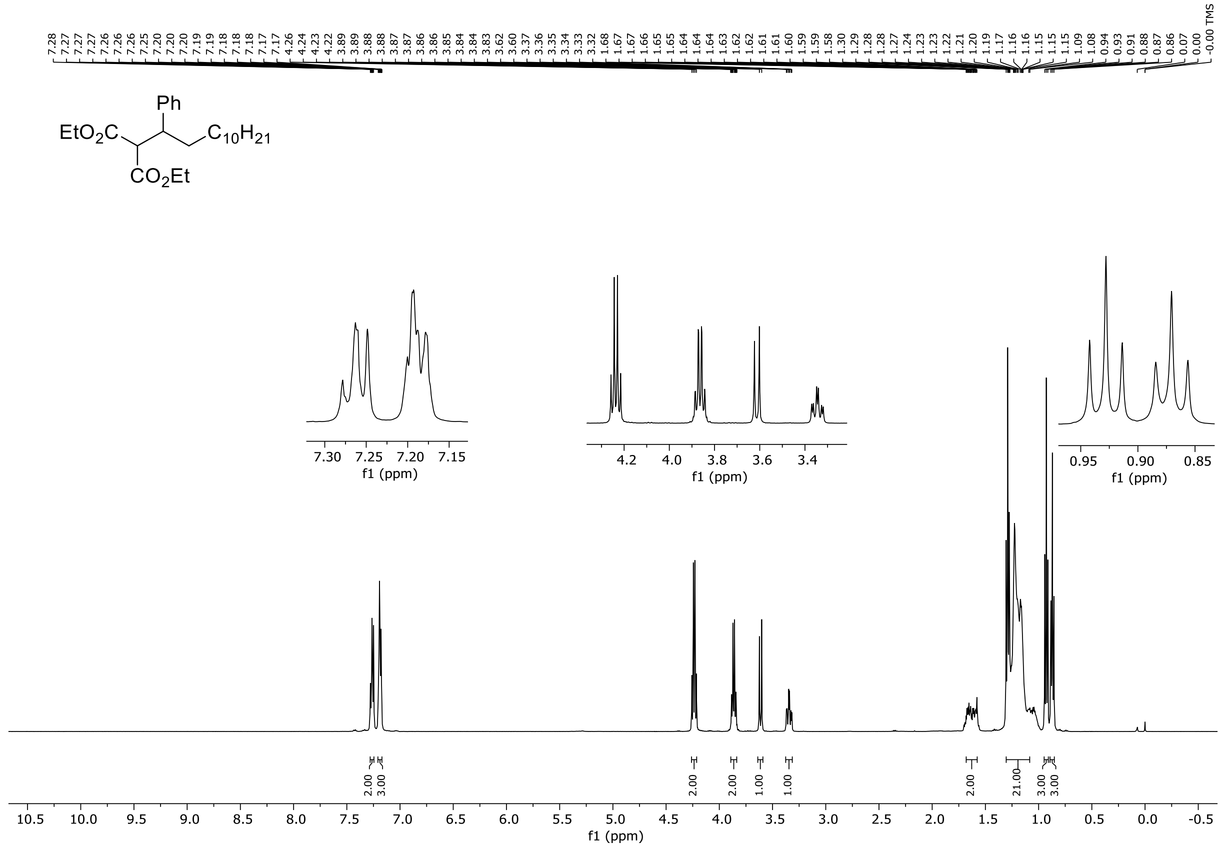

^{13}C NMR ($CDCl_3$, 126 MHz)

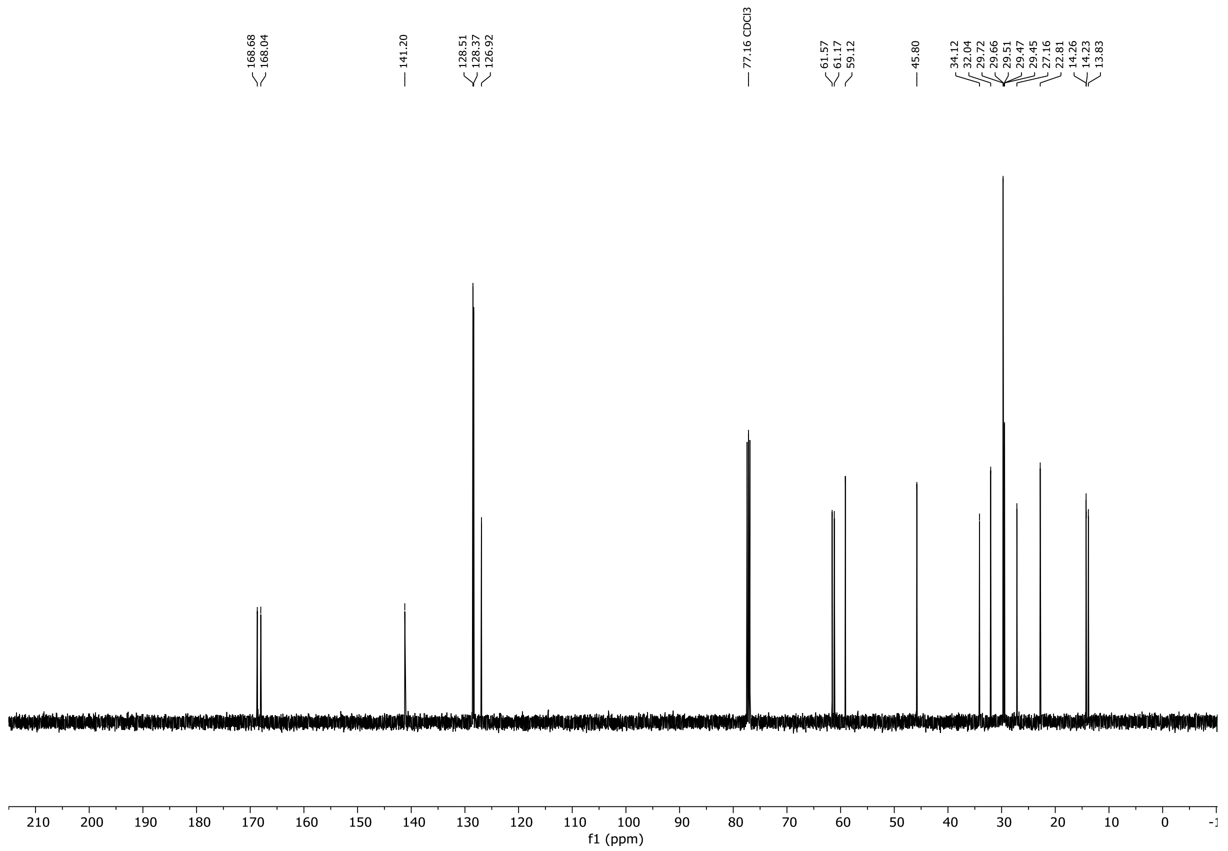

diethyl 2-(1-phenyldecyl)malonate, 3f

^{1}H NMR ($CDCl_3$, 500 MHz)

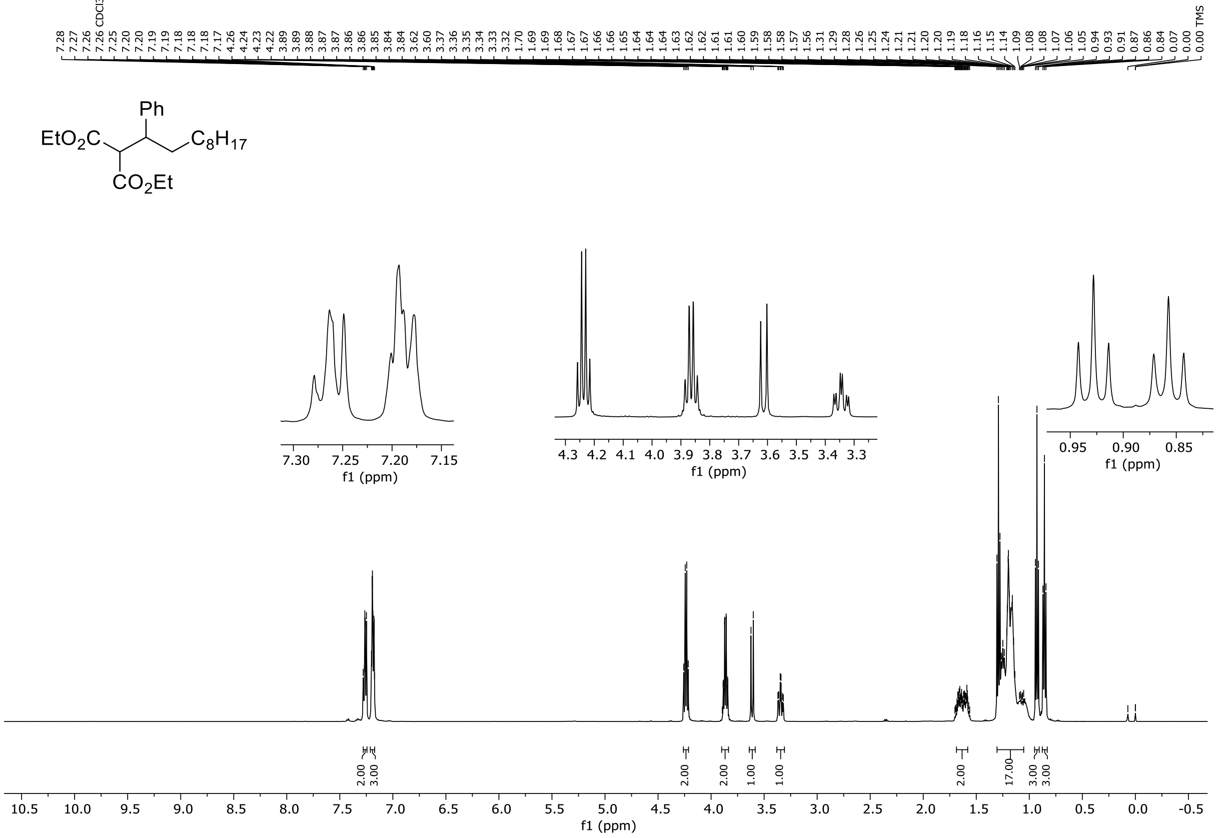

^{13}C NMR ($CDCl_3$, 126 MHz)

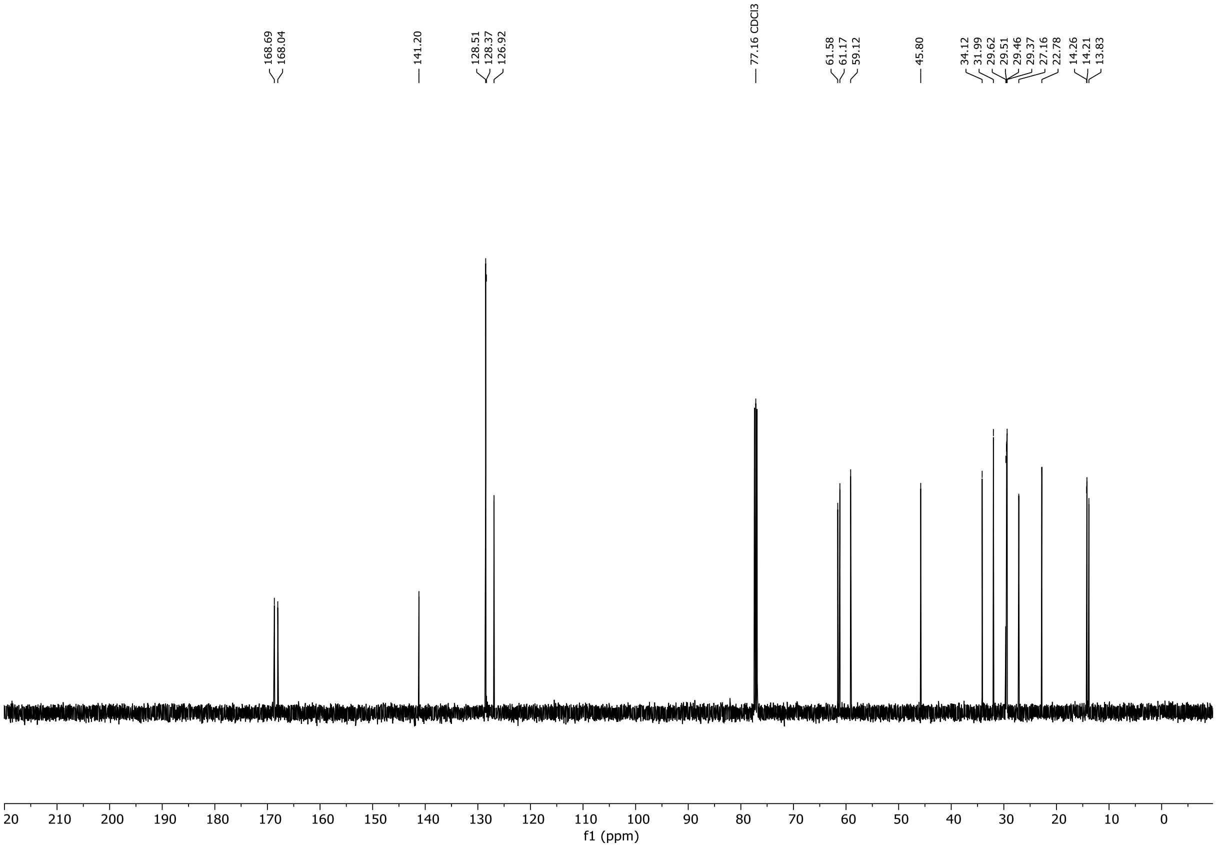

diethyl 2-(1-phenyloctyl)malonate, 3g

^{1}H NMR (CDCl3, 500 MHz)

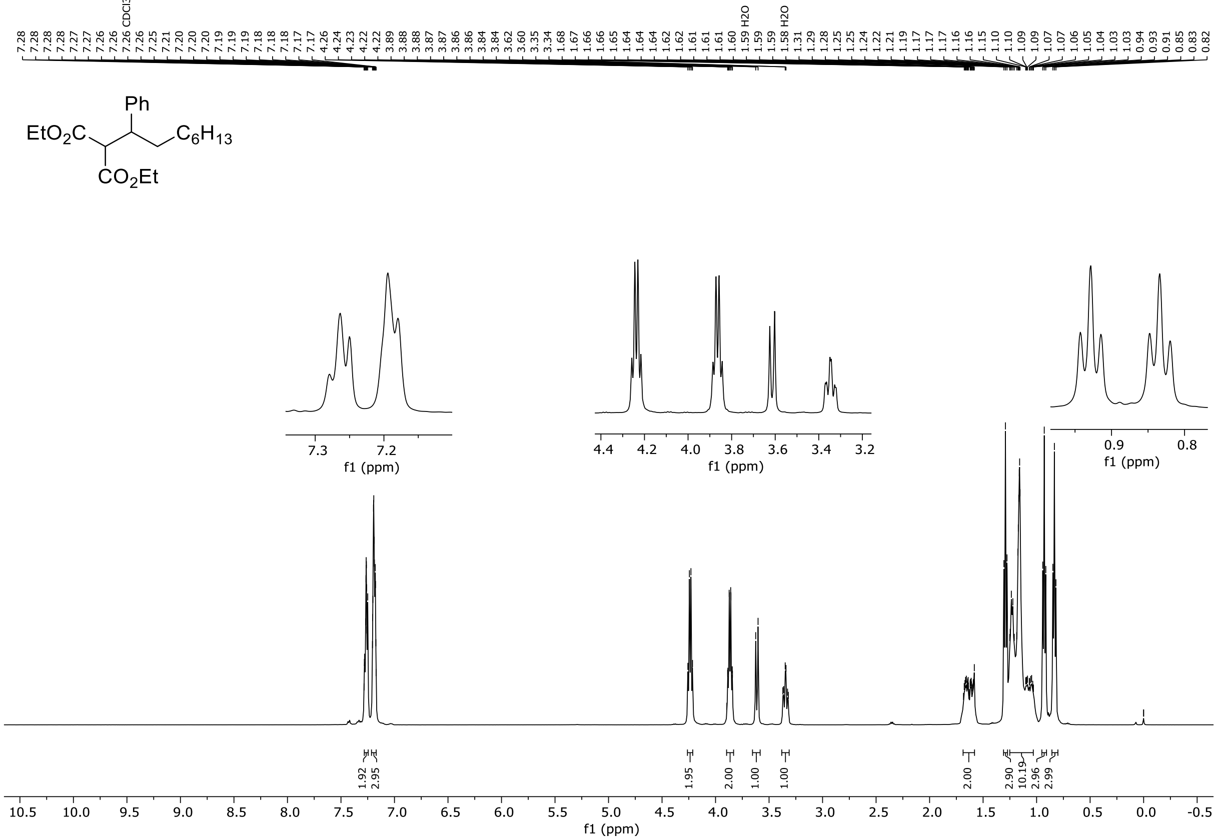

^{13}C NMR (CDCl3, 126 MHz)

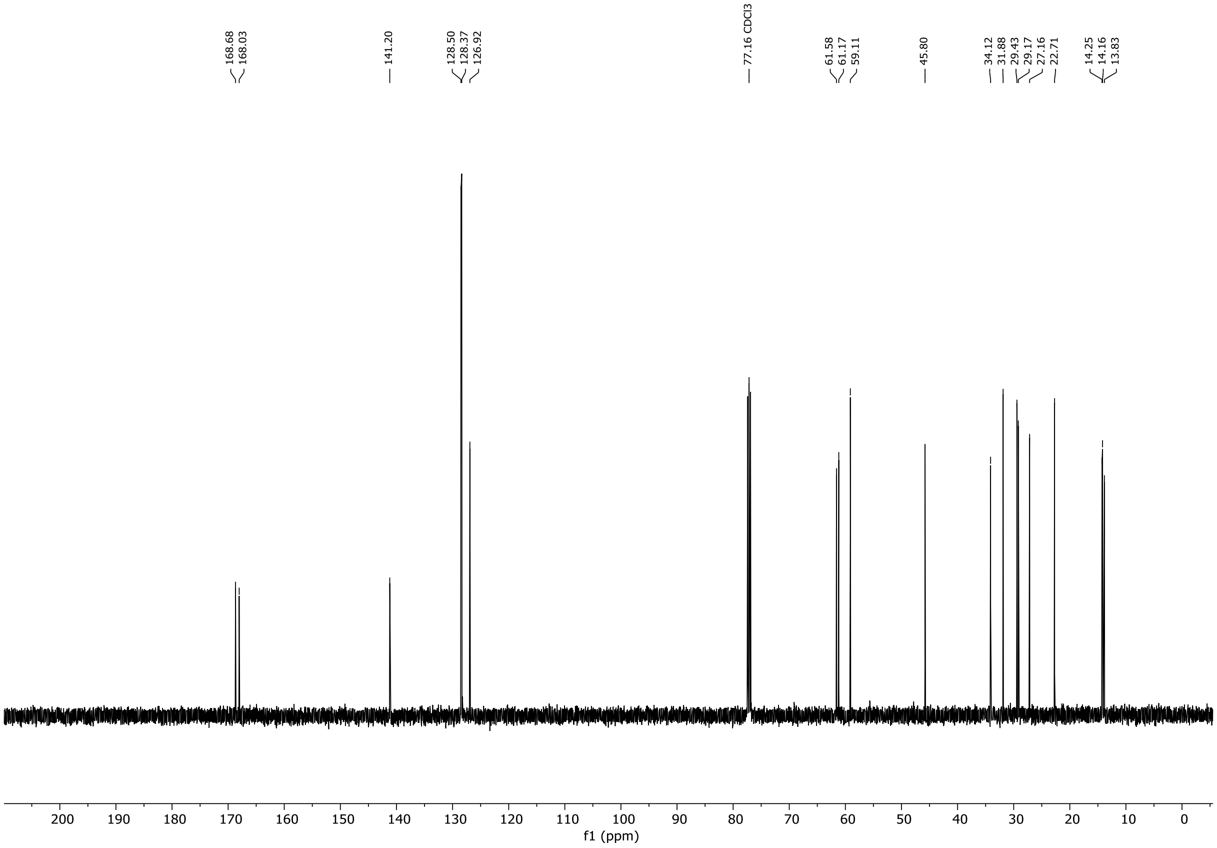

diethyl 2-(1-phenylhexyl)malonate, 3h

^{1}H NMR ($CDCl_3$, 600 MHz)

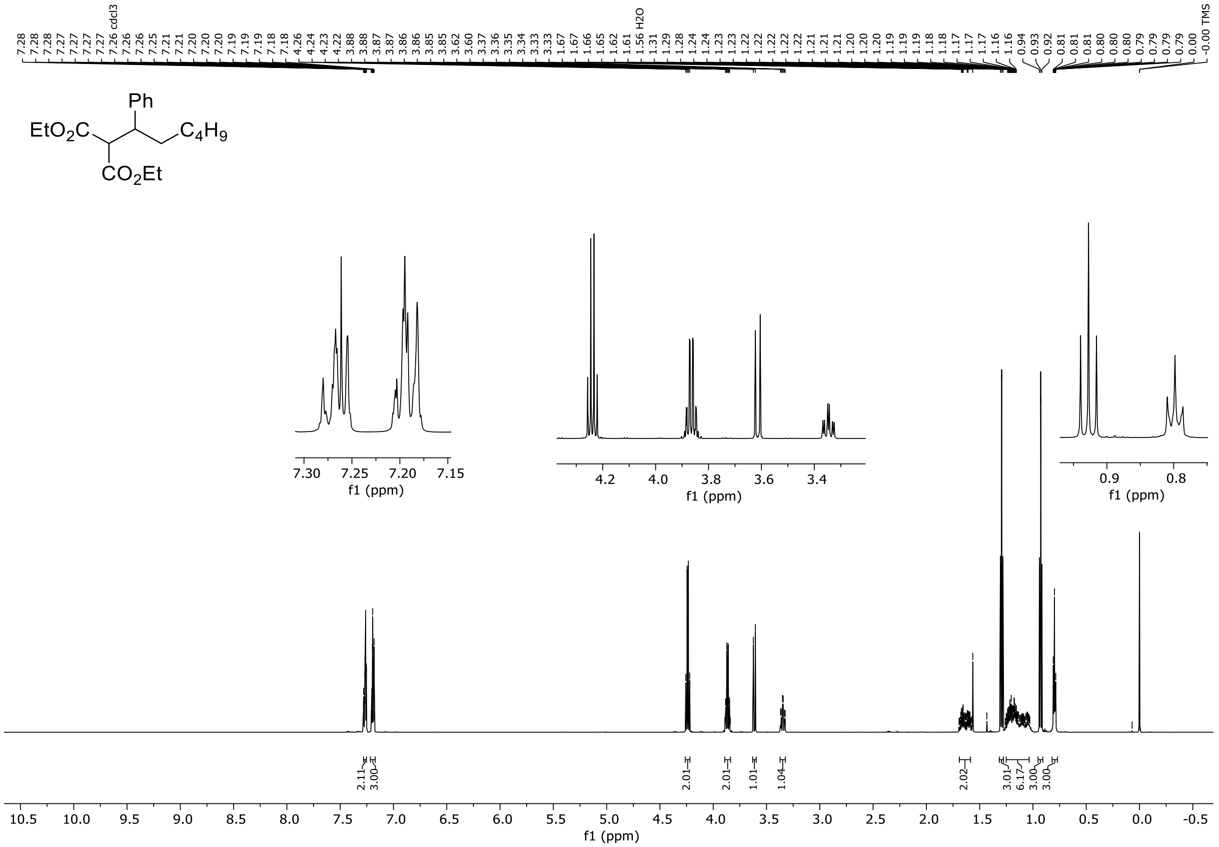

^{13}C NMR ($CDCl_3$, 126 MHz)

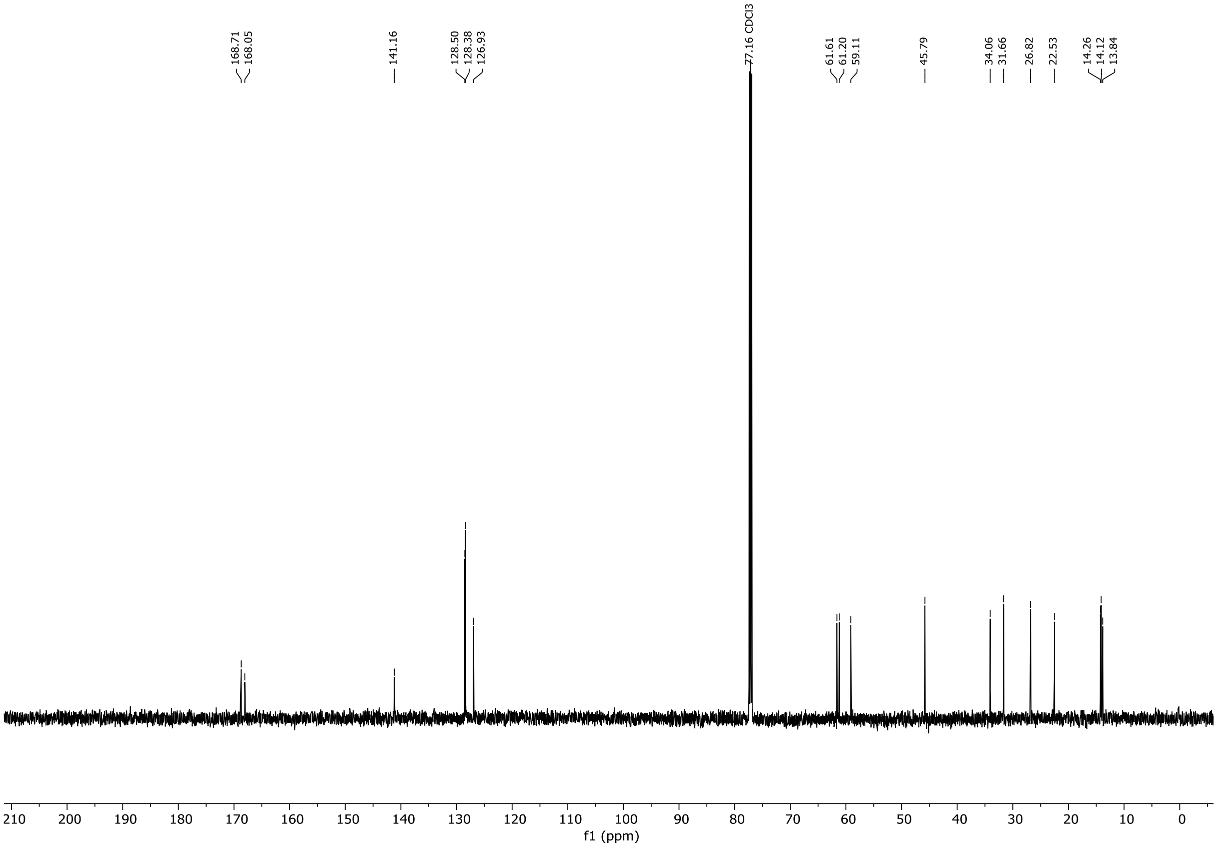

diethyl 2-(1-phenylbutyl)malonate, 3i

^{1}H NMR ($CDCl_3$, 500 MHz)

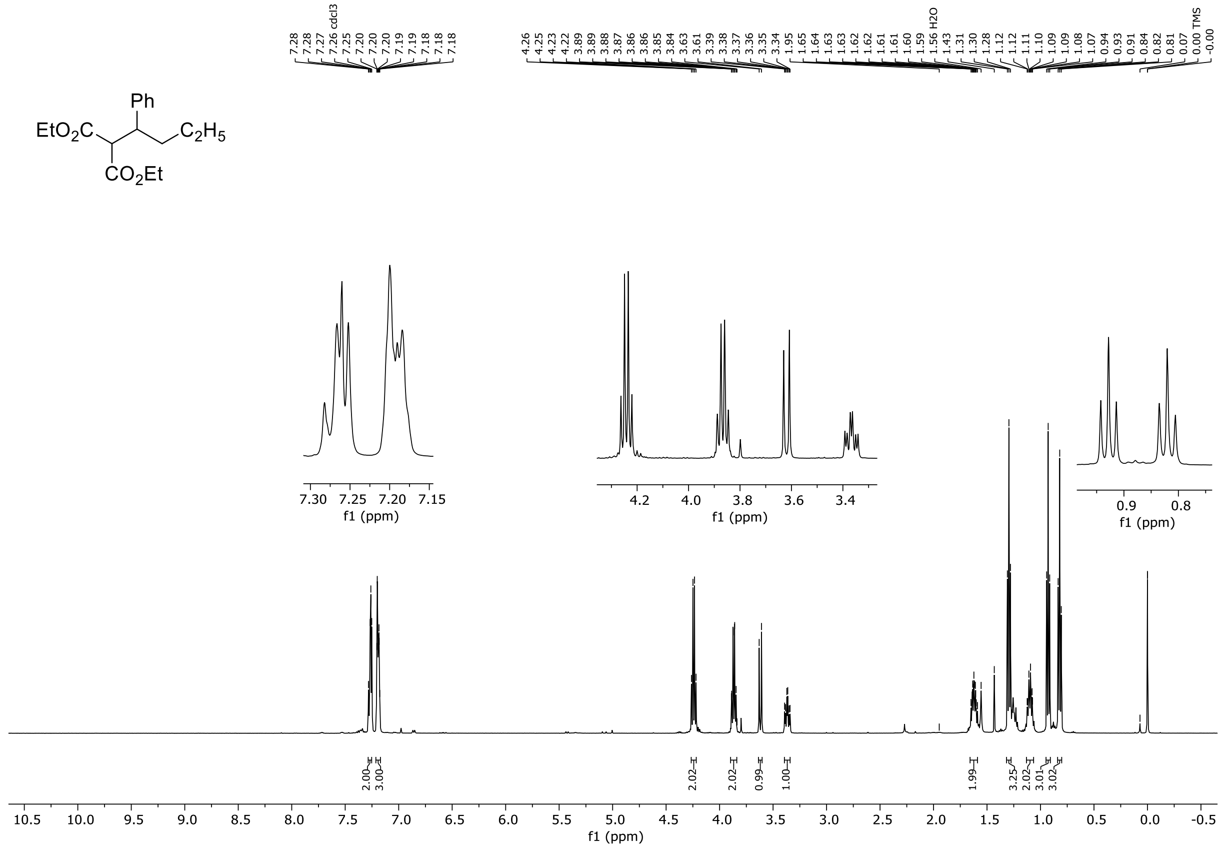

^{13}C NMR ($CDCl_3$, 126 MHz)

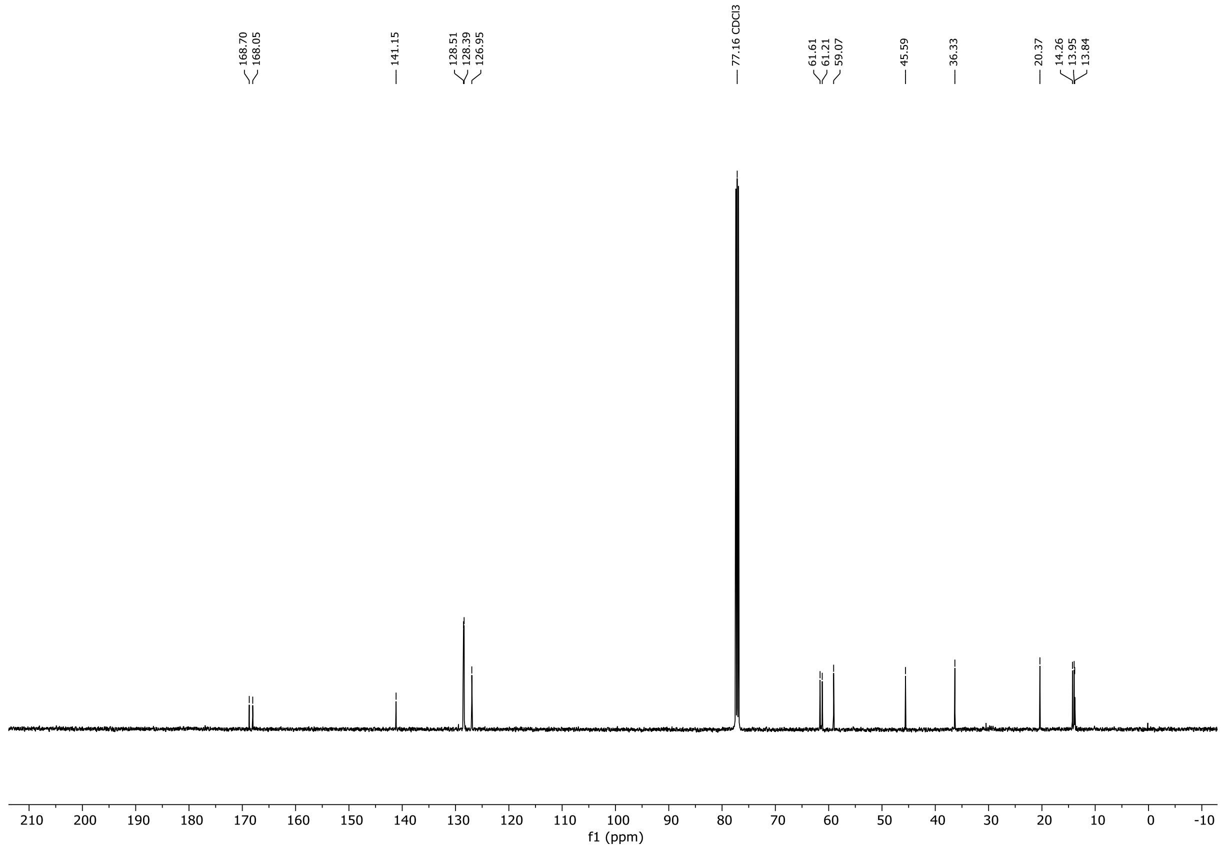

diethyl 2-(3-cyclohexyl-1-phenylpropyl)malonate, 4

^{1}H NMR (CDCl$_3$, 500 MHz)

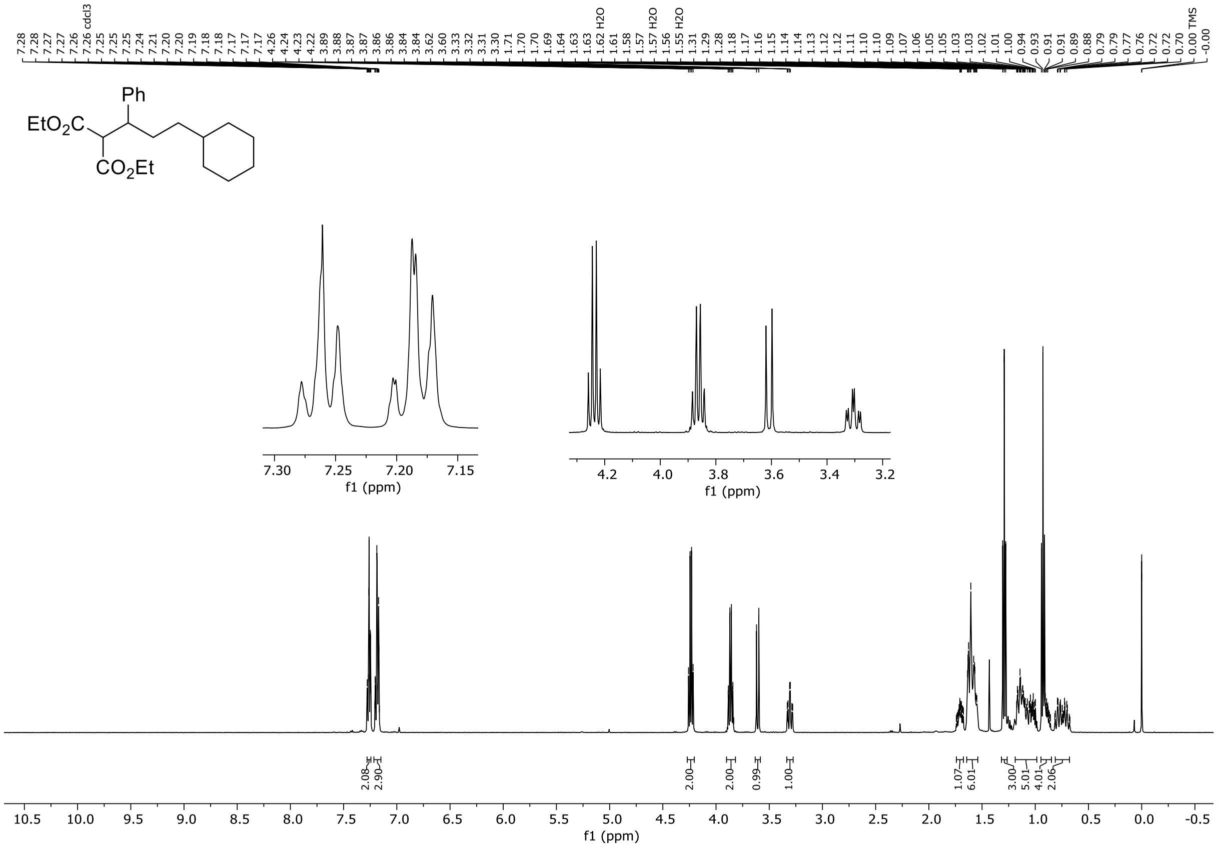

^{13}C NMR (CDCl$_3$, 126 MHz)

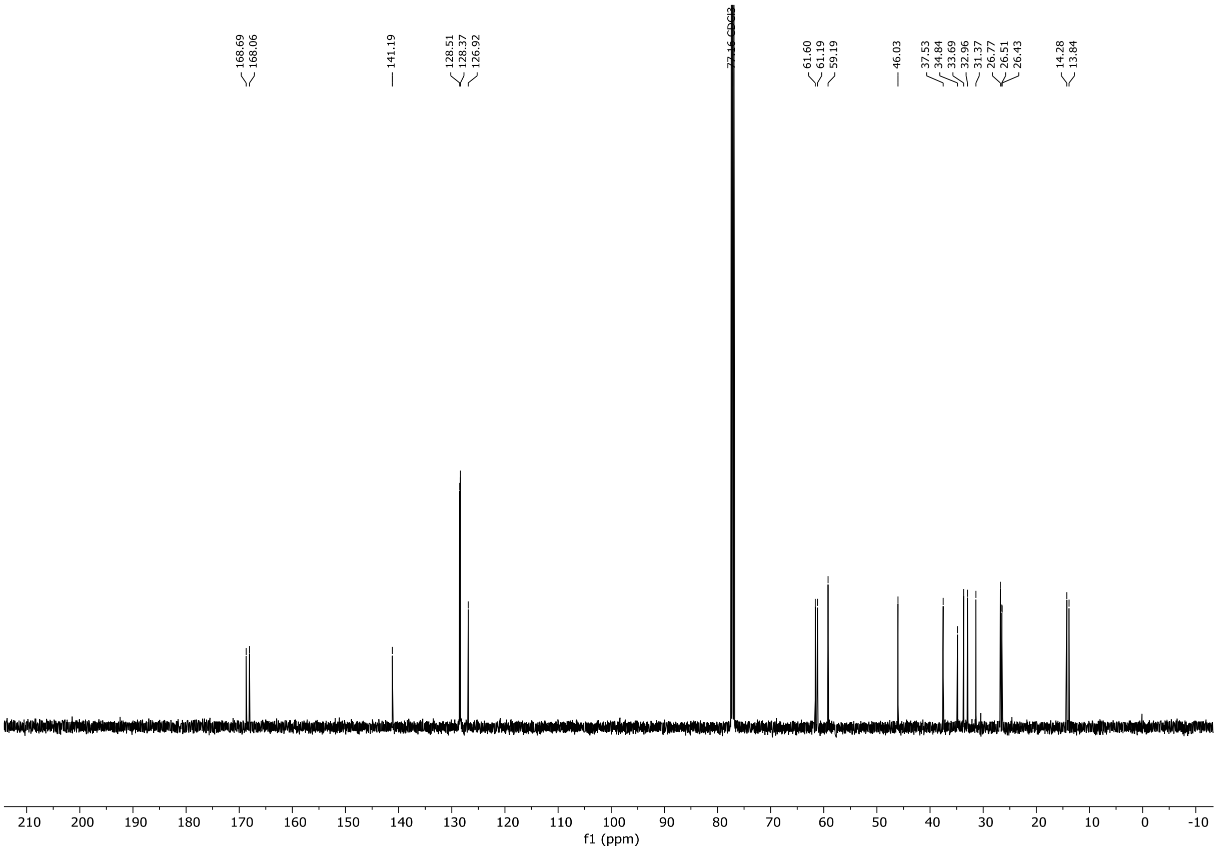

diethyl 2-(4,4-dimethyl-1-phenylpentyl)malonate, 5

^{1}H NMR ($CDCl_3$, 500 MHz)

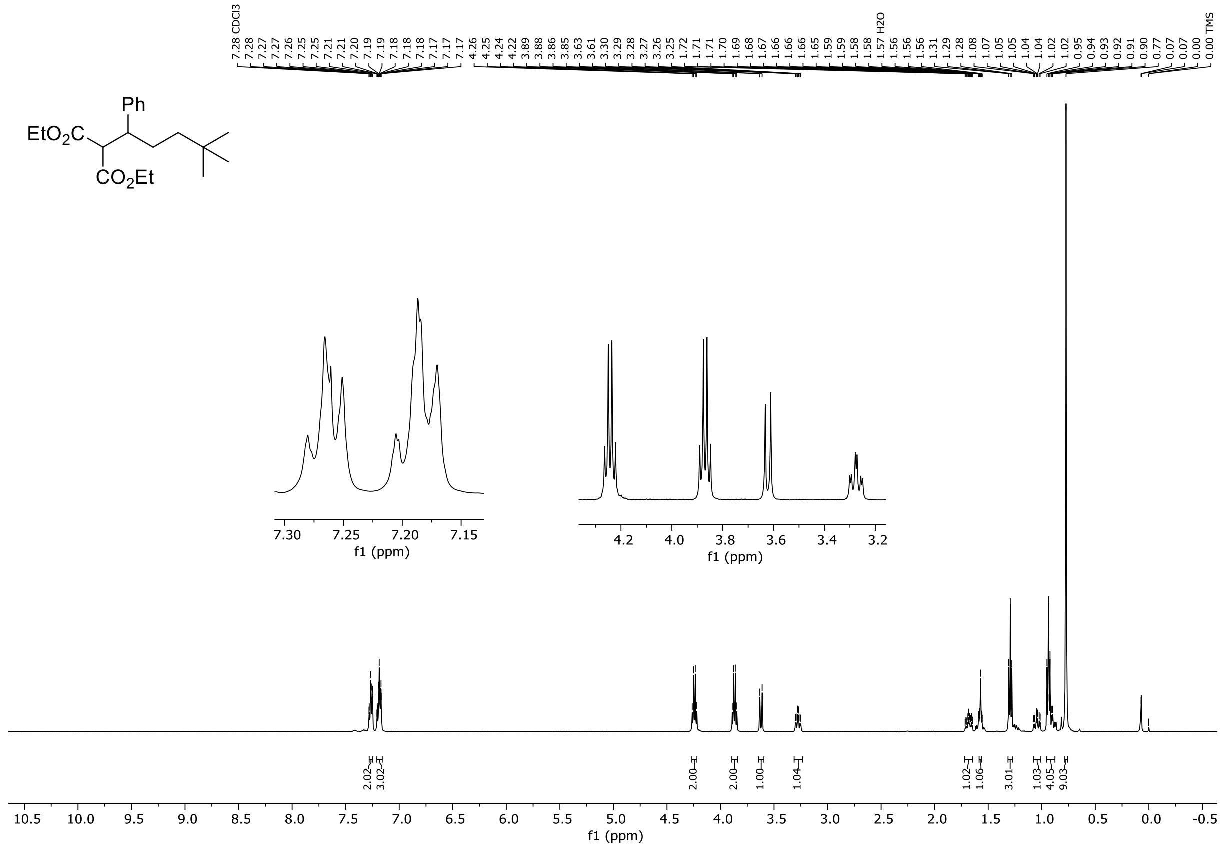

^{13}C NMR ($CDCl_3$, 126 MHz)

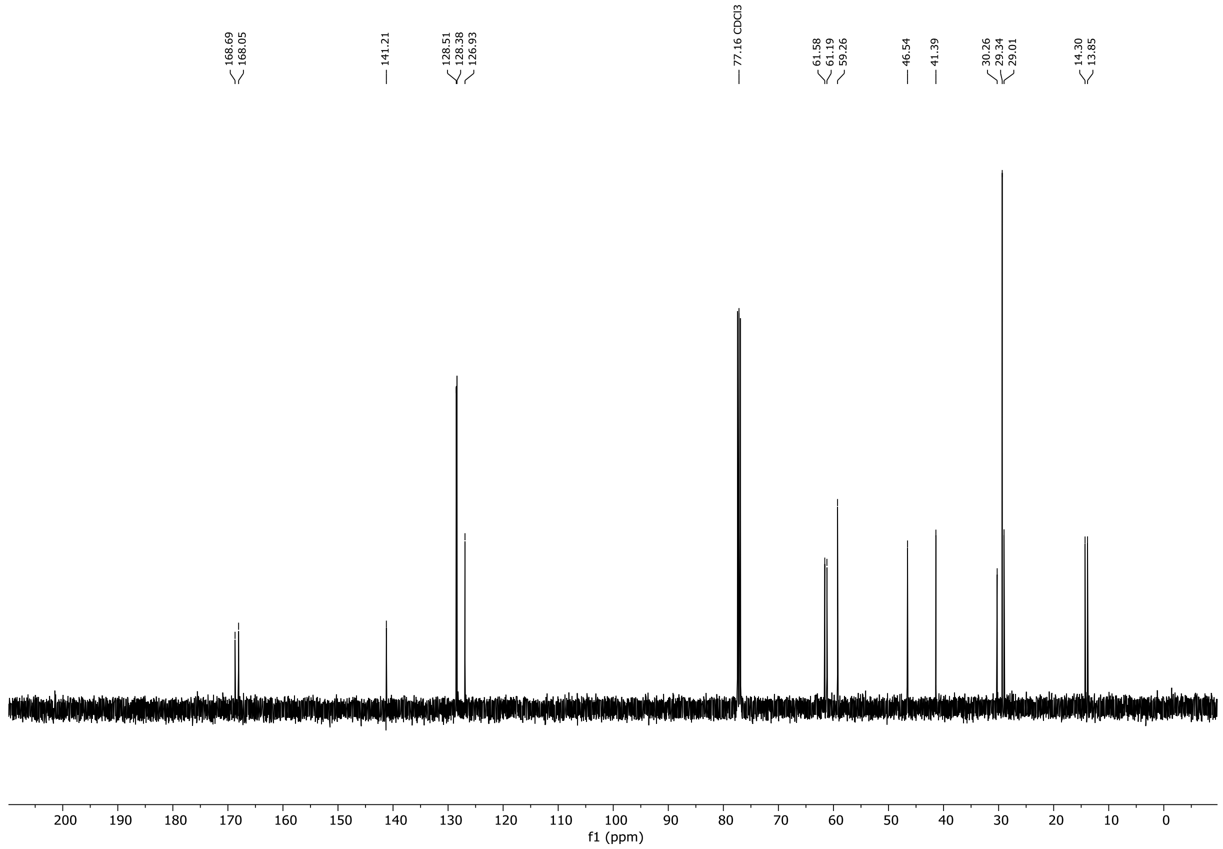

diethyl 2-(3-methyl-1-phenylhexyl)malonate, 6

^{1}H NMR (CDCl3, 500 MHz)

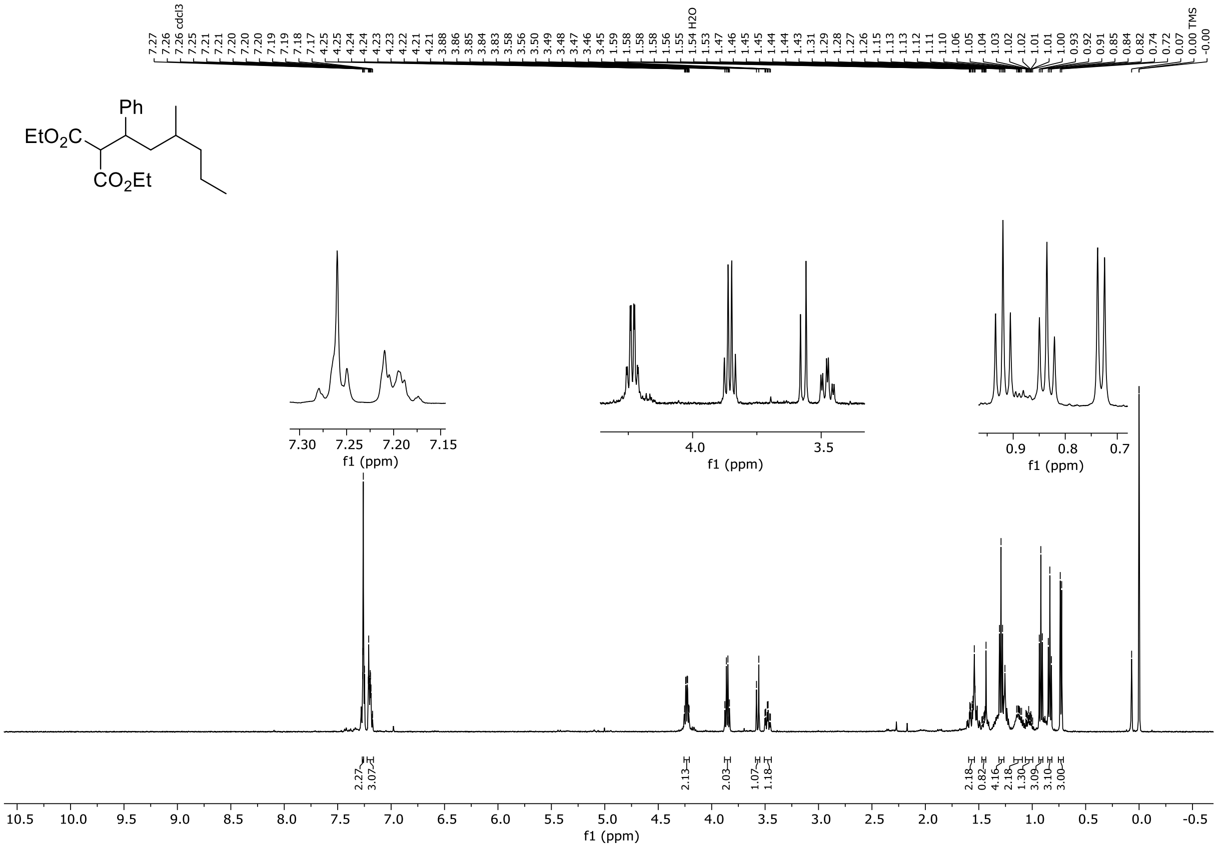

^{13}C NMR (CDCl3, 126 MHz)

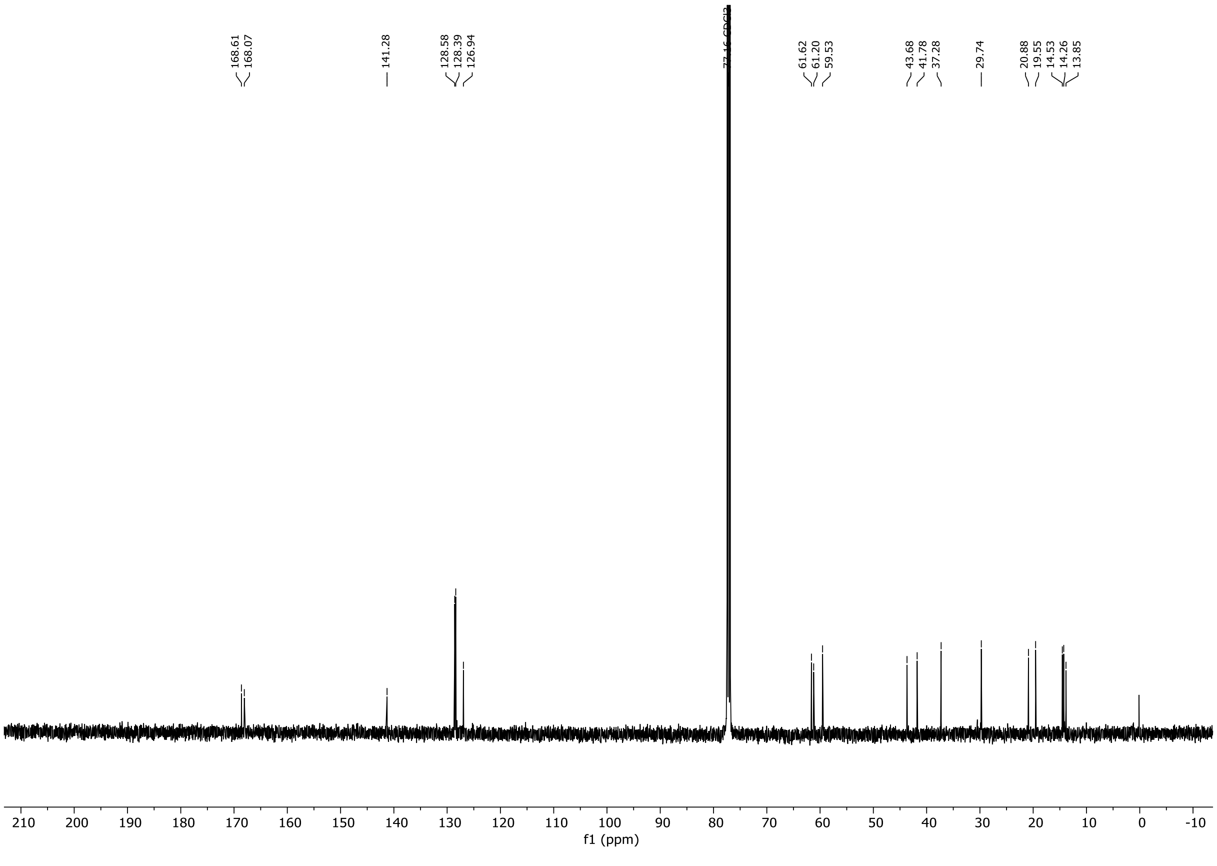

diethyl 2-(2-cyclohexyl-1-phenylethyl)malonate, 7

^{1}H NMR ($CDCl_3$, 500 MHz)

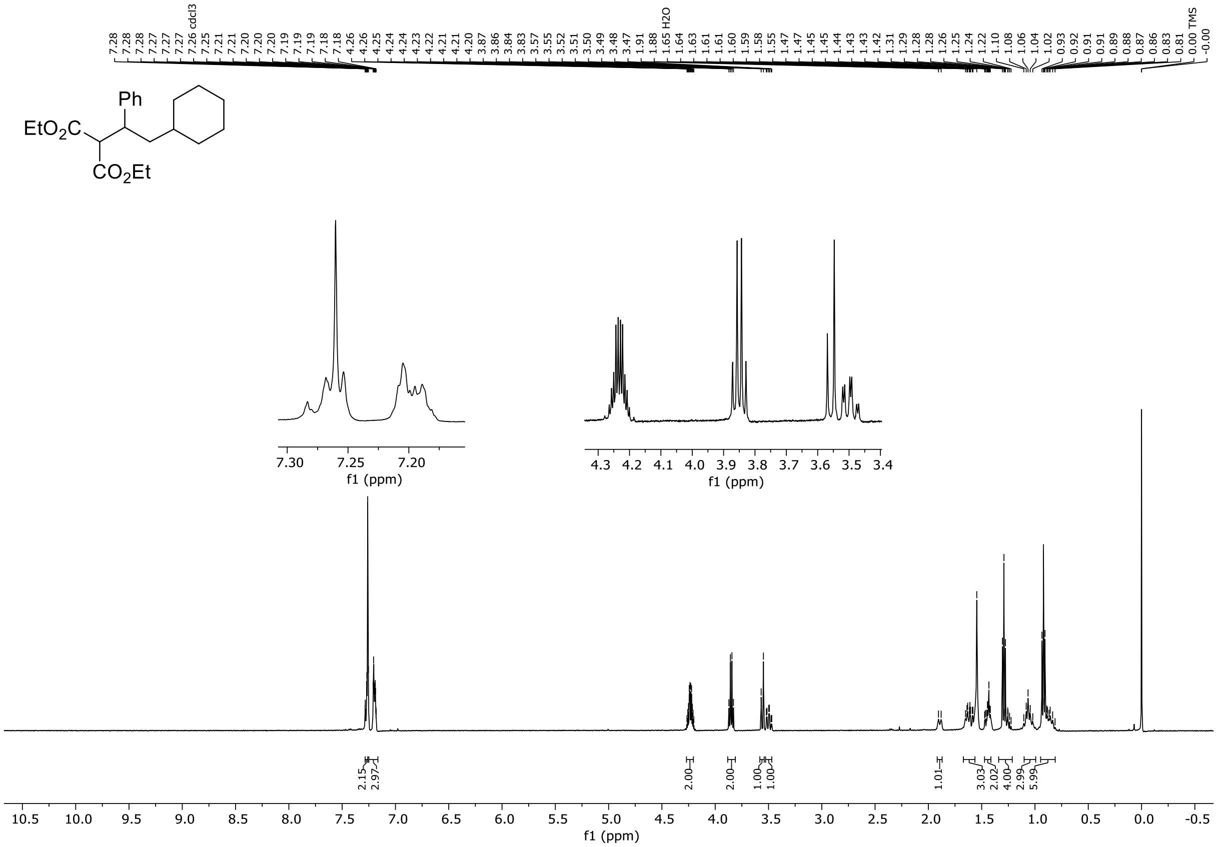

^{13}C NMR ($CDCl_3$, 126 MHz)

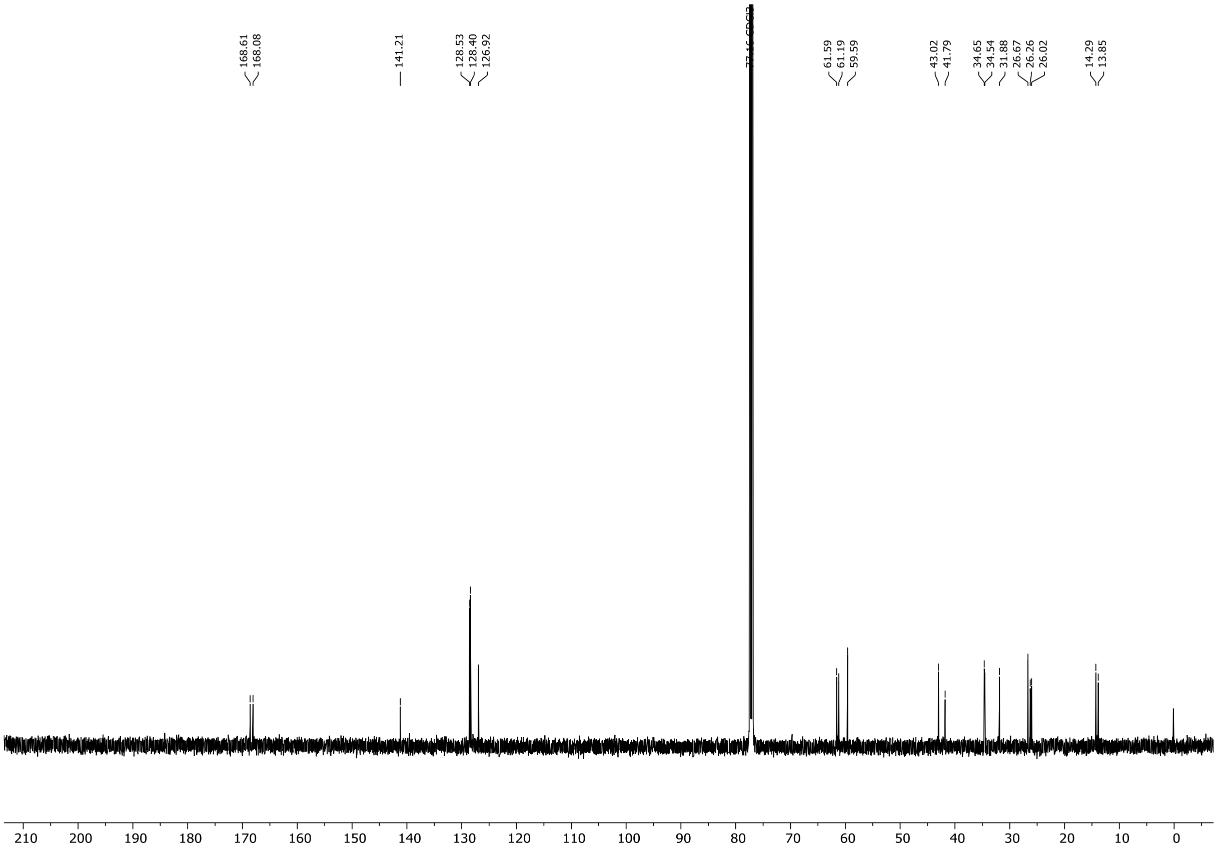

diethyl 2-(1,10-diphenyldecyl)malonate, 9

^{1}H NMR ($CDCl_3$, 500 MHz)

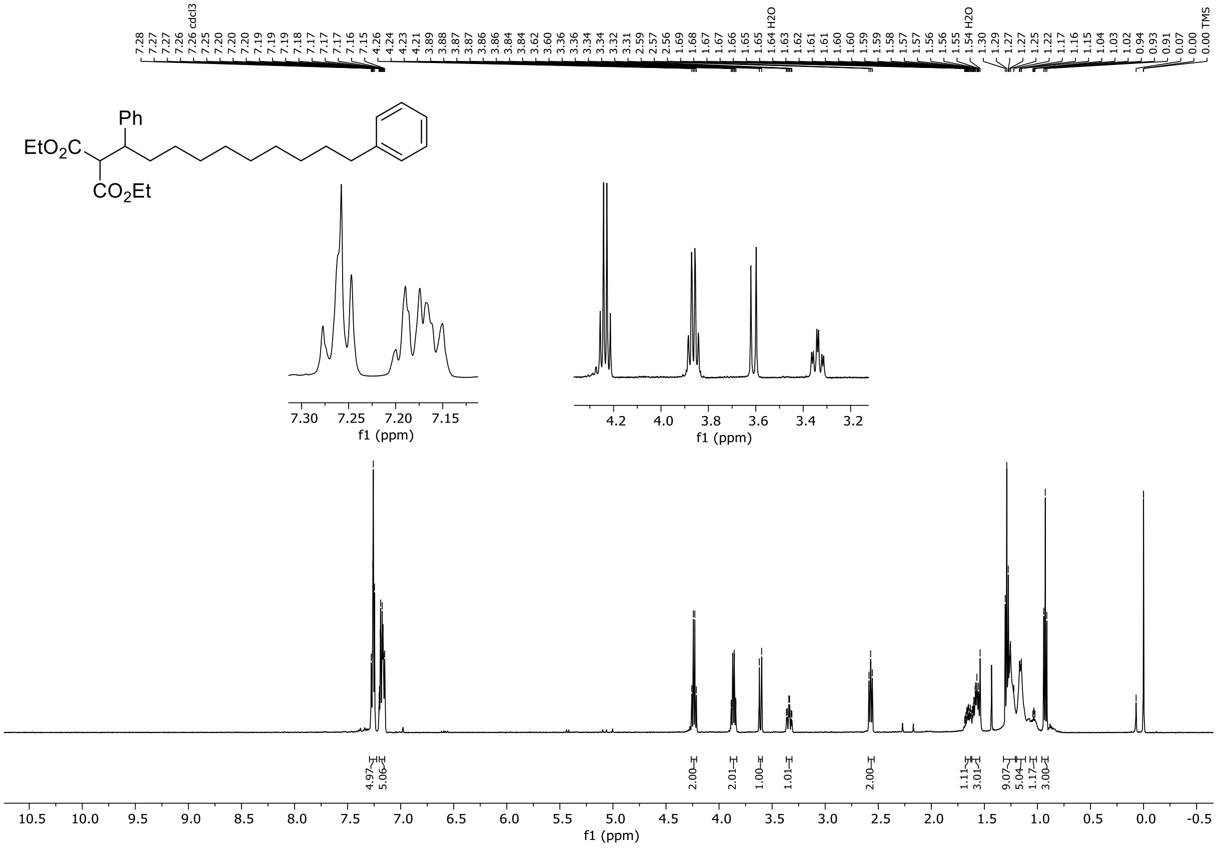

^{13}C NMR ($CDCl_3$, 126 MHz)

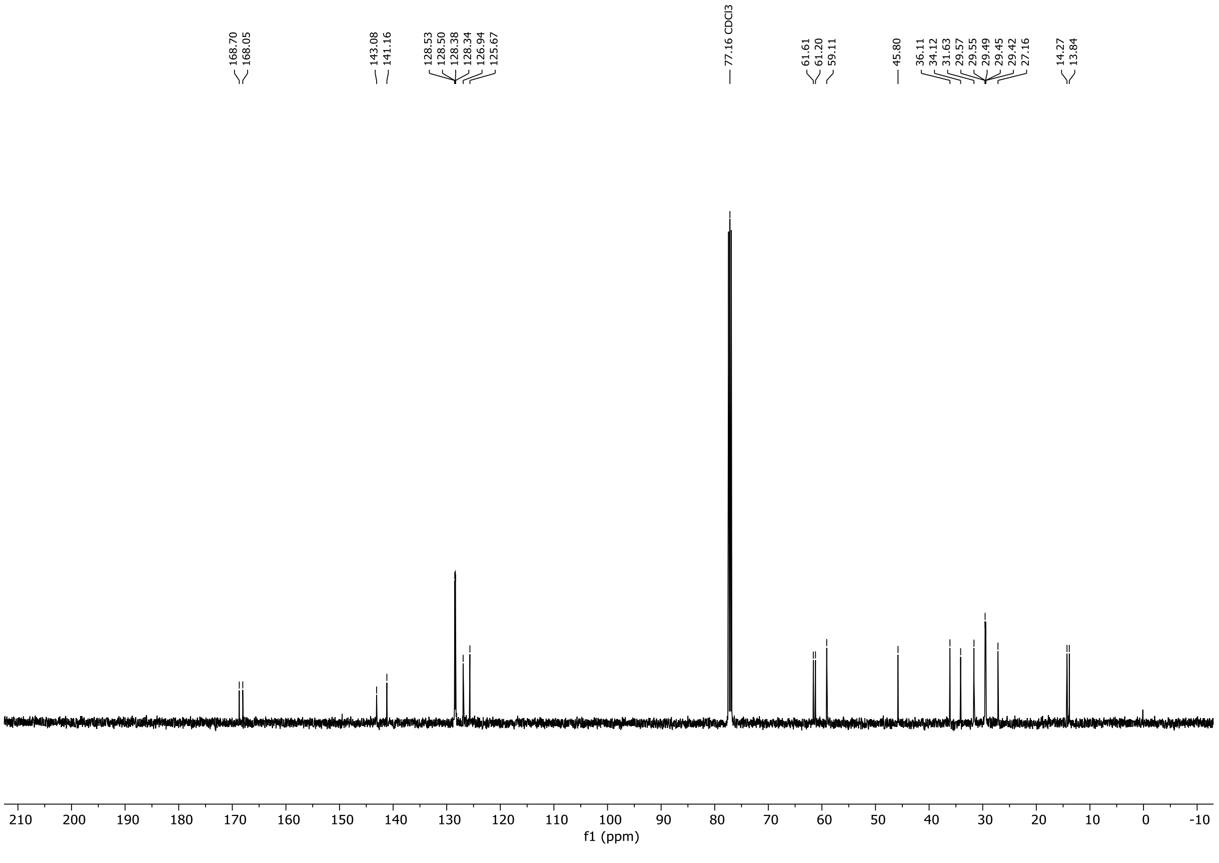

4-dodecyl 1,1-diethyl 2-phenylbutane-1,1,4-tricarboxylate, 10a

^{1}H NMR ($CDCl_3$, 500 MHz)

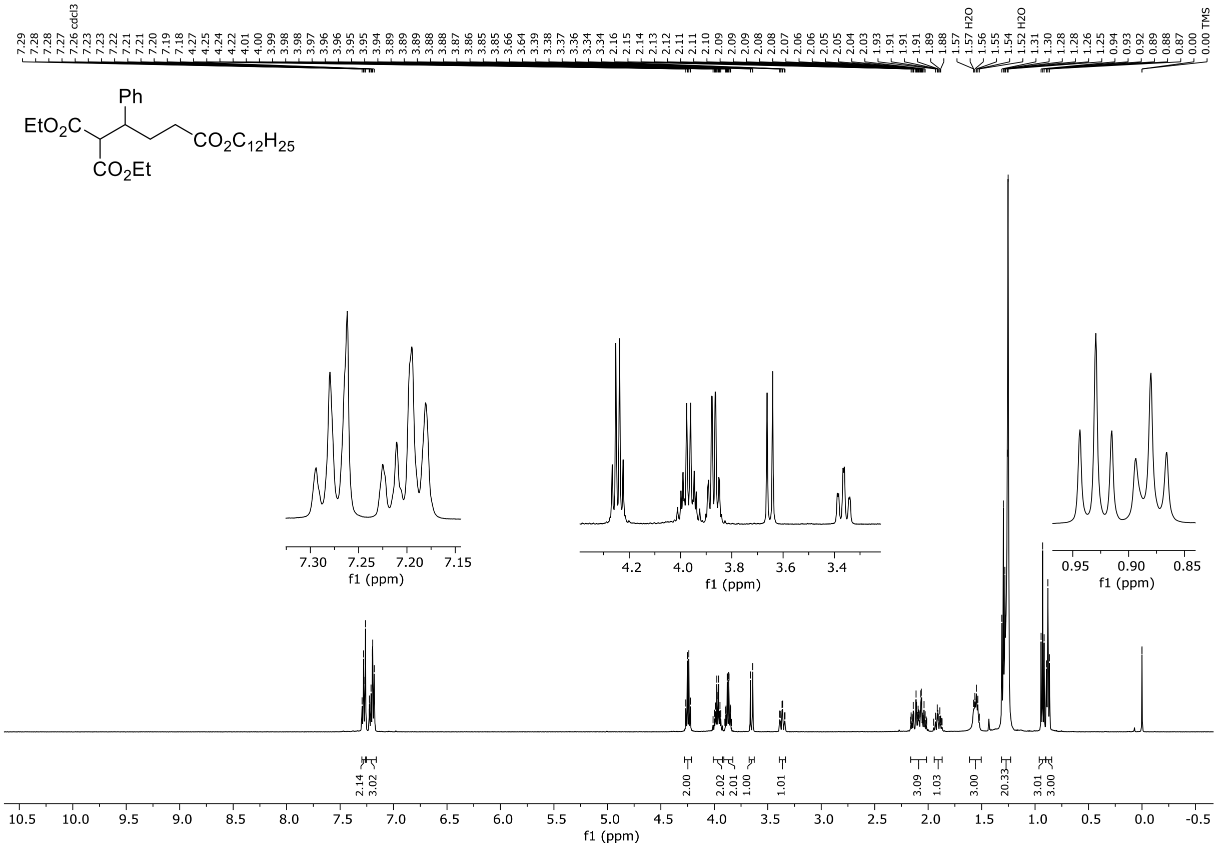

^{13}C NMR ($CDCl_3$, 126 MHz)

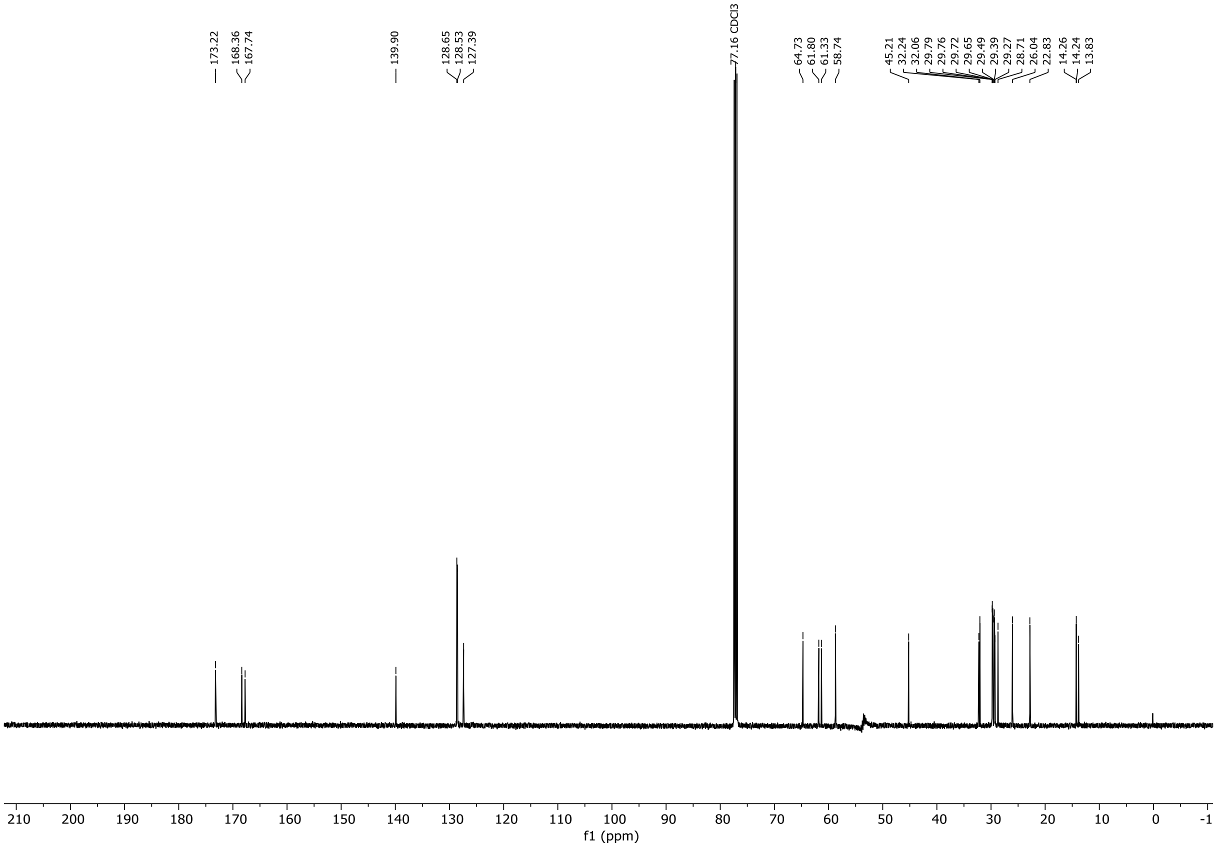

4-dodecyl 1,1-diethyl 1-phenylbutane-1,1,4-tricarboxylate, 10b

^{1}H NMR ($CDCl_3$, 500 MHz)

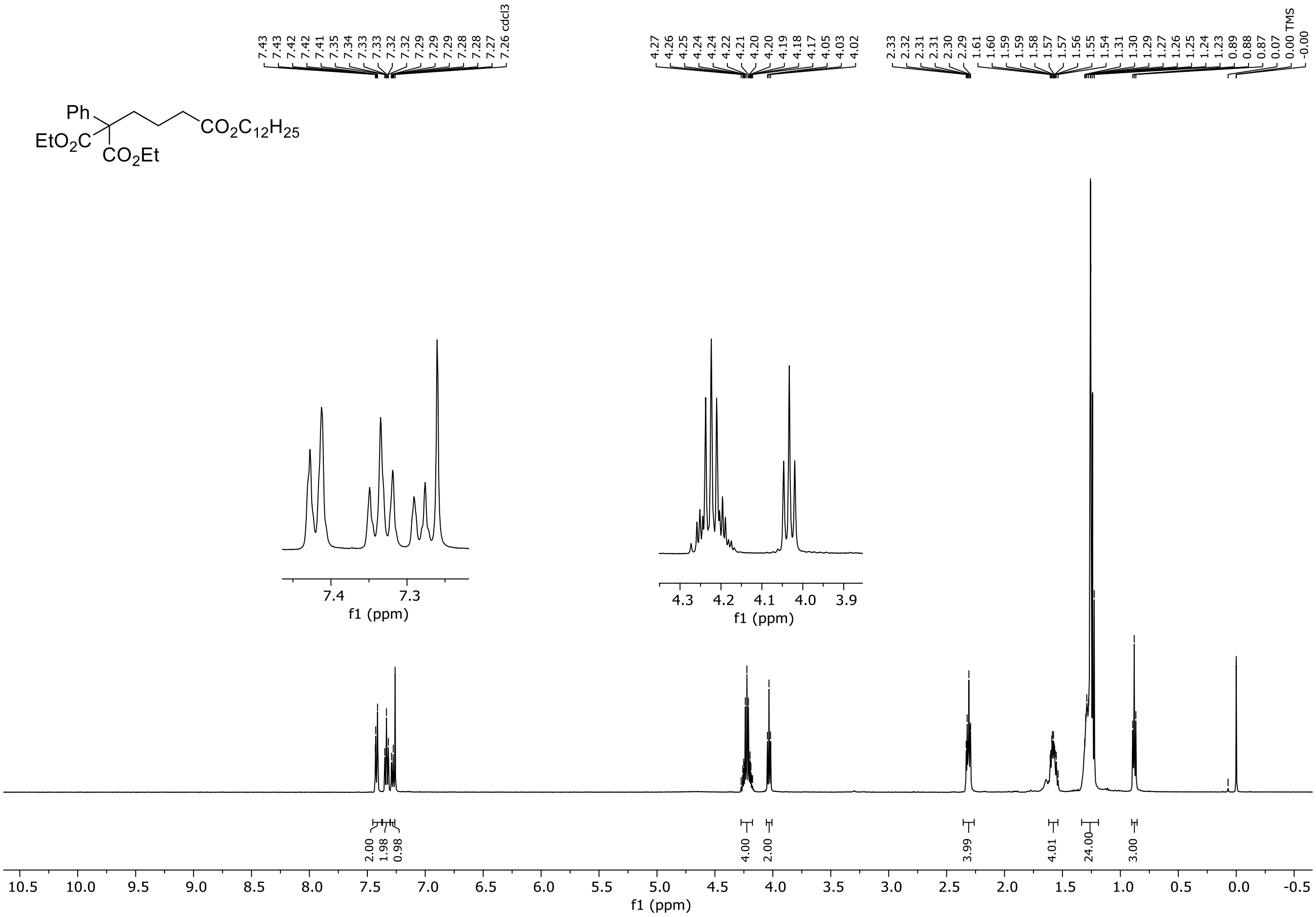

^{13}C NMR ($CDCl_3$, 126 MHz)

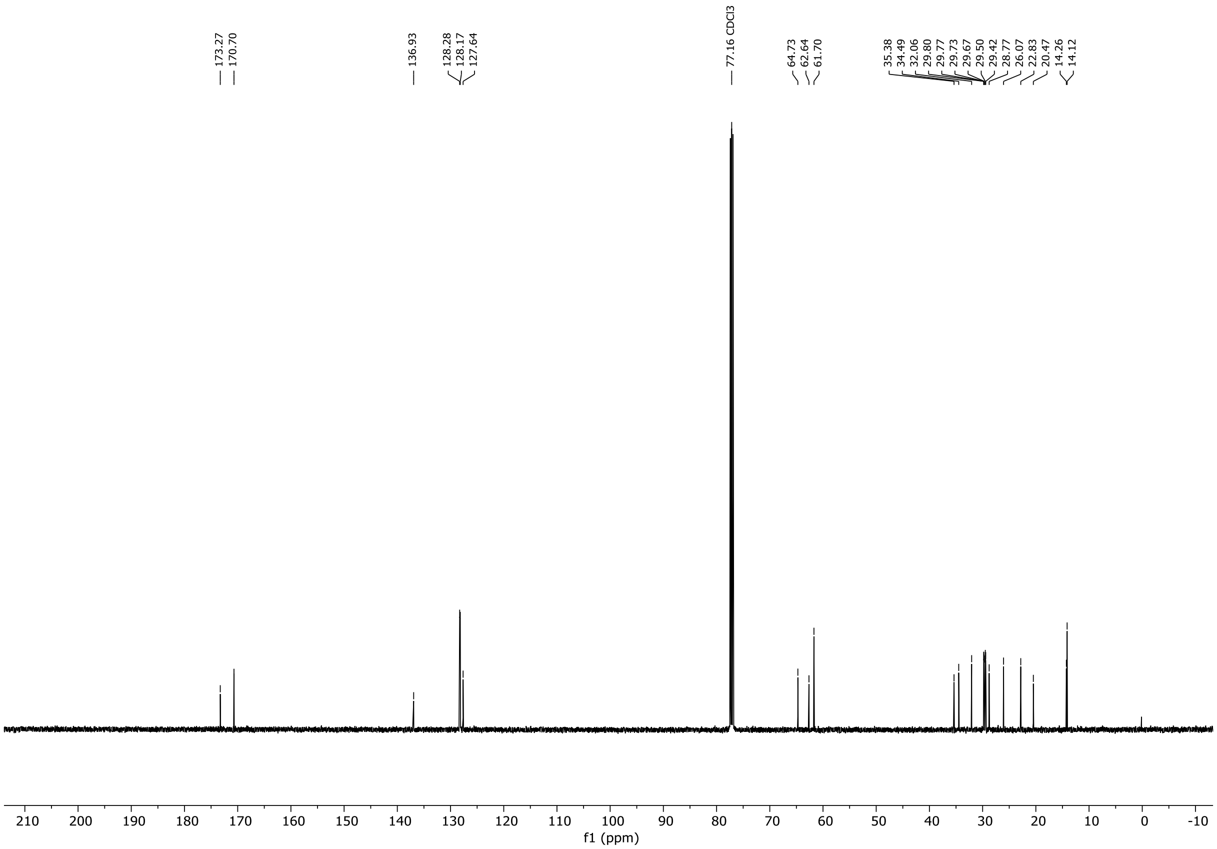

4-dodecyl 1,1-diethyl (E)-1-phenylbut-2-ene-1,1,4-tricarboxylate, 10c

^{1}H NMR (CDCl$_3$, 500 MHz)

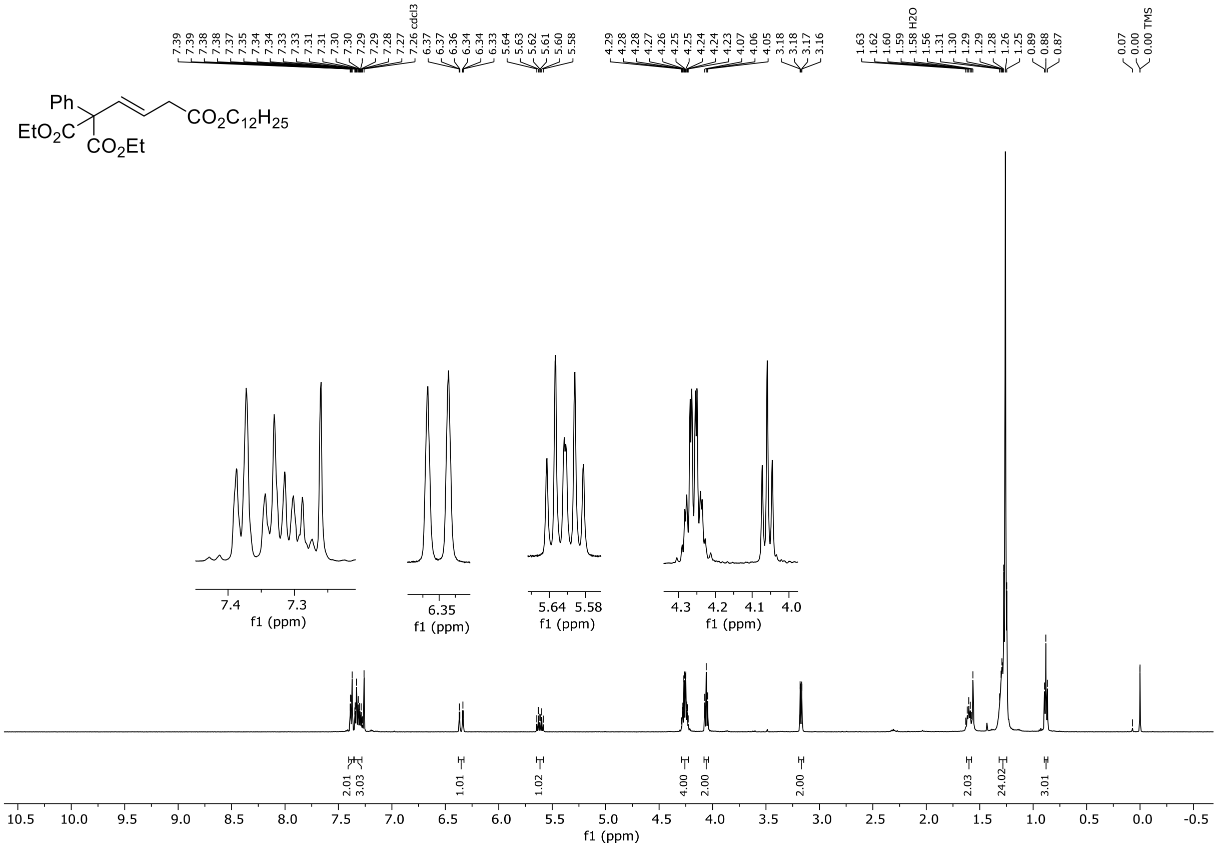

^{13}C NMR (CDCl$_3$, 126 MHz)

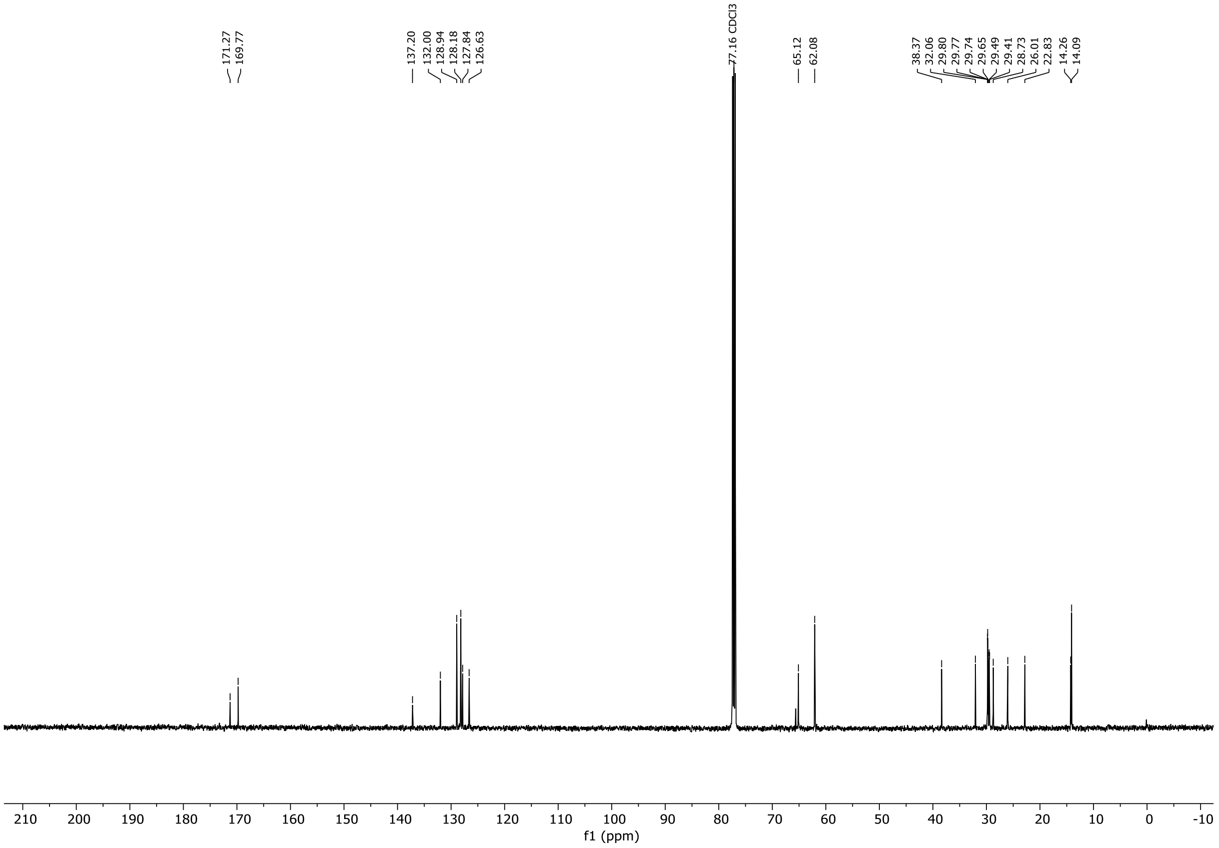

6-dodecyl 1,1-diethyl 2-phenylhexane-1,1,6-tricarboxylate, 11a

^{1}H NMR (CDCl3, 500 MHz)

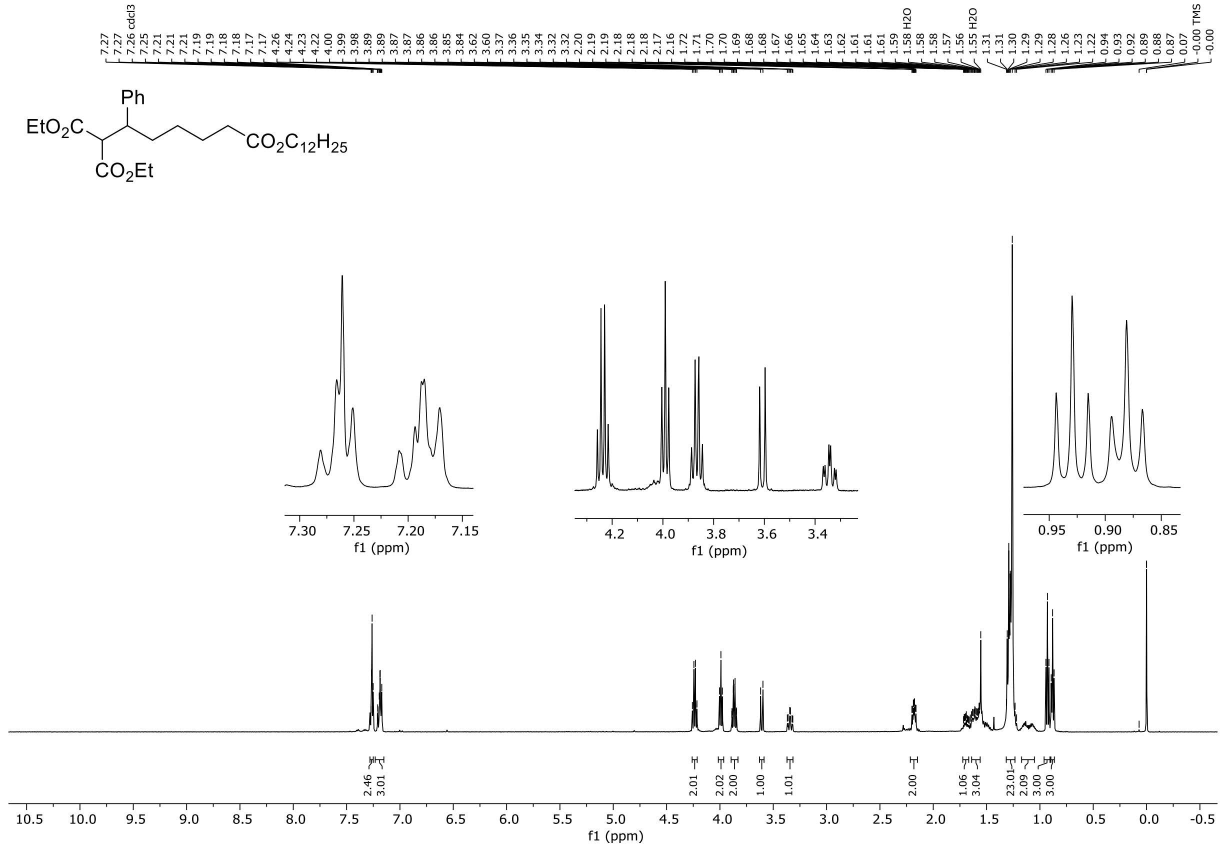

^{13}C NMR (CDCl3, 126 MHz)

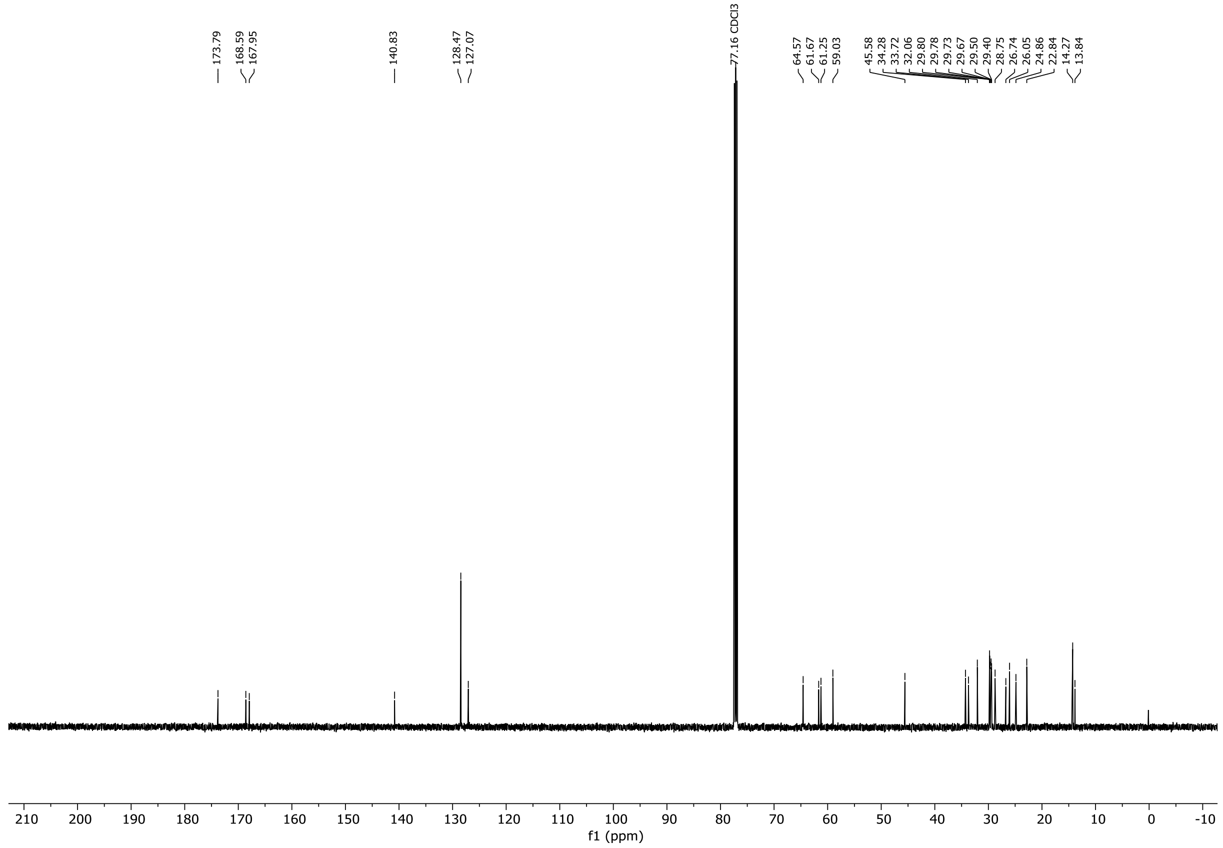

6-dodecyl 1,1-diethyl 1-phenylhexane-1,1,6-tricarboxylate, 11b

^{1}H NMR (CDCl₃, 500 MHz)

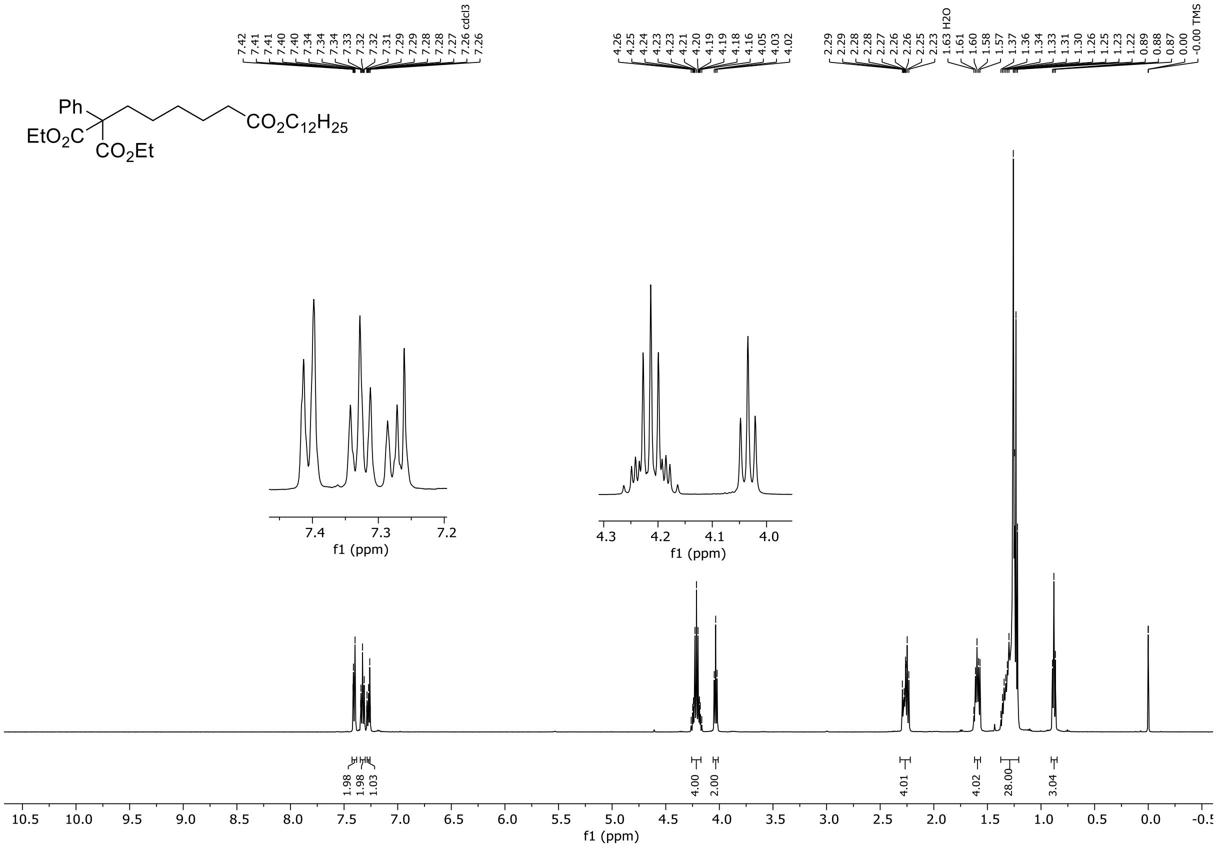

^{13}C NMR (CDCl₃, 126 MHz)

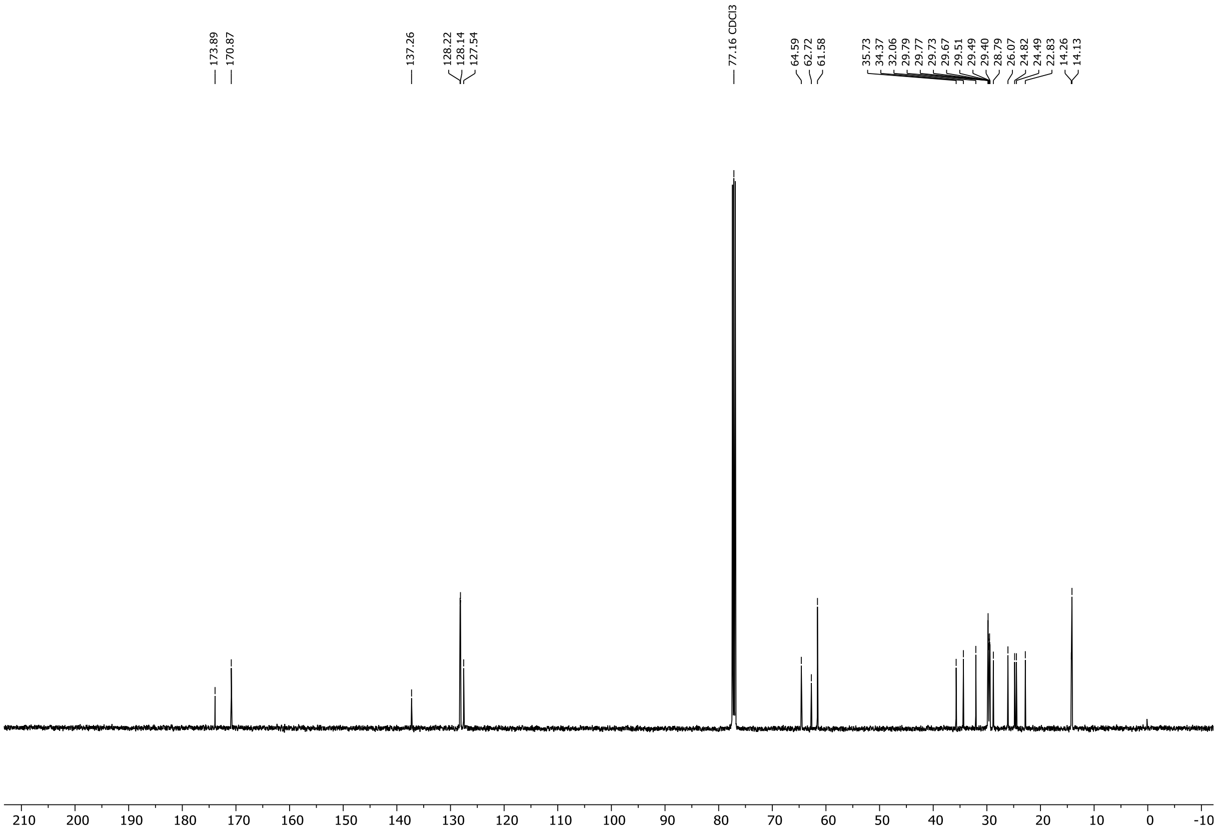

triethyl 2-phenyldecane-1,1,10-tricarboxylate, 13

^{1}H NMR (CDCl$_3$, 500 MHz)

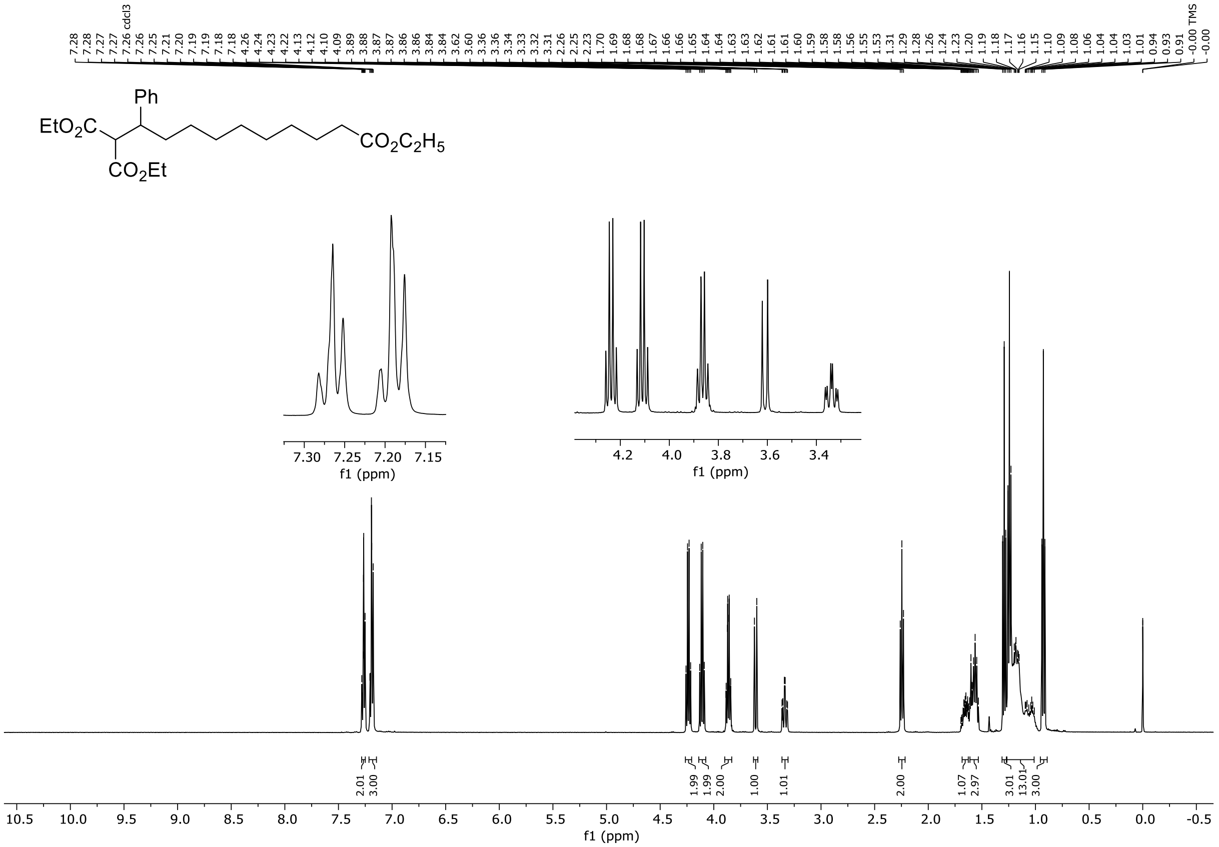

^{13}C NMR (CDCl$_3$, 126 MHz)

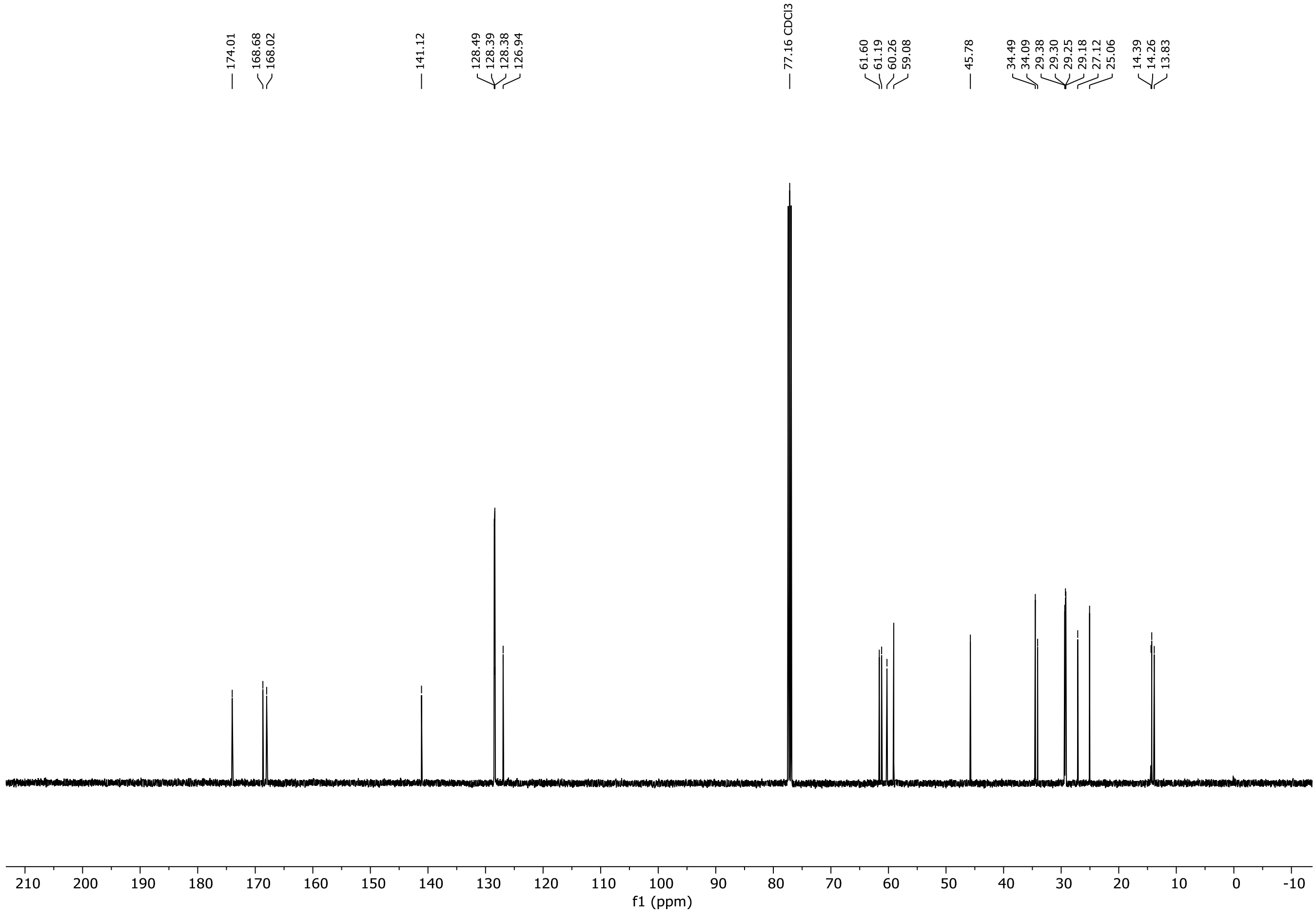

diethyl 2-(4-(2-(2-methoxyethoxy)ethoxy)-1-phenylbutyl)malonate, 14

^{1}H NMR (CDCl$_3$, 500 MHz)

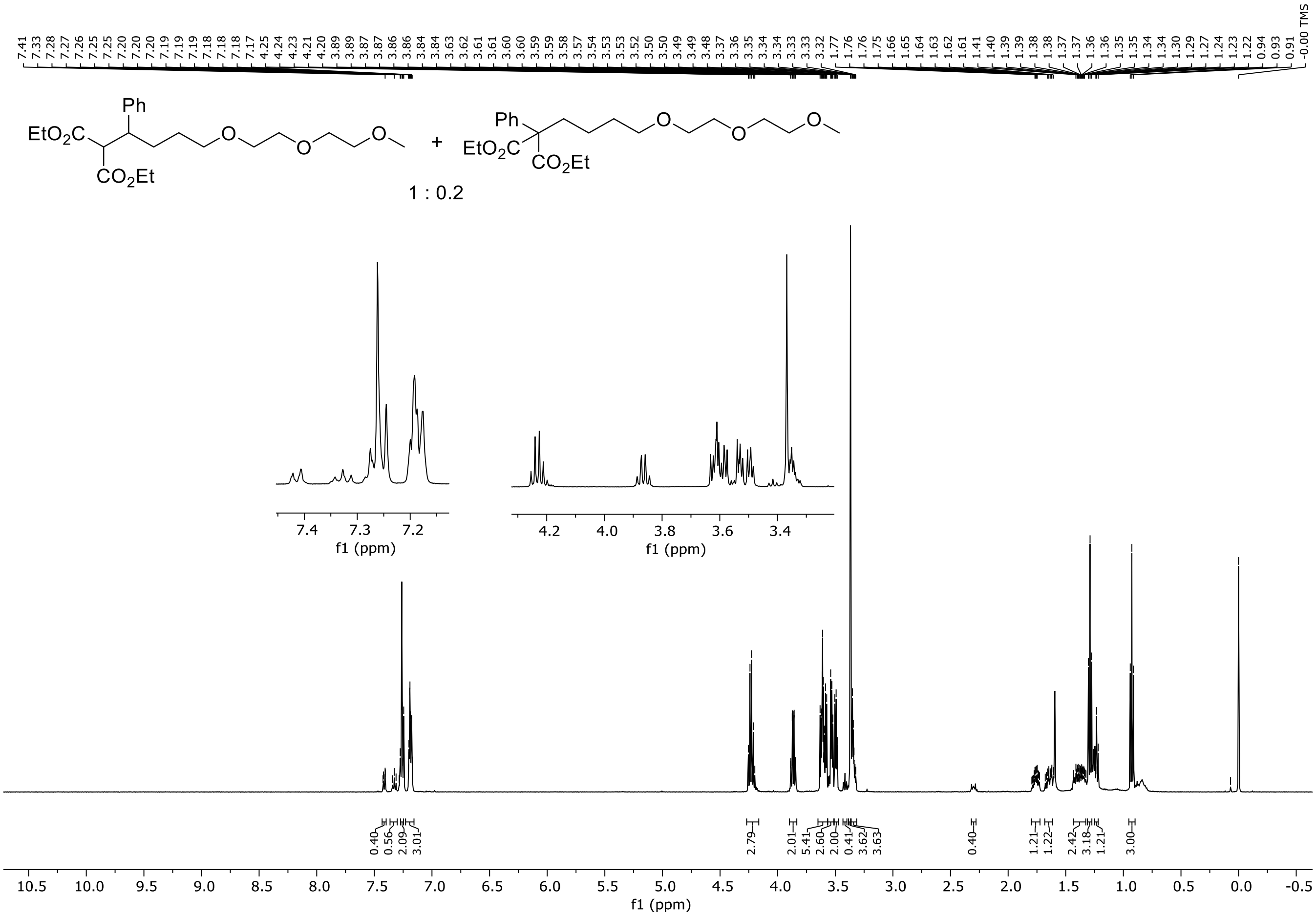

^{13}C NMR (CDCl$_3$, 126 MHz)

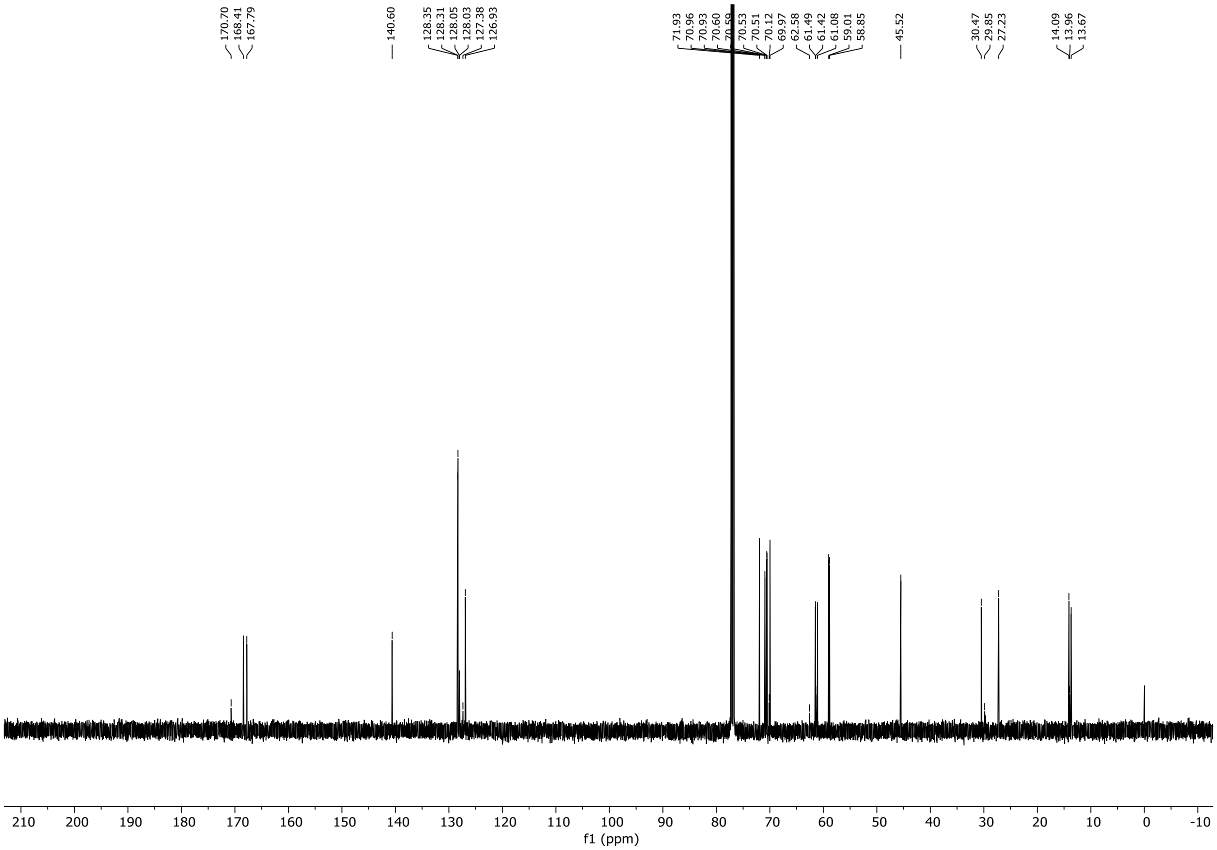

diethyl 2-(4-hydroxy-1-phenyldecyl)malonate, 16a

^{1}H NMR ($CDCl_3$, 500 MHz)

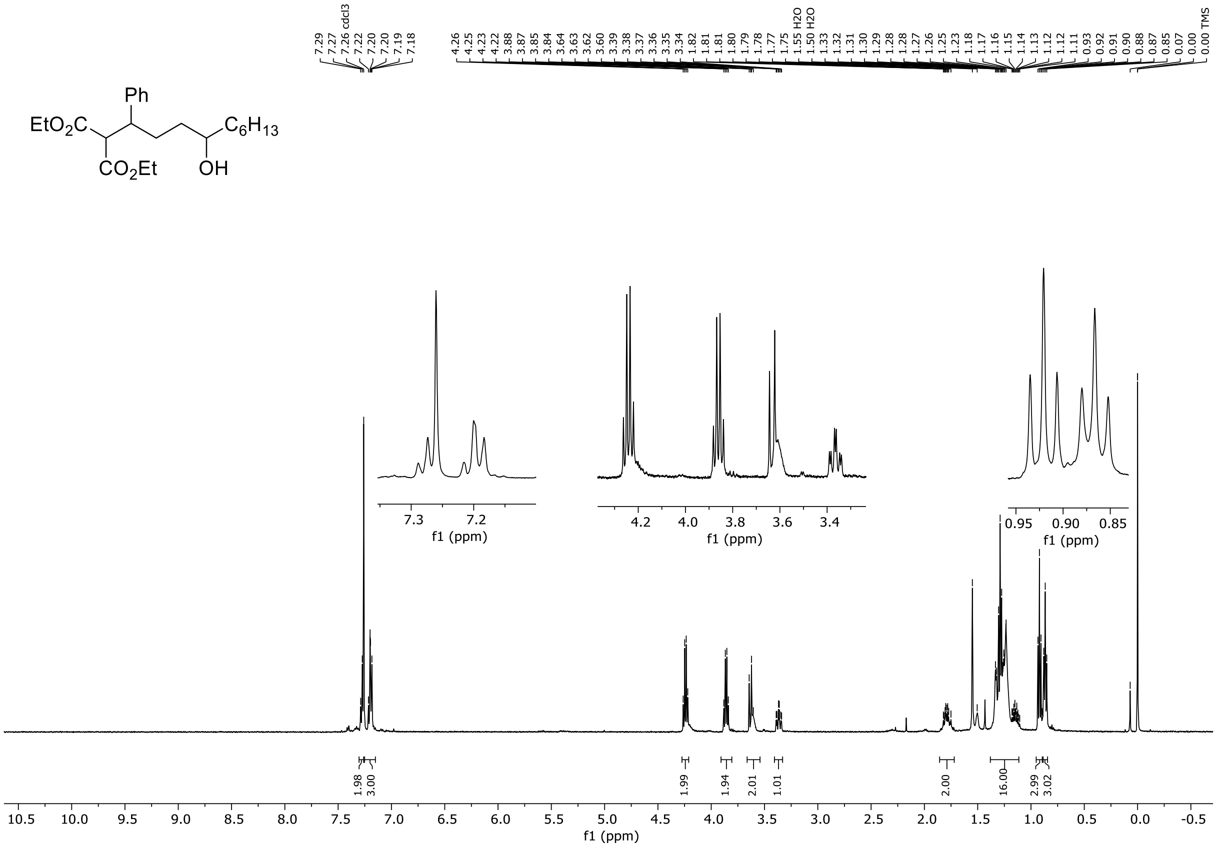

^{13}C NMR ($CDCl_3$, 126 MHz)

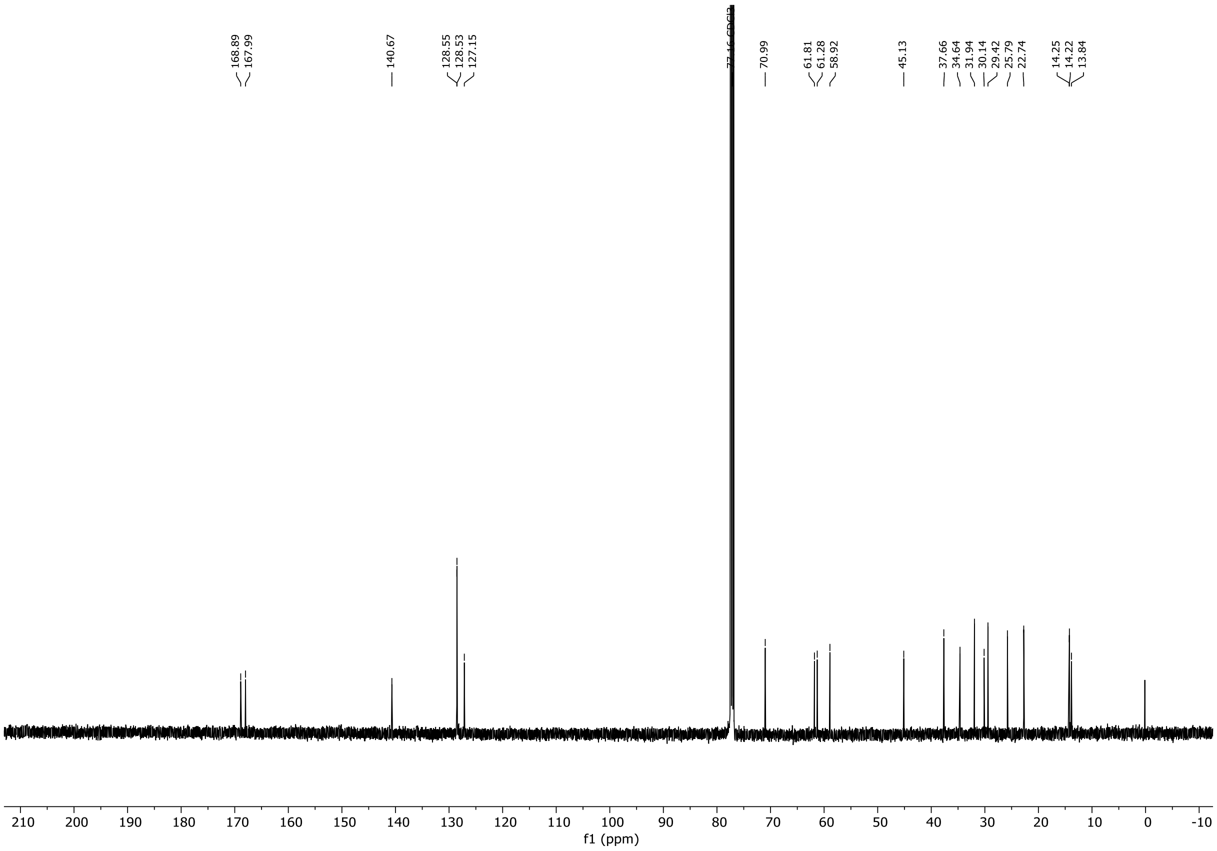

diethyl 2-(4-oxo-1-phenyldecyl)malonate, 16b

^{1}H NMR (CDCl$_3$, 500 MHz)

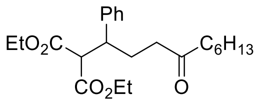

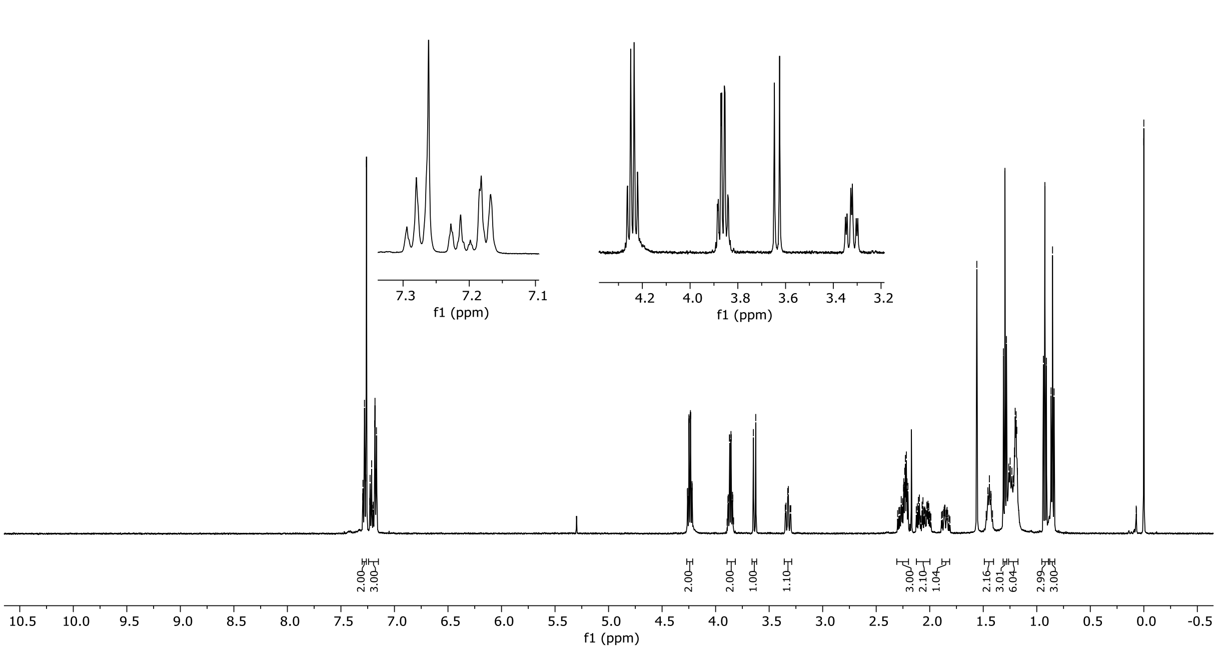

^{13}C NMR (CDCl$_3$, 126 MHz)

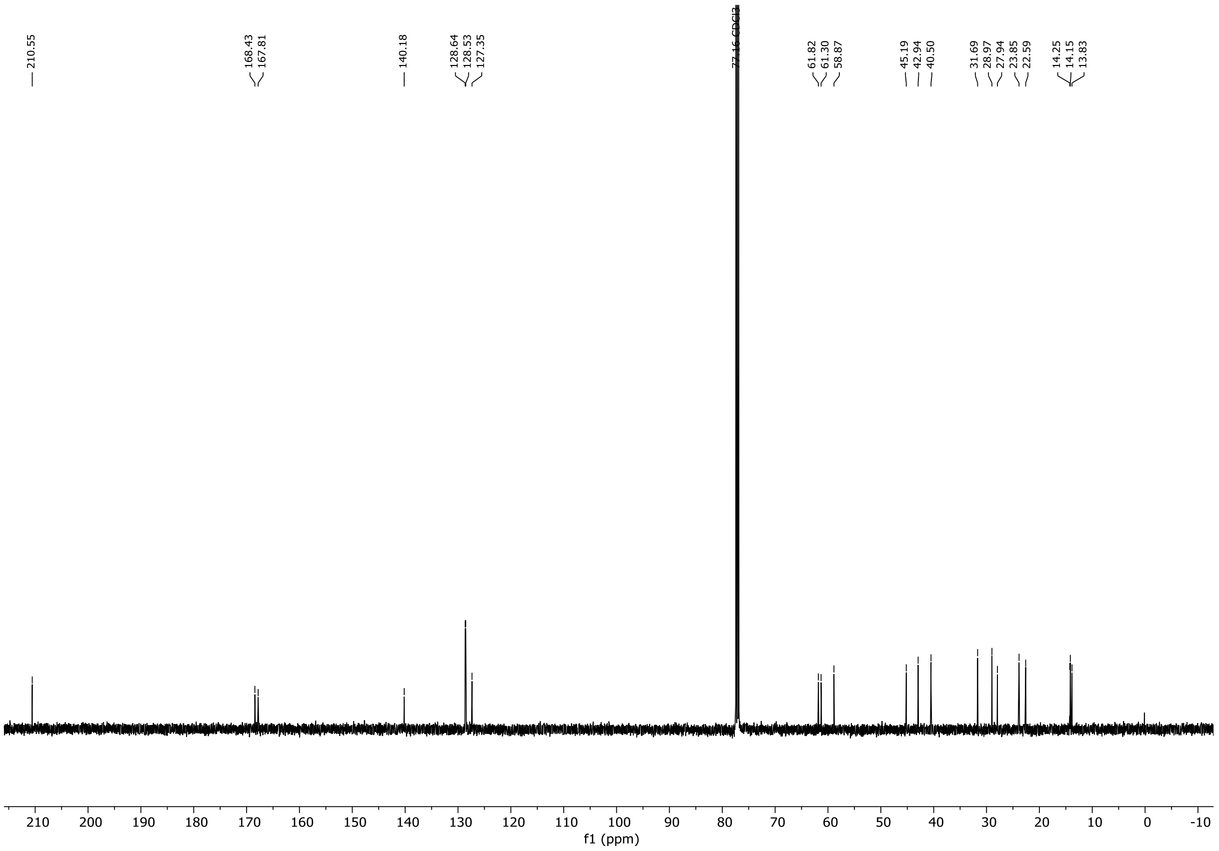

diethyl 2-(4-hydroxydecyl)-2-phenylmalonate, 16c

^{1}H NMR (CDCl3, 500 MHz)

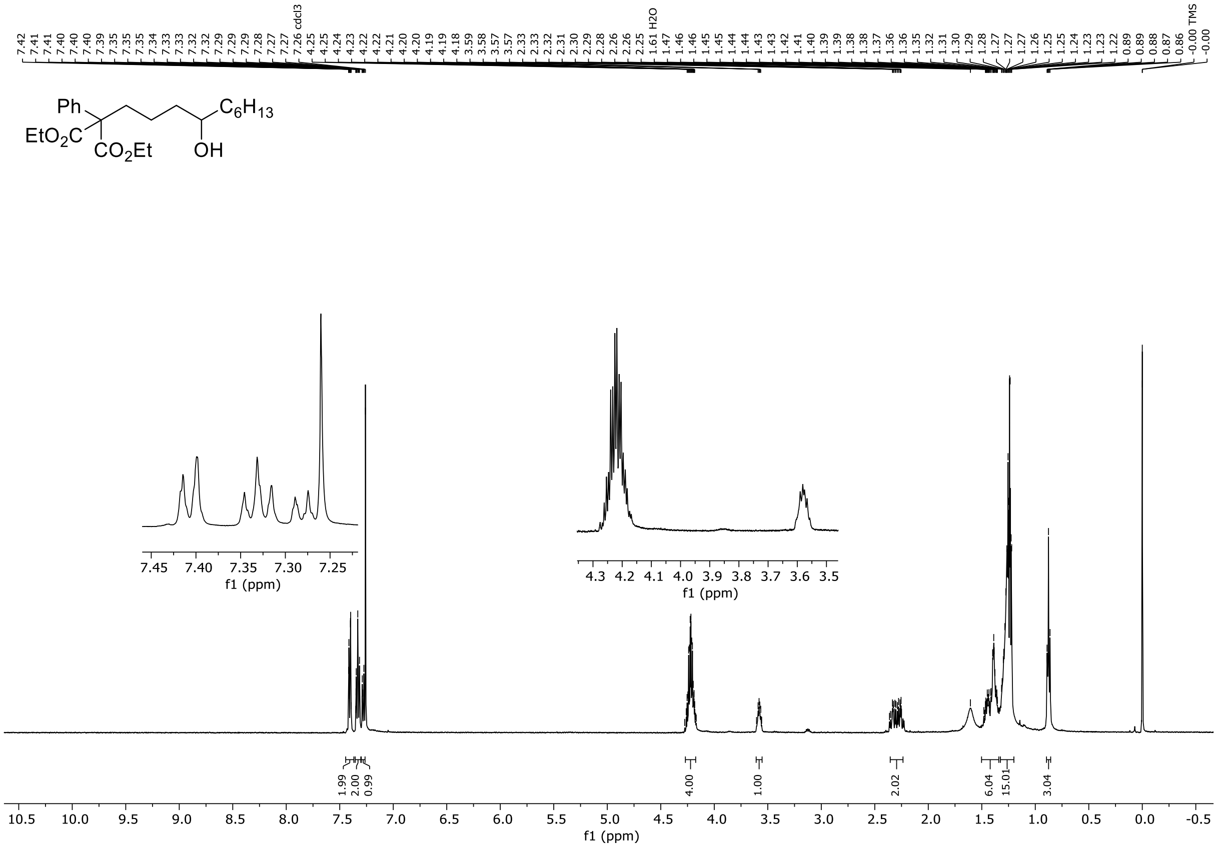

^{13}C NMR (CDCl3, 126 MHz)

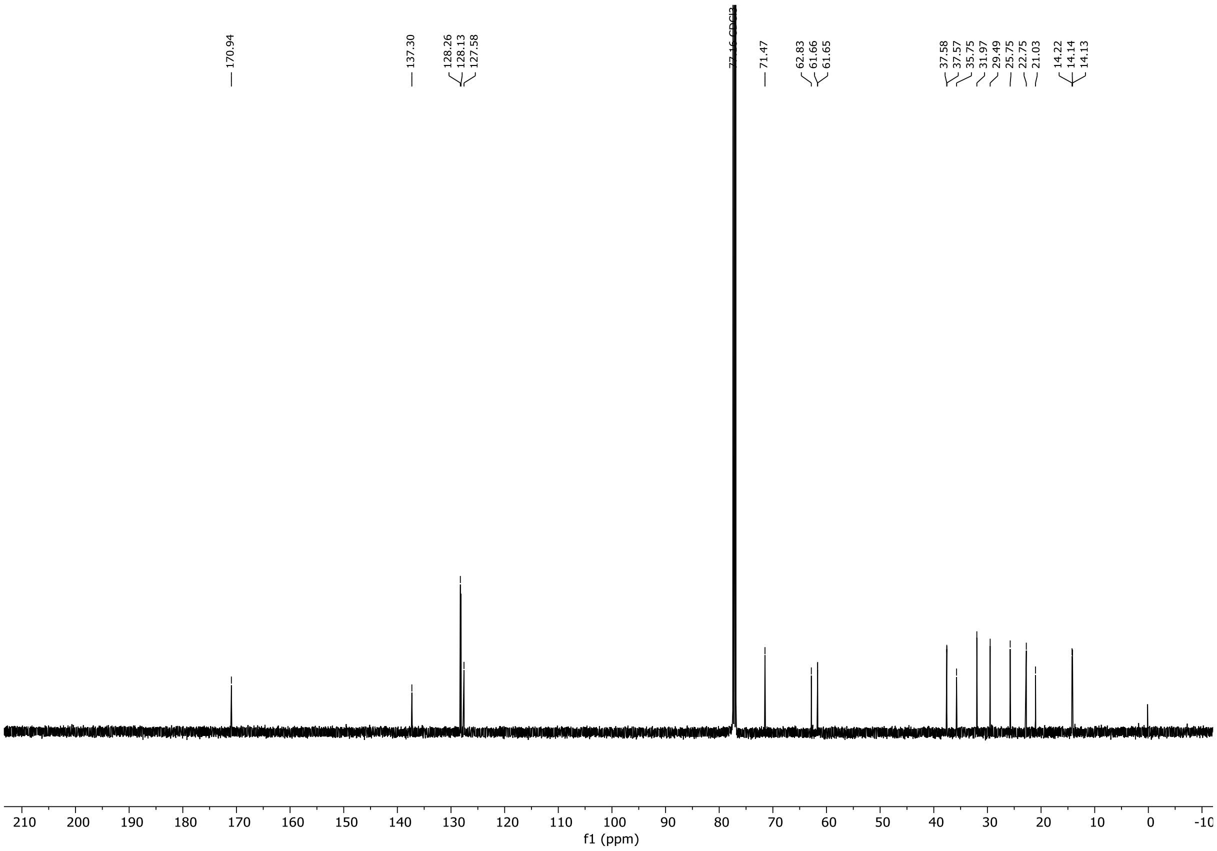

diethyl 2-(7-hydroxy-1-phenyldodecyl)malonate, 17

^{1}H NMR (CDCl$_3$, 500 MHz)

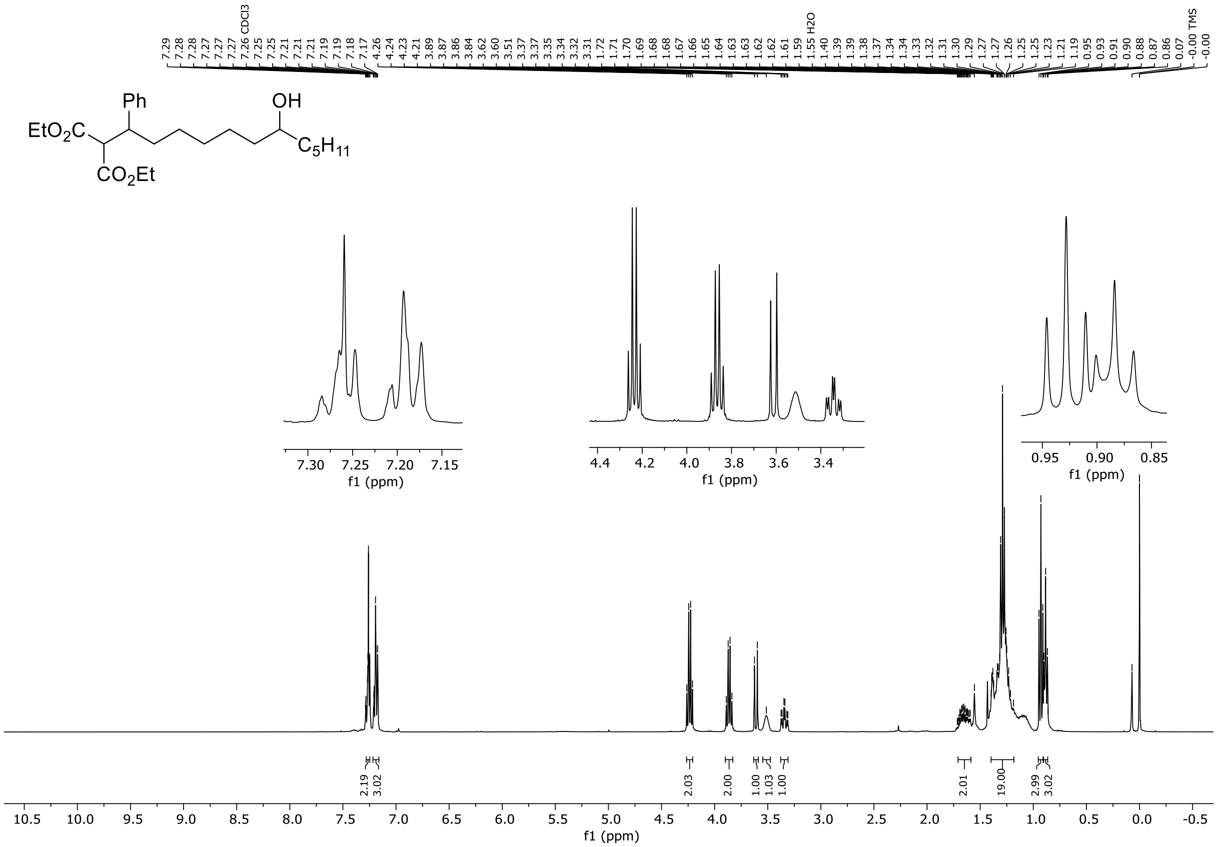

^{13}C NMR (CDCl$_3$, 126 MHz)

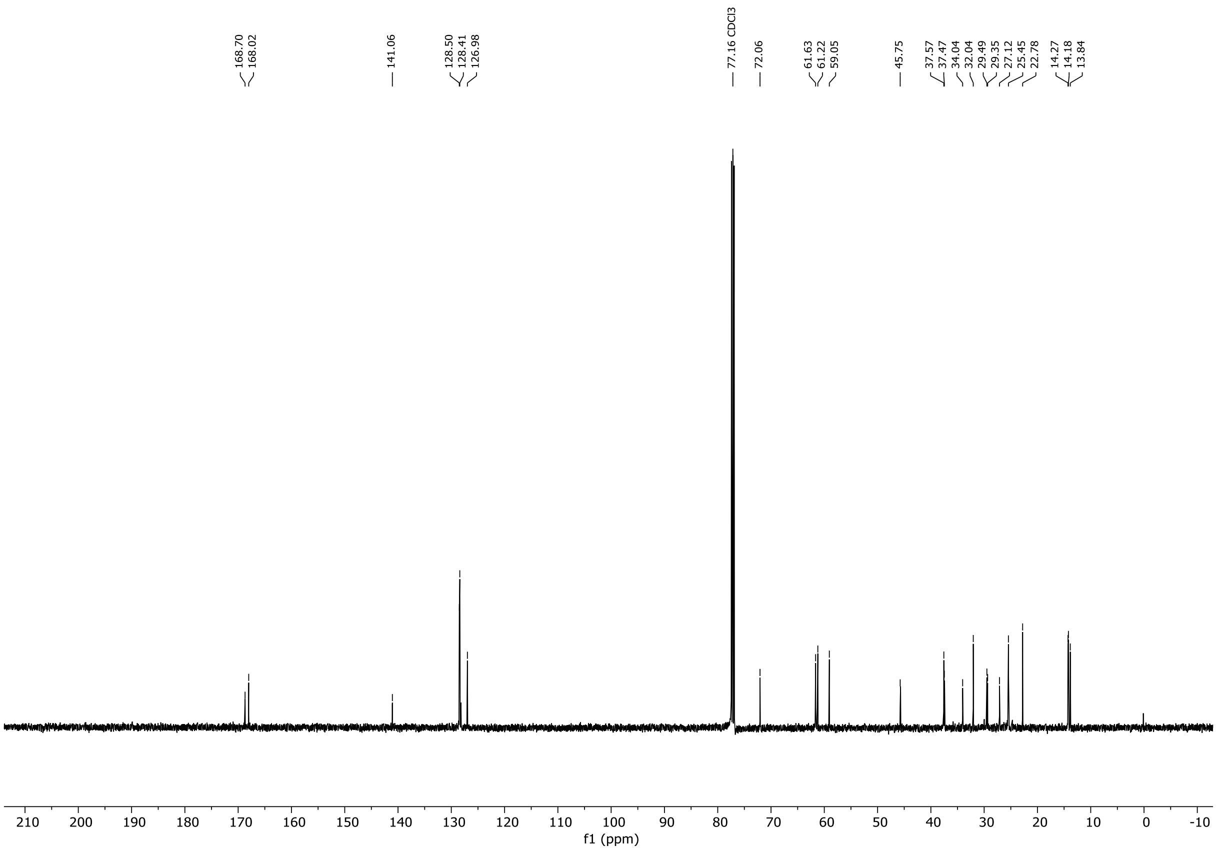

diethyl 2-(10-hydroxy-1-phenyldecyl)malonate, 18

^{1}H NMR ($CDCl_3$, 500 MHz)

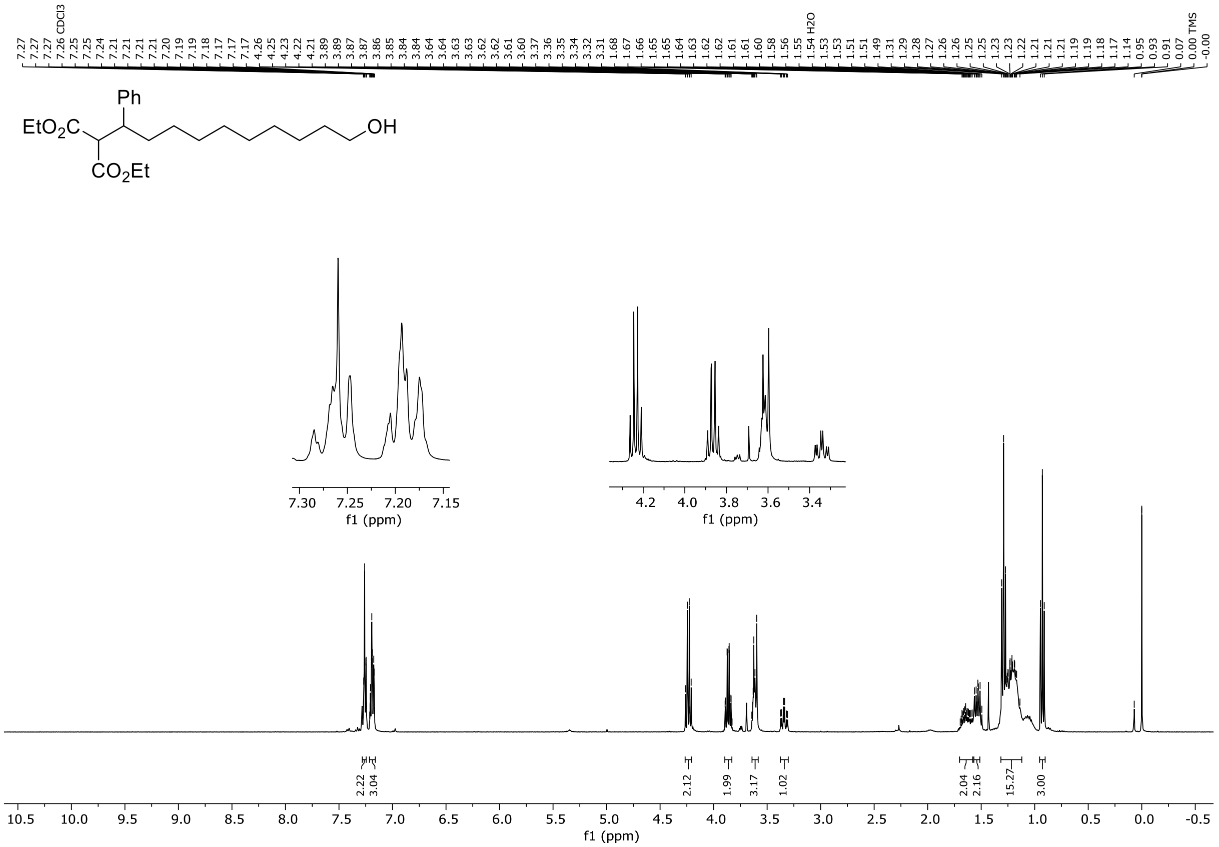

^{13}C NMR ($CDCl_3$, 126 MHz)

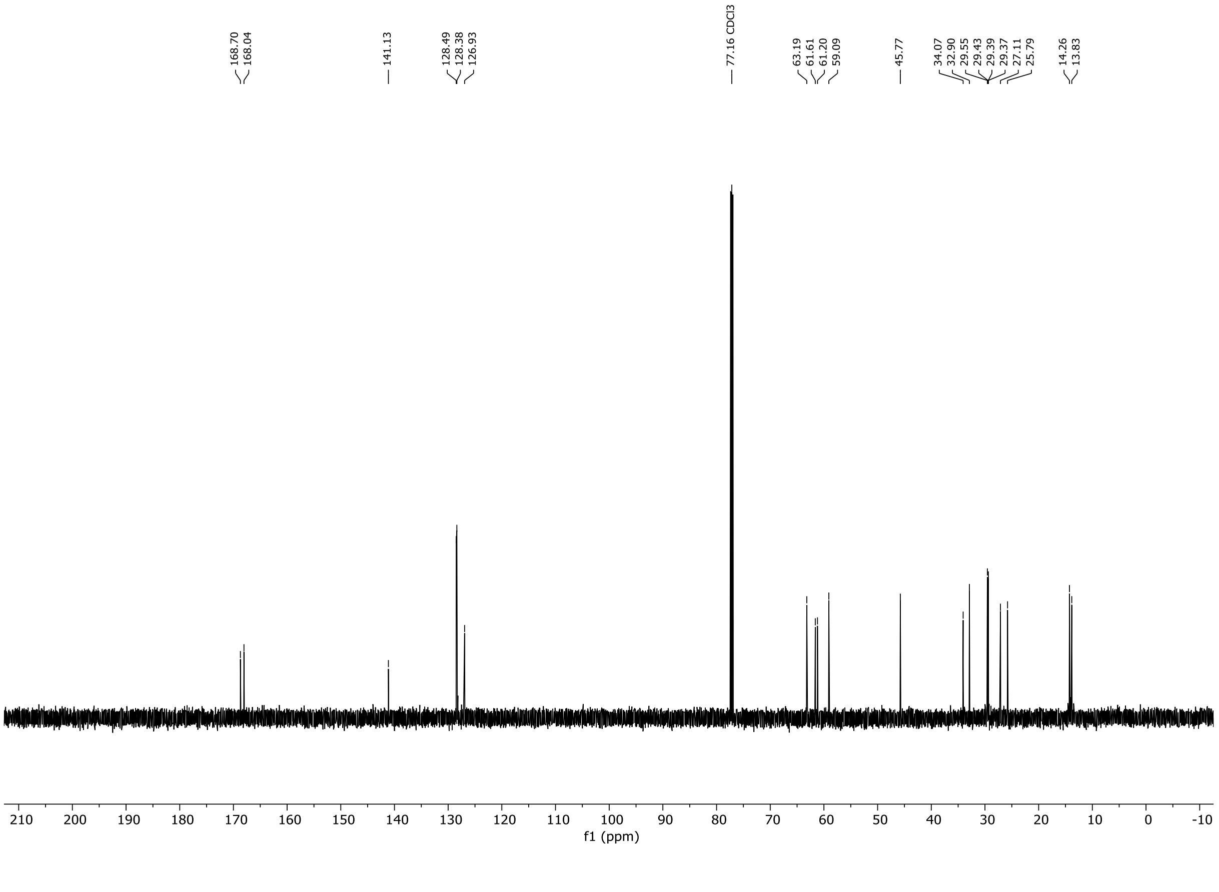

diethyl 2-(1-(4-cyanophenyl)tetradecyl)malonate, 19a

^{1}H NMR (CDCl₃, 500 MHz)

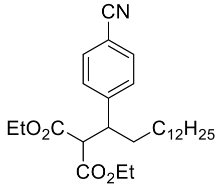

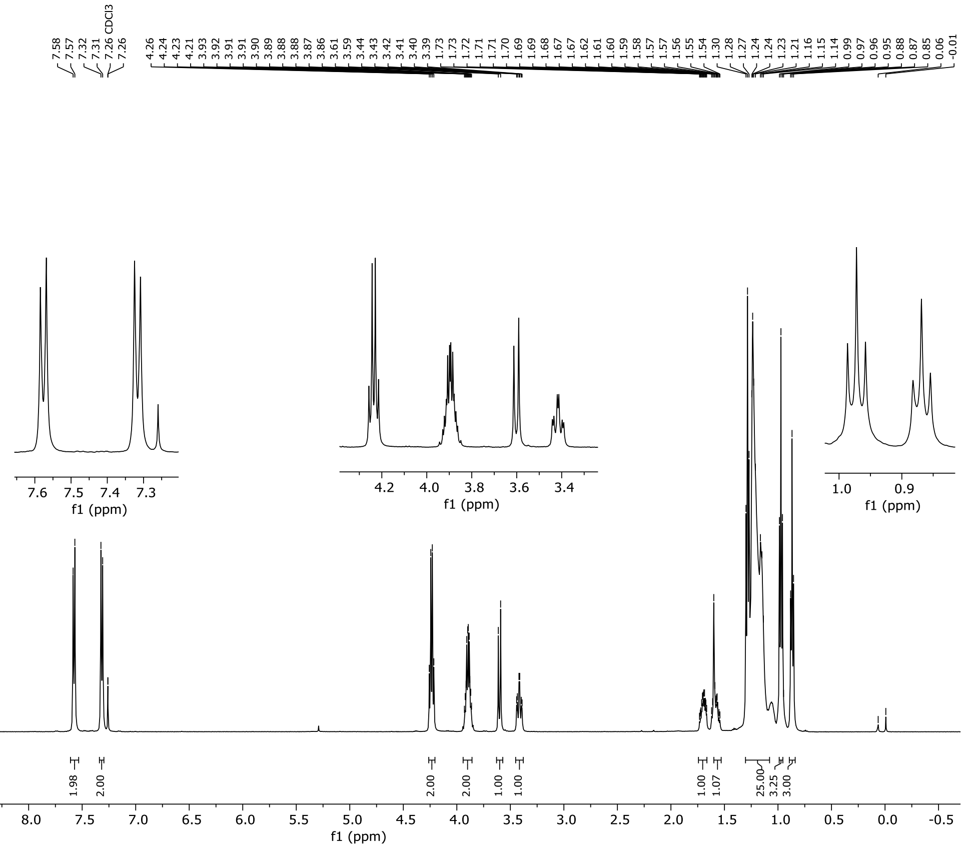

^{13}C NMR (CDCl₃, 126 MHz)

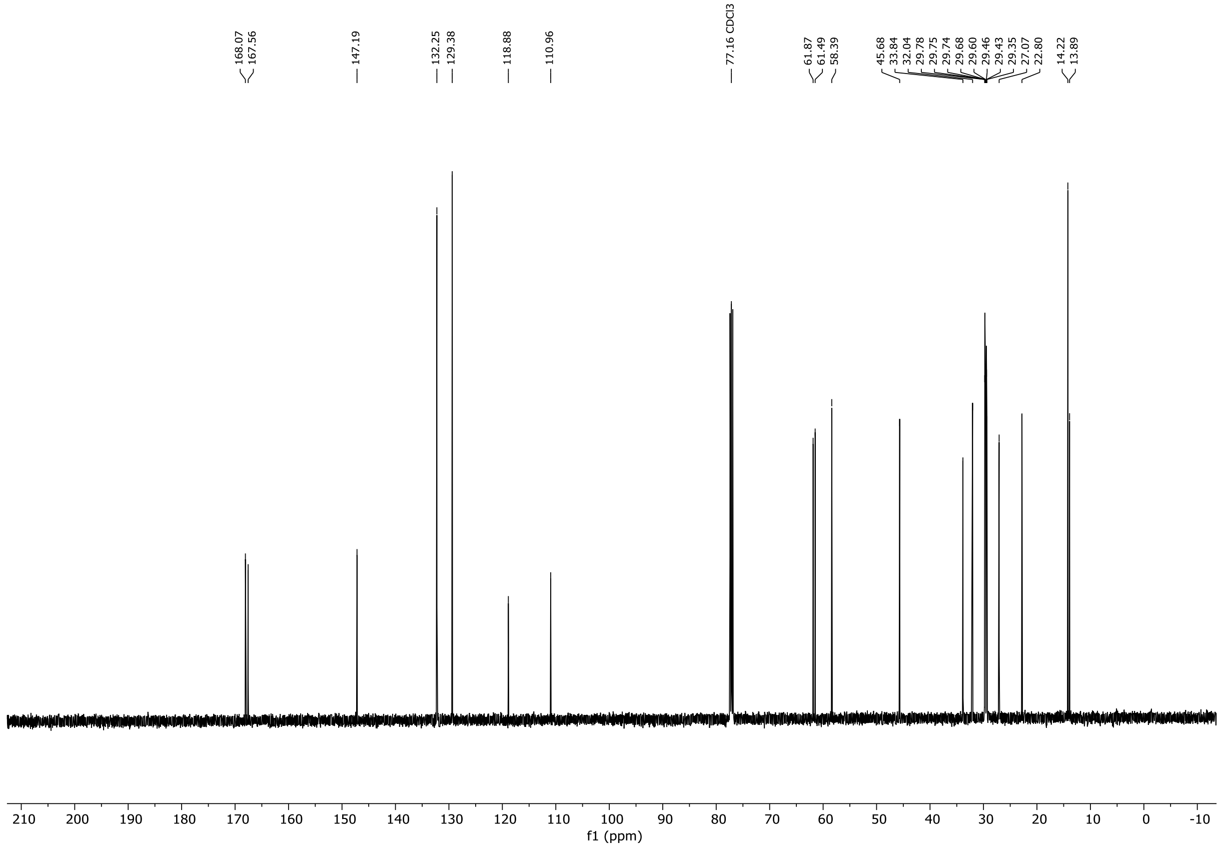

diethyl 2-(1-(4-(trifluoromethyl)phenyl)tetradecyl)malonate, 19b

^{1}H NMR ($CDCl_3$, 500 MHz)

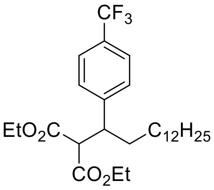

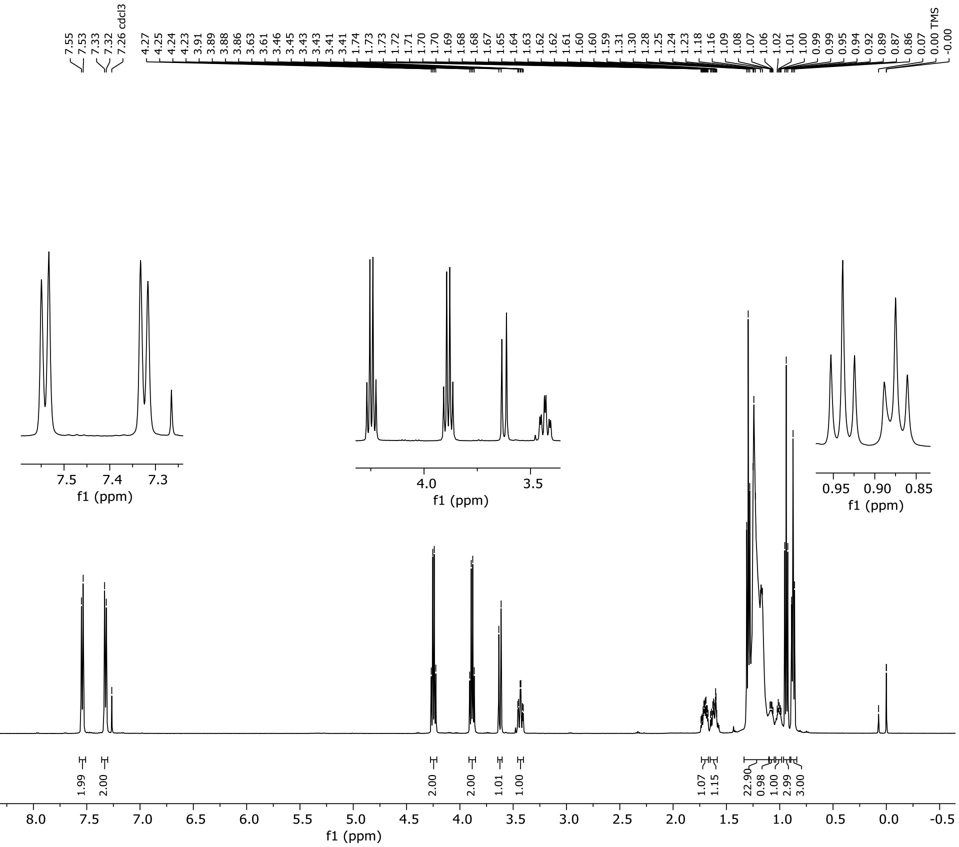

^{13}C NMR ($CDCl_3$, 126 MHz)

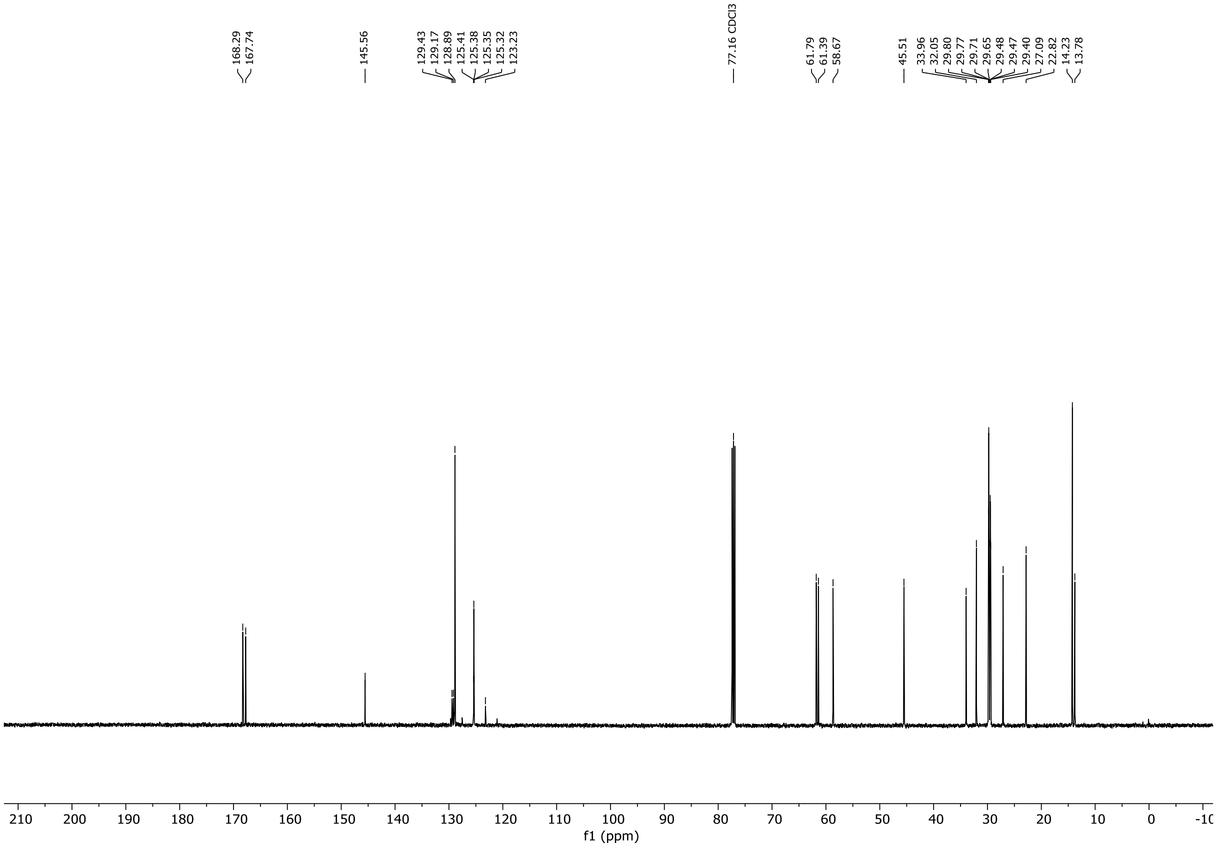

^{19}F NMR ($CDCl_3$, 470 MHz)

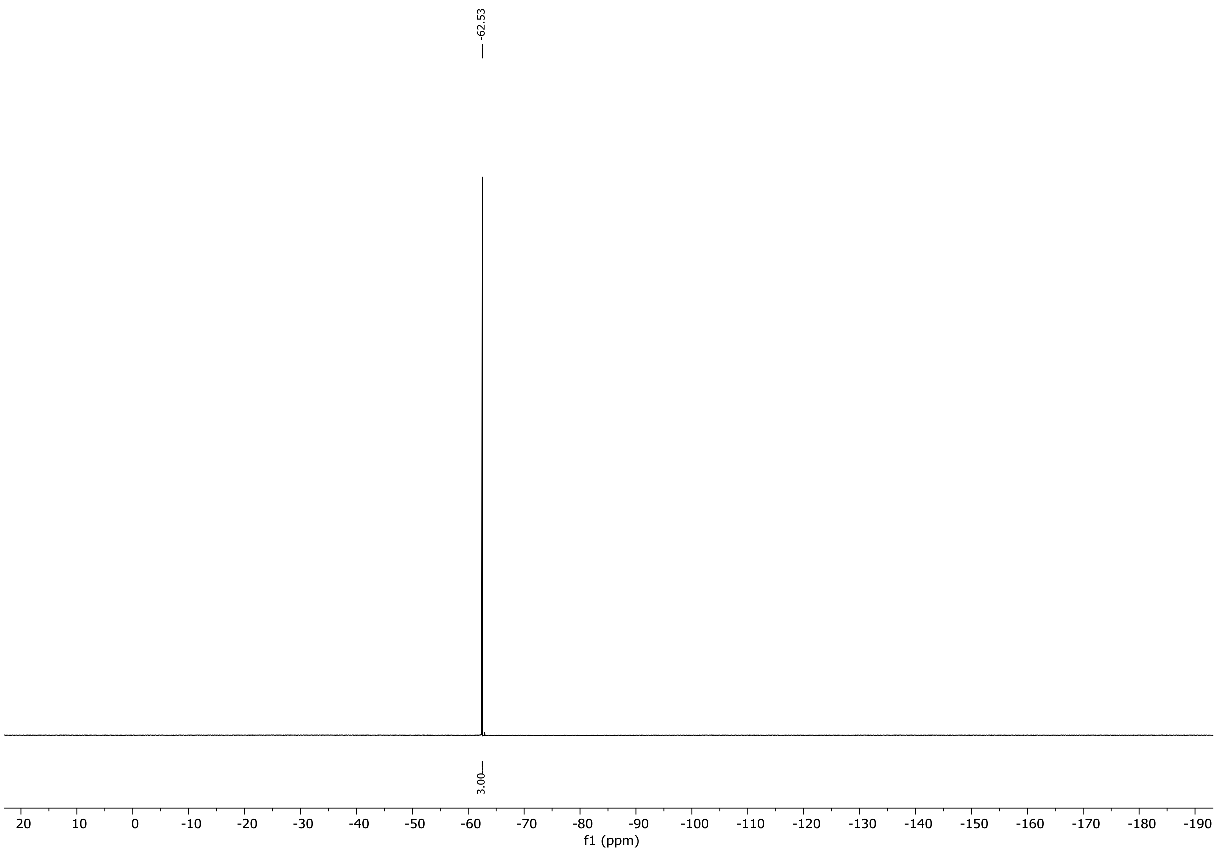

diethyl 2-(1-(4-methoxyphenyl)tetradecyl)malonate, 20a

^{1}H NMR (CDCl$_3$, 500 MHz)

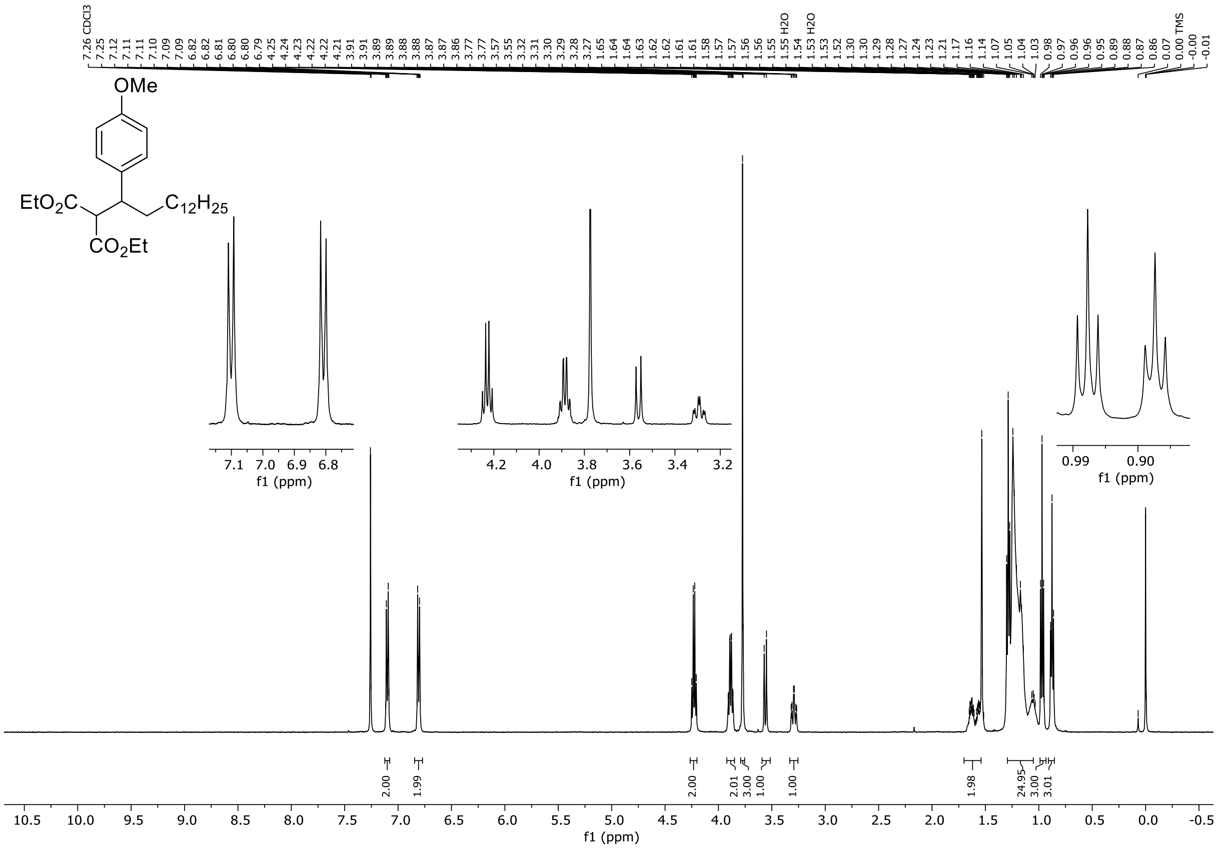

^{13}C NMR (CDCl$_3$, 126 MHz)

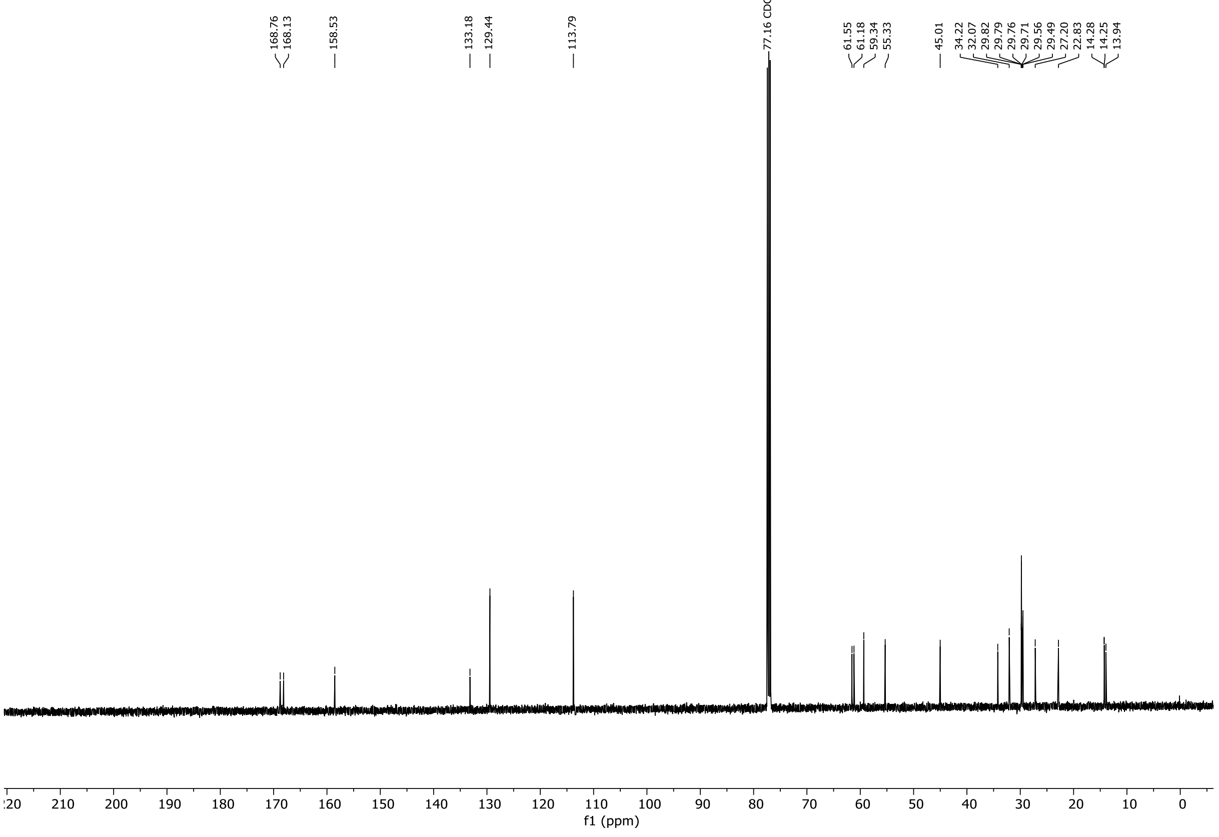

diethyl 2-(4-methoxyphenyl)-2-tetradecylmalonate, 20b

^{1}H NMR (CDCl₃, 500 MHz)

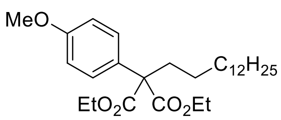

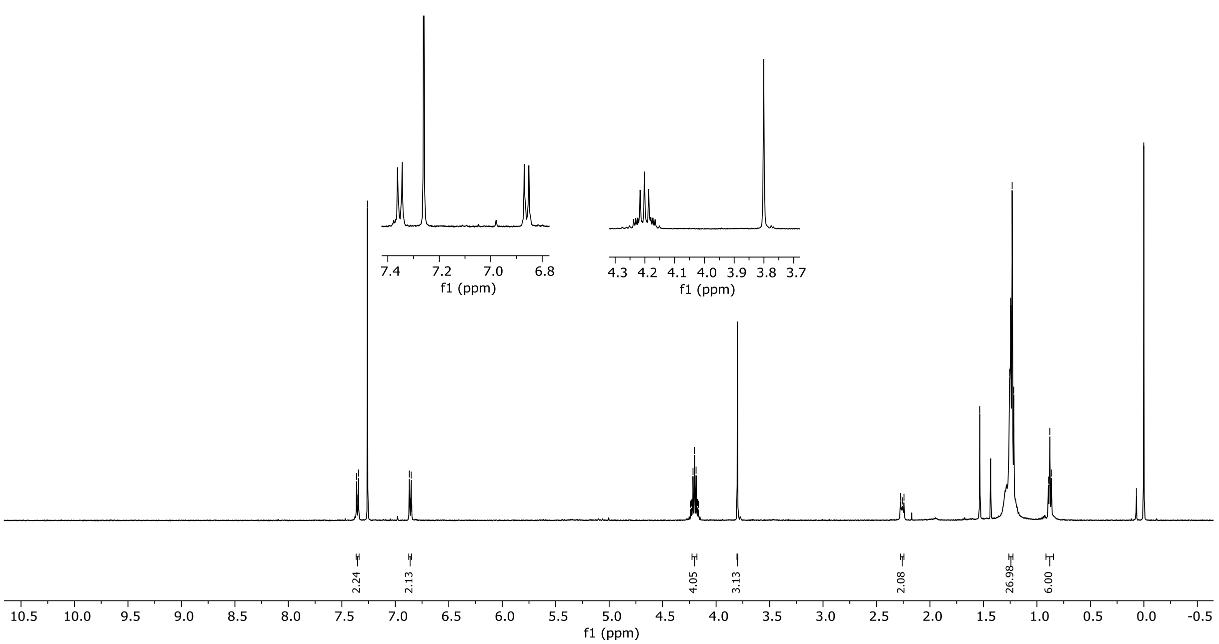

^{13}C NMR (CDCl₃, 126 MHz)

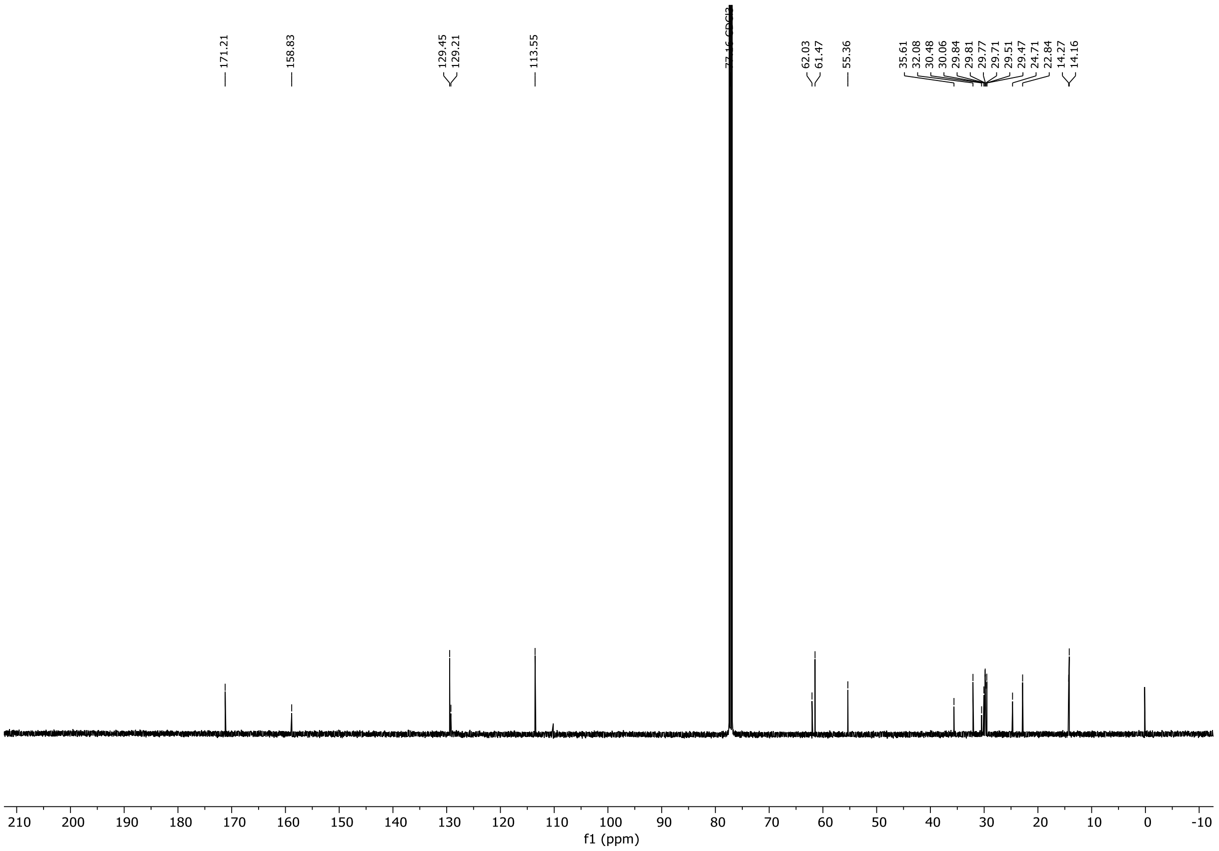

diethyl 2-(1-(3-methoxyphenyl)tetradecyl)malonate, 21

^{1}H NMR ($CDCl_3$, 600 MHz)

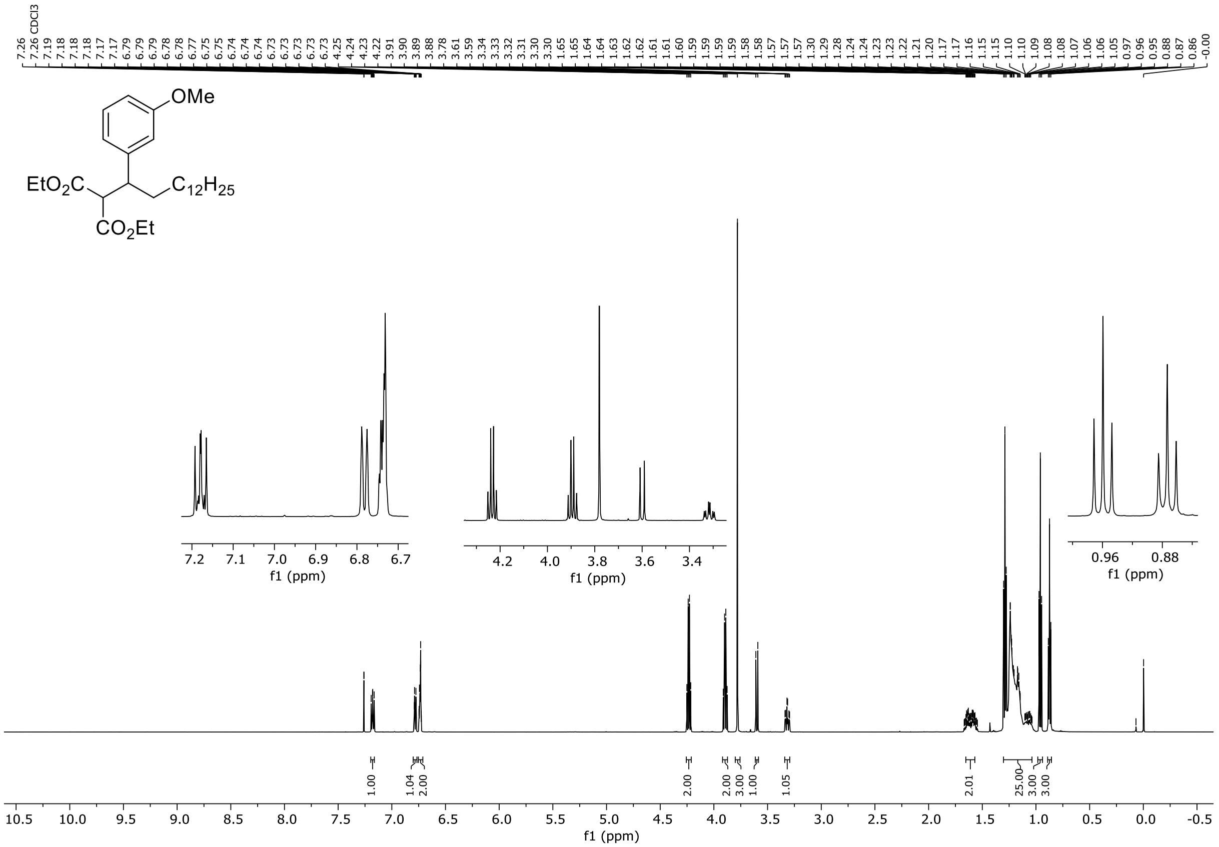

^{13}C NMR ($CDCl_3$, 126 MHz)

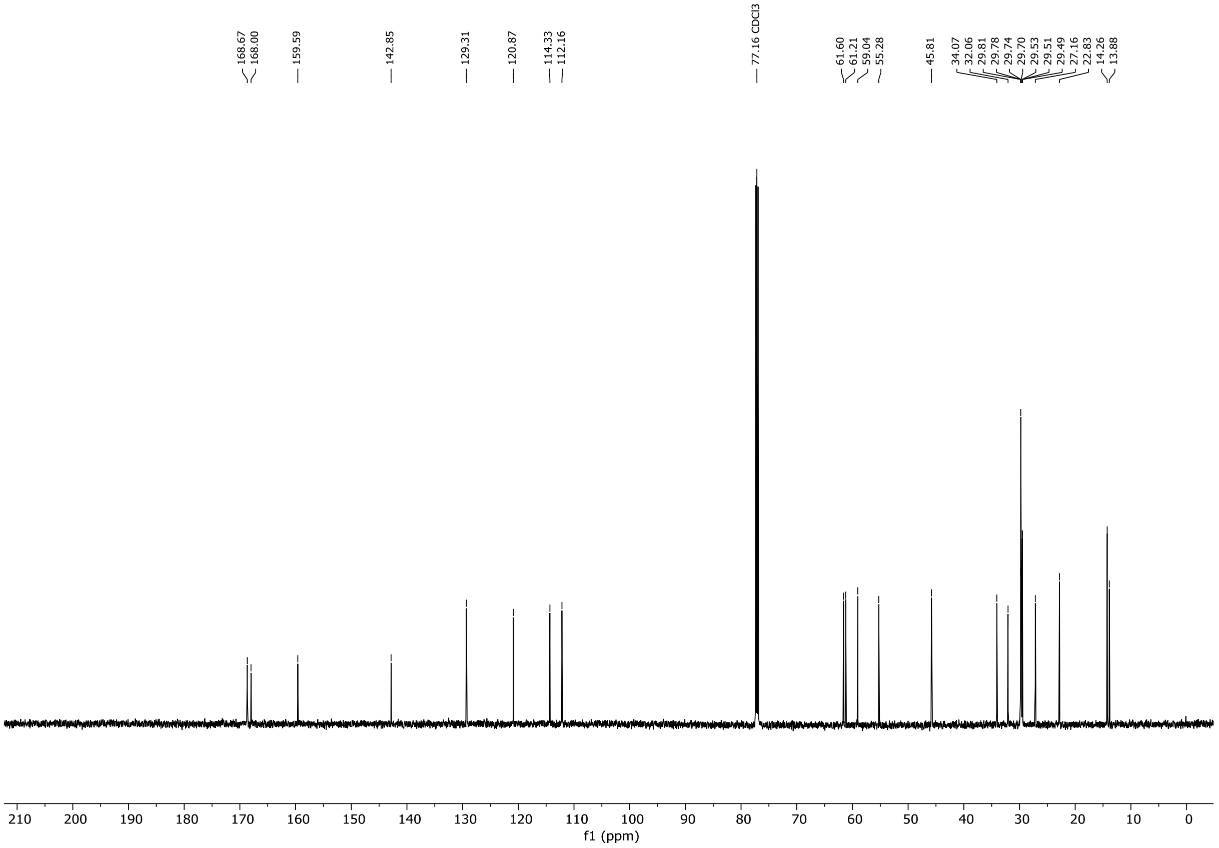

diethyl 2-(3-methoxyphenyl)-2-vinylmalonate, S3

^{1}H NMR ($CDCl_3$, 600 MHz)

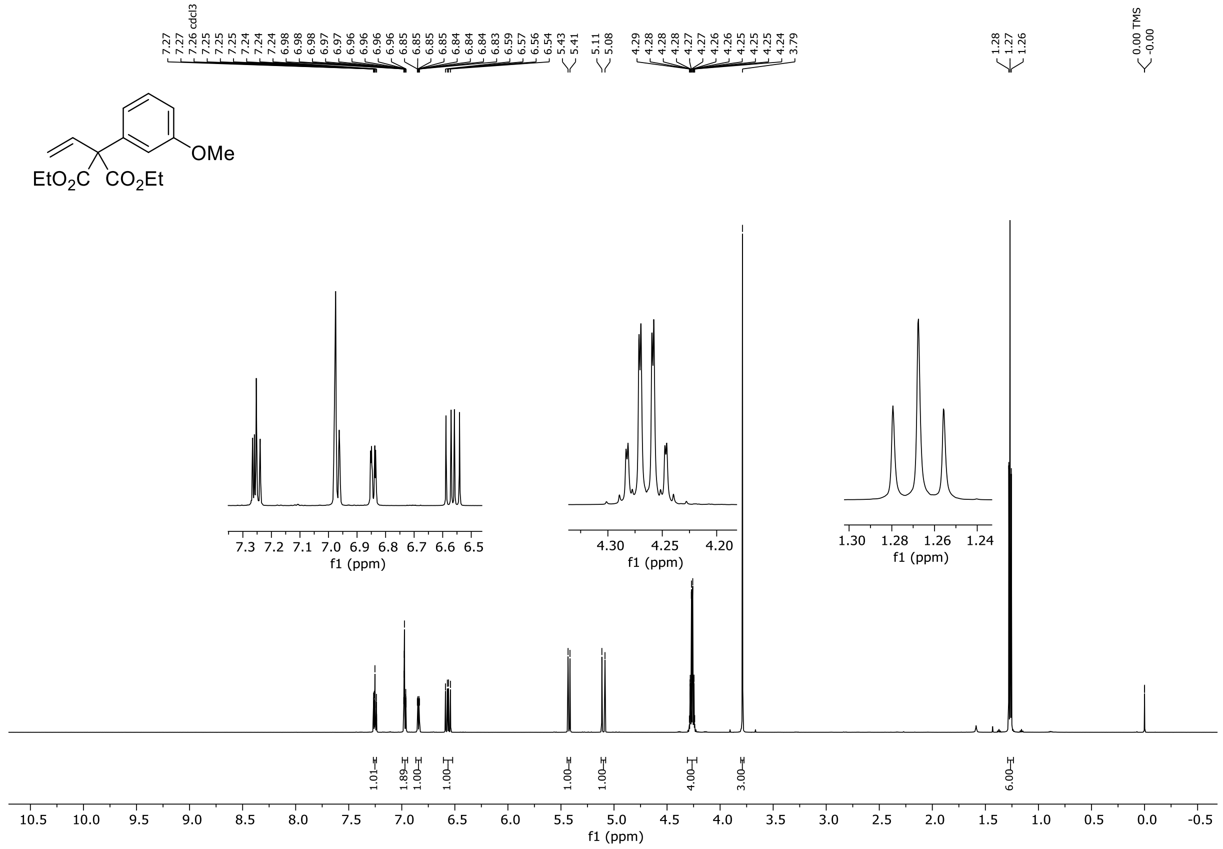

^{13}C NMR ($CDCl_3$, 126 MHz)

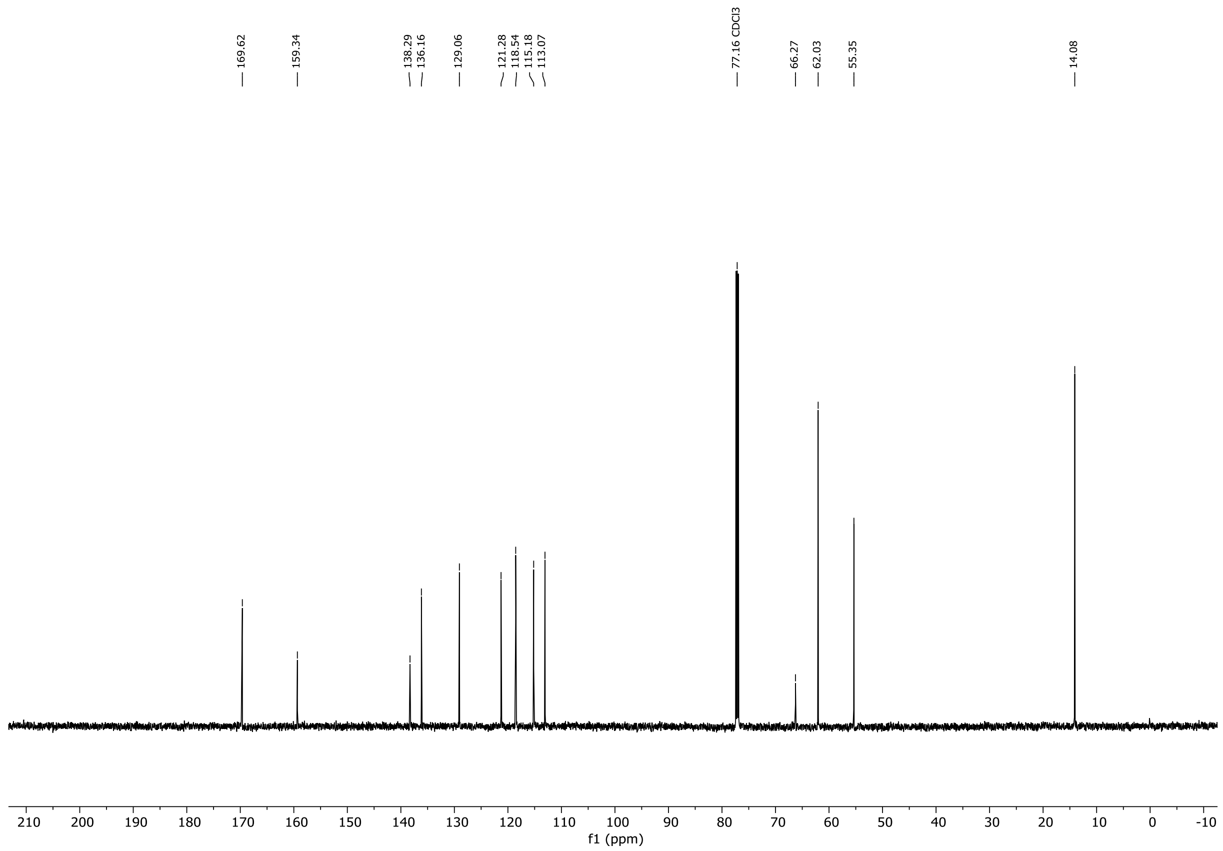

diethyl 2-(4-(trifluoromethyl)phenyl)-2-vinylmalonate, S5

^{1}H NMR (CDCl3, 500 MHz)

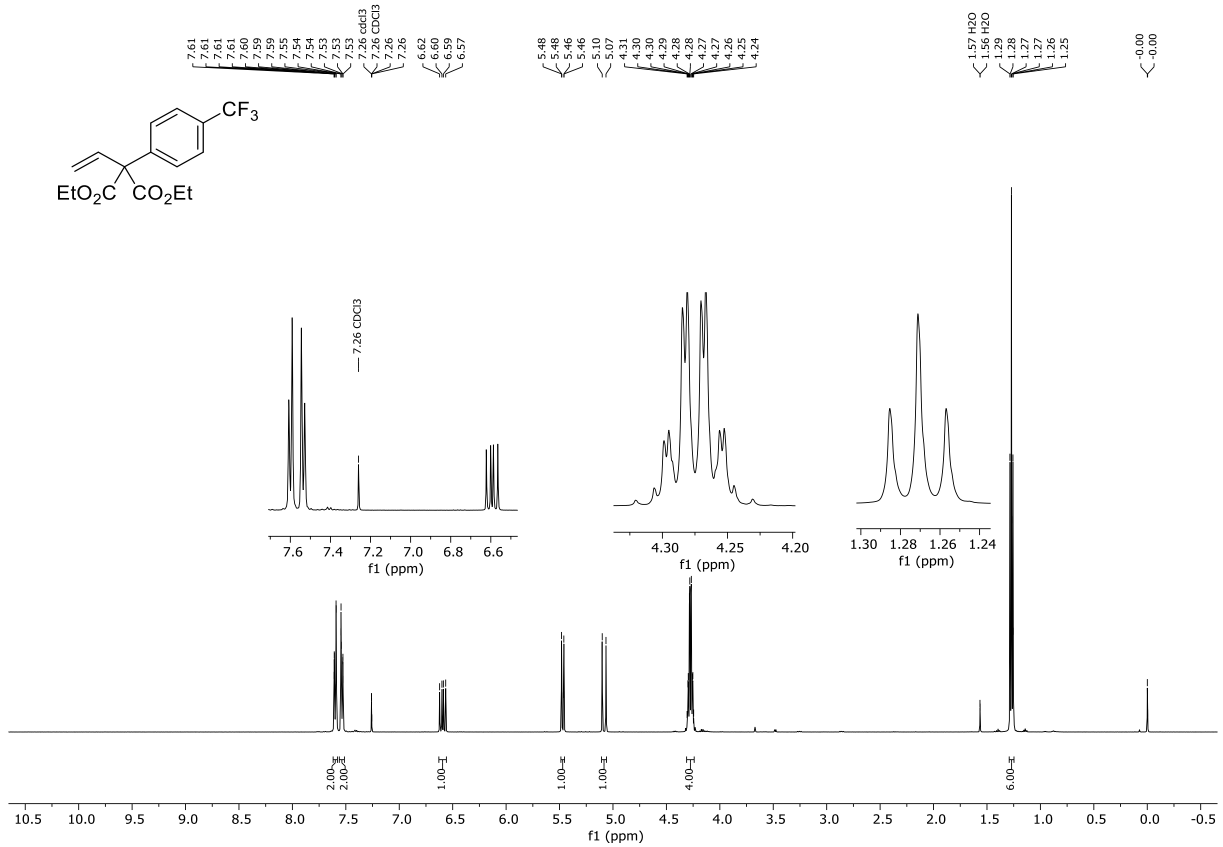

^{13}C NMR (CDCl3, 126 MHz)

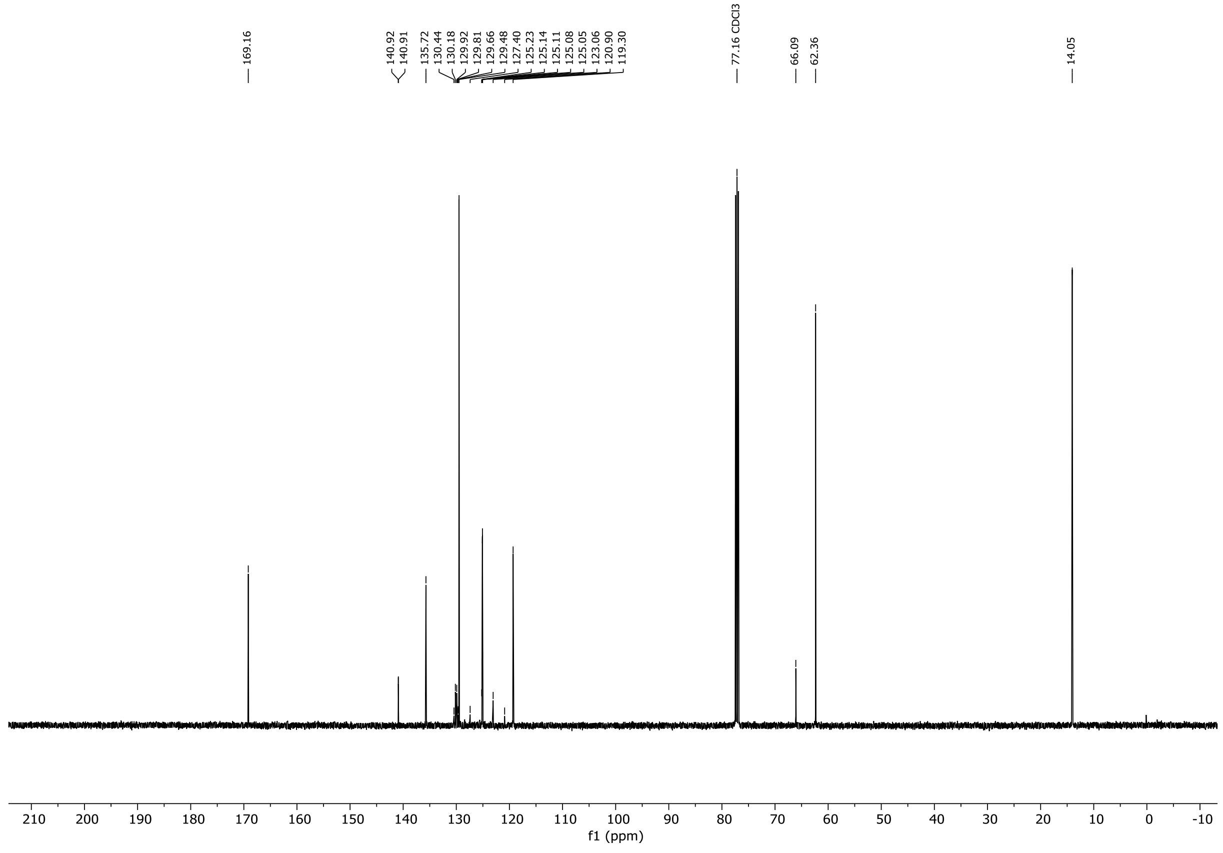

^{19}F NMR ($CDCl_3$, 470 MHz)

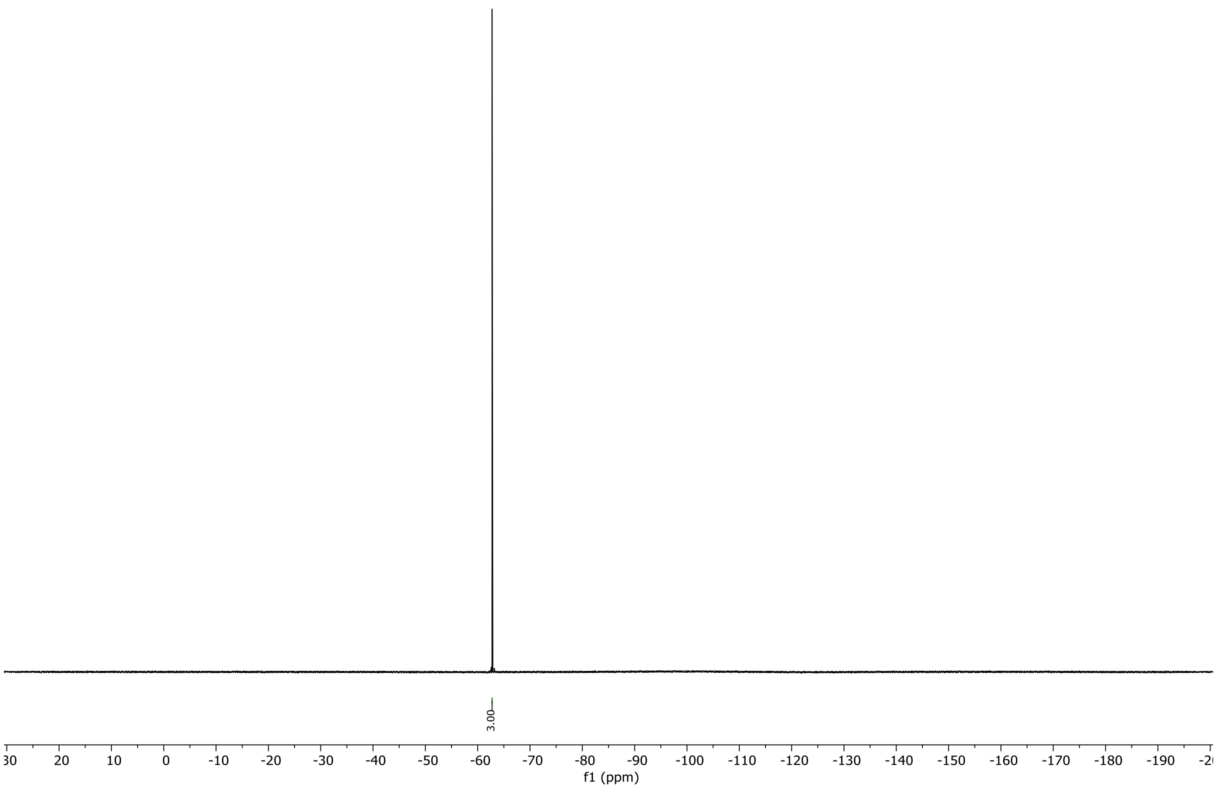

dodecyl 4-bromobutanoate, S11

^{1}H NMR (CDCl$_3$, 500 MHz)

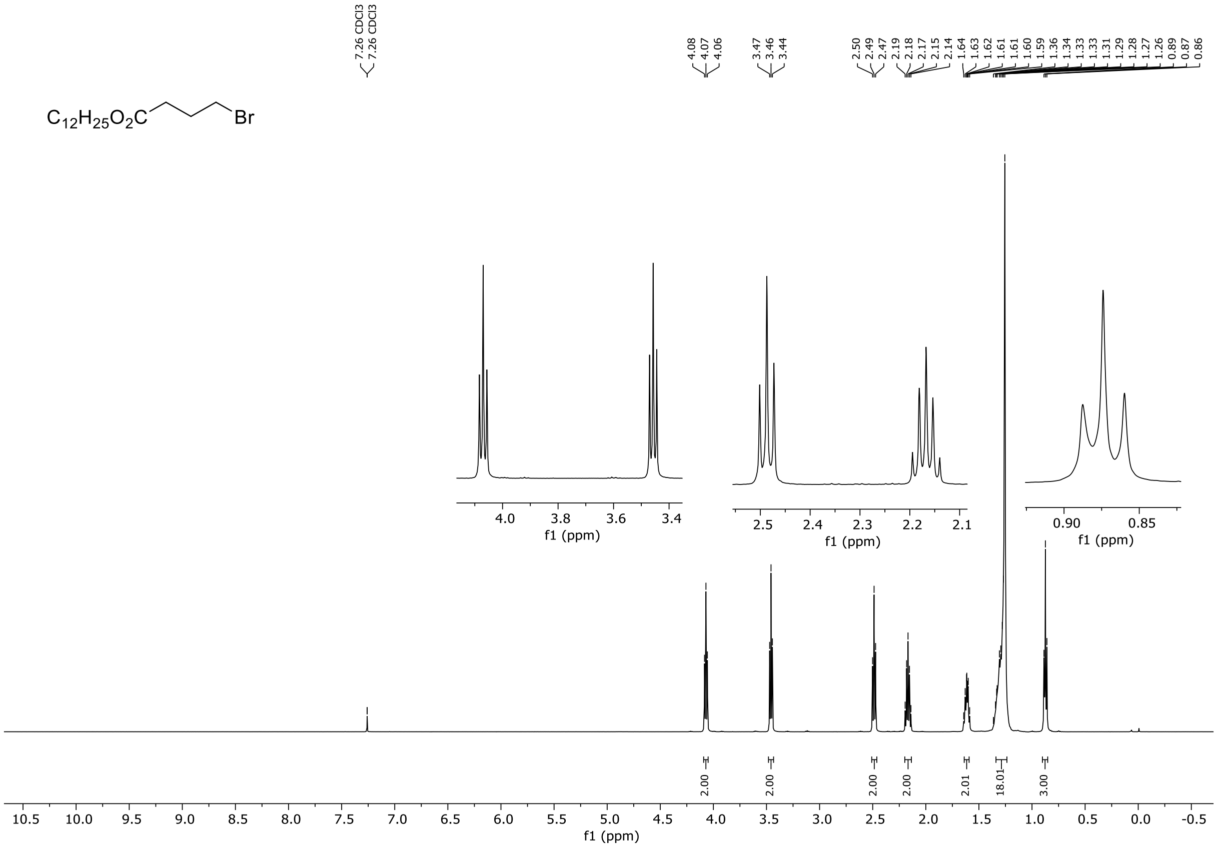

^{13}C NMR (CDCl$_3$, 126 MHz)

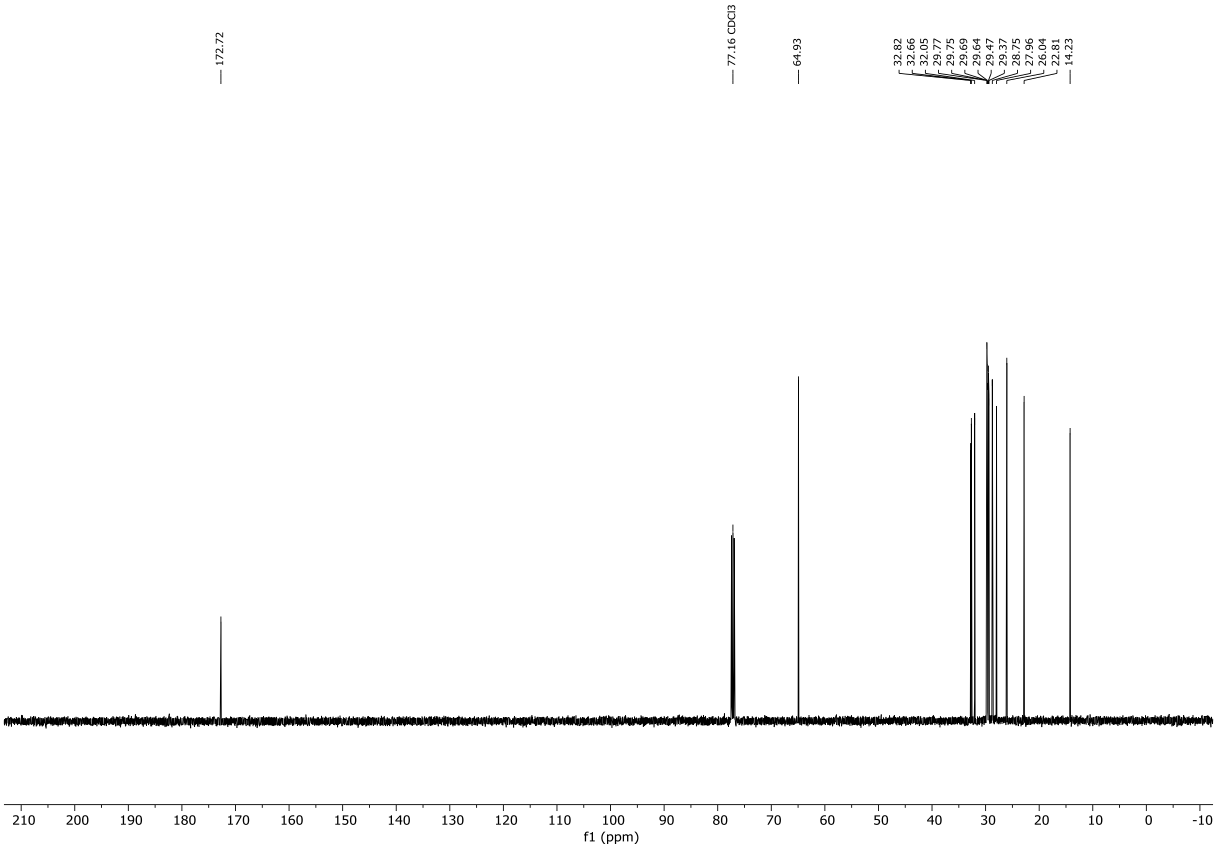

Supplement: Supplementary file 1 — ja4c02682_si_001.pdf [file ja4c02682_si_001.pdf]
